# Large Temperature Dependence of Large Kinetic Isotope Effects of Multistep Hydride Reduction of *p*‑Chloranil by NADH Models in Acetonitrile: Proton Tunneling within Loose Radical Ion-Pairs

**DOI:** 10.1021/acs.joc.5c02302

**Published:** 2025-11-13

**Authors:** Bibesh Pokhrel, Jessica Sager, Pratichhya Adhikari, Grishma Singh, Bikram Dhakal, Yun Lu

**Affiliations:** Department of Chemistry, 33140 Southern Illinois University Edwardsville, Edwardsville, Illinois 62026, United States

## Abstract

The temperature dependence of H/D kinetic isotope effects (KIEs) was determined for electron-transfer initiated multistep hydride-transfer reactions of NADH models in acetonitrile to investigate our hypothesis that more rigid systems exhibit smaller isotopic activation energy differences (Δ*E*_a_ = *E*_aD_ – *E*_aH_). Proton-tunneling occurs in close contact or partially solvent-separated radical ion-pair intermediates. Large KIEs and large Δ*E*_a_’s were observed, in contrast to the relatively small KIEs and Δ*E*_a_’s typically observed for one-step mechanisms where hydride-tunneling occurs within much tighter charge-transfer complexes. Results support the hypothesis.

## Graphical abstract

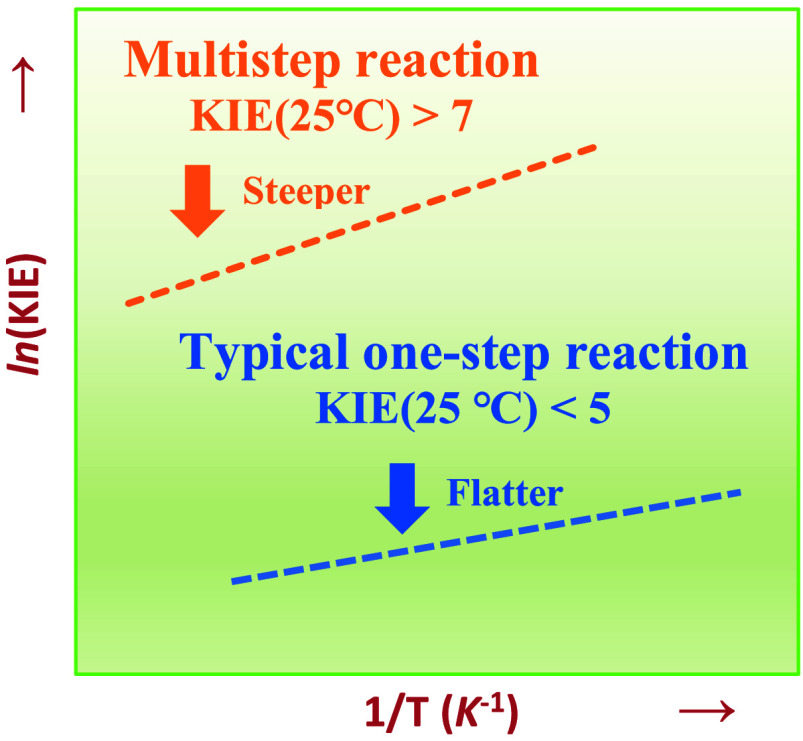

Current textbooks explain deuterium (D) kinetic isotope effects (KIEs) using the semiclassical transition state (TS) theory. KIEs originate from the isotopic zero-point energy difference at the reactant versus TS states. Within the semiclassical theory, KIEs range from 2 to 7, and the isotopic activation energy difference (Δ*E*_a_ = *E*_aD_ – *E*_aH_), which reflects temperature (*T*) dependence of KIEs, falls in between 1.0 and 1.2 kcal/mol. Observations outside of the limits have been frequently observed leading to the proposal of a nonclassical H-tunneling mechanism, where H tunnels through the classical barrier in light of its quantum mechanical wave property. Within the traditional Bell tunneling model, tunneling is suggested when Δ*E*_a_ exceeds 1.2 kcal/mol, disappears within the semiclassical range, and becomes significant again when Δ*E*_a_ is below this range, especially when it approaches zero.^1^

Many observations could not be explained by the Bell model. One prominent example is the frequently observed shift from *T*-independent KIEs (Δ*E*_a_ = 0) in enzymes to *T*-dependent KIEs in their mutants (Δ*E*_a_ > 0).^2–20^ In the mutants, the Δ*E*_a_ increases, through and beyond the semiclassical limit, as mutations appear to gradually loosen the reaction complexes. To interpret these findings, the vibration-assisted activated hydrogen tunneling (VA-AHT) model was used.^2,7,21–23^ This full-tunneling model involves two orthogonal activation processes: (1) heavy atom motions bring the donor (D-H) and acceptor (A) into a tunneling-ready state (TRS = [D-H ↔ H-A]^‡^) where H-wave functions overlap at the degenerate reactant (D-H) and product (H-A) states, *i.e*., tunneling; and (2) the more rapid heavy atom motions sample short donor–acceptor distances (DADs) for efficient tunneling. Of these two processes, only the DAD sampling for tunneling is isotope-sensitive. This is because the heavier D isotope of shorter wavelength requires shorter DADs to effectively tunnel than the H isotope. As a result, the activation energy for D-tunneling is higher (*E*_aD_ > *E*_aH_). However, when the system becomes rigid enough so that the DAD sampling is not affected by the thermal energy, *E*_aD_ and *E*_aH_ converge, resulting in Δ*E*_a_ = 0. Within this framework, the *T*-independent KIEs in enzymes are explained in terms of the constructive strong protein vibrations, also called promoting vibrations,^24,25^ which compress the donor and acceptor centers, restricting DAD fluctuations.^6,26^ When enzymes are mutated, these vibrational modes are disrupted, leading to greater energy requirements for sampling of short DADs, which is especially for D. As a result, Δ*E*_a_ increases. The VA-AHT model that involves ground-state H-tunneling has been claimed to be able to explain all of the KIE observations.^7^

These explanations emphasize a potential catalytic role for fast, local protein vibrations in facilitating short DAD sampling, a topic that has generated debate in the field.^27,28^ The core of these controversial explanations is whether the *T*-dependence of KIEs reliably reflects the *T*-dependence of DAD fluctuations.^29^ Simulations using ensemble-averaged variational TS theory combined with a multidimensional H-tunneling model describe the temperature effect on KIEs of the hydride transfer in a dihydrofolate reductase as arising from its effect on microscopic mechanisms, specifically, the position of the TS and the shape of the energy barrier.^30^ Meanwhile, it has been argued that the VA-AHT model was originally developed to explain KIEs in nonadiabatic hydrogen-atom transfer reactions and may not be appropriate for the more adiabatic hydride- and proton-transfer reactions.^21,31^ Nonetheless, the relationship between DAD distributions and Δ*E*_a_ has been observed over the enzymes that catalyze all three types of H-transfers (H^**•**^, H^+^, and H^–^). This suggests that the relationship likely exists but the rate expression may take different mathematical forms depending on the type of H-transfer, some of which may involve transfer to the excited state of products.^31^ Further investigation of the DAD−Δ*E*_a_ relationship could not only enhance our understanding of the role of protein dynamics in DAD sampling for catalysis, but also provide insights for evaluating current or developing future H-tunneling models.

We have initiated a project to investigate the relationship between DAD distributions and Δ*E*_a_ for hydride-transfer reactions in solution. Our hypothesis, based on enzyme observations and corresponding explanations within the VA-AHT model, is that a more rigid system with narrowly distributed DADs gives rise to a smaller Δ*E*_a_ value.^32^ To test this, we have primarily examined hydride-transfer reactions involving NADH/NAD^+^ models in solution.^32–40^ These systems are ideal for three reasons: (1) NADH/NAD^+^-dependent redox enzymes have been extensively studied in this context, allowing for direct comparisons that may provide insight into the role of protein dynamics in DAD sampling; (2) much evidence have shown that reactions of NADH/NAD^+^ and their models involve H-tunneling processes;^2,3,7,16,32–34,36–52^ and (3) the hydride-transfer occurs within charge-transfer (CT) complexes, where DAD and system rigidity can be modulated through structural modifications. We have explored the electronic and steric effects,^32,33,37,39,40^ solvent effects,^35,36^ as well as remote heavy-group vibrational effects^38^ on the *T*-dependence of KIEs for the NADH/NAD^+^ model reactions. The observed trends support the proposed DAD−Δ*E*_a_ relationship, with findings implicating that the strength of CT complexation primarily controls the system rigidity and T-dependence of KIEs.

It is known that hydride-transfer reactions involving NADH/NAD^+^ model compounds could also proceed via a single electron-transfer initiated multistep mechanism when the hydride acceptor is a sufficiently strong one-electron oxidant, especially when electron transfer and the subsequent H transfer attack two different sites of the acceptor systems. This alternative pathway involves sequential electron–proton–electron (e-H^+^-e) or electron–hydrogen atom (e–H^•^) transfer steps.^53,54^ In such cases, initial electron transfer occurs within the CT complex of the donor (DH) and acceptor (A), leading to generation of a radical ion-pair (DH^+•^A^–•^), followed by either proton or hydrogen atom transfer. Unlike the compact CT complexes for direct hydride-transfer, the resulting radical ion-pair is likely to be partially separated by solvation, producing a relatively loosely associated ion-pair. This multistep mechanism is expected to involve longer DADs for the subsequent H^+^- or H^•^-transfer from which KIE originates, especially when the individual radical ions are stable enough so that they could escape from the solvent cage for the ion-pair complex. According to our hypothesis, such increased DADs would lead to a greater *T*-dependence of KIEs.

In this work, we study the effect of multistep hydride-transfer mechanisms on the *T*-dependence of KIEs to further investigate the hypothesized DAD−Δ*E*_a_ relationship. *p*-Chloranil (2,3,5,6-tetrachlorobenzoquinone, Cl_4_Q) was chosen as the hydride acceptor for its well-documented reactivity with NADH models via a multistep e-H^+^-e transfer pathway.^55–60^ The first NADH model selected is 10-methylacridine (MAH). The kinetics of its reaction with Cl_4_Q has been reported in acetonitrile at 25 °C.^55^ Importantly, the free radical cations of MAH and related 9-substitueted derivative have been captured by UV–vis and ESR spectroscopy in their acid-promoted reactions with a benzoquinone structure and a Fe^IV^ = O complex in acetonitrile.^55,61^ This indicates that MAH^+•^ is stable enough to escape from the solvent cage, leading to a relatively loose, partially solvent-separated ion-pair intermediate, which contrasts sharply with the tight charge-transfer (CT) complexes in one-step hydride-transfer mechanisms. This distinction allows us to better probe the Δ*E*_a_ differences. The second model chosen is Hantzsch ester (HEH). It is known that the heterolytic acidic dissociation enthalpy of HEH^+•^ (Δ*H*_het_(HEH^+•^) = 4.5 kcal/mol in acetonitrile) is significantly smaller than that of MAH^+•^ (9.2 kcal/mol) (*i.e*., by about 3.5 p*K*_a_ units).^62^ Therefore, HEH^+•^ is more reactive, as compared to MAH^+•^, so as to have less chance to escape the solvent cage leading to less solvent-separated or even close contact ion-pair intermediate. Therefore, the expectations following our hypothesis are (1) the reaction of MAH with Cl_4_Q would produce a Δ*E*_a_ value larger than those of general one-step hydride-transfer reactions; and (2) the Δ*E*_a_ is larger than that for the reaction of HEH.

Other NADH models selected are 10-N-R-substituted acridines (RAH), where R = H (HAH), *n*-C_3_H_7_ (PAH), and PhCH_2_ (BAH). These donors along with MAH, except HAH, have been used to test whether the remote heavy group vibrations affect the DAD fluctuations and Δ*E*_a_’s of the one-step hydride-transfer reactions.^38^ In that work, our expectation is that a heavier group would slow down the system constructive vibrations and broaden the DAD distributions thereby increasing the Δ*E*_a_. Although no significant effect was observed, in this work, we will investigate whether there is such effect in the multistep mechanism.

These reactions in acetonitrile are shown in Figure 1. The hydride affinities are listed in Figure 1 as well to calculate free energy changes (Δ*G°*(H_T_^–^)) of the overall hydride-transfer reactions.^37,39^

**Figure 1:**
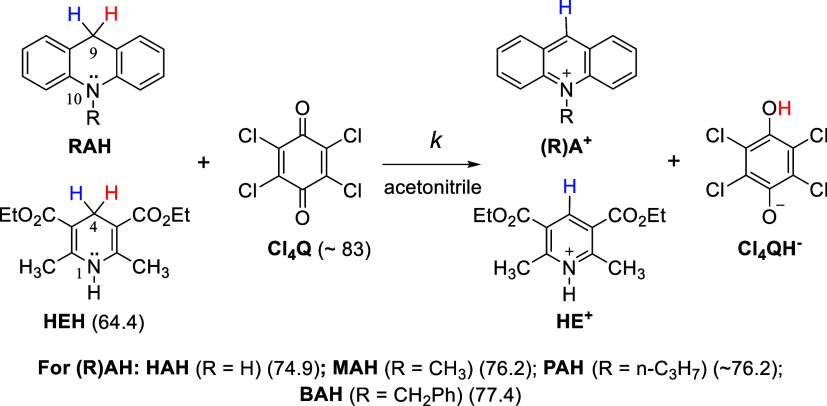
Apparent hydride-transfer reactions from RAH and HEH to Cl_4_Q. Numbers in parentheses are the hydride affinity values of their oxidized forms in acetonitrile (in kcal/mol).^63^

The stoichiometry of the reactions of benzoquinones with NADH model compounds has been investigated.^56–58,61^ It was found that two molecules of hydride donor react with three molecules of benzoquinone. For example, 2MAH + 3Cl_4_Q → 2MA^+^ + 2Cl_4_Q^–**•**^ + Cl_4_QH_2_. The three-step reactions are shown in order as

$$\begin{array}{l} \mathrm{MAH}+{\mathrm{Cl}}_{4}Q\to {\mathrm{MA}}^{+}+{\mathrm{Cl}}_{4}{\mathrm{QH}}^{-}\left(\mathrm{slow}\right)\\ {\mathrm{Cl}}_{4}{\mathrm{QH}}^{-}+{\mathrm{Cl}}_{4}Q\to {\mathrm{Cl}}_{4}{\mathrm{QH}}^{•}+{\mathrm{Cl}}_{4}Q^{-•}\left(\mathrm{fast}\right)\\ 2{\mathrm{Cl}}_{4}{\mathrm{QH}}^{•}\to {\mathrm{Cl}}_{4}Q+{\mathrm{Cl}}_{4}{\mathrm{QH}}_{2}\left(\mathrm{fast}\right) \end{array}$$

The first second-order rate-determining apparent hydride-transfer step is the one that we study for the e-H^+^-e sequential transfer mechanism as schematically shown below.
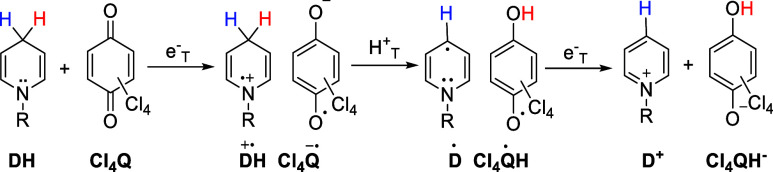


Figure 2 shows the T-dependence of KIEs for the reactions of Cl_4_Q with MAH and HEH, as well as that for a reported typical direct hydride-transfer reaction (Δ*E*_a_ = 0.96 kcal/mol^33,37^ ) for comparison. The last hydride-transfer system was chosen as it has comparable Δ*G°*(H_T_^–^) to those studied in this work. The Δ*G°*(H_T_^–^)’s, the second-order rate constants (*k*’s) and KIEs at 25 °C, as well as Δ*E*_a_’s for the reactions of Cl_4_Q are listed in Table 1. Note that we determined the T-dependence of KIEs for the reaction of MAH with either MAH or Cl_4_Q being excess. The pseudo first-order rate constant (*k* ^pfo^) determined with excess Cl_4_Q is 2/3 of the *k* ^pfo^ determined with excess MAH at the same concentration, consistent with above stoichiometry. The *k* and Δ*E*_a_ values determined from both methods are the same within the experimental error (see footnotes b and c in Table 1).

**Figure 2:**
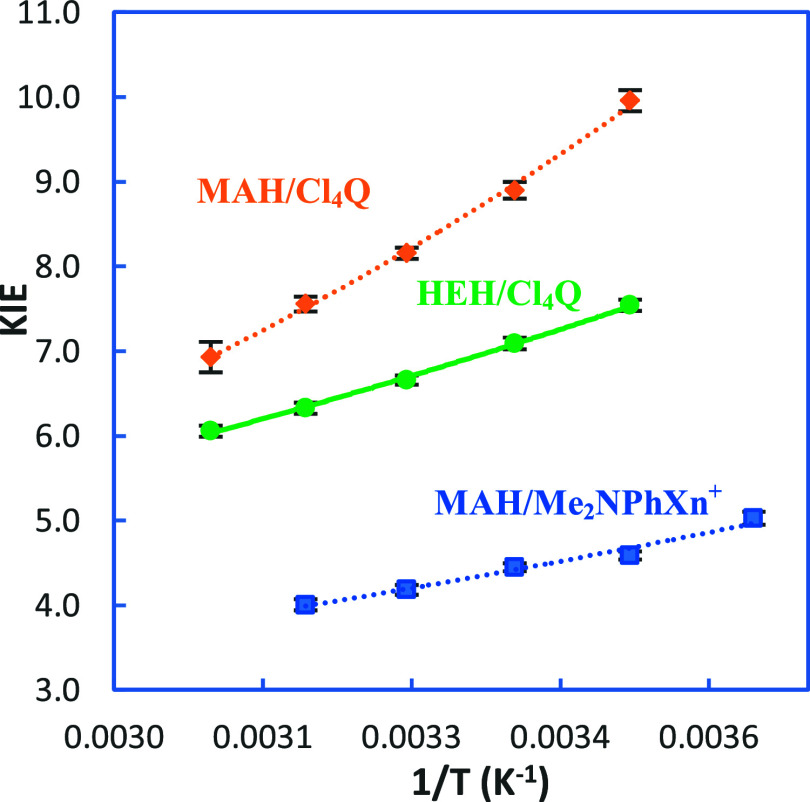
Arrhenius plots of KIEs for the reactions of MAH versus HEH with Cl_4_Q (from 15 to 55 °C) as well as a typical one-step reaction of MAH with 9-(*p*-*N*,*N*-dimethylphenyl)­xanthylium ion (Me_2_NPhXn^+^BF_4_^–^) in acetonitrile (Δ*G°*(H_T_^–^) = −10.5 kcal/mol, KIE = 5.0–4.1 from 5 to 45 °C, Δ*E*_a_ = 0.96 kcal/mol^33,37^ ). Lines represent nonlinear regression using an Arrhenius-type exponential equation (KIE vs. EXP­(1/T)).

**Table 1:** Δ*G°*(H_T_^–^)’s, KIEs and Δ*E*_a_’s for the Multistep Hydride-Transfer Reactions of Cl_4_Q in Acetonitrile^a^,^b^

reaction system	Δ*G°*(H_T_^–^) (kcal/mol)	*k*_H_^25°C^ (M^–1^s^–1^)	KIE^25°C^	Δ*E*_a_ (kcal/mol)
-HAH	–8.1	5.17(0.04) × 10	9.20 (0.10)	1.64 (0.08)
-MAH	–6.8	1.89(0.01) × 10	8.90 (0.10)	1.67 (0.13)
-MAH^c^		1.92(0.01) × 10	8.53 (0.16)	1.70 (0.13)
-PAH^c^	∼ – 6.8	6.77(0.05) × 10	8.30 (0.12)	1.65 (0.15)
-BAH	–5.6	1.23(0.01) × 10	8.89 (0.09)	1.54 (0.12)
-HEH	–18.6	1.76(0.01) × 10^2^	7.09 (0.07)	1.04 (0.05)

a Numbers in parathesis are pooled standard deviations (standard deviations calculated from the average values of three independent experiments are included in SI, which are comparable to the pooled standard deviations).

b *k* ^pfo^ determined with Cl_4_Q being excess, unless otherwise noted (see SI for concentration conditions).

c Donor is in excess.

Since HEH possesses an acidic H at the N-1 position, the HEH^+•^ intermediate, if any, could also potentially lose the proton in the multistep mechanisms. To exclude the possibility, we determined the N–H/D KIE for the reaction in acetonitrile. The KIE was found to be 1.01 (±0.01) (at 25 °C, see SI). This secondary KIE result supports the mechanism where only the 4-H transfers in the rate-determining step.

The Δ*E*_a_’s for the reactions of RAHs (1.5–1.7 kcal/mol) are significantly larger than those typically observed for one-step hydride-transfer reactions between two carbons. The latter reactions reported with a wide range of Δ*G°* from −3 to −40 kcal/mol have shown generally KIE < 5, and Δ*E*_a_ = ∼0 to 1.35 kcal/mol (as exemplified in Figure 1).^32,33,35,37–39,49^ The KIE difference is consistent with the general trend that proton-transfer reactions show larger KIEs than hydride-transfer.^21^ The large Δ*E*_a_ values correspond with loose radical ion-pair complexes of larger DADs, as expected due to partial escape of RAH^+•^ from the solvent cage for the complexes. The (large) DAD—(large) Δ*E*_a_ relationship is further confirmed by comparison with the HEH reaction, which has a smaller Δ*E*_a_ value (1.04 kcal/mol) and where the proton-transfer is expected to take place in a less solvent-separated or close contact ion-pair of smaller DADs.

In all RAH reactions studied, both KIEs and Δ*E*_a_’s appear largely unaffected by the remote heavy 10-R substitutions. This is similar to the insignificant R effect observed in the corresponding one-step hydride-transfer reactions.^38^ Therefore, in both mechanisms, the remote substitutions which have similar electronic properties and are expected to introduce systematically different vibrational effects do not significantly affect the DAD distributions and the Δ*E*_a_ values. Therefore, it would be the strength of the CT complexation or the electrostatic attraction of the oppositely charged radical ions that largely determines the system rigidity and the Δ*E*_a_ magnitudes.^38^

Furthermore, the larger DAD in the reactions of RAHs with Cl_4_Q may also explain the significantly larger KIE values (8.30 to 9.20 at 25 °C) than that for the reaction of HEH (7.09). The VA-AHT model also predicts that a larger DAD gives rise to a larger KIE value and this trend can become evident when additional factors affecting the KIE within the model are fixed.^3,7,21,39^ While it is challenging to fully evaluate the contribution of the additional factors across different systems, we found this explanation may also be applied to explain the observed larger proton KIEs in the multistep reactions of Cl_4_Q than hydride KIEs in the one-step process in which hydride-transfer occurs in CT complexes with smaller DADs. It should be pointed out that the literature explanation of the small KIEs observed in hydride-transfer reactions of NADH models uses the bent TS structures at which the isotopic zero-point energy difference is relatively large.^42^ While this explanation is based on the semiclassical TS theory, exactly how structures affect KIEs through isotopic wave function overlap differences in a H-tunneling mechanism remains mysterious.^39^

In summary, *T*-dependence of KIEs for the multistep hydride-transfer reactions of Cl_4_Q with NADH models were determined to investigate our hypothesis that a more rigid system gives rise to a smaller Δ*E*_a_ value. The KIE arises from the proton-transfer step within a relatively loose radical ion-pair complex intermediate. Our findings show a large *T*-dependence of large KIEs. This is in sharp contrast to the behavior seen in direct hydride-transfer reactions within a tightly bound CT complex. Furthermore, in a multistep mechanism, if the radical ions are highly reactive that they have limited opportunity to escape the solvent cage for their complexes, a smaller Δ*E*_a_ may still result.

The rigidity−Δ*E*_a_ relationship trend found among the multistep reactions (RAH vs HEH) is consistent with the trend observed in our studies of direct hydride-transfer reactions where the CT complex rigidity is affected by structural modifications. In both cases, a tighter system appears to consistently correspond with a smaller Δ*E*_a_ value. Taken together, a radical ion-pair complex for multistep hydride-transfer may be treated as an extreme case of the covalently bound CT complex from a one-step mechanism.^53^ When charge transfers to an extent so as to form a radical ion-pair, DAD could increase. As a result, Δ*E*_a_ could increase from a one-step to a multistep mechanism. Therefore, by comparing NADH model reactions with different mechanisms and thus different DADs for *nucleus* tunneling, we explored the DAD−Δ*E*_a_ relationship. Our results support the relationship within our overall hypothesis that likely works for all three kinds of H-transfers, as have already been observed in various enzymes.

## Supplementary Material



## Data Availability

The data underlying this study are available in the published article and its online Supporting Information.

## Notes

Synthesis and kinetic measurement procedures, *T*-dependence of KIEs plots for the reactions of HAH, PAH and BAH, primary kinetic data (PDF)

## References

[1] Sharma S. C., Klinman J. P. (2008). Experimental Evidence
for Hydrogen Tunneling when the
Isotopic Arrhenius Prefactor (AH/AD) is Unity. J. Am. Chem. Soc..

[2] Nagel Z. D., Klinman J. P. (2010). Update 1 of: Tunneling and dynamics in enzymatic hydride
transfer. Chem. Rev..

[3] Pudney C. R., J L., Sutcliffe M. J., Hay S., Scrutton N. S. (2010). Direct Analysis
of Donor-Acceptor Distance and Relationship to Isotope Effects and
the Force Constant for Barrier Compression in Enzymatic H-Tunneling
Reactions. J. Am. Chem. Soc..

[4] Roston D., Cheatum C. M., Kohen A. (2012). Hydrogen Donor-Acceptor Fluctuations
from Kinetic Isotope Effects: A Phenomenological Model. Biochemistry.

[5] Stojković V., Perissinotti L., Willmer D., Benkovic S., Kohen A. (2012). Effects of
the donor acceptor distance and dynamics on hydride tunneling in the
dihydrofolate reductase catalyzed reaction. J. Am. Chem. Soc..

[6] Klinman J. P., Kohen A. (2013). Hydrogen Tunneling Links Protein Dynamics to Enzyme Catalysis. Annu. Rev. Biochem..

[7] Kohen A. (2015). Role of Dynamics
in Enzyme Catalysis: Substantial vs. Semantic Controversies. Acc. Chem. Res..

[8] Pagano P., Guo Q., Ranasinghe C., Schroeder E., Robben K., Häse F., Ye H., Wickersham K., Aspuru-Guzik A., Major D. T., Gakhar L., Kohen A., Cheatum C. M. (2019). Oscillatory Active-Site Motions Correlate
with Kinetic Isotope Effects in Formate Dehydrogenase. ACS Catal..

[9] Knapp M. J., Klinman J. P. (2002). Environmentally coupled hydrogen
tunneling. Linking
catalysis to dynamics. Eur. J. Biochem..

[10] Knapp M. J., Rickert K., Klinman J. P. (2002). Temperature-dependent
isotope effects
in soybean lipoxygenase-1: Correlating hydrogen tunneling with protein
dynamics. J. Am. Chem. Soc..

[11] Agrawal N., Hong B., Mihai C., Kohen A. (2004). Vibrationally Enhanced
Hydrogen Tunneling in the *Escherichia coli* Thymidylate Synthase Catalyzed Reaction. Biochemistry.

[12] Nagel Z. D., Meadows C. W., Dong M., Bahnson B. J., Klinman J. P. (2012). Active
Site Hydrophobic Residues Impact Hydrogen Tunneling Differently in
a Thermophilic Alcohol Dehydrogenase at Optimal versus Nonoptimal
Temperatures. Biochemistry.

[13] Francis K., Sapienza P., Lee A., Kohen A. (2016). The Effect of Protein
Mass Modulation on Human Dihydrofolate Reductase. Biochemistry.

[14] Geddes A., Paul C. E., Hay S., Hollmann F., Scrutton N. S. (2016). Donor–Acceptor
Distance Sampling Enhances the Performance of “Better than
Nature” Nicotinamide Coenzyme Biomimetics. J. Am. Chem. Soc..

[15] Romero E., Ladani S. T., Hamelberg D., Gadda G. (2016). Solvent-Slaved Motions
in the Hydride Tunneling Reaction Catalyzed by Human Glycolate Oxidase. ACS Catal..

[16] Howe G. W., van der Donk W. A. (2019). Temperature-Independent Kinetic Isotope
Effects as
Evidence for a Marcus-like Model of Hydride Tunneling in Phosphite
Dehydrogenase. Biochemistry.

[17] Hu S., Offenbacher A. R., Thompson E. M., Gee C. L., Wilcoxen J., Carr C. A. M., Prigozhin D. M., Yang V., Alber T., Britt R. D., Fraser J. S., Klinman J. P. (2019). Biophysical Characterization
of a Disabled Double Mutant of Soybean Lipoxygenase: The “Undoing”
of Precise Substrate Positioning Relative to Metal Cofactor and an
Identified Dynamical Network. J. Am. Chem. Soc..

[18] Mhashal A. R., Major D. T. (2021). Temperature-Dependent
Kinetic Isotope Effects in R67
Dihydrofolate Reductase from Path-Integral Simulations. J. Phys. Chem. B.

[19] Singh P., Vandemeulebroucke A., Li J., Schulenburg C., Fortunato G., Kohen A., Hilvert D., Cheatum C. M. (2021). Evolution
of the Chemical Step in Enzyme Catalysis. ACS
Catal..

[20] Wang Z., Singh P. N., Czekster M. C., Kohen A., Schramm V. L. (2014). Protein
Mass-Modulated Effects in the Catalytic Mechanism of Dihydrofolate
Reductase: Beyond Promoting Vibrations. J. Am.
Chem. Soc..

[21] Klinman J. P. (2010). A new model
for the origin of kinetic hydrogen isotope effects. J. Phys. Org. Chem..

[22] Hay S., Sutcliffe M. J., Scrutton N. S. (2007). Promoting motions in enzyme catalysis
probed by pressure studies of kinetic isotope effects. Proc. Natl. Acad. Sci. U.S.A..

[23] Loveridge, E. J. ; Allemann, R. Direct Methods for the Analysis of Quantum-Mechanical Tunneling: Dihydrofolate Reductase. In Quantum Tunnelling in Enzyme-Catalyzed Reactions; Scrutton, N. S. ; Allemann, R. K. , Eds.; RSC Publishing, 2009; pp 179–198.

[24] Schwartz S. D. (2023). Protein
Dynamics and Enzymatic Catalysis. J. Phys. Chem.
B.

[25] Roy R. K., Antoniou D., Schwartz S. D. (2025). The Shaping of Enzymatic Free Energy
Barriers through the Creation of Rate-Promoting Vibrations via Inter-Residue
Cross-Talk on Multiple Time Scales. J. Phys.
Chem. B.

[26] Klinman J. P., Offenbacher A. R. (2018). Understanding Biological Hydrogen Transfer Through
the Lens of Temperature Dependent Kinetic Isotope Effects. Acc. Chem. Res..

[27] O’Ferrall R. A. M. (2010). Introduction
to a symposium in print on tunnelling. J. Phys.
Org. Chem..

[28] Klinman J. P., Miller S. M., Richards N. G. J. (2025). A Foundational
Shift in Models for
Enzyme Function. J. Am. Chem. Soc..

[29] Kamerlin S. C. L., Warshel A. (2010). An analysis of all
the relevant facts and arguments
indicates that enzyme catalysis does not involve large contributions
from nuclear tunneling. J. Phys. Org. Chem..

[30] Pu J., Ma S., Gao J., Truhlar D. G. (2005). Small temperature dependence of the
kinetic isotope effect for the hydride transfer reaction catalyzed
by *Escherichia coli* dihydrofolate reductase. J. Phys. Chem. B.

[31] Hammes-Schiffer S. (2025). Explaining
Kinetic Isotope Effects in Proton-Coupled Electron Transfer Reactions. Acc. Chem. Res..

[32] Lu Y., Wilhelm S., Bai M., Maness P., Ma L. (2019). Replication
of the Enzymatic Temperature Dependency of the Primary Hydride Kinetic
Isotope Effects in Solution: Caused by the Protein Controlled Rigidity
of the Donor-Acceptor Centers?. Biochemistry.

[33] Maness P., Koirala S., Adhikari P., Salimraftar N., Lu Y. (2020). Substituent Effects on Temperature
Dependence of Kinetic Isotope
Effects in Hydride-Transfer Reactions of NADH/NAD+ Analogues in Solution:
Reaction Center Rigidity Is the Key. Org. Lett..

[34] Bai M., Koirala S., Lu Y. (2021). Direct Correlation
Between Donor-Acceptor
Distance and Temperature Dependence of Kinetic Isotope Effects in
Hydride-Tunneling Reactions of NADH/NAD+ Analogues. J. Org. Chem..

[35] Adhikari P., Song M., Bai M., Rijal P., DeGroot N., Lu Y. (2022). Solvent Effects on the Temperature Dependence of Hydride Kinetic
Isotope Effects: Correlation to the Donor–Acceptor Distances. J. Phys. Chem. A.

[36] Bai M., Rijal P., Salarvand S., Lu Y. (2023). Correlation of Temperature
Dependence of Hydride Kinetic Isotope Effects with Donor-Acceptor
Distances in Two Solvents of Different Polarities. Org. Biol. Chem..

[37] Beach A., Adhikari P., Singh G., Song M., DeGroot N., Lu Y. (2024). Structural Effects
on the Temperature Dependence of Hydride Kinetic
Isotope Effects of the NADH/NAD+ Model Reactions in Acetonitrile:
Charge-Transfer Complex Tightness Is a Key. J. Org. Chem..

[38] Singh G., Austin A., Bai M., Bradshaw J., Hammann B. A., Kabotso D. E. K., Lu Y. (2024). Study of the Effects of Remote Heavy
Group Vibrations on the Temperature Dependence of Hydride Kinetic
Isotope Effects of the NADH/NAD+ Model Reactions. ACS Omega.

[39] Austin A., Sager J., Phan L., Lu Y. (2025). Structural Effects
on the Hydride-Tunneling Kinetic Isotope Effects of NADH/NAD+ Model
Reactions: Relating to the Donor–Acceptor Distances. J. Org. Chem..

[40] Bai M., Singh G., Lu Y. (2025). Rigidity Analysis of Hydride Tunneling
Ready States from Secondary Kinetic Isotope Effects and Hammett Correlations:
Relating to the Temperature Dependence of Kinetic Isotope Effects. J. Phys. Org. Chem..

[41] Huskey W. P., Schowen R. L. (1983). Reaction-coordinate
tunneling in hydride transfer reactions. J.
Am. Chem. Soc..

[42] Powell M. F., Bruice T. C. (1983). Effect of isotope scrambling and tunneling on the kinetic
and product isotope effects for reduced nicotinamide adenine dinucleotide
model hydride transfer reactions. J. Am. Chem.
Soc..

[43] Kreevoy M. M., Ostovic D., Truhlar D. G., Garrett B. C. (1986). Phenomenological
manifestations of large-curvature tunneling in hydride-transfer reactions. J. Phys. Chem. A.

[44] Kim Y., Kreevoy M. M. (1992). The experimental
manifestations of corner-cutting tunneling. J. Am. Chem. Soc..

[45] Han
Lee I.-S., Jeoung E. H., Kreevoy M. M. (2001). Primary Kinetic
Isotope Effects on Hydride Transfer from 1,3-Dimethyl-2-phenylbenzimidazoline
to NAD+ Analogues. J. Am. Chem. Soc..

[46] Kil H. J., Lee I.-S. H. (2009). Primary Kinetic Isotope Effects on Hydride Transfer
from Heterocyclic Compounds to NAD + Analogues. J. Phys. Chem. A.

[47] Schowen, R. L. The strengths and Weaknesses of Model Reactions for the Assesement if Tunneling in Enzymic Reactions. In Quantum tunnelling in enzyme catalyzed reactions; Allemann, R. ; Scrutton, N. , Eds.; Royal Society of Chemistry: London, UK, 2009; Vol., pp 291–313.

[48] Hammann B., Razzaghi M., Kashefolgheta S., Lu Y. (2012). Imbalanced Tunneling
Ready States in Alcohol Dehydrogenase Model Reactions: Rehybridization
Lags behind H-Tunneling. Chem. Commun..

[49] Liu Q., Zhao Y., Hammann B., Eilers J., Lu Y., Kohen A. (2012). A Model Reaction Assesses
Contribution of H-Tunneling and Coupled
Motions to Enzyme Catalysis. J. Org. Chem..

[50] Kashefolgheta S., Razzaghi M., Hammann B., Eilers J., Roston D., Lu Y. (2014). Computational Replication
of the Abnormal Secondary Kinetic Isotope
Effects in a Hydride Transfer Reaction in Solution with a Motion Assisted
H-Tunneling Model. J. Org. Chem..

[51] Maharjan B., Boroujeni M. R., Lefton J., White O. R., Razzaghi M., Hammann B. A., Derakhshani-Molayousefi M., Eilers J. E., Lu Y. (2015). Steric Effects
on the Primary Isotope Dependence of Secondary Kinetic
Isotope Effects in Hydride Transfer Reactions in Solution: Caused
by the Isotopically Different Tunneling Ready State Conformations?. J. Am. Chem. Soc..

[52] Derakhshani-Molayousefi M., Kashefolgheta S., Eilers J. E., Lu Y. (2016). Computational Replication
of the Primary Isotope Dependence of Secondary Kinetic Isotope Effects
in Solution Hydride-Transfer Reactions: Supporting the Isotopically
Different Tunneling Ready State Conformations. J. Phys. Chem. A.

[53] Bunting J. W. (1991). Merged
Mechanisms for Hydride Transfer from 1,4-dihydropyridines. Bioorg. Chem..

[54] Yasui S., Ohno A. (1986). Model studies with nicotinamide derivatives. Bioorg. Chem..

[55] Fukuzumi S., Kotani H., Lee Y.-M., Nam W. (2008). Sequential Electron-Transfer
and Proton-Transfer Pathways in Hydride-Transfer Reactions from Dihydronicotinamide
Adenine Dinucleotide Analogues to Non-heme Oxoiron­(IV) Complexes and
p-Chloranil. Detection of Radical Cations of NADH Analogues in Acid-Promoted
Hydride-Transfer Reactions. J. Am. Chem. Soc..

[56] Fukuzumi S., Ohkubo K., Tokuda Y., Suenobu T. (2000). Hydride Transfer from
9-Substituted 10-Methyl-9,10-dihydroacridines to Hydride Acceptors
via Charge-Transfer Complexes and Sequential Electron-Proton-Electron
Transfer. A Negative Temperature Dependence of the Rates. J. Am. Chem. Soc..

[57] Fukuzumi S., Koumitsu S., Hironaka K., Tanaka T. (1987). Energetic Comparison
between Photoinduced Electron-Transfer Reactions from NADH Model Compounds
to Organic and Inorganic Oxidants and Hydride-Transfer Reactions from
NADH Model Compounds to /?-Benzoquinone Derivatives. J. Am. Chem. Soc..

[58] Fukuzumi S., Nishizawa N., Tanaka T. (1984). Mechanism of Hydride Transfer from
an NADH Model Compound to p -Benzoquinone Derivatives. J. Org. Chem..

[59] Fukuzumi S., Lee Y. M., Nam W. (2021). Deuterium kineticisotopeeffectsasredox
mechanisticcriterions. Bull. Korean Chem. Soc..

[60] Ishikawat M., Fukuzumi S. (1990). Primary Kinetic lsotope
Effects on Acid-catalysed Reduction
of p-Benzoquinone Derivatives by an Acid-stable NADH Analogue. J. Chem. Soc., Faraday Trans..

[61] Yuasa J., Yamada S., Fukuzumi S. (2008). Detection of a radical cation of
an NADH analogue in two-electron reduction of a protonated p-quinone
derivative by an NADH analogue. Angew. Chem.,
Int. Ed..

[62] Shen G. B., Qian B. C., Fu Y. H., Zhu X. Q. (2022). Thermodynamics of
the elementary steps of organic hydride chemistry determined in acetonitrile
and their applications. Org. Chem. Front..

[63] Zhu X. Q., Deng F. H., Yang J. D., Li X. T., Chen Q., Lei N. P., Meng F. K., Zhao X. P., Han S. H., Hao E. J., Mu Y. Y. (2013). A classical
but new kinetic equation
for hydride transfer reactions. Org. Biomol.
Chem..

