# Supplementary material for: Large Temperature Dependence of Large Kinetic Isotope Effects of Multistep Hydride Reduction of p‑Chloranil by NADH Models in Acetonitrile: Proton Tunneling within Loose Radical Ion-Pairs
Source: J Org Chem. 2025 Nov 13;90(47):16912–7. doi: 10.1021/acs.joc.5c02302 (PMC12670409; doi:10.1021/acs.joc.5c02302)
Supplement: Supplementary file 1 [file jo5c02302_si_001.pdf]

Supporting Information

Large Temperature Dependence of Large Kinetic Isotope Effects of Multistep Hydride Reduction of *p*-Chloranil by NADH Models in Acetonitrile: Proton Tunneling within Loose Radical Ion-Pairs

Bibesh Pokhrel, Jessica Sager, Pratichhya Adhikari, Grishma Singh, Bikram Dhakal, Yun Lu*
Department of Chemistry, Southern Illinois University Edwardsville, Edwardsville, Illinois 62026, United States

yulu@siue.edu

General Procedures…………………………………………………………………………… S2
Kinetic Procedures…………………………………………………………………………… S2
Plots of temperature dependence of KIEs……………………………………………………….. S3
Raw rate constants (Tables S1-S7)……………………………………………………………… S4
References ……………………………………………………………………………………… S6
Data availability statement……………………………………………………………………… S6

General Procedures

Syntheses of the hydride donors of MAH, PAH, BAH and HEH and their dideuterio analogues have been reported by us.[1,2] The 9,10-dihydroacridine (HAH) and its dideuterated derivative (HAH-9,9-d,d) were synthesized by reduction of the 9(10H)-acridone (commercially available) with $LiAlH_4$ and $LiAlD_4$ in dry ether, respectively. Procedure is similar to that used to synthesize the dideuterated MAH, PAH and BAH for which the respective N-substituted acridones were reduced by $LiAlD_4$.[1] The product was purified by column chromatography and then recrystallization from 95% ethanol. M.p. 170.0-172.0. ^{1}H NMR ($CDCl_3$) δ(ppm): m (7.06-7.11, 4H), t (6.83-6.86, 2H), d (6.65-6.67, 2H), s (5.94, 1H), s (4.06, 2H). The HEH-ND was synthesized by gently refluxing the solution of the normal HEH in 2:1(v/v) THF/D_2O under nitrogen, repeated three times. The final product was recrystallized from CH_3OD for use. The deuterium content in all of the relevant compounds is generally > 98%.

HPLC grade acetonitrile was redistilled twice under nitrogen, with the presence of $KMnO_4$/K_2CO_3 (to remove the reducing impurity) and P_2O_5 (to remove water) in order, for kinetic measurements.

Kinetic procedures

Kinetic measurements were carried out by following the same procedures in our recent publications. Freshly distilled acetonitrile was used for each day's measurements. The pseudo-first order rate constants (k^{pfo}'s) were determined using the SF-61DX2 Hi-Tech KinetAsyst double-mixing stopped-flow instrument. The *Abs* - time data of the corresponding 12-15 half-lives were collected to derive the initial k^{pfo} by fitting the 12 half-live data (~99.98% completion of the reaction). Based on this k^{pfo} value, we calculated the half-life time, and ran 3-6 formal kinetic measurements over a fixed number (typically 12.5 or 13.5) of half-lives, with one minute time interval between runs to allow temperature equilibration of the reaction solutions before injecting into the thermostated reaction cell. Using the software integrated with the instrument, we fitted 12 half-lives of the *Abs* - time data. For the Tables S3 and S5 results only, due to slow reactions, data for 3.5 half-lives were collected and fitting of 3.0 half-lives data was performed to determine the k^{pfo}. If a small spike of the absorbance change is observed at the very beginning due to solution mixing, the data selected for the fit would exclude that part of the data. Figure S1 shows one example as to how the fit behaves and how the k^{pfo} is derived.

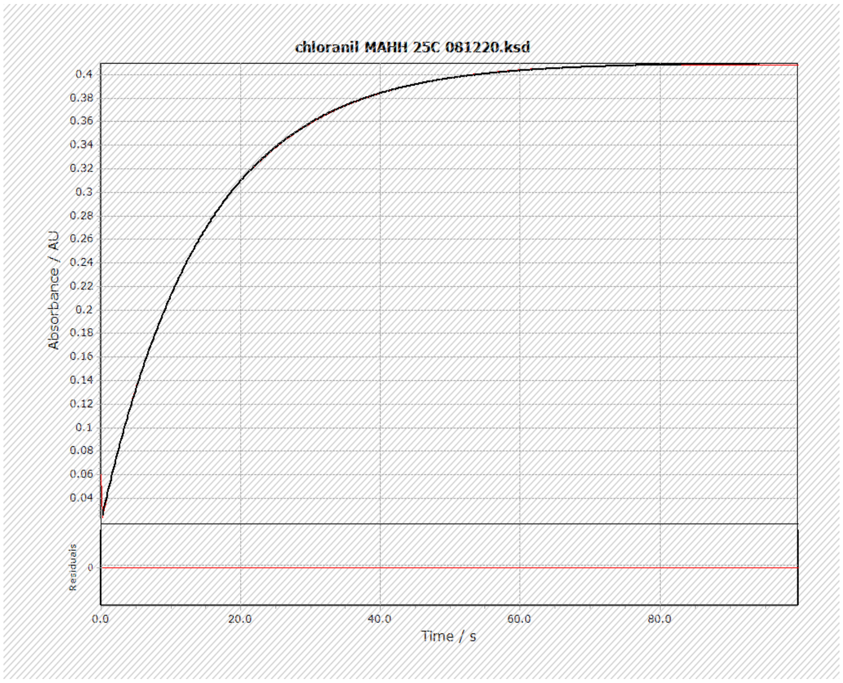

Figure S1. The fit to the Abs - time data for the reaction between MAH (3.52 x 10^{-3} M) and chloranil (4.40 x 10^{-5} M) in acetonitrile at 25°C at 436 nm (due to MA^+ formation) following the first-order kinetic law (Definition: Y = -A*exp(-R*X) + C; R is k^{pfo}). This is copied from the Kinetic Studio report generated from the fitting software from the instrument. The red kinetic trace (with 13.5 $t_{1/2}$) is from the experimental data, and the black curve (with 12.0 $t_{1/2}$) is the fit.

Measurements of k^{pfo}'s for 1° KIE derivation were performed over a temperature range of 40 °C at the same day and repeated on two additional days. E_{aH} and E_{aD} were derived to calculate the ΔE_a. A typical kinetic procedure is as follows. Three to six consecutive kinetic runs (or six, each covering 13.5 half-lives) were performed back-to-back for each isotopic reaction. The procedure was then repeated at other temperatures as quickly as possible (*e.g.*, 15, 25, 35, 45, 55 °C, in order) to maintain the constant instrument settings and minimize any aging of the reaction solutions. (Although the reaction solutions are relatively stable, they were wrapped with aluminum foil and kept in a refrigerator between runs to avoid introducing any unknown impurities.) Repetitions of kinetic measurements sometimes used different batches of substrates and solvents, and sometimes were done by

different workers. That was to eliminate the effect of possible different impurity from unknown sources or the human errors on the KIE measurements.

Therefore, one KIE value was obtained from *at least* 9 repetitions (3 days of measurements with 3 (in some cases, 6) repetitions each day). Pooled standard deviations were reported, and individual standard deviations from the three separate days of measurements are also provided in Tables S1 to S7 for comparison. Kinetic results (from the extent of reaction of close to 1% to 99.98% (corresponding to 12 half-lives)) were fitted very well/excellently to the first-order rate law for k^{pfo} derivation (see the subsequent Data Availability Statement) and to the Arrhenius equation for E_a derivation (see the subsequent data Tabels S1 to S6 and exemplified Arrhenius plot of second-order rate constants (k_2's) in Figure S2). The R^2 values ranging from 0.9990 to 1.0000, most commonly very close to or exactly 1.0000. The k_2 was derived from k^{pfo}/[excess substrate].

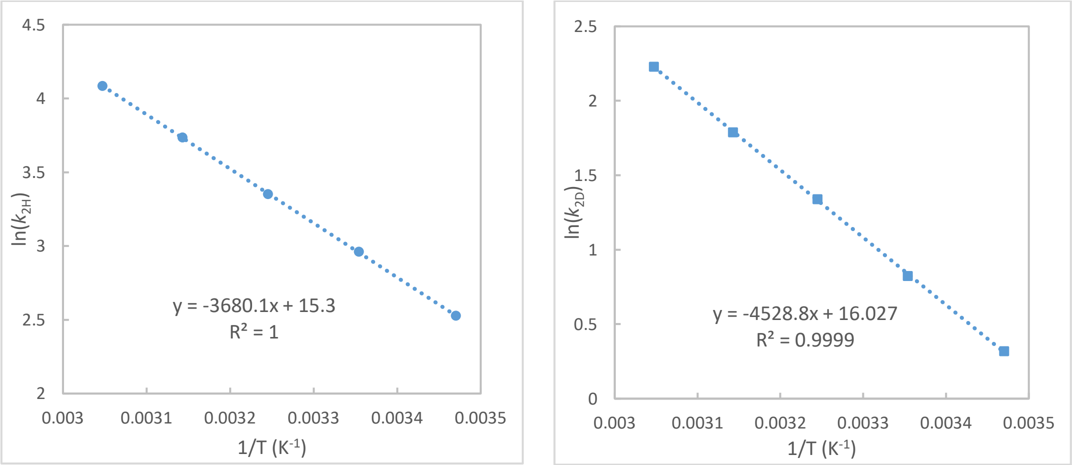

Figure S2. Exemplified Arrhenius plots for hydride (left) and deuteride (right) transfer reactions from MAH and MAH-d_2 in acetonitrile (temperatures are 15, 25, 35, 45, and 55 °C, respectively). Data and concentration conditions are from Table S3 Day 1 experiment (from the subsequent Data Availability Statement). The same plots for other reactions can be drawn using the data in the Data Availability Statement.

The kinetic measurement for the reaction of MAH has been reported in the literature.[3] All measurements with RAHs in this work used the same procedures. The kinetic scans of the reaction of HEH with chloranil is included in Figure S3. The kinetics of the reactions were determined by following the absorbance decay at 395 nm due to HEH with time. Detailed kinetic measurement conditions and the raw data can be found from Tables S1 to S7.

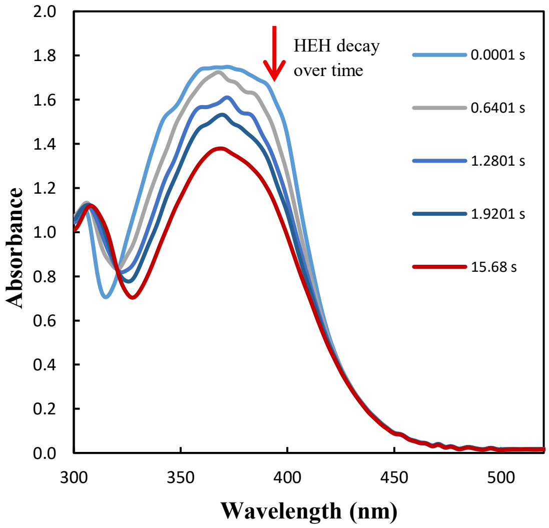

Figure S3. Kinetic scans of the reaction of HEH with chloranil at 25 °C in acetonitrile ([HEH] = 0.220 mM, [Chloranil] = 6.60 mM)

Plots of Temperature Dependence of KIEs (Figure S4)

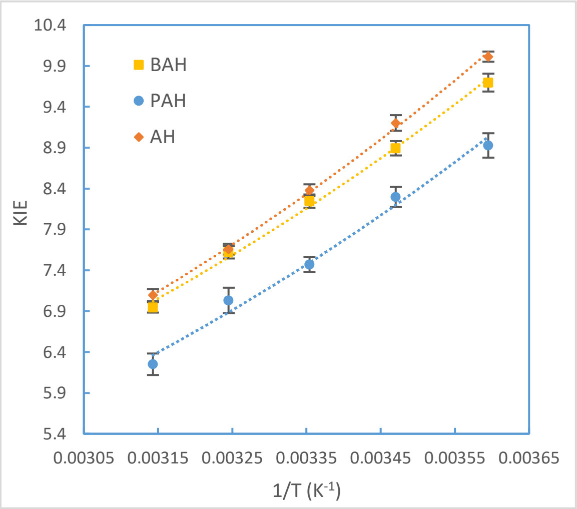

Figure S4. The Arrhenius plot of KIEs for hydride transfer reactions from AH, PAH and BAH to Cl_4Q in acetonitrile (temperatures are 15, 25, 35, 45, and 55 °C, respectively). Lines represent nonlinear regression using an Arrhenius-type exponential equation (KIE *vs.* EXP(1/T)). Those with MAH and HEH can be seen from the main paper.

Kinetic Data

Table S1. The temperature effects on the rate constants and 1° KIEs of the hydride transfer reaction from HAH to chloranil (excess) in acetonitrile [a,b]

**Temp (°C)**	k_{2H} **($M^{-1}s^{-1}$)**	k_{2D} **($M^{-1}s^{-1}$)**	**1° KIE**
55.0	1.31(0.01)(0.01) x10^2	1.84(0.01)(0.01) x10	7.10(0.07)(0.06)
45.0	9.77(0.06)(0.02) x10	1.28(0.01)(0.01) x10	7.66(0.07)(0.05)
35.0	7.21(0.05)(0.09)) x10	8.61(0.04)(0.04)	8.38(0.07)(0.14)
25.0	5.17(0.04)(0.09) x10	5.62(0.03)(0.10)	9.20(0.10)(0.09)
15.0	3.68(0.01)(0.03) x10	3.68(0.02)(0.02)	10.00(0.06)(0.13)

ΔE_a = 1.64 (0.08)(0.04)

[a] Repeated on three different days with 3 repetitions each day. Numbers in the first parenthesis for each value are the pooled standard deviations S(pooled), numbers in the second parathesis are the standard deviations of the three values from three days of measurements; [b] [HAH] = 0.044 mM, [chloranil] = 3.52 mM. Absorbance growth at 355 nm due to HA^+ was followed for kinetic measurements.

Table S2. The temperature effects on the rate constants and 1° KIEs of the hydride transfer reaction from MAH to chloranil (excess) in acetonitrile [a,b]

**Temp (°C)**	k_{2H} **($M^{-1}s^{-1}$)**	k_{2D} **($M^{-1}s^{-1}$)**	**1° KIE**
55.0	6.18(0.15)(0.29) x 10	8.92(0.09)(0.22)	6.93 (0.18)(0.32)
45.0	4.25(0.05)(0.10) x 10	5.62(0.03)(0.09)	7.56 (0.09)(0.08)
35.0	2.84(0.02)(0.04) x 10	3.48(0.02)(0.09)	8.16 (0.07)(0.18)
25.0	1.89(0.01)(0.06) x 10	2.12(0.02)(0.03)	8.90 (0.10)(0.18)
15.0	1.22(0.004)(0.02) x 10	1.23(0.02)(0.02)	9.96 (0.13)(0.14)

ΔE_a = 1.67 (0.13)(0.14)

[a] Repeated on three different days with 3 repetitions each day. Numbers in the first parenthesis for each value are the pooled standard deviations S(pooled), numbers in the second parathesis are the standard deviations of the three values from three days of measurements; [b] [MAH] = 0.044 mM, [Chloranil] = 3.52 mM. Absorbance growth at 365 nm due to MA^+ was followed for kinetic measurements.

Table S3. The temperature effects on the rate constants and 1° KIEs of the hydride transfer reaction from MAH (excess) to chloranil in acetonitrile [a,b]

**Temp (°C)**	$\boldsymbol{k_{2H}}$ **(M^{-1}s^{-1})**	$\boldsymbol{k_{2D}}$ **(M^{-1}s^{-1})**	**1° KIE**
55.0	5.91(0.02)(0.08) x 10	9.36(0.10)(0.15)	6.31 (0.07)(0.19)
45.0	4.20(0.04)(0.03) x 10	5.87(0.06)(0.09)	7.15 (0.09)(0.13)
35.0	2.84(0.02)(0.02) x 10	3.80(0.04)(0.03)	7.47 (0.09)(0.03)
25.0	1.92(0.01)(0.01) x 10	2.26(0.04)(0.02)	8.53 (0.16)(0.05)
15.0	1.25(0.00$_3$)(0.01) x 10	1.37(0.01)(0.01)	9.10 (0.05)(0.07)
	ΔE_a = 1.70 (0.13)(0.09)		

[a] Repeated on three different days with 3 repetitions each day. Numbers in the first parenthesis for each value are the pooled standard deviations S(pooled), numbers in the second parathesis are the standard deviations of the three values from three days of measurements; [b] [MAH] = 3.52 mM, [Chloranil] = 0.044 mM. Absorbance growth at 358 nm due to MA^+ was followed for kinetic measurements.

Table S4. The temperature effects on the rate constants and 1° KIEs of the hydride transfer reaction from PAH (excess) to Chloranil in acetonitrile [a,b]

**Temp (°C)**	$\boldsymbol{k_{2H}}$ **(M^{-1}s^{-1})**	$\boldsymbol{k_{2D}}$ **(M^{-1}s^{-1})**	**1° KIE**
55.0	1.53(0.02)(0.01) x 10^2	2.45(0.05)(0.07) x 10	6.25 (0.13)(0.21)
45.0	1.21(0.01)(0.02) x 10^2	1.72(0.03)(0.03) x 10	7.03 (0.16)(0.14)
35.0	9.15(0.09)(0.13) x 10	1.23(0.01)(0.03) x 10	7.47 (0.09)(0.24)
25.0	6.77(0.05)(0.01) x 10	8.17(0.11)(0.14)	8.30 (0.12)(0.07)
15.0	5.01(0.07)(0.22) x 10	5.61(0.05)(0.09)	8.93 (0.15)(0.29)
	ΔE_a = 1.65 (0.15)(0.06)		

[a] Repeated on three different days with 3 repetitions each day. Numbers in the first parenthesis for each value are the pooled standard deviations S(pooled), numbers in the second parathesis are the standard deviations of the three values from three days of measurements;. [b] [PAH] = 3.52 mM, [Chloranil] = 0.044 mM. Absorbance growth at 358 nm due to PA^+ was followed for kinetic measurements.

Table S5. The temperature effects on the rate constants and 1° KIEs of the hydride transfer reaction from BAH to Chloranil (excess) in acetonitrile [a,b]

**Temp (°C)**	$\boldsymbol{k_{2H}}$ **(M^{-1}s^{-1})**	$\boldsymbol{k_{2D}}$ **(M^{-1}s^{-1})**	**1° KIE**
55.0	3.70(0.03)(0.09) x 10	5.33(0.03)(0.09)	6.95 (0.06)(0.07)
45.0	2.62(0.02)(0.05) x 10	3.44(0.03)(0.06)	7.62 (0.08)(0.08)
35.0	1.80(0.01)(0.02) x 10	2.19(0.02)(0.05)	8.25 (0.08)(0.24)
25.0	1.23(0.01)(0.02) x 10	1.39(0.01)(0.05)	8.89 (0.09)(0.35)
15.0	8.19(0.04)(0.10)	8.44(0.08)(0.07) x 10^{-1}	9.70 (0.11)(0.16)
	ΔE_a = 1.54 (0.12)(0.07)		

[a] Repeated on three different days with 3 repetitions each day. Numbers in the first parenthesis for each value are the pooled standard deviations S(pooled), numbers in the second parathesis are the standard deviations of the three values from three days of measurements;. [b] [BAH] = 0.044 mM, [Chloranil] = 3.52 mM. Absorbance growth at 358 nm due to BA^+ was followed for kinetic measurements.

Table S6. The temperature effects on the rate constants and 1° KIEs of the hydride transfer reaction from HEH to Chloranil in acetonitrile [a,b]

**Temp (°C)**	$\boldsymbol{k_{2H}}$ **(M^{-1}s^{-1})**	$\boldsymbol{k_{2D}}$ **(M^{-1}s^{-1})**	**1° KIE**
55.0	4.33(0.04)(0.08) x 10^2	7.15(0.04)(0.15) x 10	6.06 (0.06)(0.02)
45.0	3.23(0.03)(0.06) x 10^2	5.10(0.03)(0.09) x 10	6.33 (0.07)(0.07)
35.0	2.41(0.02)(0.03) x 10^2	3.61(0.02)(0.08) x 10	6.66 (0.06)(0.09)

25.0	1.76(0.01)(0.04) x 10^2	2.49(0.02)(0.04) x 10	7.09 (0.07)(0.03)
15.0	1.25(0.01)(0.03) x 10^2	1.66(0.01)(0.05) x 10	7.54 (0.07)(0.01)

ΔE_a = 1.04 (0.05)(0.02)

[a] Repeated on three different days with 3 repetitions on Day 1 and 6 repetitions on Days 2 and 3. Numbers in the first parenthesis for each value are the pooled standard deviations S(pooled), numbers in the second parathesis are the standard deviations of the three values from three days of measurements; [b] [HEH] = 0.220 mM, [Chloranil] = 6.60 mM. Absorbance decay of HEH at 395 nm was followed for kinetic measurements.

Table S7. Kinetic data for determination of the NH/ND-2° KIEs on HEH and standard deviations (SD's) for its reaction with Cl_4Q in acetonitrile

# of Measurements	*k*(NH) ($M^{-1}s^{-1}$) [a]	*k*(ND) ($M^{-1}s^{-1}$) [a]	NH/ND-2° KIE [b]
1	1.729 (0.013) x10^2	1.702 (0.013) x10^2	1.02 (0.01)
2	1.726 (0.008) x10^2	1.725 (0.004) x10^2	1.00 (0.01)
3	1.753 (0.004) x10^2	1.736 (0.010) x10^2	1.01 (0.01)
			**Average**
			1.01 (0.01) [c]

[a] Repeated three times with 6 repetitions each day. Numbers in the first parenthesis for each value are the pooled standard deviations S(pooled); [b] [HEH] = 0.220 mM, [Chloranil] = 6.60 mM. Absorbance decay of HEH at 395 nm was followed for kinetic measurements.

References

1. Singh, G.; Austin, A.; Bai, M.; Bradshaw, J.; Hammann, B. A.; Kabotso, D. E. K.; Lu, Y. Study of the Effects of Remote Heavy Group Vibrations on the Temperature Dependence of Hydride Kinetic Isotope Effects of the NADH/NAD+ Model Reactions. *ACS Omega* **2024**, *9*, 20593-20600.
2. Ava, A.; Sager, J.; Phan, L.; Lu, Y. Structural Effects on the Hydride-Tunneling Kinetic Isotope Effects of NADH/NAD+ Model Reactions: Relating to the Donor–Acceptor Distances. *J. Org. Chem.* **2025**, *90*, 3110-3115.

Data Availability Statement

Primary kinetic data for Tables S1-S7 are presented below. We directly copied the original data from the corresponding excel data file. Due to the decimal point place difference in between the two places, data may slightly differ at the last digit of their numbers. Meanwhile, we provide the *Abs* – time (t) data (plots) for the measurements of the pseudo first-order rate constants (k^{pfo}'s).

Primary kinetic data for the rate constants in Table S1

Day 1 data (June 27, 2025)

	Pseudo-first-order rate constants k^{pfo} (s^{-1})						
**Temp (°C)**	**Trial H1**	**Trial H2**	**Trial H3**	**Average k_H^{pfo} (s^{-1})**	**Stdev**	**k_{2H} [b] ($M^{-1}s^{-1}$)**	**Stdev [a]**
55	0.30330	0.30723	0.30364	0.3047	0.002177	1.30E+02	0.92790
45	0.23055	0.22828	0.22844	0.2291	0.001267	9.76E+01	0.53988
35	0.16721	0.16735	0.16674	0.1671	0.00032	7.12E+01	0.13616
25	0.12017	0.11830	0.11871	0.1191	0.000983	5.07E+01	0.41885
15	0.08574	0.08564	0.08559	0.0857	0.00008	3.65E+01	0.03255
**Temp (°C)**	**Trial D1**	**Trial D2**	**Trial D3**	**Average k_D^{pfo} (s^{-1})**	**Stdev**	**k_{2D} [b] ($M^{-1}s^{-1}$)**	**Stdev [a]**
55	0.04332	0.0434	0.04328	0.0433	6.1101E-05	1.85E+01	0.026037
45	0.03018	0.02998	0.03029	0.0302	0.00015716	1.28E+01	0.06697
35	0.02022	0.0203	0.02047	0.0203	0.00012767	8.66E+00	0.05441
25	0.01291	0.01303	0.01286	0.0129	8.7369E-05	5.51E+00	0.03723

15	0.00868	0.00866	0.00858	0.0086	5.2915E-05	3.68E+00	0.02255

[a] = (Stdev(for k^{pfo})/k^{pfo})*k_2; [b] = k^{pfo}/(2[Cl_4Q]/3)

<table>
<tr><td>Three kinetic runs together with the averaged data</td><td>First-order kinetic fit of the averaged data as an example</td></tr>
<tr><td>
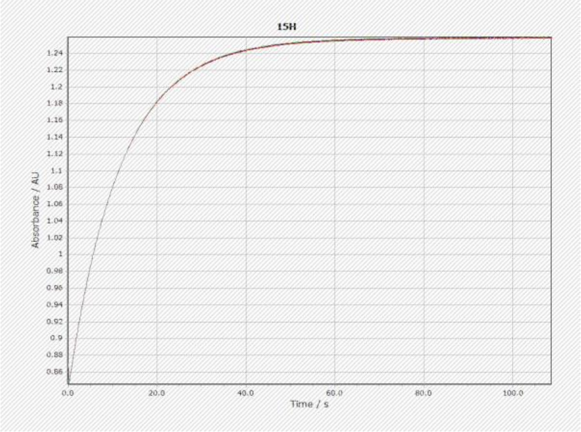

</td><td>
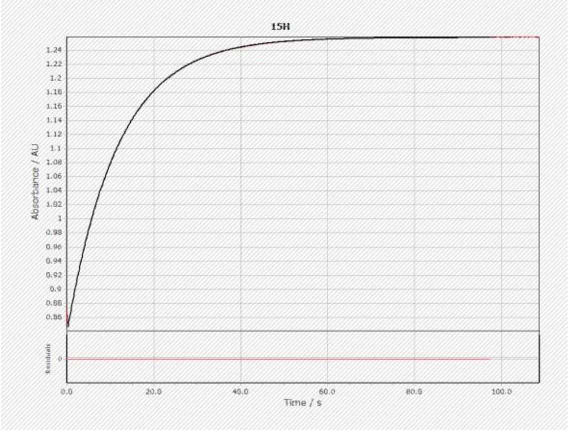

</td></tr>
<tr><td colspan="2">15℃ H</td></tr>
<tr><td>
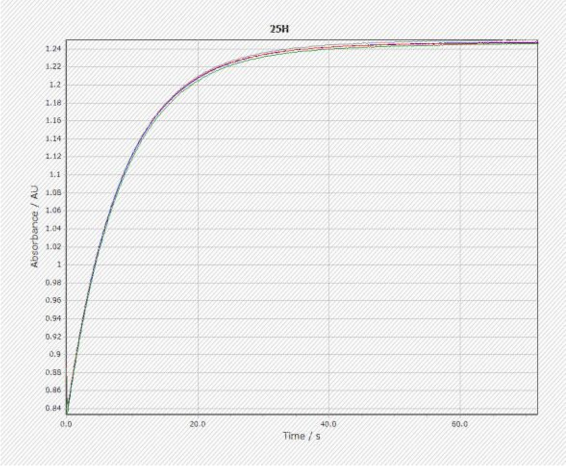

</td><td>
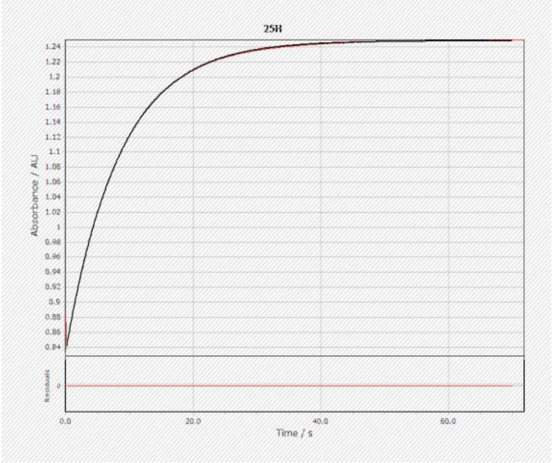

</td></tr>
<tr><td colspan="2">25℃ H</td></tr>
<tr><td>
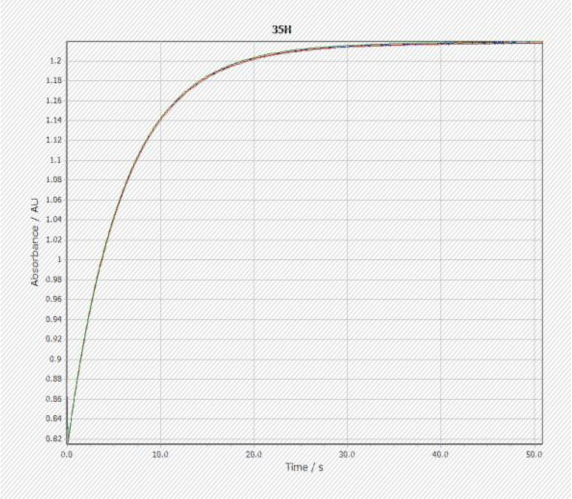

</td><td>
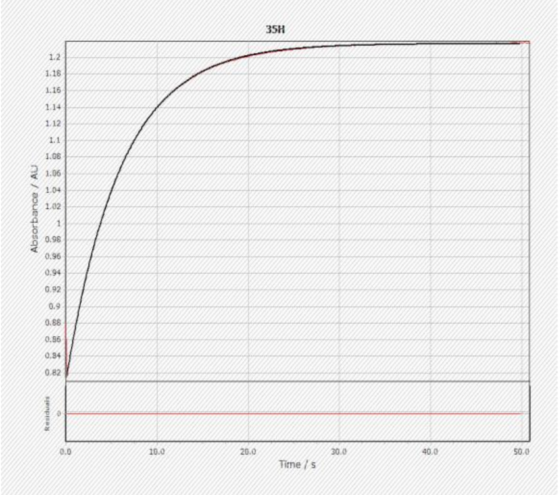

</td></tr>
<tr><td colspan="2">35℃ H</td></tr>
</table>

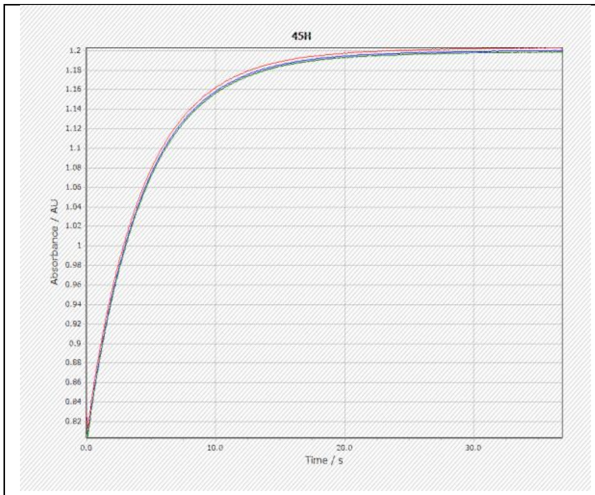

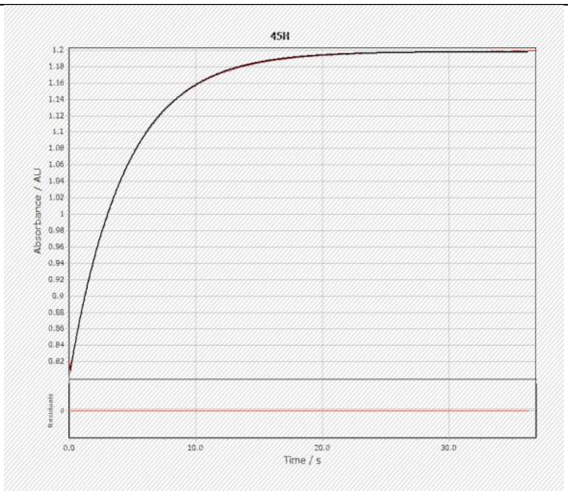

45°C H

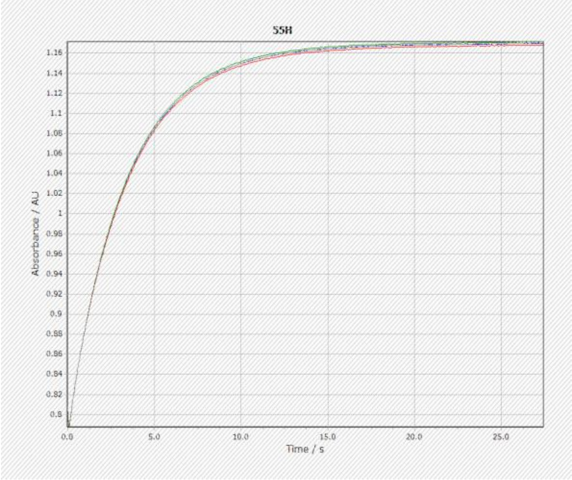

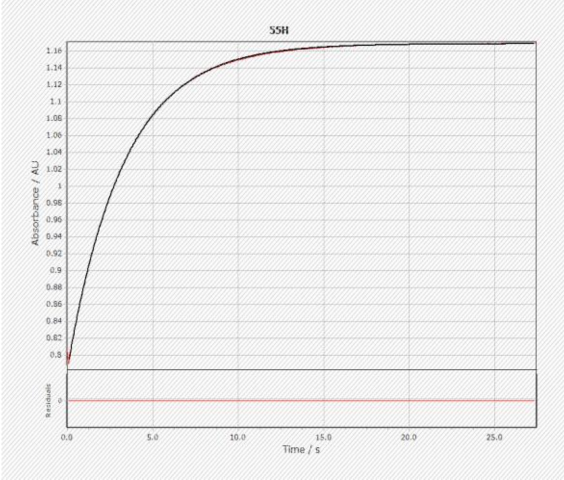

55°C H

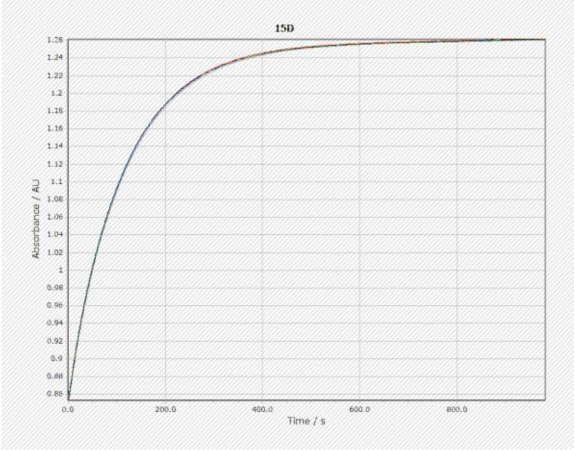

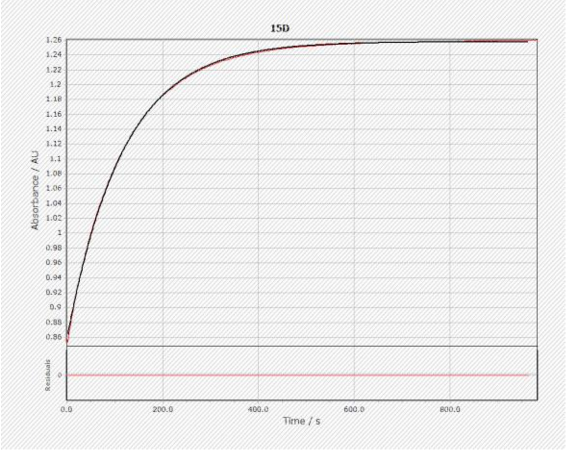

15°C D

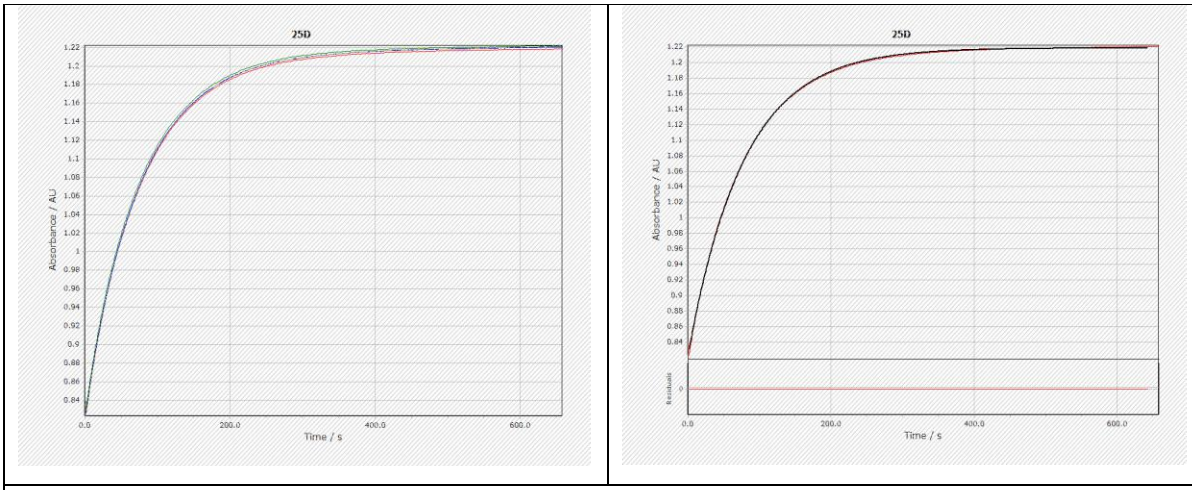

25°C D

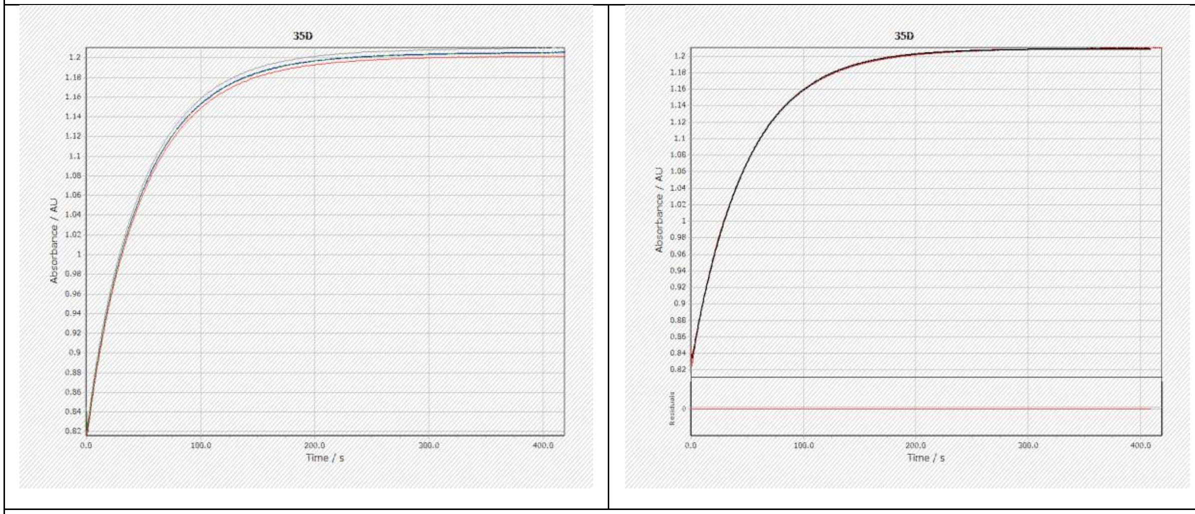

35°C D

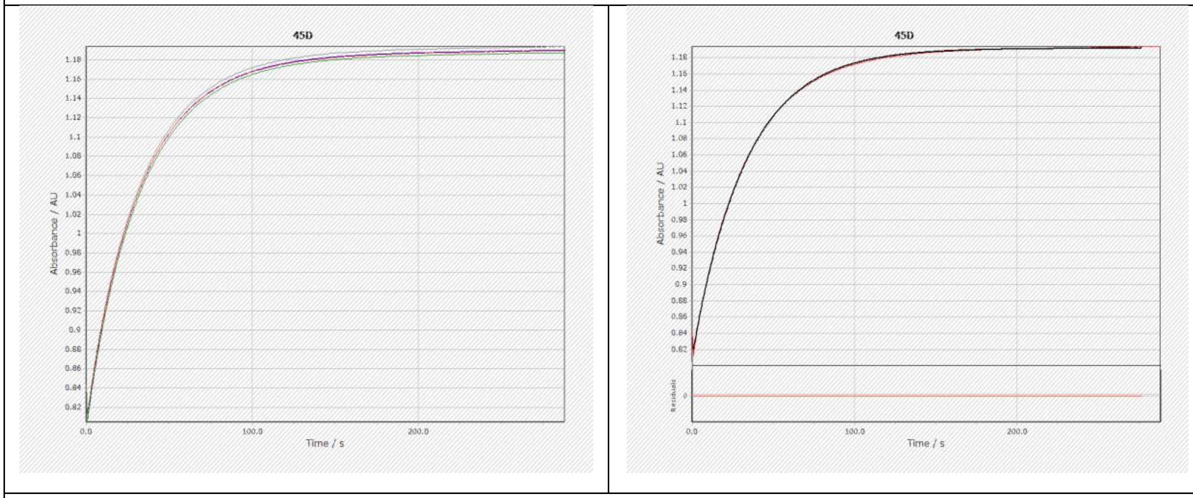

45°C D

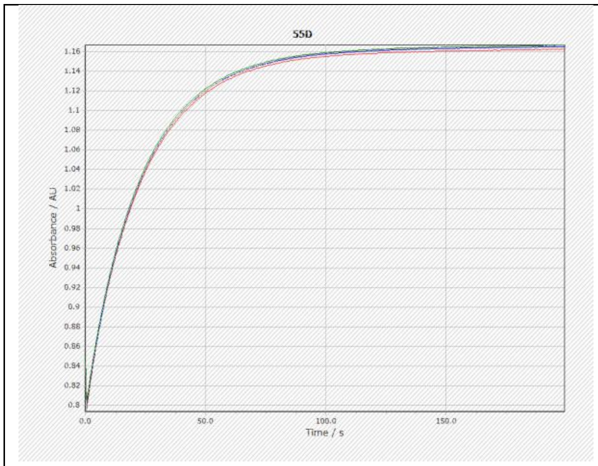

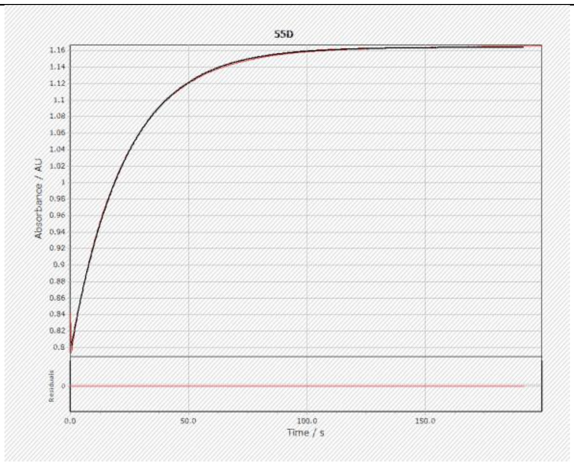

55℃ D

Day 2 data (July 01, 2025)

	Pseudo-first-order rate constants k^{pfo} (s^{-1})						
**Temp (°C)**	**Trial H1**	**Trial H2**	**Trial H3**	**Average k_H^{pfo} (s^{-1})**	**Stdev [b]**	**k_{2H} [b] (M^{-1}s^{-1})**	**Stdev [a]**
55	0.30937	0.30738	0.30832	0.3084	0.000996	1.31E+02	0.42422
45	0.22988	0.22844	0.23149	0.2299	0.001526	9.80E+01	0.65019
35	0.17061	0.17222	0.17041	0.1711	0.000992	7.29E+01	0.42286
25	0.12308	0.12322	0.12367	0.1233	0.000308	5.26E+01	0.13137
15	0.08632	0.08666	0.08623	0.0864	0.000227	3.68E+01	0.09664
**Temp (°C)**	**Trial D1**	**Trial D2**	**Trial D3**	**Average k_D^{pfo} (s^{-1})**	**Stdev**	**k_{2D} [b] (M^{-1}s^{-1})**	**Stdev [a]**
55	0.04355	0.04316	0.04246	0.0431	0.0005523	1.83E+01	0.235354
45	0.02959	0.03000	0.02995	0.0298	0.00022368	1.27E+01	0.09532
35	0.02005	0.02013	0.02024	0.0201	9.5394E-05	8.58E+00	0.04065
25	0.01336	0.01330	0.01318	0.0133	9.1652E-05	5.66E+00	0.03906
15	0.00863	0.00874	0.00863	0.0087	6.3509E-05	3.69E+00	0.02706

[a] = (Stdev(for k^{pfo})/k^{pfo})*k_2; [b] = k^{pfo}/(2[Cl_4Q]/3)

Three kinetic runs together with the averaged data	First-order kinetic fit of the averaged data as an example
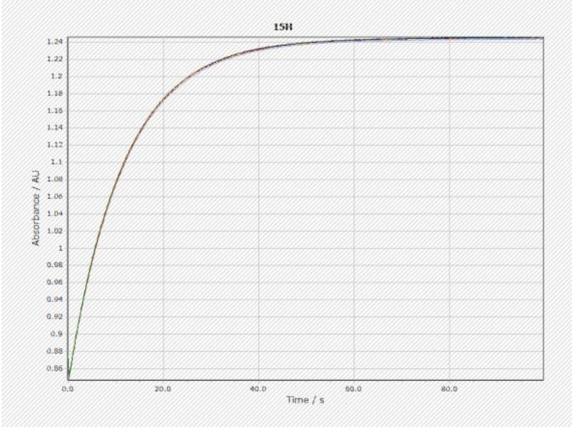	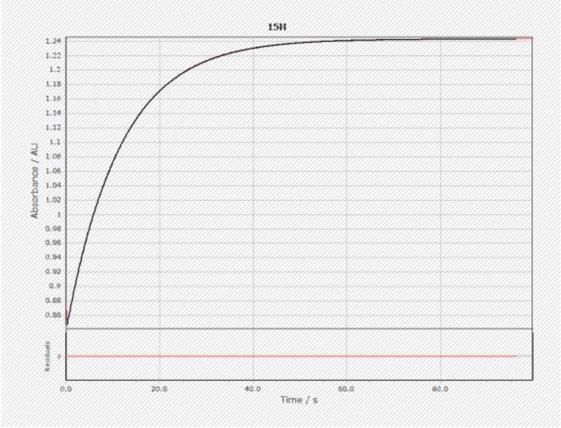
15°C H	
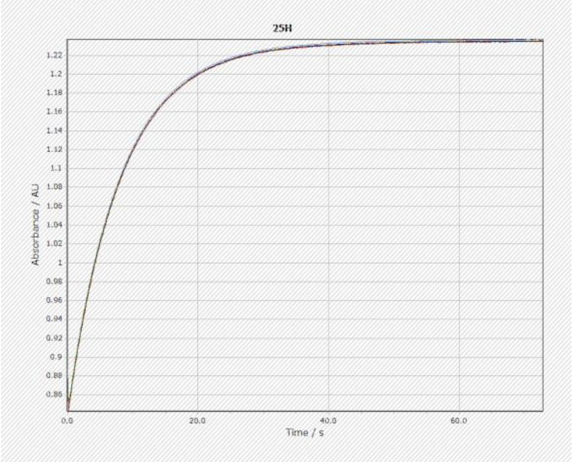	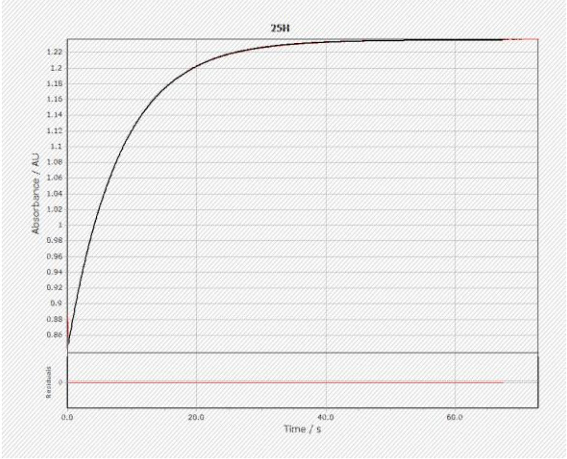
25°C H	
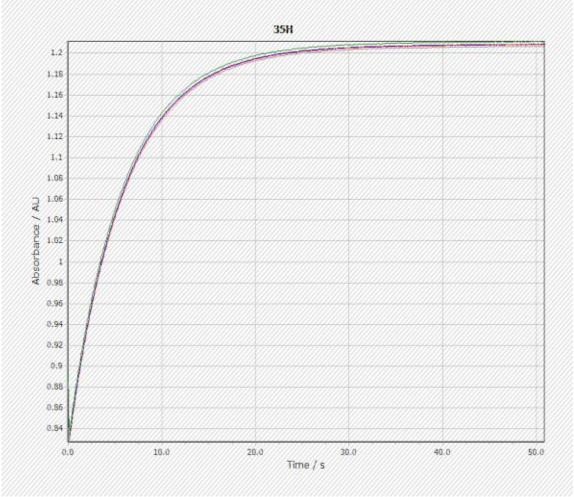	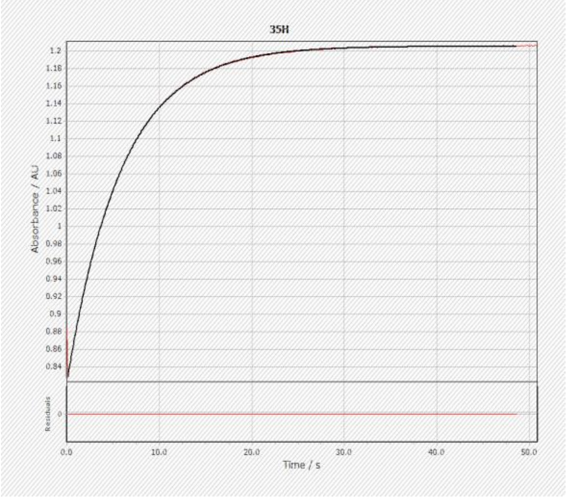
35°C H	

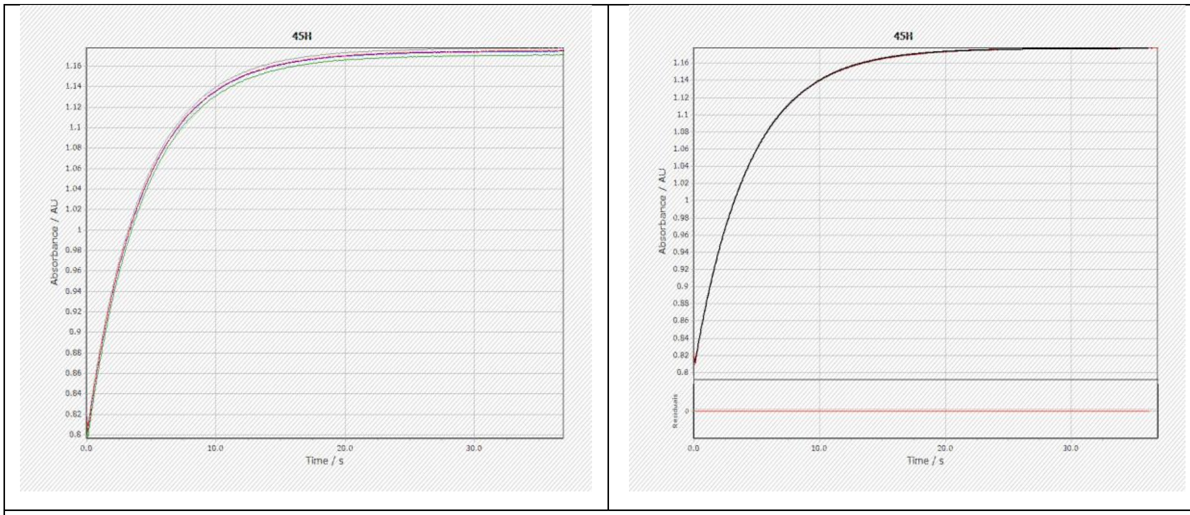

45°C H

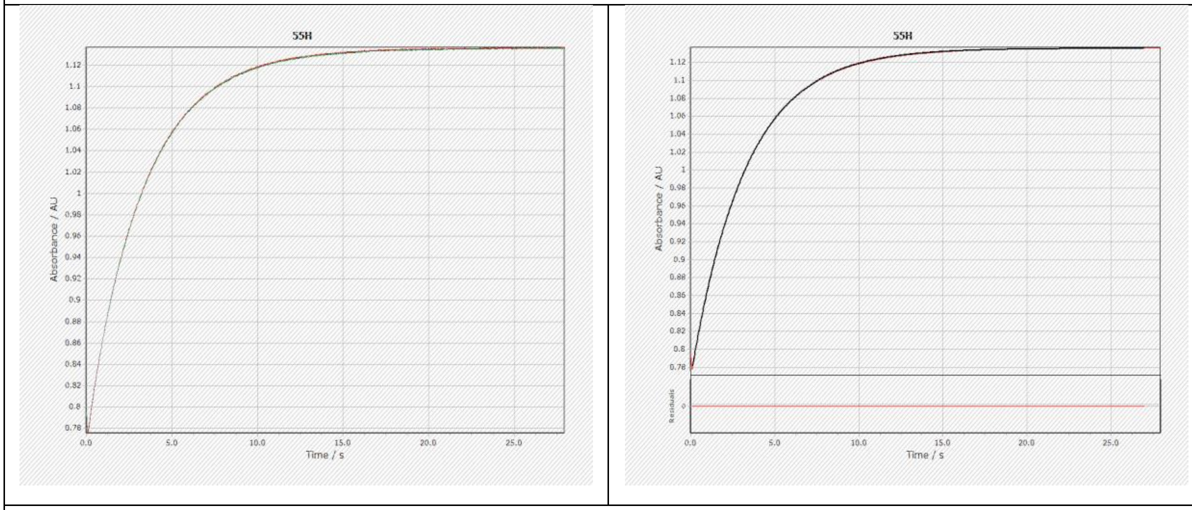

55°C H

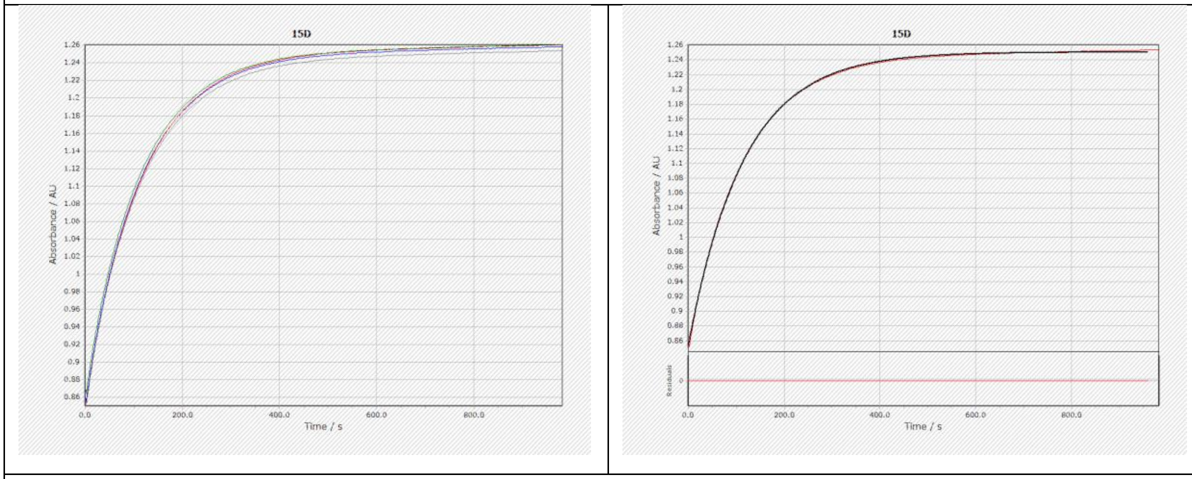

15°C D

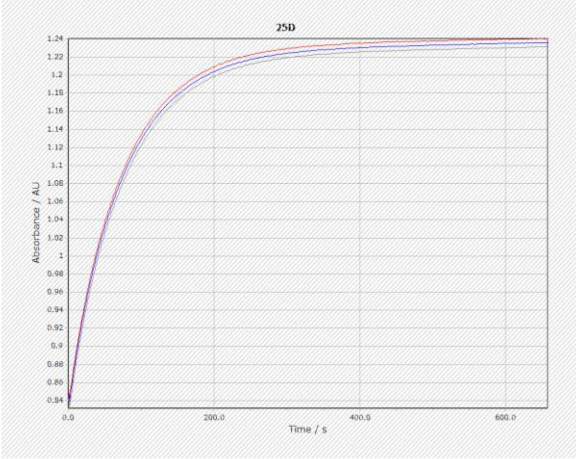

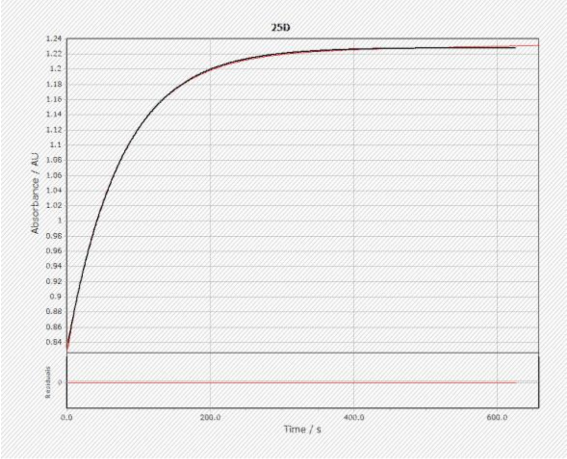

25°C D

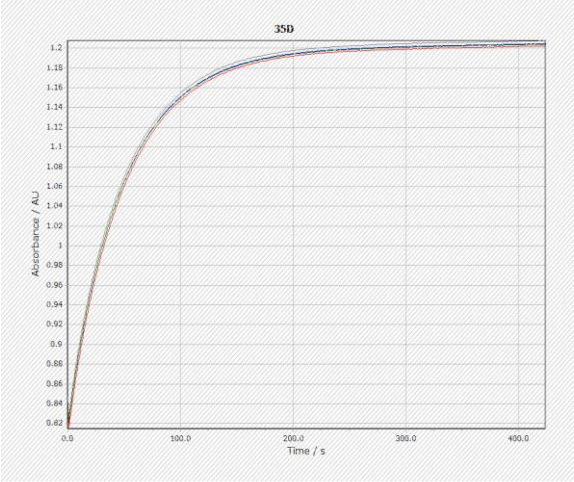

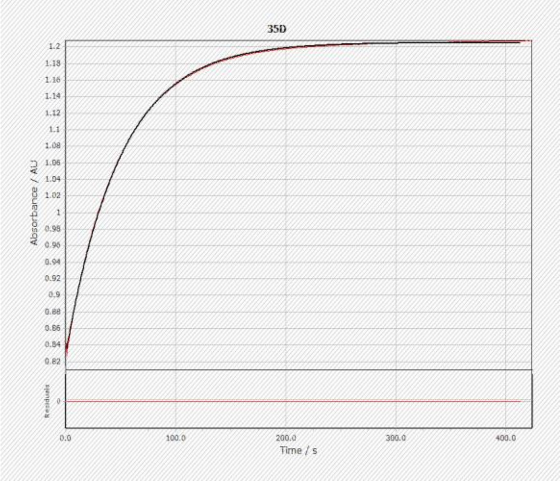

35°C D

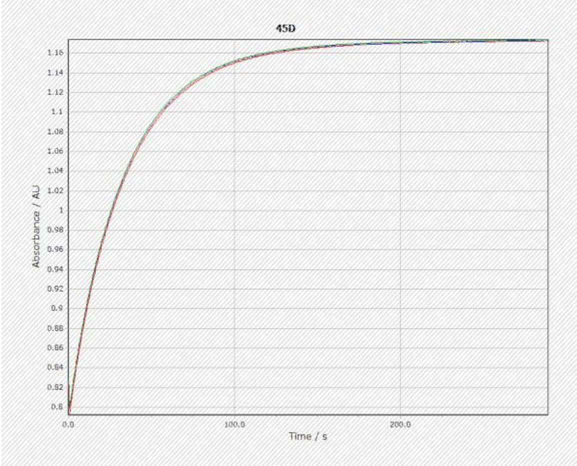

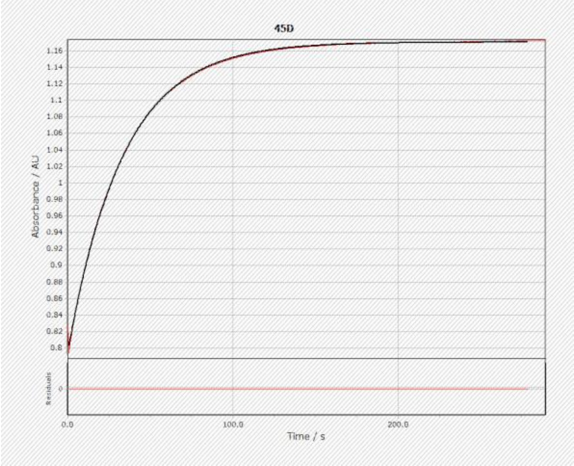

45°C D

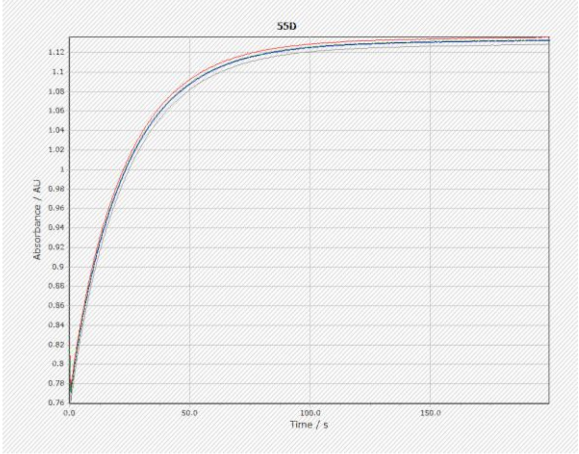

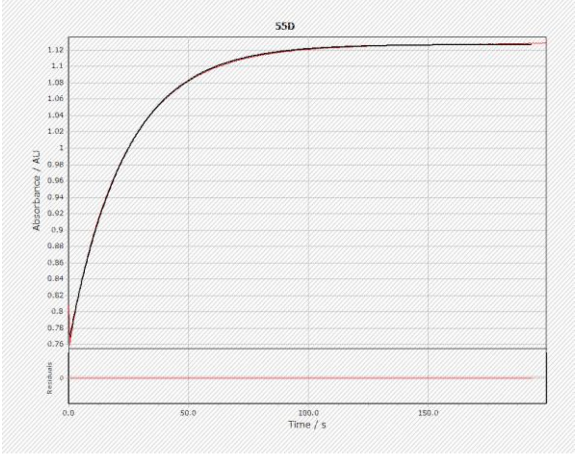

55°C D

Day 3 data (July 02, 2025)

Pseudo-first-order rate constants

k^{pfo} (s^{-1})

Temp (°C)	Trial H1	Trial H2	Trial H3	Average k_H^{pfo} (s^{-1})	Stdev	k_{2H} [b] ($M^{-1}s^{-1}$)	Stdev [a]
55	0.30652	0.30857	0.3029	0.3060	0.002871	1.30E+02	1.22344
45	0.22953	0.22739	0.23037	0.2291	0.001537	9.76E+01	0.65477
35	0.16817	0.16941	0.17149	0.1697	0.001678	7.23E+01	0.71489
25	0.12165	0.12334	0.1205	0.1218	0.001429	5.19E+01	0.60875
15	0.0872	0.08676	0.08719	0.0871	0.000251	3.71E+01	0.10704

Temp (°C)	Trial D1	Trial D2	Trial D3	Average k_D^{pfo} (s^{-1})	Stdev	k_{2D} [b] ($M^{-1}s^{-1}$)	Stdev [a]
55	0.0432	0.0431	0.04305	0.0431	7.6376E-05	1.84E+01	0.032547
45	0.0297	0.02998	0.02988	0.0299	0.00014189	1.27E+01	0.06047
35	0.02004	0.0202	0.02016	0.0201	8.3267E-05	8.58E+00	0.03548
25	0.01338	0.01342	0.0133	0.0134	6.1101E-05	5.70E+00	0.02604
15	0.00856	0.00860	0.00855	0.0086	2.6458E-05	3.65E+00	0.01127

[a] = (Stdev(for k^{pfo})/k^{pfo})*k_2; [b] = k^{pfo}/(2[Cl_4Q]/3)

Three kinetic runs together with the averaged data	First-order kinetic fit of the averaged data as an example

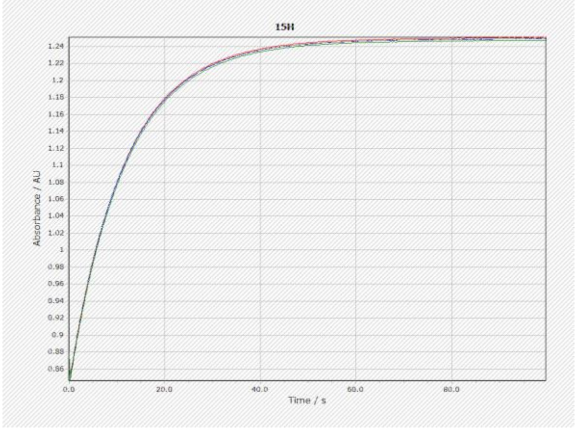

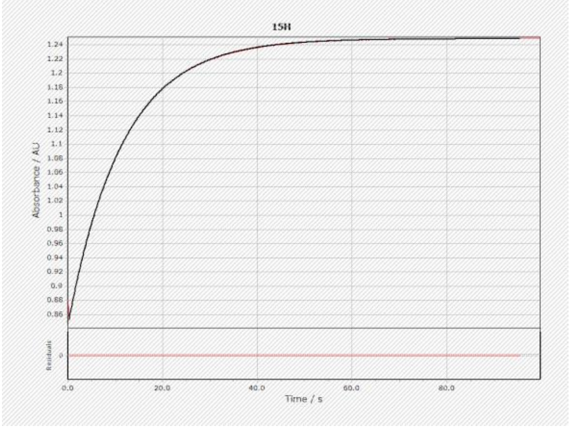

15°C H

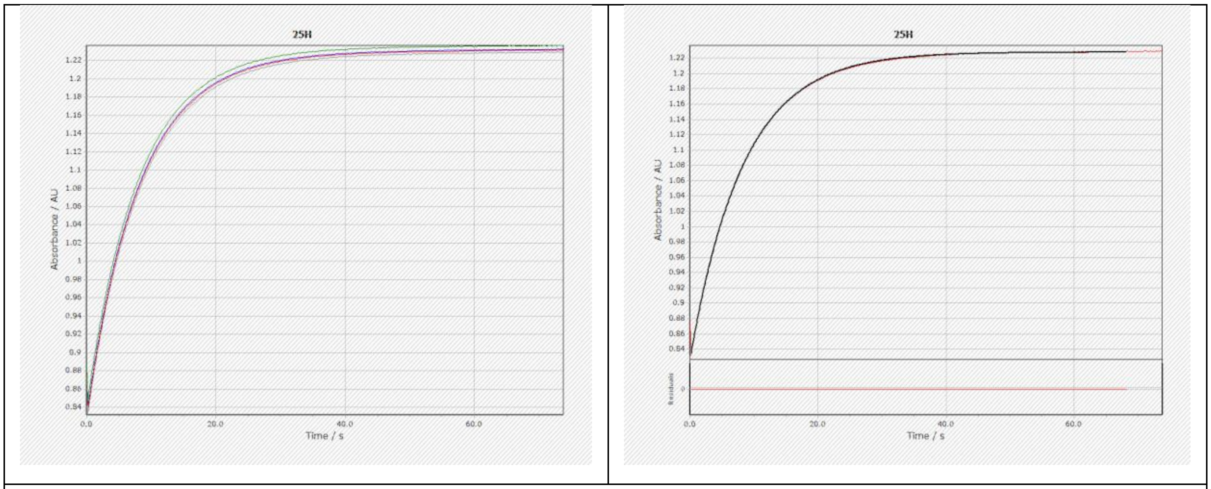

25°C H

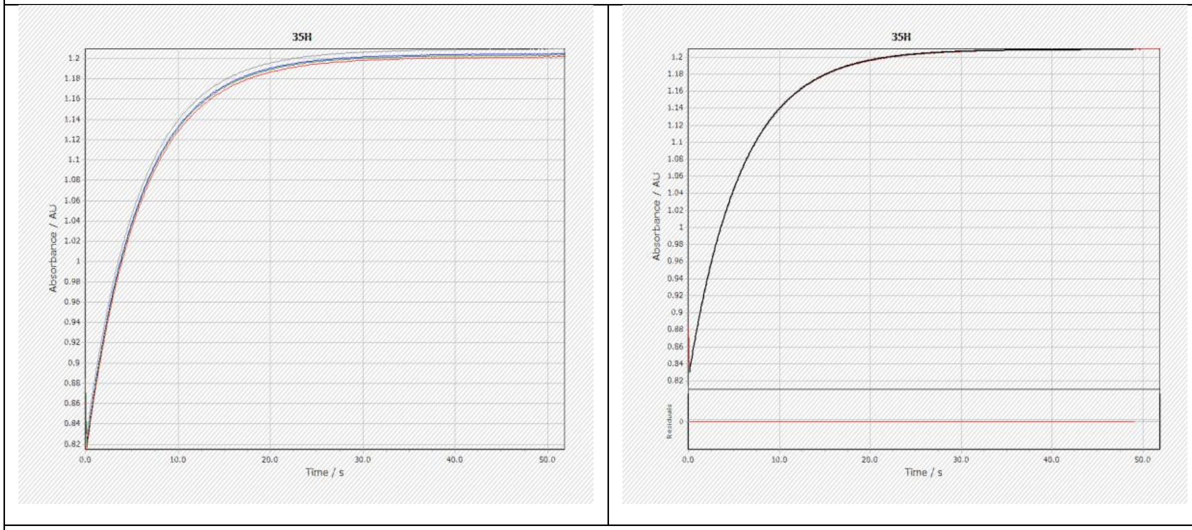

35°C H

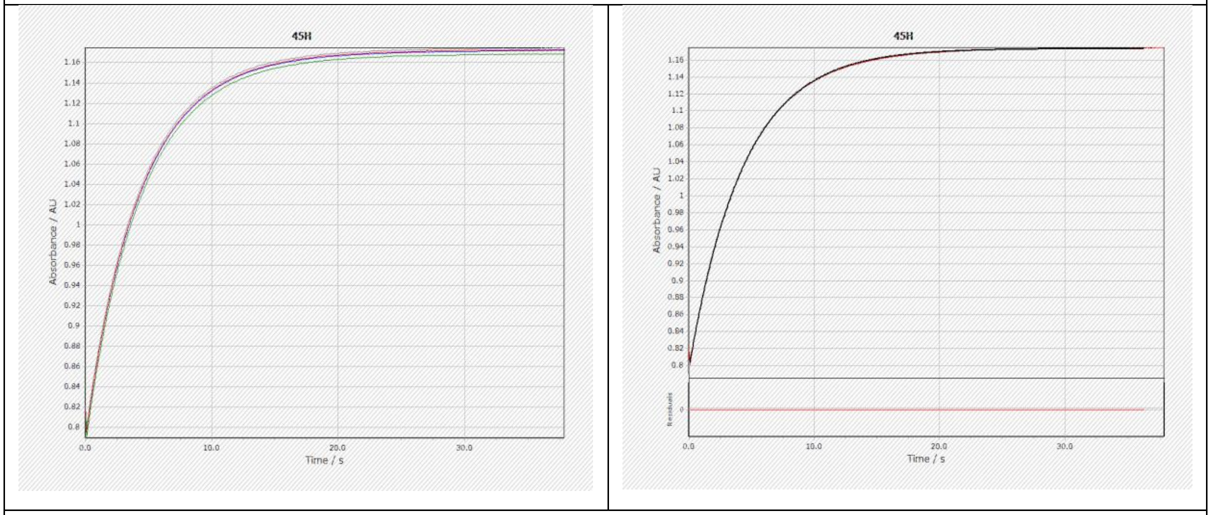

45°C H

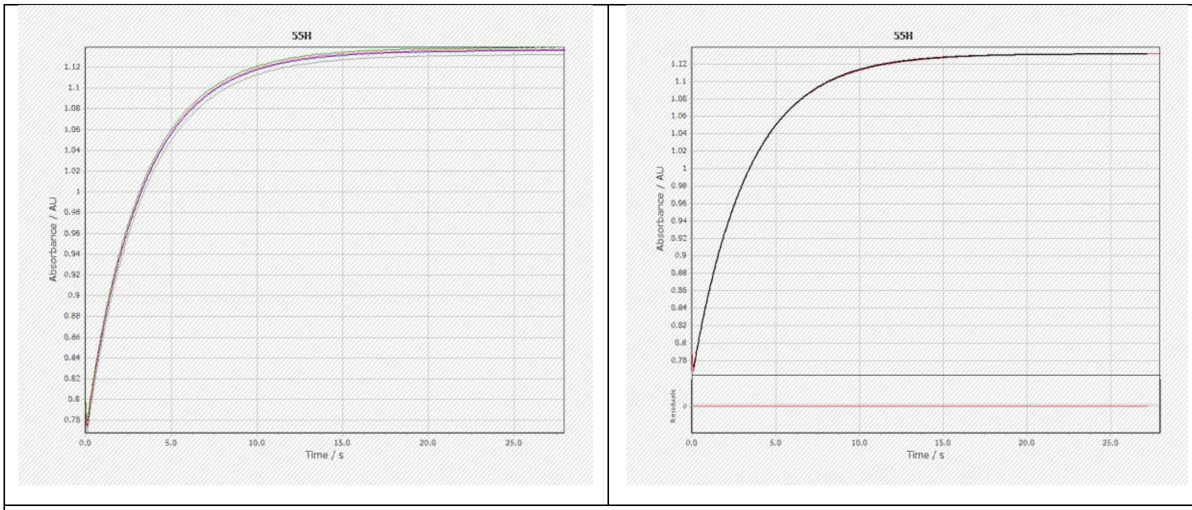

55°C H

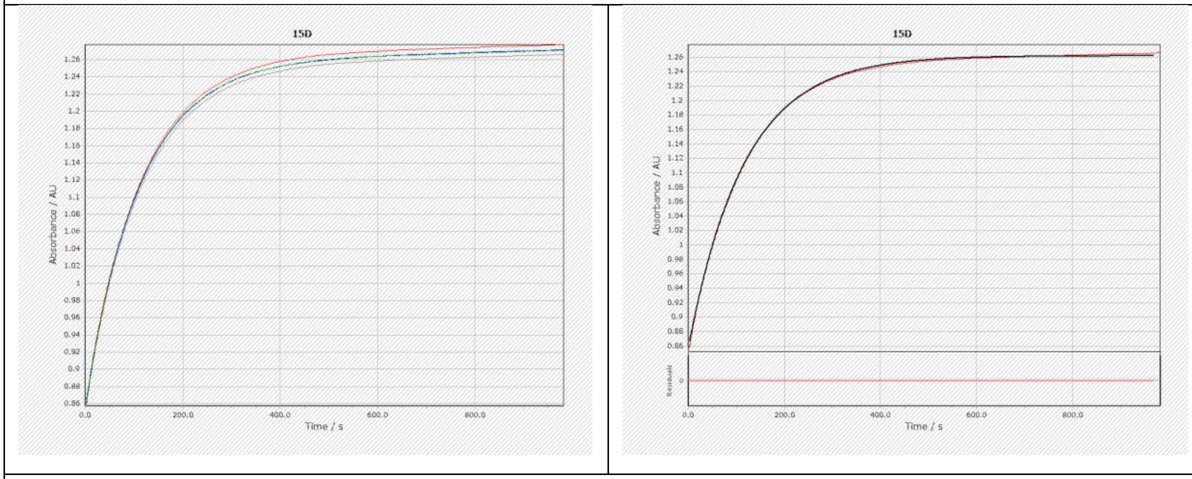

15°C D

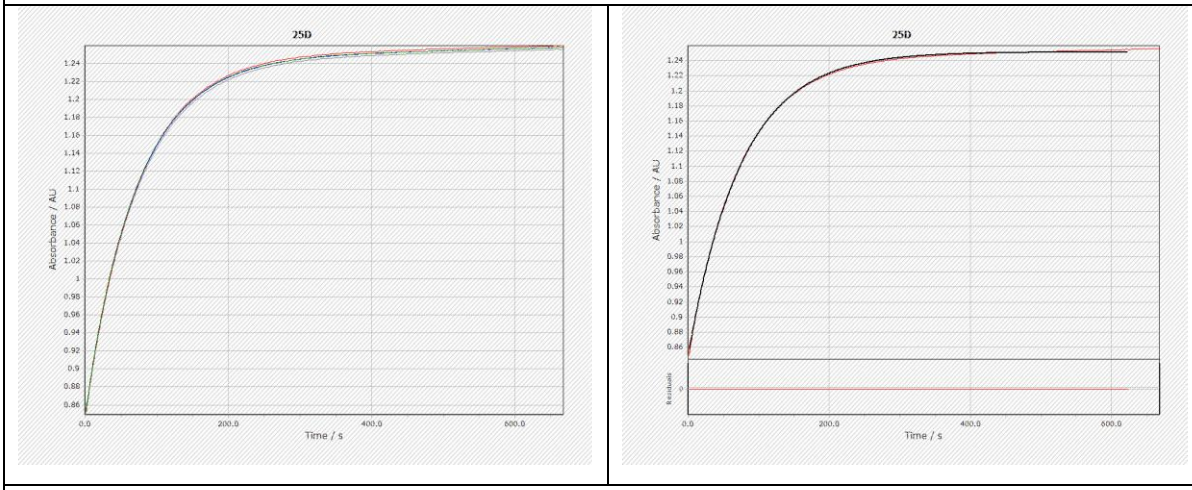

25°C D

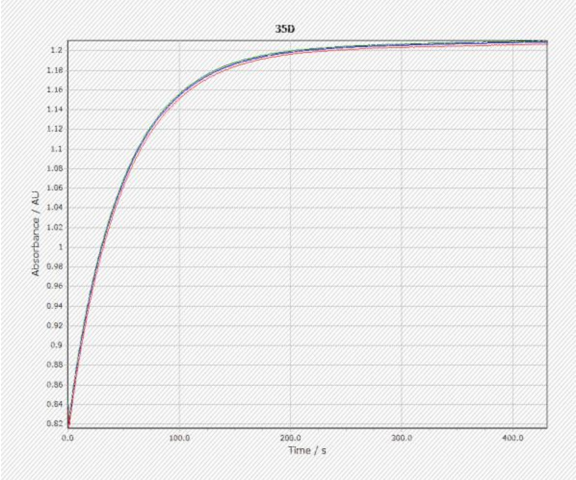

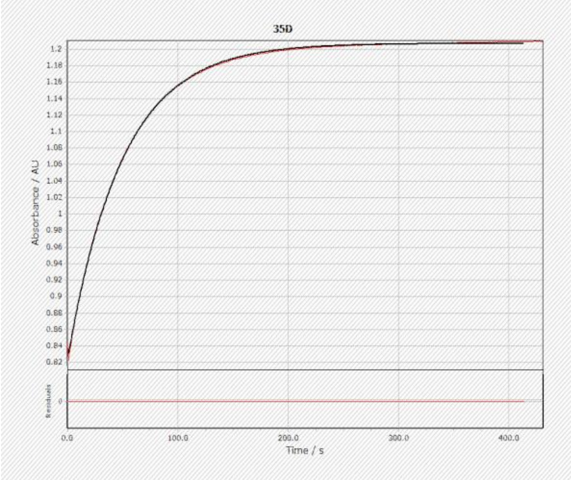

35°C D

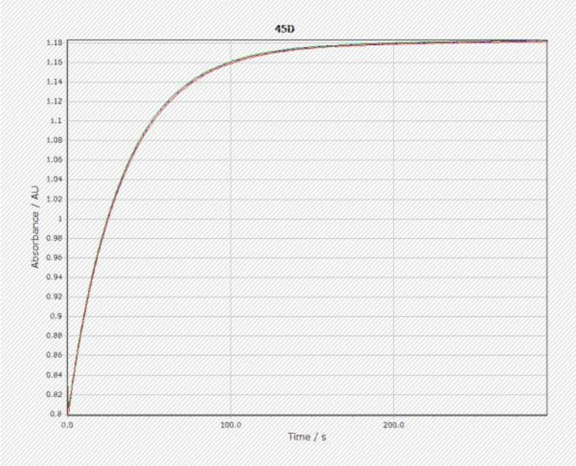

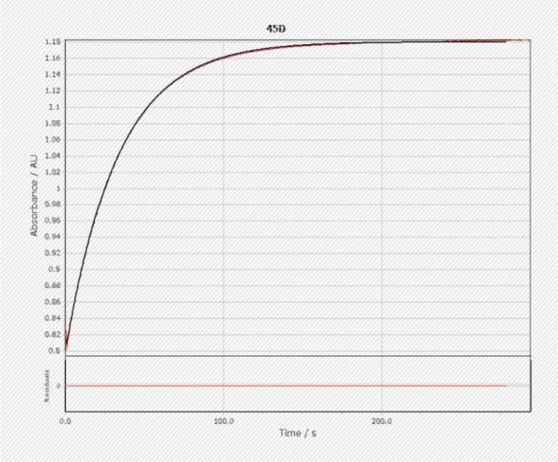

45°C D

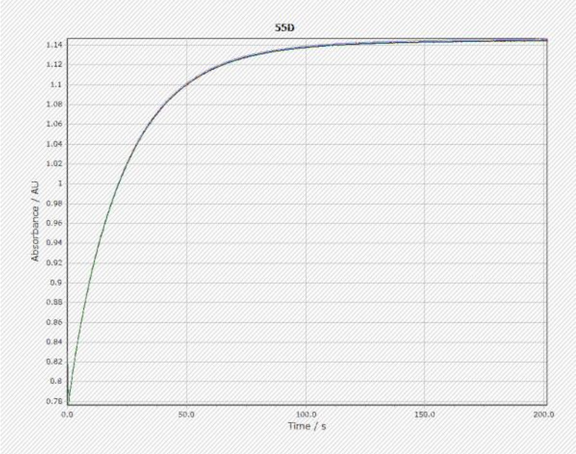

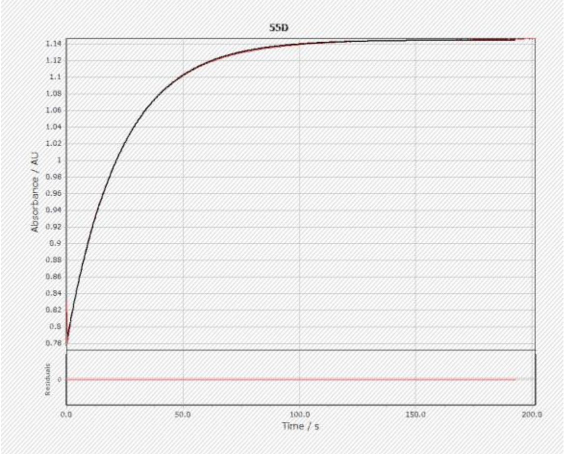

55°C D

Primary kinetic data for the rate constants in Table S2

Day 1 data (December 16, 2024)

	Pseudo-first-order rate constants k^{pfo} (s^{-1})						
**Temp (°C)**	**Trial H1**	**Trial H2**	**Trial H3**	**Average k_H^{pfo} (s^{-1})**	**Stdev**	**k_{2H} [b] ($M^{-1}s^{-1}$)**	**Stdev [a]**
55	0.14196	0.13909	0.14482	0.1420	0.002865	6.05E+01	1.22088
45	0.1002	0.10105	0.10051	0.1006	0.00043	4.29E+01	0.18330
35	0.06618	0.0651	0.06586	0.0657	0.000555	2.80E+01	0.23639
25	0.0438	0.04366	0.04374	0.0437	7.02E-05	1.86E+01	0.02993
15	0.02853	0.02838	0.02846	0.0285	7.51E-05	1.21E+01	0.03198
**Temp (°C)**	**Trial D1**	**Trial D2**	**Trial D3**	**Average k_D^{pfo} (s^{-1})**	**Stdev**	**k_{2D} [b] ($M^{-1}s^{-1}$)**	**Stdev [a]**
55	0.02156	0.02129	0.02133	0.0214	0.000146	9.12E+00	0.062095
45	0.01329	0.01334	0.01337	0.0133	4.04E-05	5.68E+00	0.01722
35	0.00816	0.0081	0.00816	0.0081	3.46E-05	3.47E+00	0.01476
25	0.00485	0.00492	0.00491	0.0049	3.79E-05	2.09E+00	0.01613
15	0.00296	0.00289	0.00285	0.0029	5.57E-05	1.24E+00	0.02373

[a] = (Stdev(for k^{pfo})/k^{pfo})*k_2; [b] = k^{pfo}/(2[Cl_4Q]/3)

Three kinetic runs together with the averaged data	First-order kinetic fit of the averaged data as an example
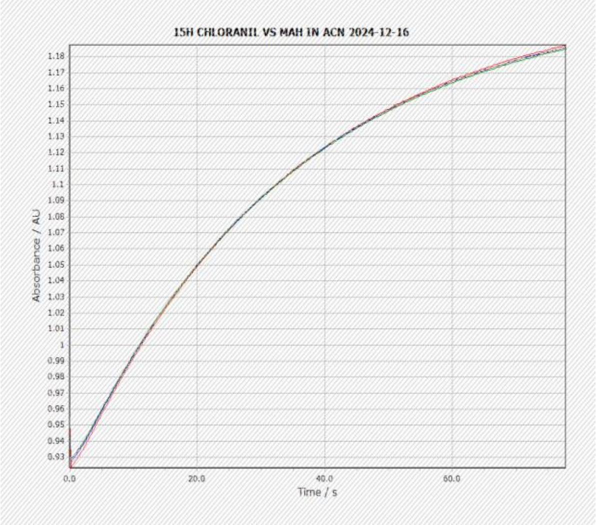	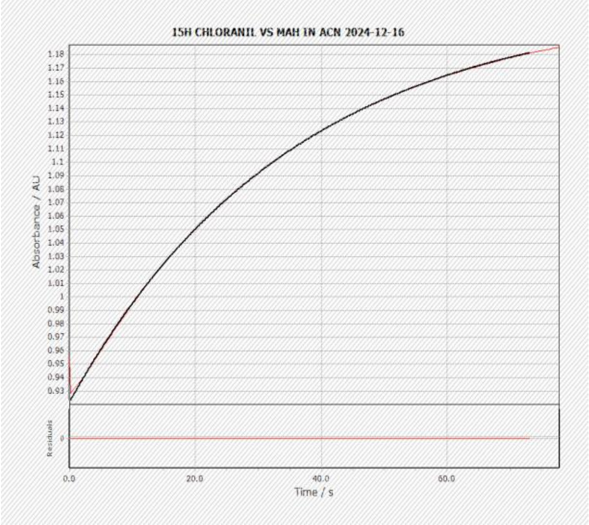
15°C H	

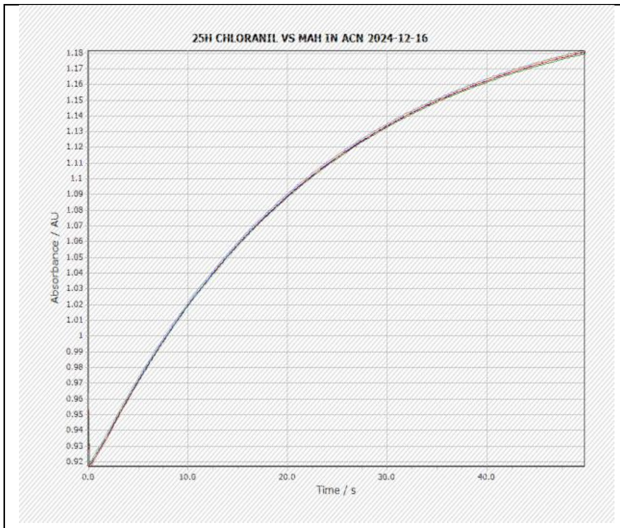
25H CHLORANIL VS MAH IN ACN 2024-12-16
Absorbance / AU
Time / s

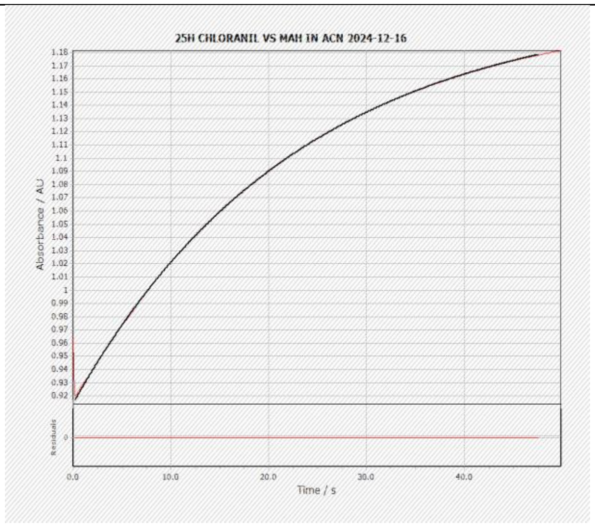
25H CHLORANIL VS MAH IN ACN 2024-12-16
Absorbance / AU
Residuals
Time / s

25°C H

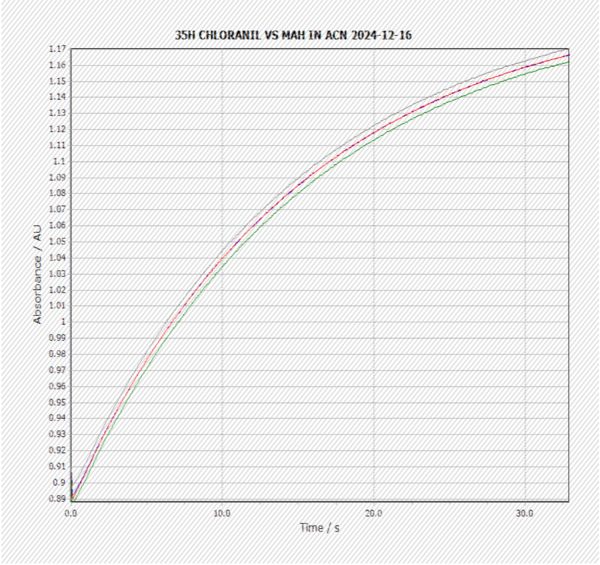
35H CHLORANIL VS MAH IN ACN 2024-12-16
Absorbance / AU
Time / s

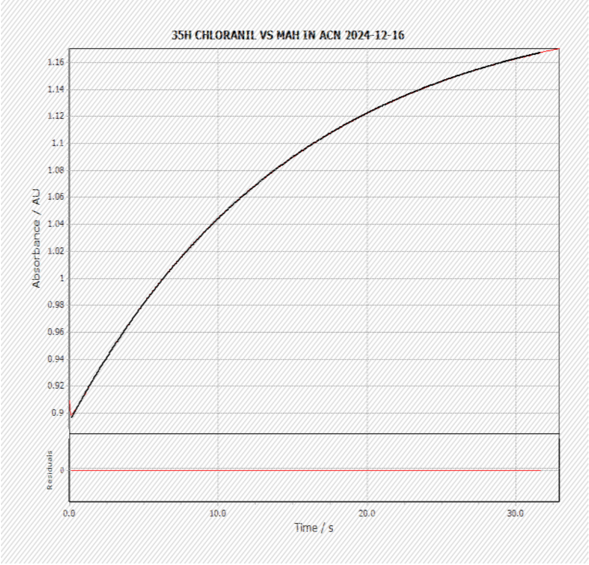
35H CHLORANIL VS MAH IN ACN 2024-12-16
Absorbance / AU
Residuals
Time / s

35°C H

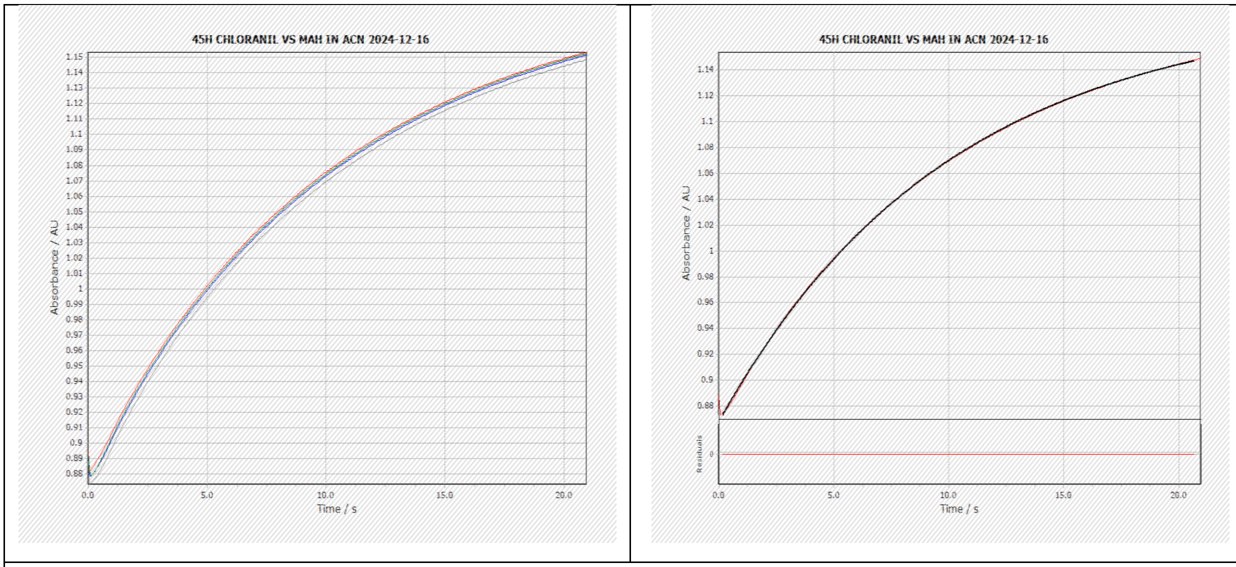
45H CHLORANIL VS MAH IN ACN 2024-12-16
Absorbance / AU
Time / s
45H CHLORANIL VS MAH IN ACN 2024-12-16
Absorbance / AU
Residuals
Time / s

45°C H

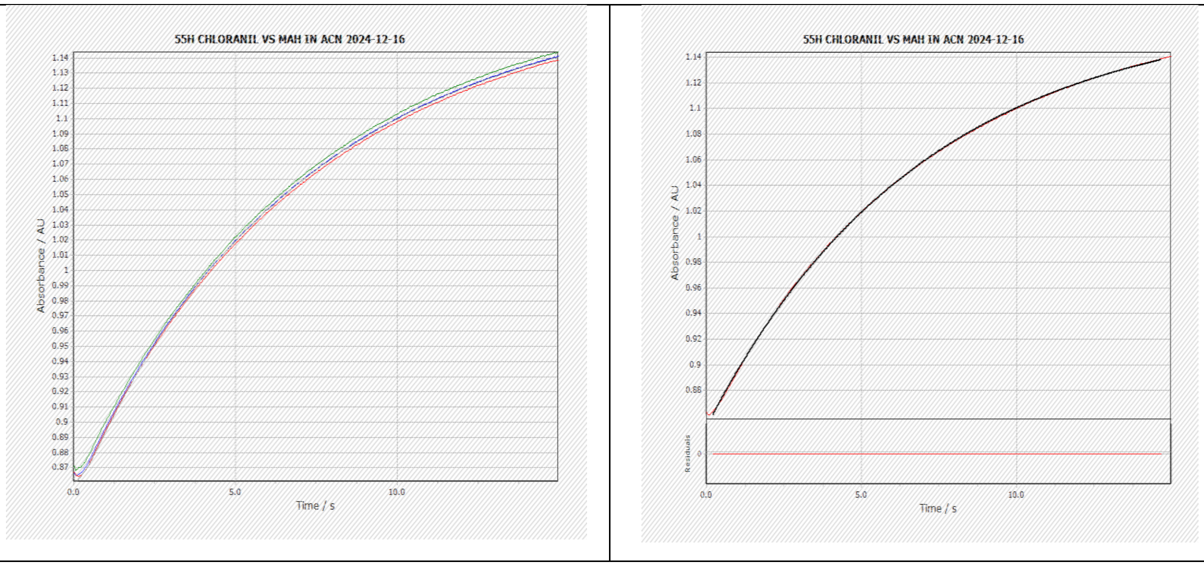
55H CHLORANIL VS MAH IN ACN 2024-12-16
Absorbance / AU
Time / s
55H CHLORANIL VS MAH IN ACN 2024-12-16
Absorbance / AU
Residuals
Time / s

55°C H

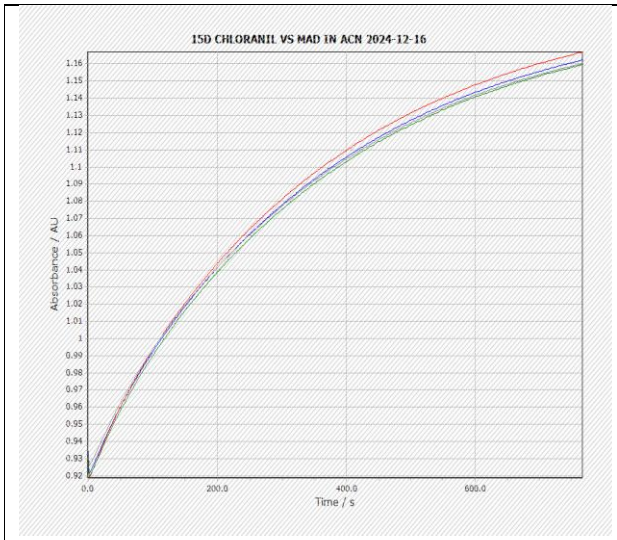

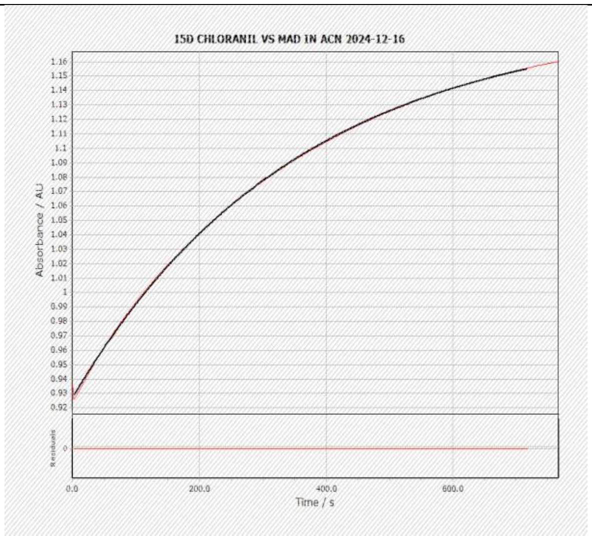

15°C D

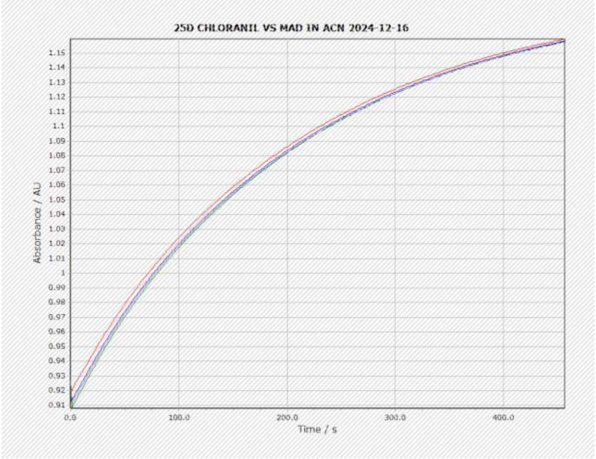

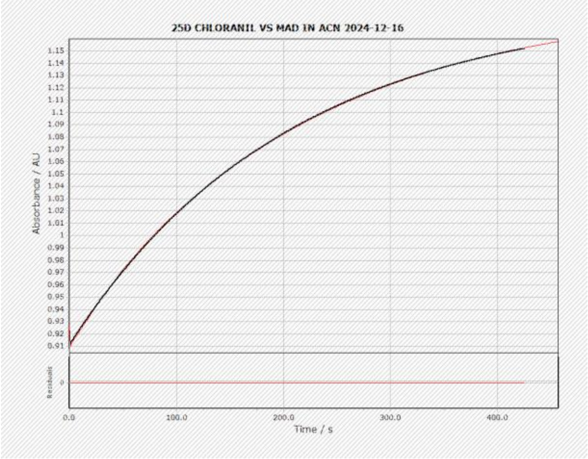

25°C D

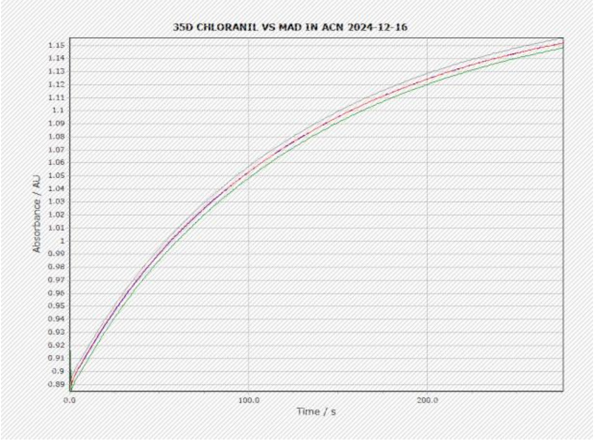

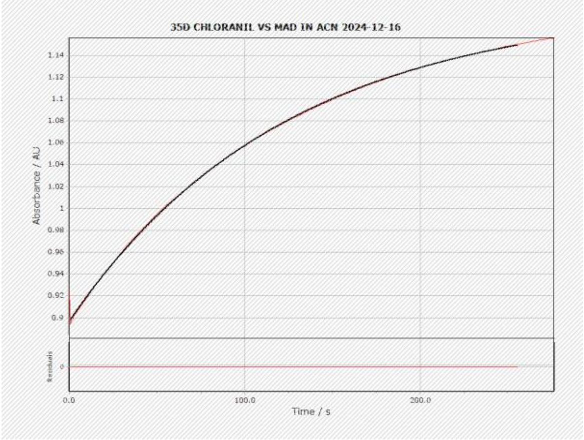

35°C D

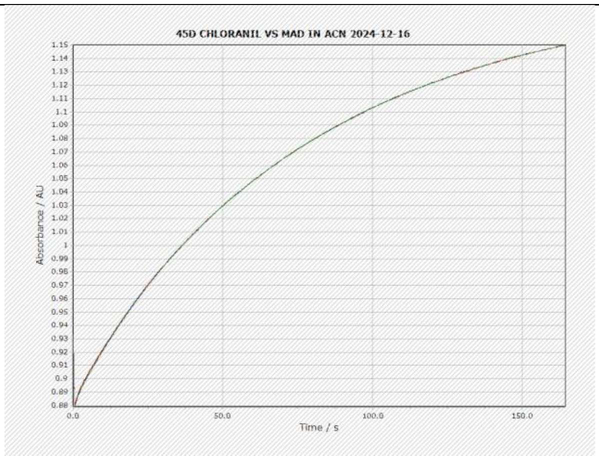

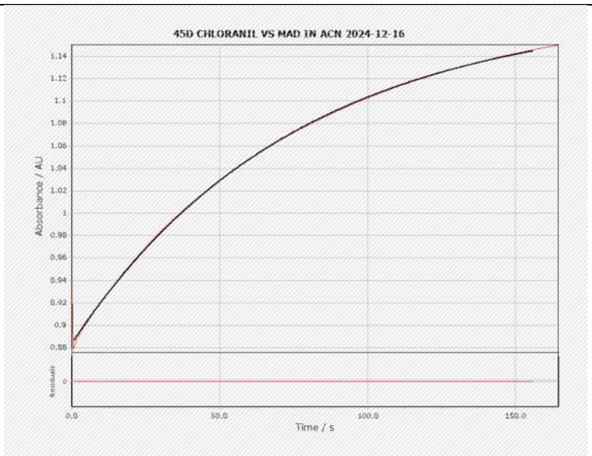

45℃ D

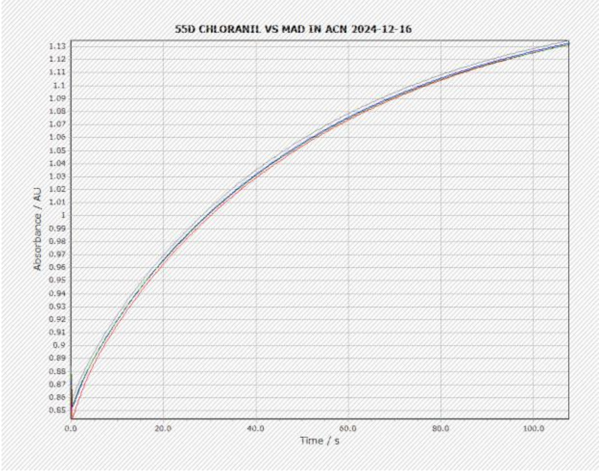

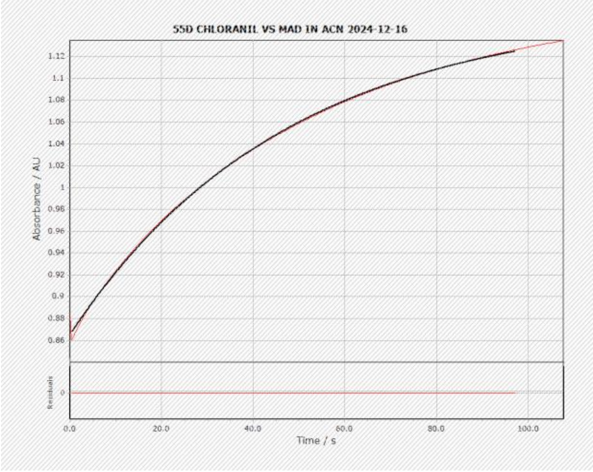

55℃ D

Three kinetic runs together with the averaged data	First-order kinetic fit of the averaged data as an example

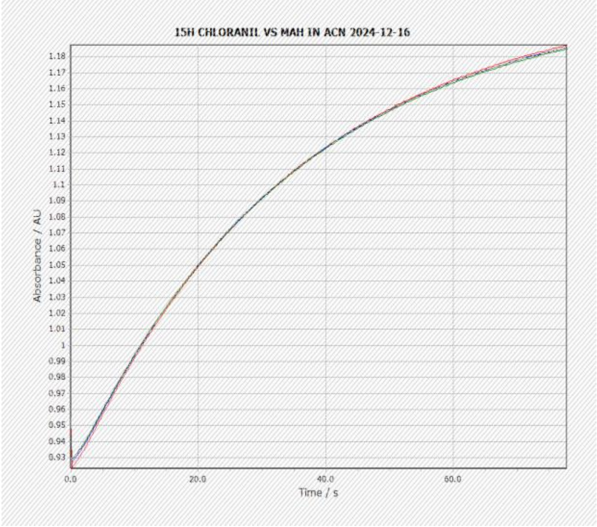

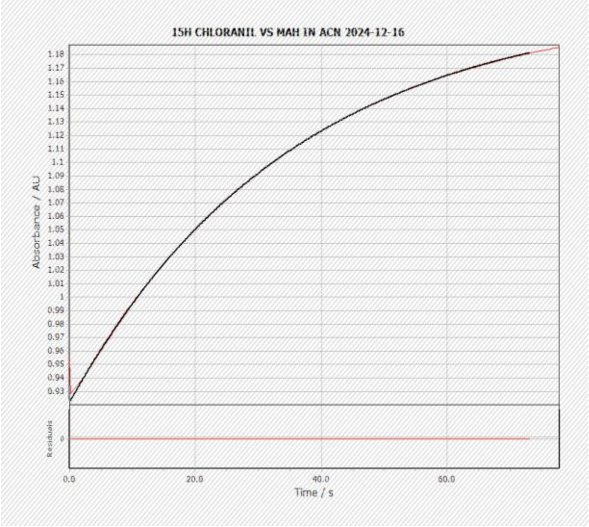

15℃ H

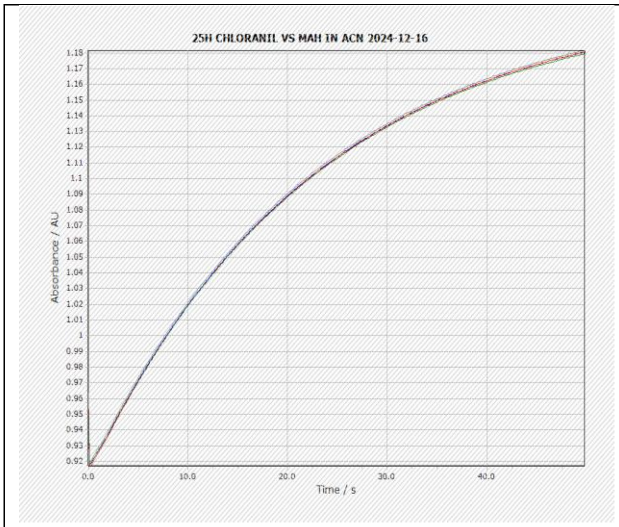
25H CHLORANIL VS MAH IN ACN 2024-12-16
Absorbance / AU
Time / s

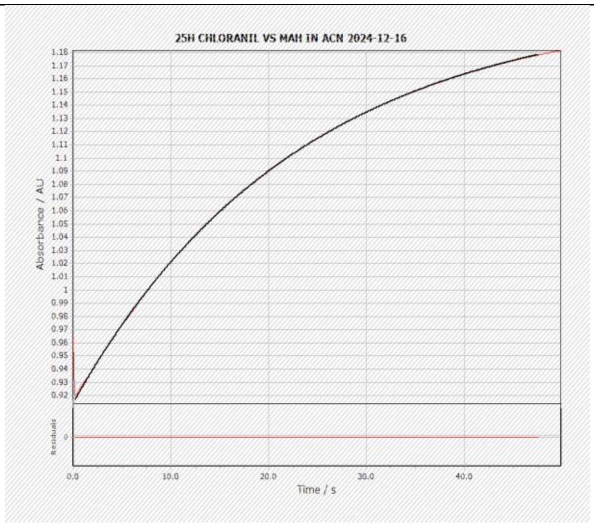
25H CHLORANIL VS MAH IN ACN 2024-12-16
Absorbance / AU
Residuals
Time / s

25°C H

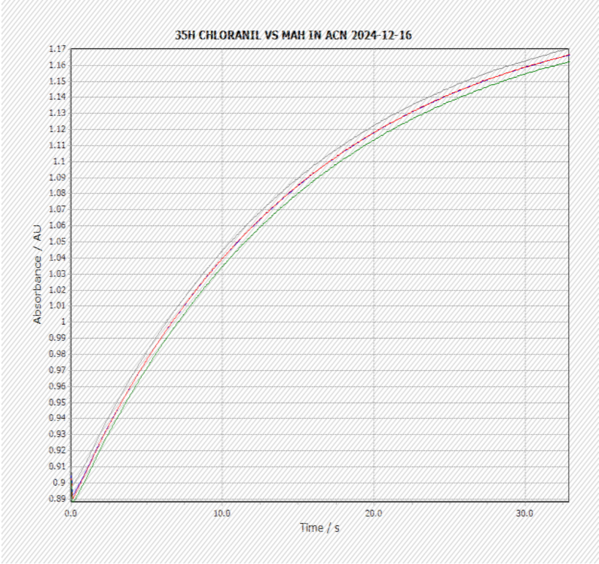
35H CHLORANIL VS MAH IN ACN 2024-12-16
Absorbance / AU
Time / s

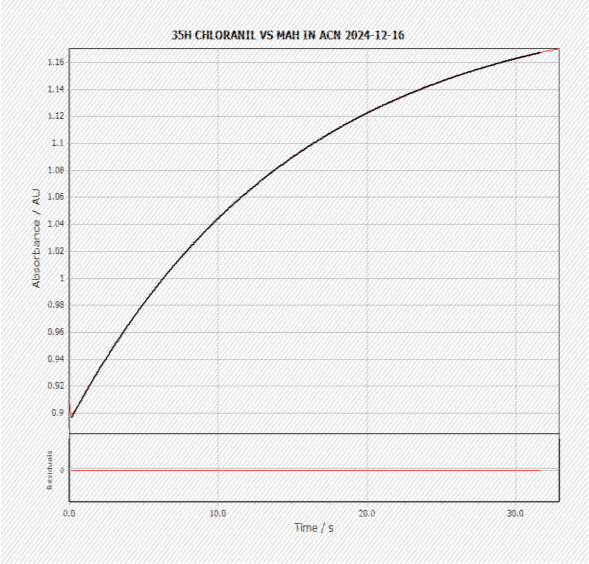
35H CHLORANIL VS MAH IN ACN 2024-12-16
Absorbance / AU
Residuals
Time / s

35°C H

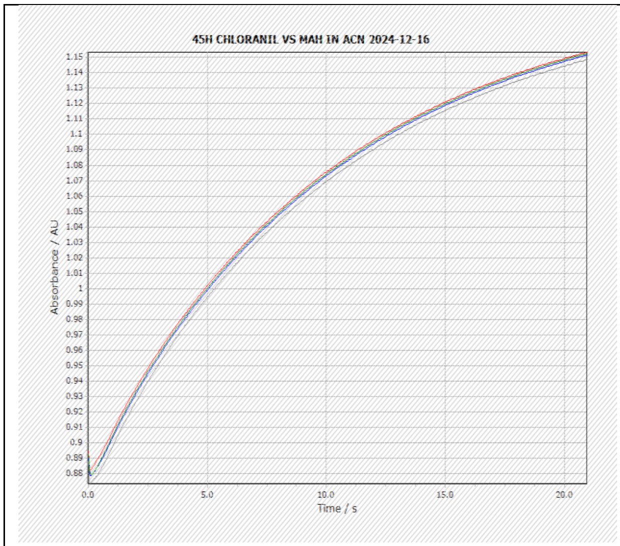
45H CHLORANIL VS MAH IN ACN 2024-12-16
Absorbance / AU
Time / s

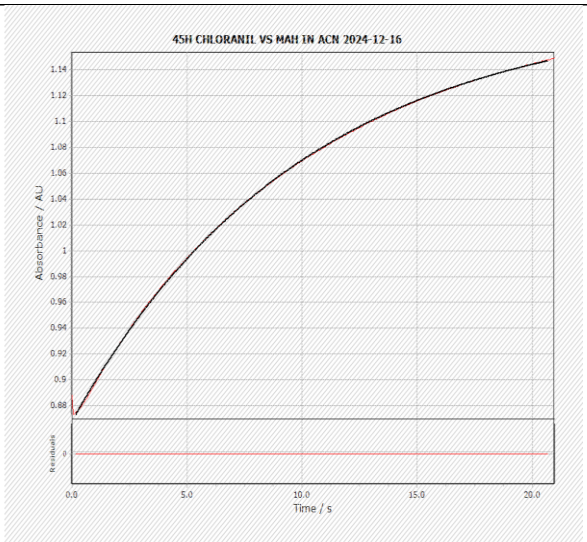
45H CHLORANIL VS MAH IN ACN 2024-12-16
Absorbance / AU
Residuals
Time / s

45℃ H

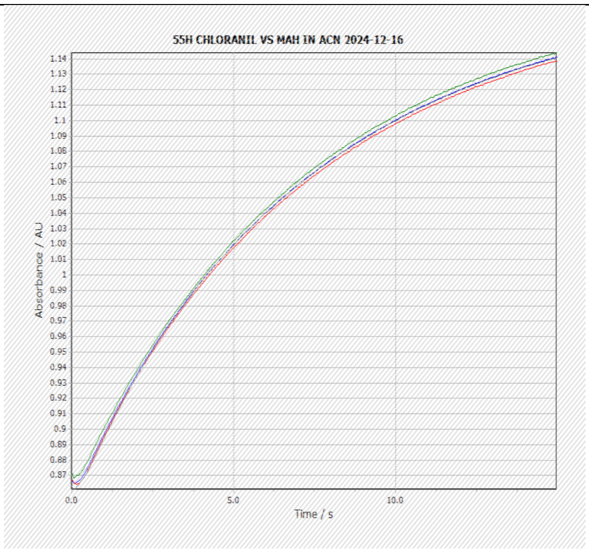
55H CHLORANIL VS MAH IN ACN 2024-12-16
Absorbance / AU
Time / s

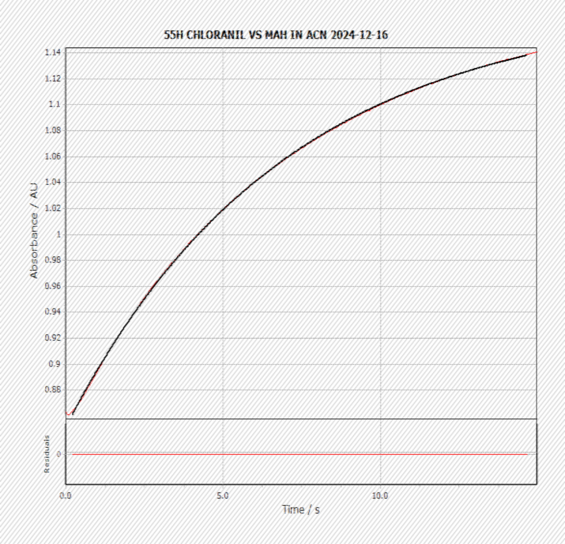
55H CHLORANIL VS MAH IN ACN 2024-12-16
Absorbance / AU
Residuals
Time / s

55℃ H

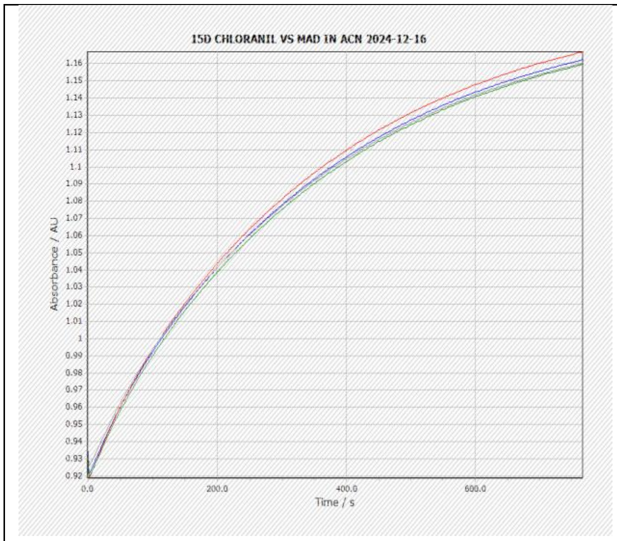

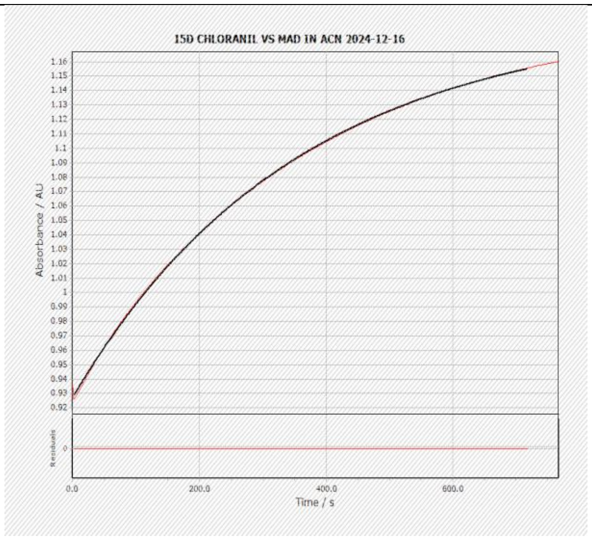

15°C D

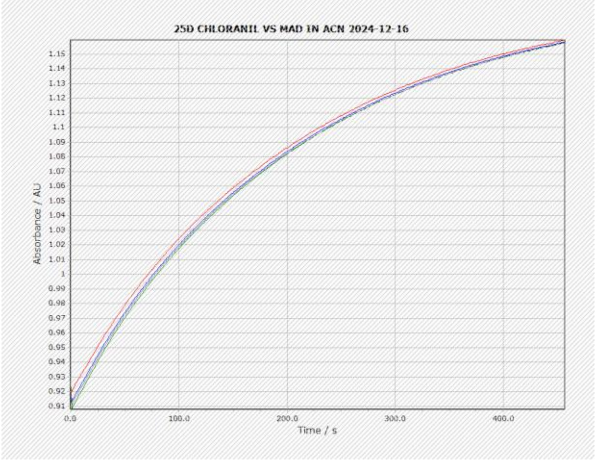

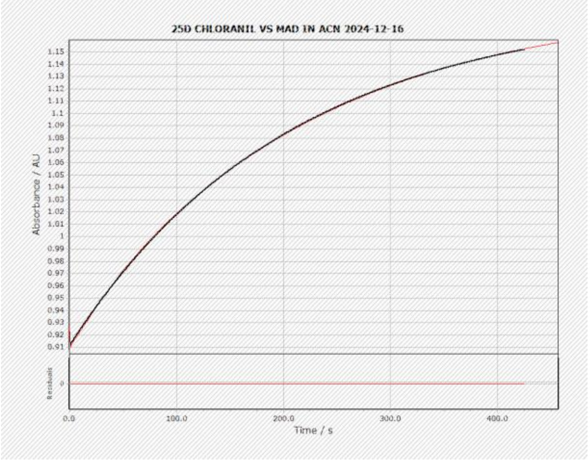

25°C D

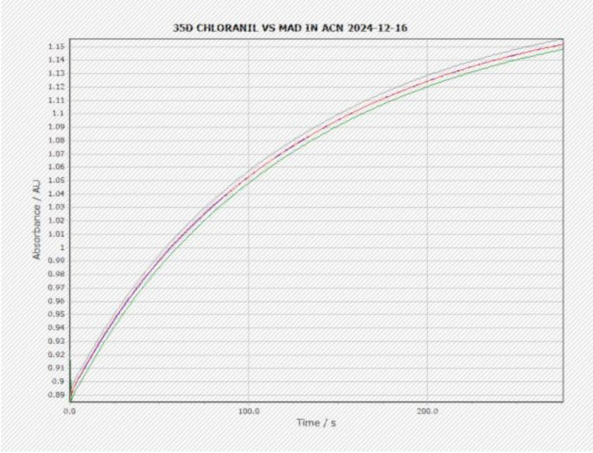

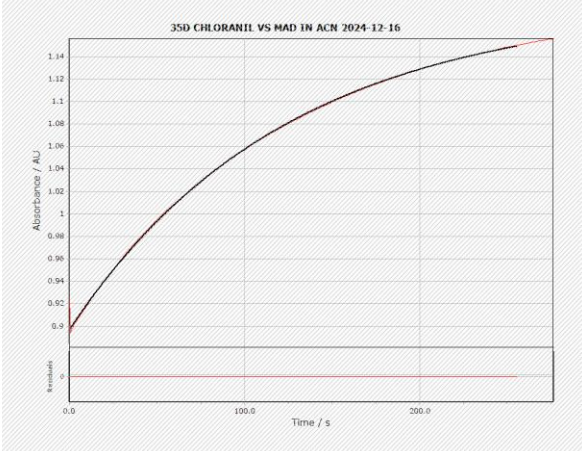

35°C D

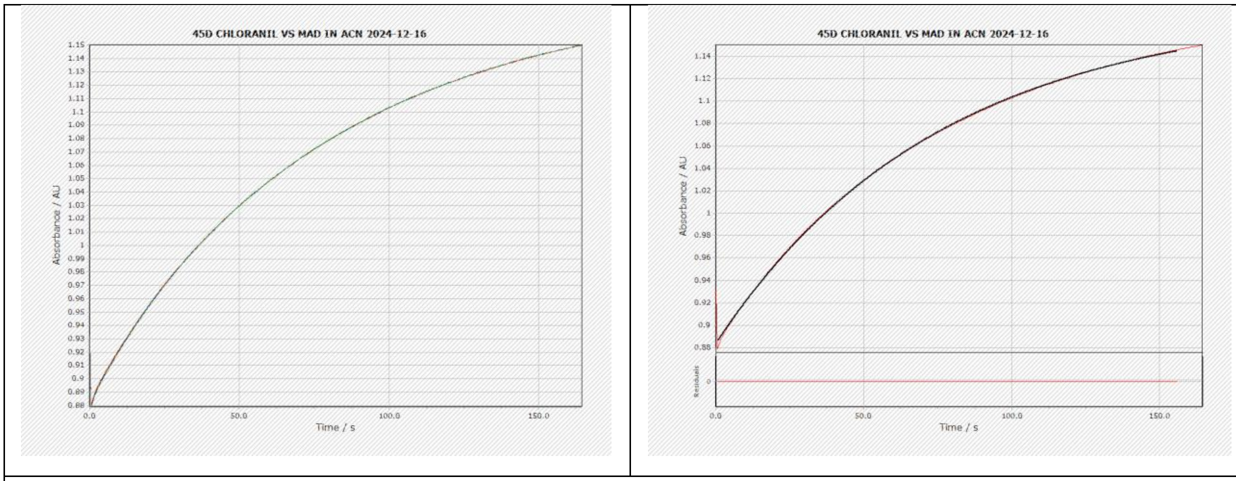

45°C D

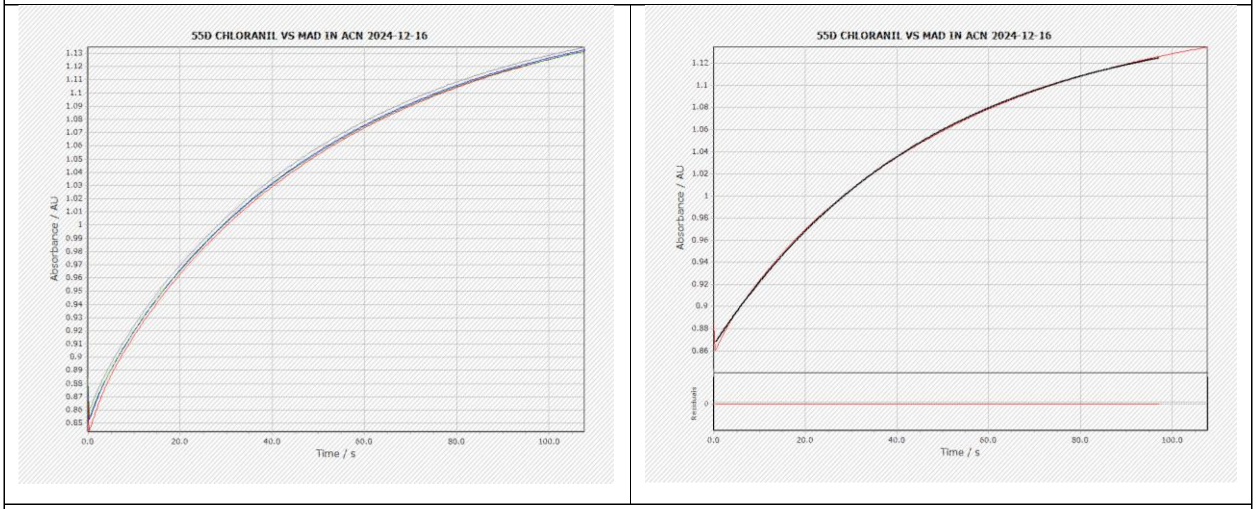

55°C D

Day 2 data (December 17, 2024)

Pseudo-first-order rate constants

k^{pfo} (s^{-1})

Temp (°C)	Trial H1	Trial H2	Trial H3	Average k_H^{pfo} (s^{-1})	Stdev	k_{2H} [b] (M^{-1}s^{-1})	Stdev [a]
55	0.13757	0.13975	0.1439	0.1404	0.003216	5.98E+01	1.37032
45	0.09611	0.09866	0.09606	0.0969	0.001487	4.13E+01	0.63362
35	0.06676	0.06718	0.06657	0.0668	0.000312	2.85E+01	0.13302
25	0.04337	0.04347	0.04301	0.0433	0.000242	1.84E+01	0.10310
15	0.02858	0.02849	0.02836	0.0285	0.000111	1.21E+01	0.04713

Temp (°C)	Trial D1	Trial D2	Trial D3	Average k_D^{pfo} (s^{-1})	Stdev	k_{2D} [b] (M^{-1}s^{-1})	Stdev [a]
55	0.02045	0.02044	0.02023	0.0204	0.000124	8.68E+00	0.052939
45	0.01305	0.01289	0.0129	0.0129	8.96E-05	5.52E+00	0.03819
35	0.008	0.00798	0.00798	0.0080	1.15E-05	3.40E+00	0.00492
25	0.00504	0.00499	0.00489	0.0050	7.64E-05	2.12E+00	0.03255
15	0.00285	0.0028	0.00281	0.0028	2.65E-05	1.20E+00	0.01127

[a] = (Stdev(for k^{pfo})/k^{pfo})*k_2; [b] = k^{pfo}/(2[Cl_4Q]/3)

<table>
<tr><td>Three kinetic runs together with the averaged data</td><td>First-order kinetic fit of the averaged data as an example</td></tr>
<tr><td>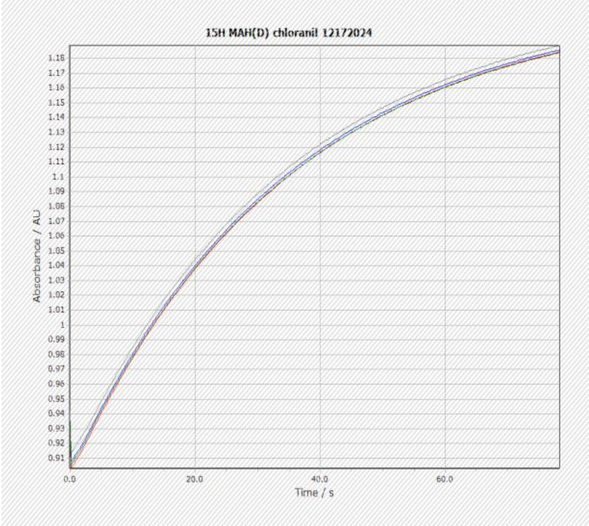
</td><td>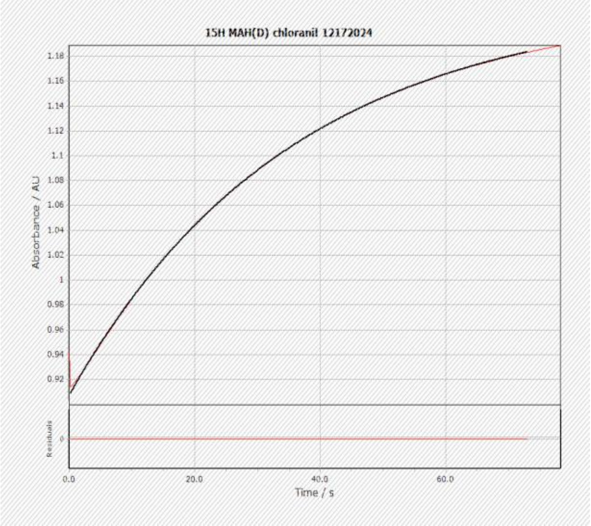
</td></tr>
<tr><td colspan="2">15°C H</td></tr>
<tr><td>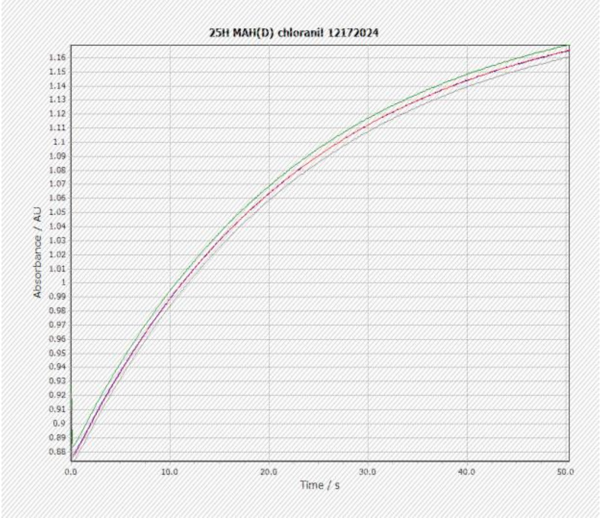
</td><td>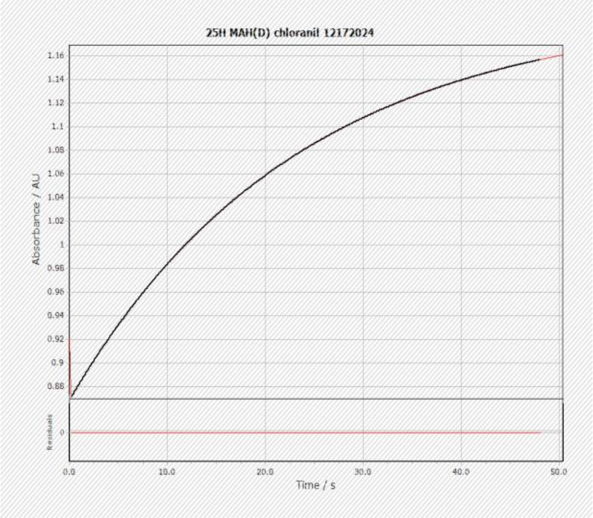
</td></tr>
<tr><td colspan="2">25°C H</td></tr>
</table>

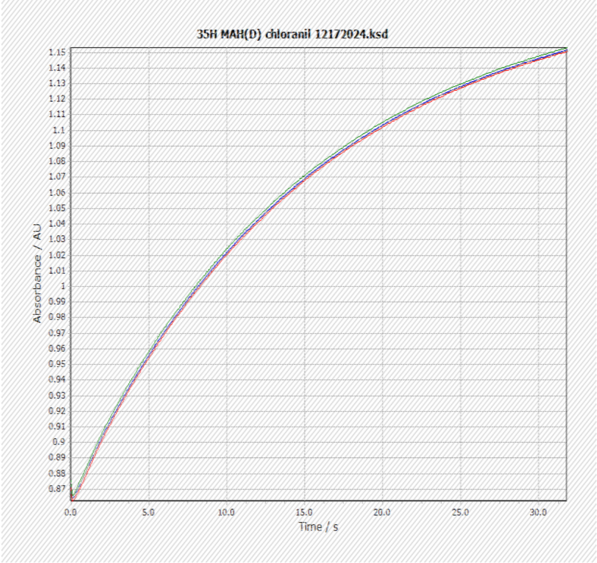
35H MAH(D) chloranil 12172024.ksd
Absorbance / AU
Time / s

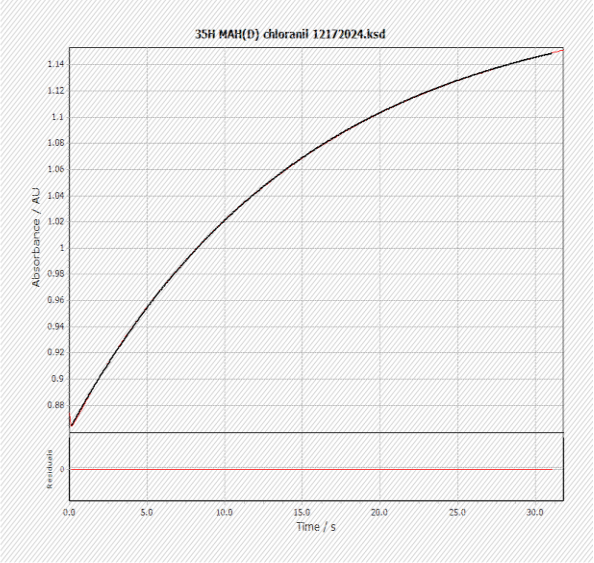
35H MAH(D) chloranil 12172024.ksd
Absorbance / AU
Residuals
Time / s

35°C H

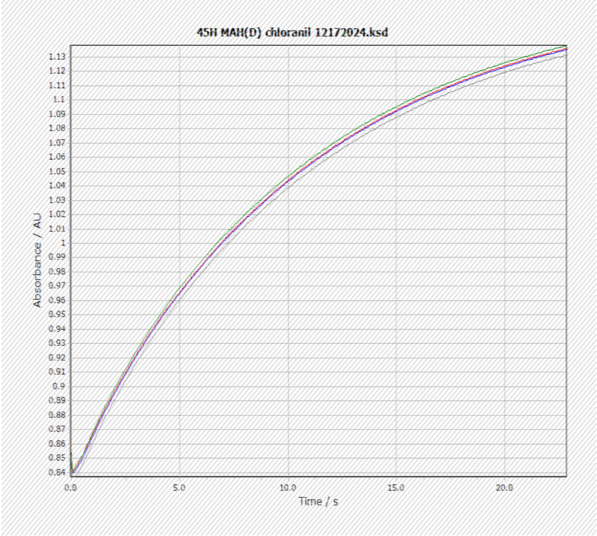
45H MAH(D) chloranil 12172024.ksd
Absorbance / AU
Time / s

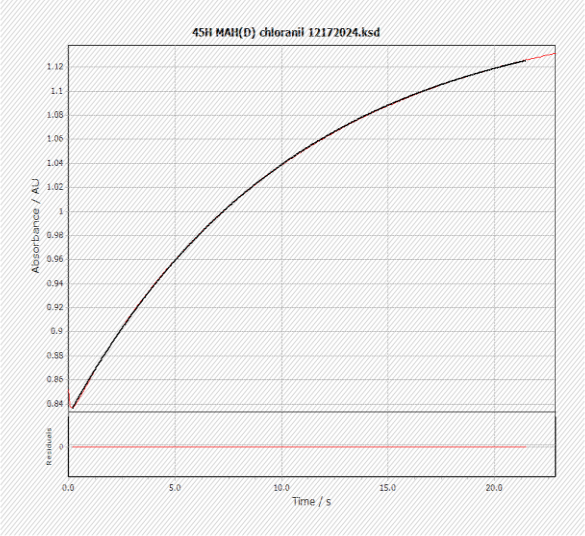
45H MAH(D) chloranil 12172024.ksd
Absorbance / AU
Residuals
Time / s

45°C H

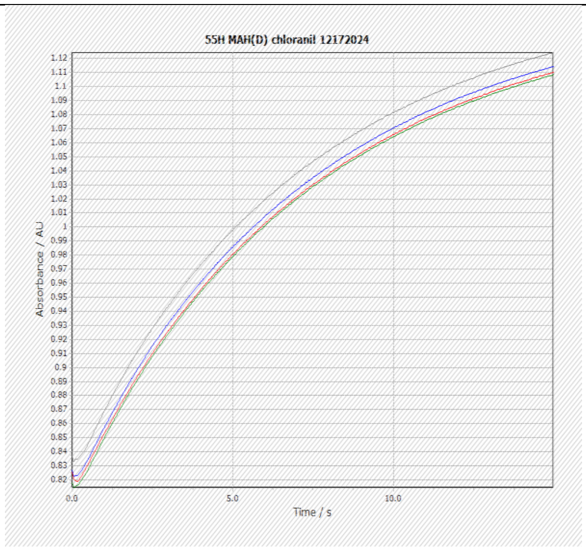

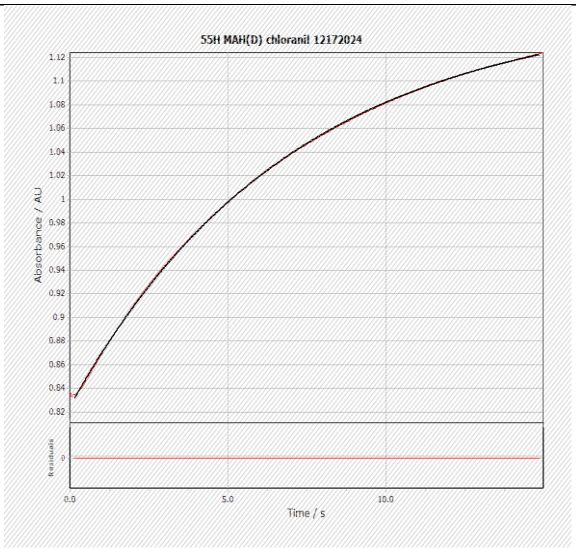

55°C H

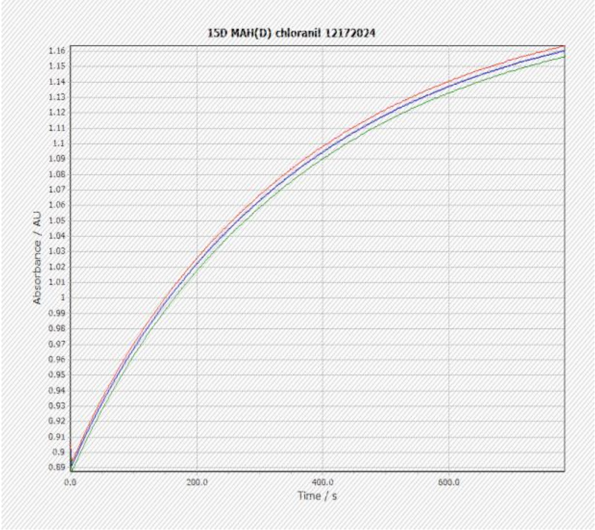

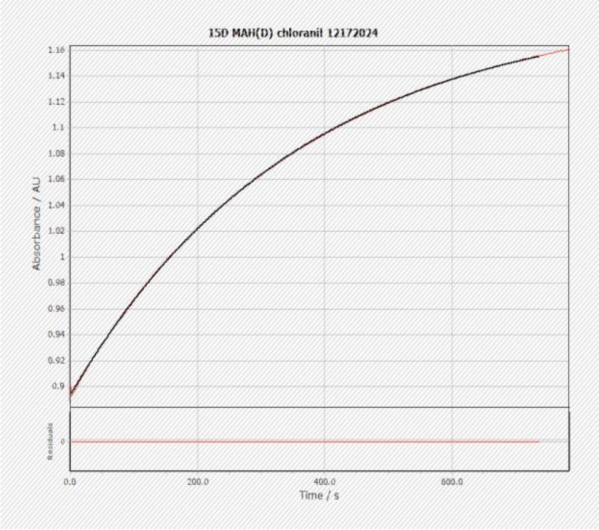

15°C D

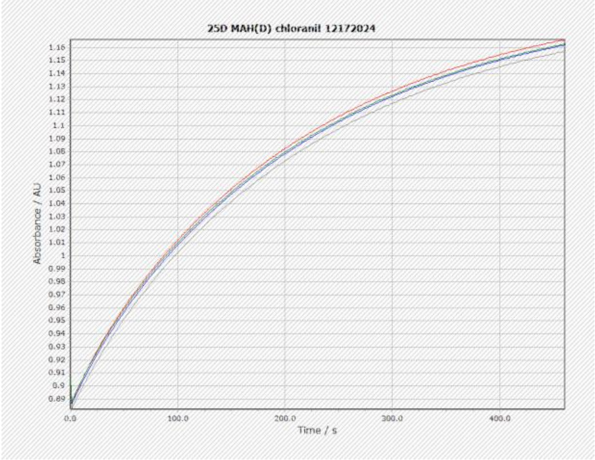

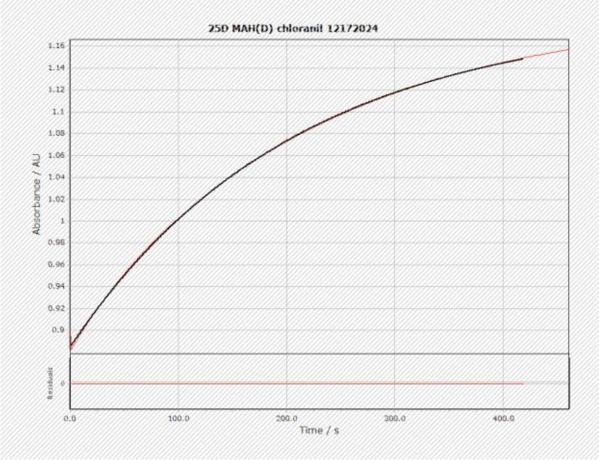

25°C D

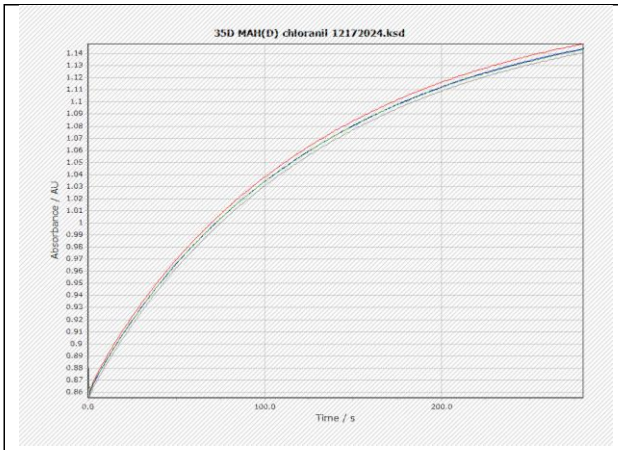

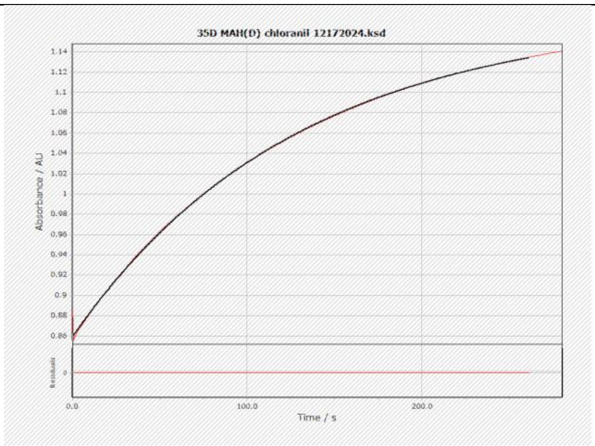

35°C D

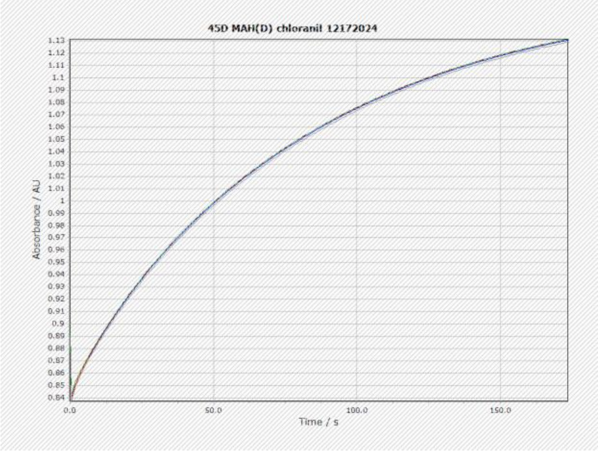

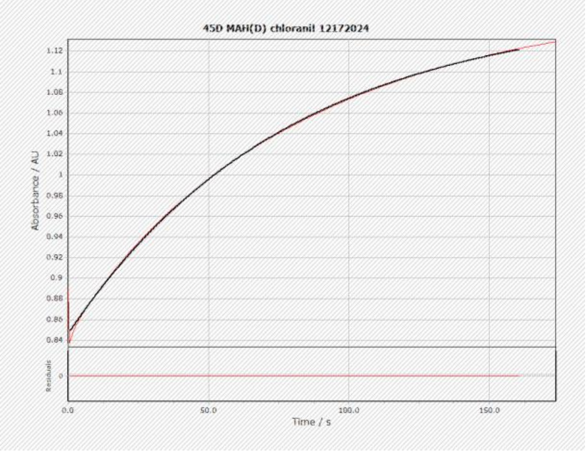

45°C D

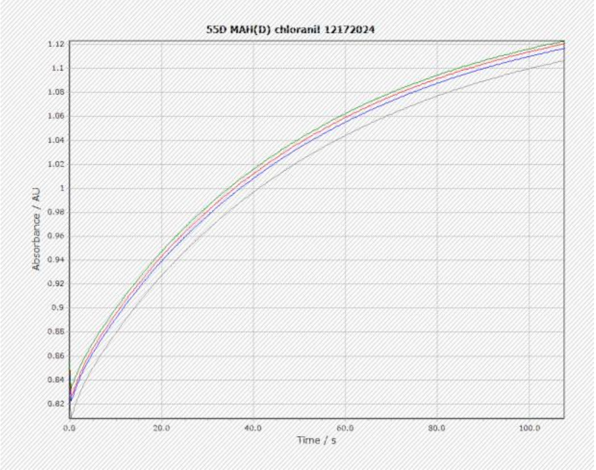

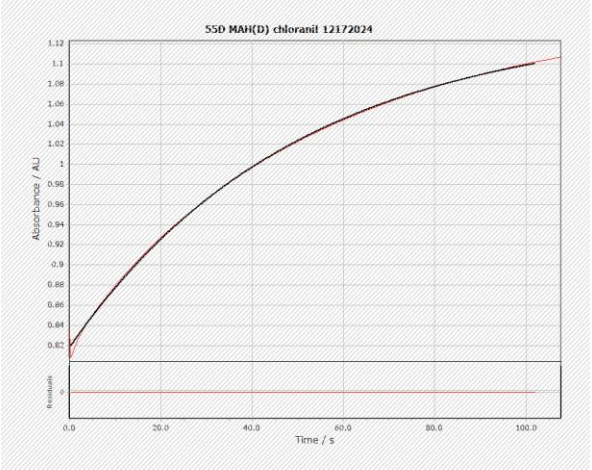

55°C D

Day 3 data (December 23, 2024)

Pseudo-first-order rate constants

k^{pfo} (s^{-1})

Temp (°C)	Trial H1	Trial H2	Trial H3	Average k_H^{pfo} (s^{-1})	Stdev	k_{2H} [b] ($M^{-1}s^{-1}$)	Stdev [a]
55	0.15609	0.15377	0.1483	0.1527	0.004	6.51E+01	1.70443
45	0.10114	0.10277	0.10100	0.1016	0.000984	4.33E+01	0.41931
35	0.06745	0.06739	0.06723	0.0674	0.000114	2.87E+01	0.04846
25	0.04579	0.04597	0.04542	0.0457	0.00028	1.95E+01	0.11950
15	0.02911	0.02924	0.02908	0.0291	8.5E-05	1.24E+01	0.03624

Temp (°C)	Trial D1	Trial D2	Trial D3	Average k_D^{pfo} (s^{-1})	Stdev	k_{2D} [b] ($M^{-1}s^{-1}$)	Stdev [a]
55	0.0214	0.02089	0.02077	0.0210	0.000335	8.96E+00	0.142549
45	0.01336	0.0133	0.01325	0.0133	5.51E-05	5.67E+00	0.02347
35	0.00839	0.00831	0.00846	0.0084	7.51E-05	3.57E+00	0.03198
25	0.00505	0.00505	0.00506	0.0051	5.77E-06	2.15E+00	0.00246
15	0.00293	0.00293	0.00292	0.0029	5.77E-06	1.25E+00	0.00246

[a] = (Stdev(for k^{pfo})/k^{pfo})*k_2; [b] = k^{pfo}/(2[Cl_4Q]/3)

Three kinetic runs together with the averaged data	First-order kinetic fit of the averaged data as an example
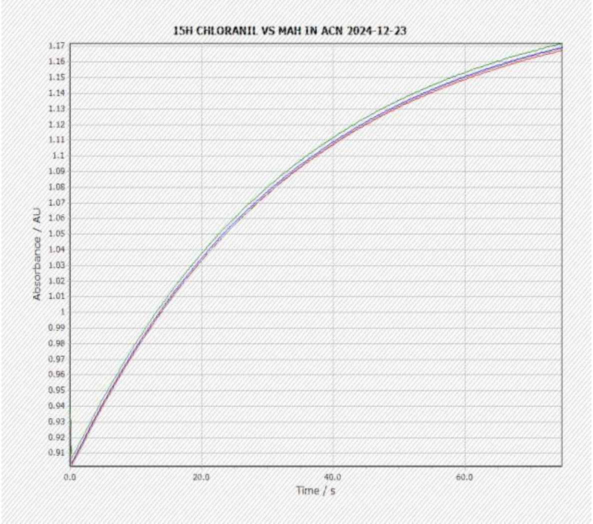	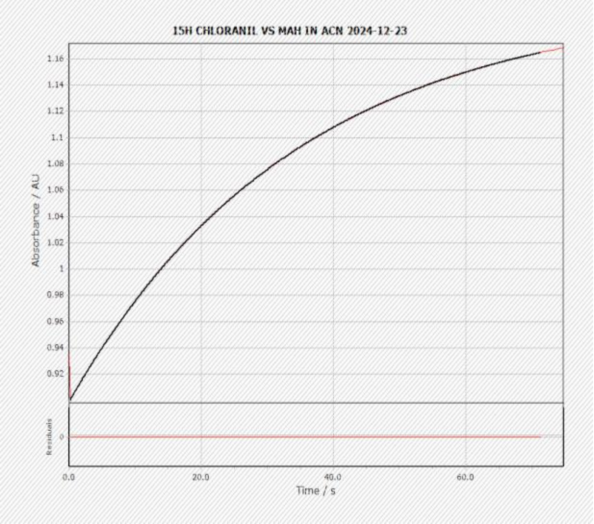

15°C H

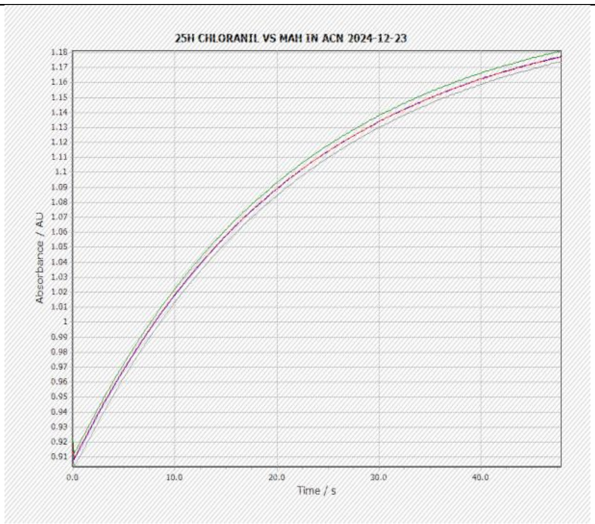
25H CHLORANIL VS MAH IN ACN 2024-12-23
Absorbance / AU
Time / s

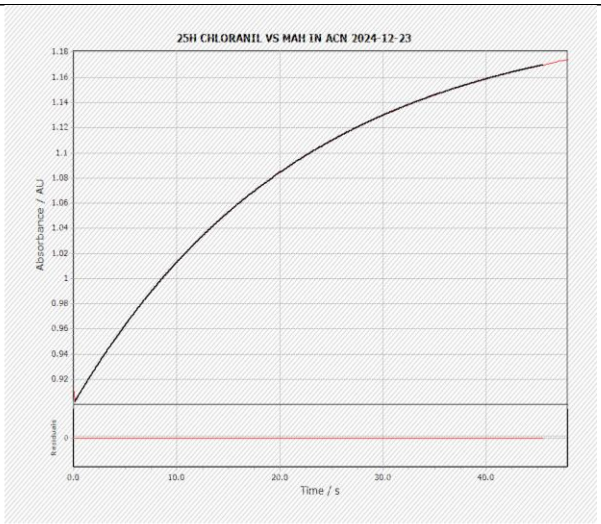
25H CHLORANIL VS MAH IN ACN 2024-12-23
Absorbance / AU
Residuals
Time / s

25°C H

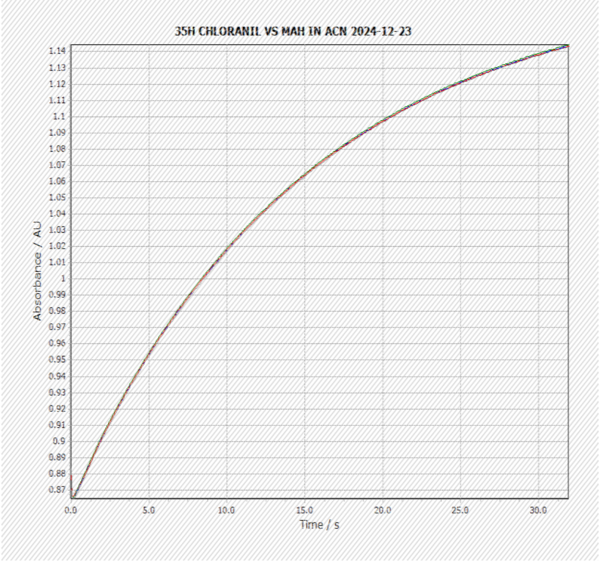
35H CHLORANIL VS MAH IN ACN 2024-12-23
Absorbance / AU
Time / s

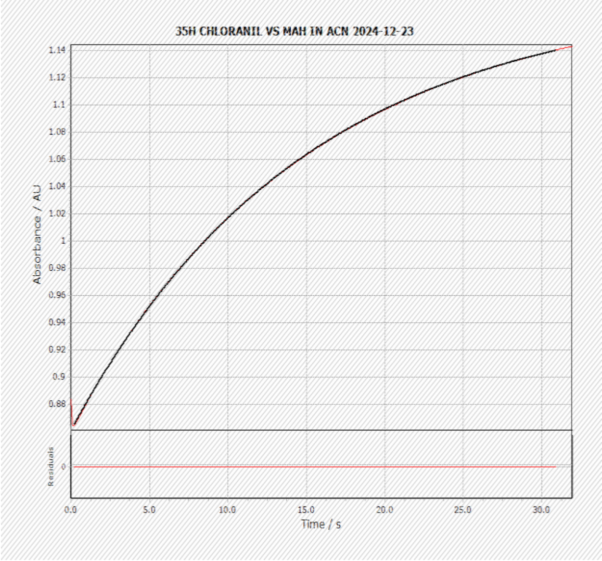
35H CHLORANIL VS MAH IN ACN 2024-12-23
Absorbance / AU
Residuals
Time / s

35°C H

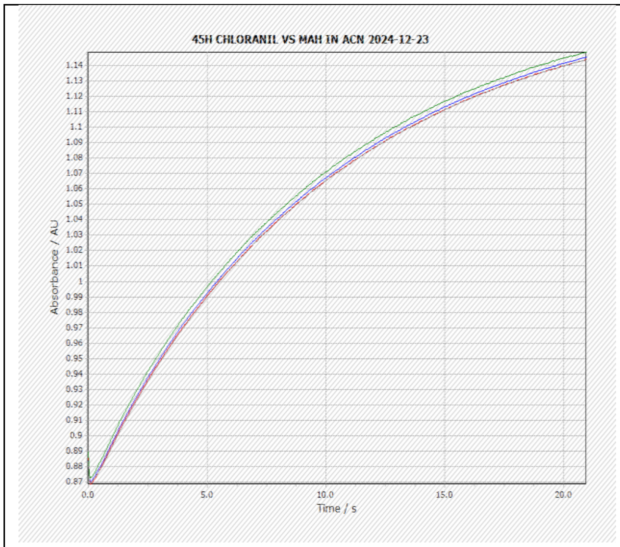
45H CHLORANIL VS MAH IN ACN 2024-12-23
Absorbance / AU
Time / s

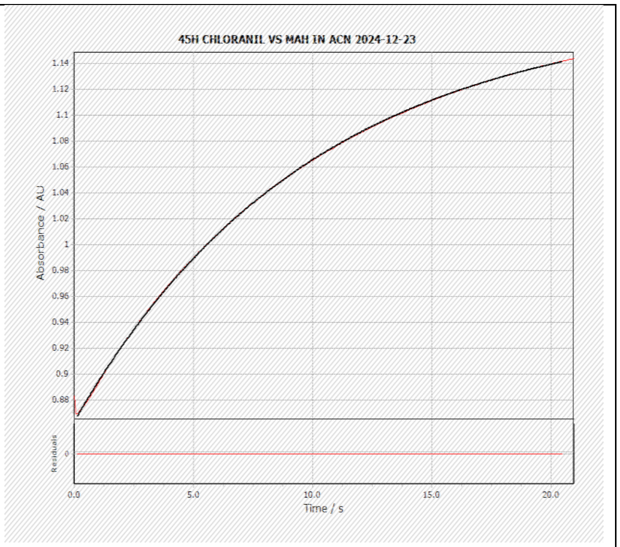
45H CHLORANIL VS MAH IN ACN 2024-12-23
Absorbance / AU
Residuals
Time / s

45°C H

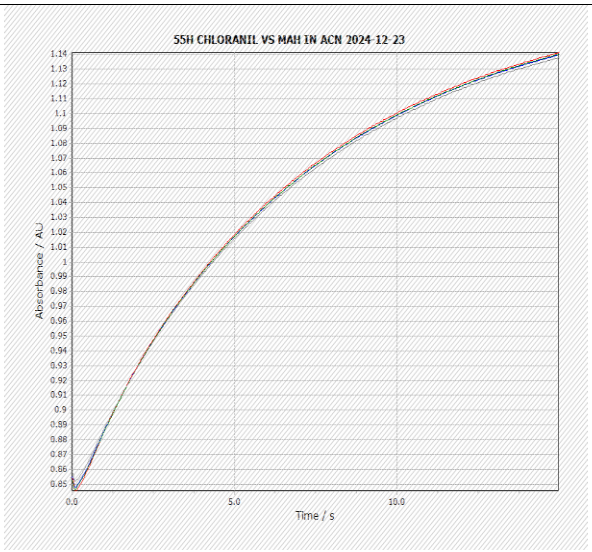
55H CHLORANIL VS MAH IN ACN 2024-12-23
Absorbance / AU
Time / s

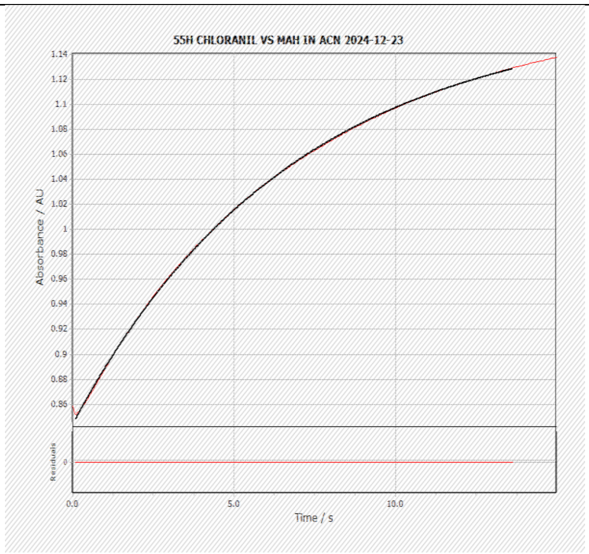
55H CHLORANIL VS MAH IN ACN 2024-12-23
Absorbance / AU
Residuals
Time / s

55°C H

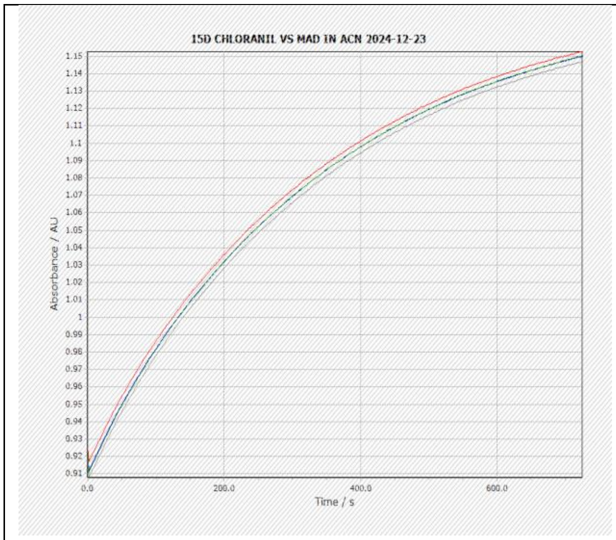

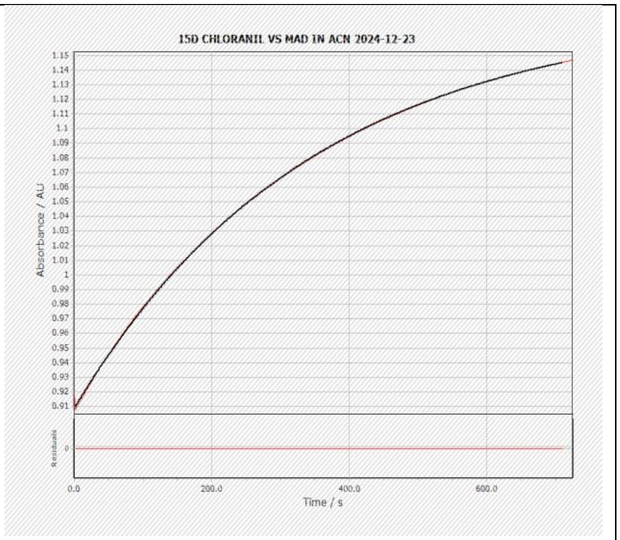

15°C D

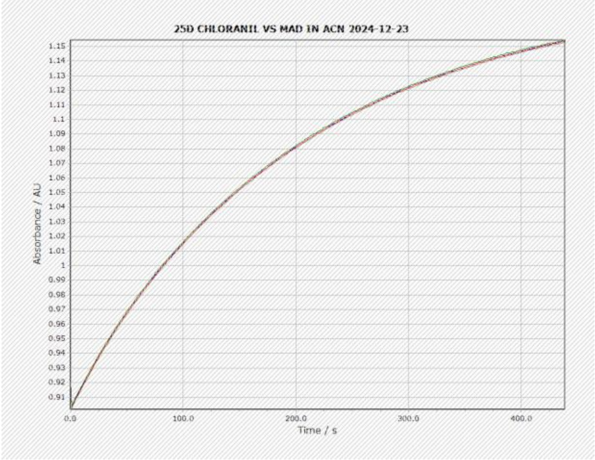

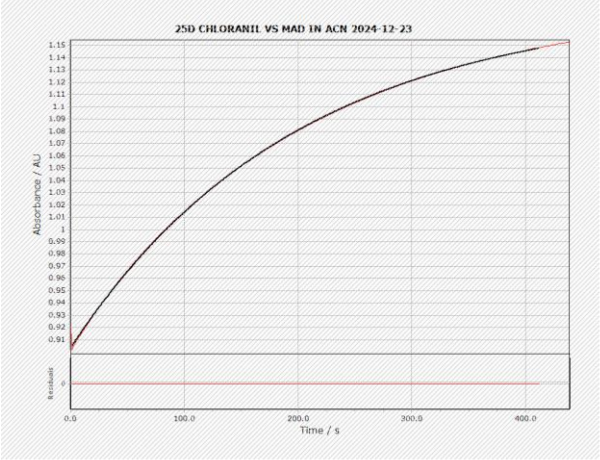

25°C D

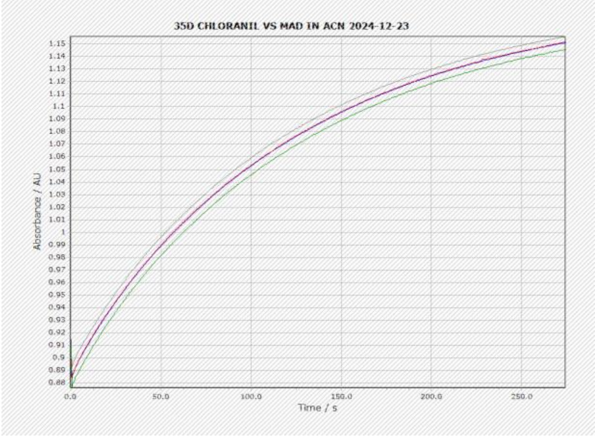

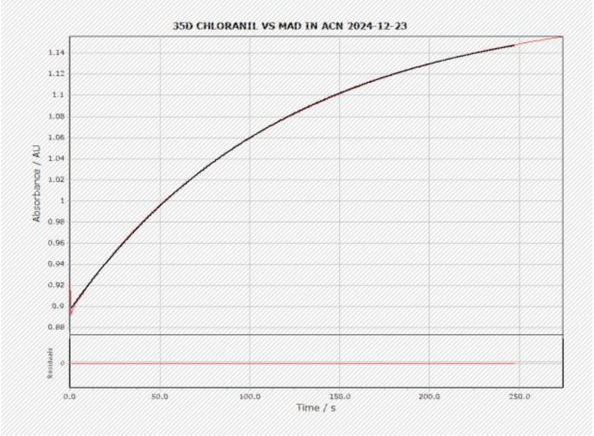

35°C D

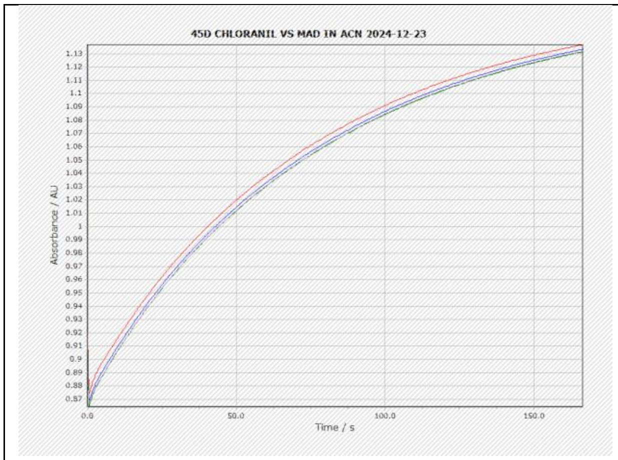
45D CHLORANIL VS MAD IN ACN 2024-12-23
Absorbance / AU
Time / s

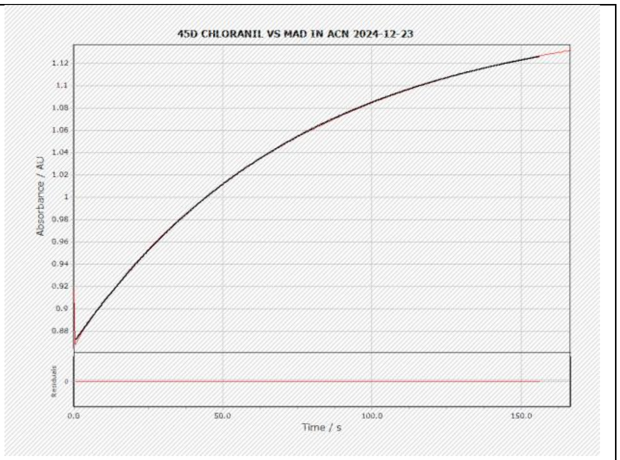
45D CHLORANIL VS MAD IN ACN 2024-12-23
Absorbance / AU
Residuals
Time / s

45°C D

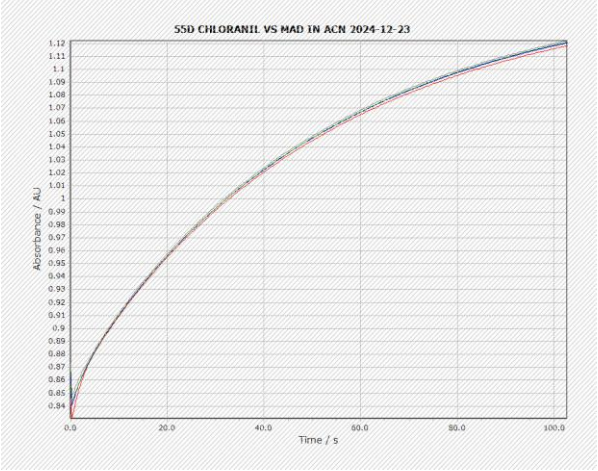
55D CHLORANIL VS MAD IN ACN 2024-12-23
Absorbance / AU
Time / s

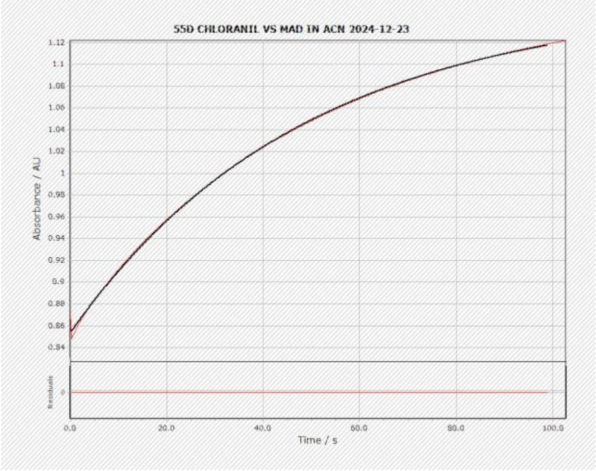
55D CHLORANIL VS MAD IN ACN 2024-12-23
Absorbance / AU
Residuals
Time / s

55°C D

Primary kinetic data for the rate constants in Table S3

Day 1 data (August 12, 2020)

	Pseudo-first-order rate constants k^{pfo} (s^{-1})						
**Temp (°C)**	**Trial H1**	**Trial H2**	**Trial H3**	**Average k_H^{pfo} (s^{-1})**	**Stdev**	**k_{2H} [b] ($M^{-1}s^{-1}$)**	**Stdev [a]**
55	0.20814	0.20852	0.21079	0.20915	0.001432934	5.94E+01	0.40708
45	0.14837	0.14729	0.14673	0.147463	0.000833627	4.19E+01	0.23683
35	0.09949	0.10046	0.10141	0.100453	0.000960017	2.85E+01	0.27273
25	0.06821	0.06786	0.06792	0.067997	0.000187172	1.93E+01	0.05317
15	0.04394	0.04405	0.04416	0.04405	0.00011	1.25E+01	0.03125
**Temp (°C)**	**Trial D1**	**Trial D2**	**Trial D3**	**Average k_D^{pfo} (s^{-1})**	**Stdev**	**k_{2D} [b] ($M^{-1}s^{-1}$)**	**Stdev [a]**
55	0.03314	0.03242	0.0326	0.03272	0.0003747	9.30E+00	0.10645
45	0.02093	0.02106	0.02111	0.02103	9.29157E-05	5.98E+00	0.02640
35	0.01331	0.01339	0.01358	0.01343	0.000138684	3.81E+00	0.03940
25	0.00793	0.00806	0.00808	0.00802	8.14453E-05	2.28E+00	0.02314
15	0.00484	0.00485	0.00482	0.00484	1.52753E-05	1.37E+00	0.00434

[a] = (Stdev(for k^{pfo})/k^{pfo})*k_2; [b] = k^{pfo}/[MAH]

Three kinetic runs together with the averaged data	First-order kinetic fit of the averaged data as an example
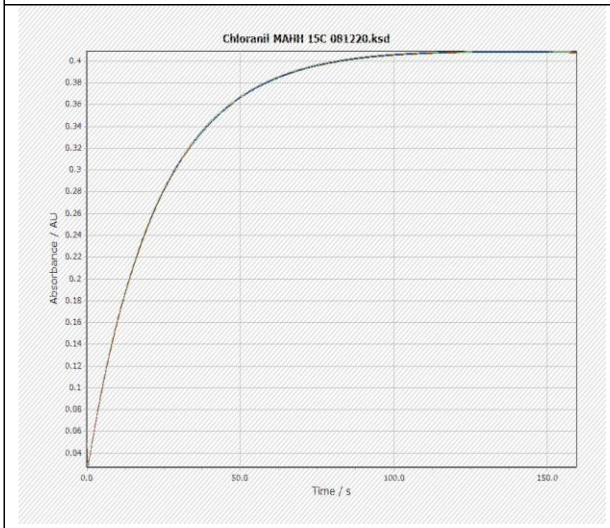	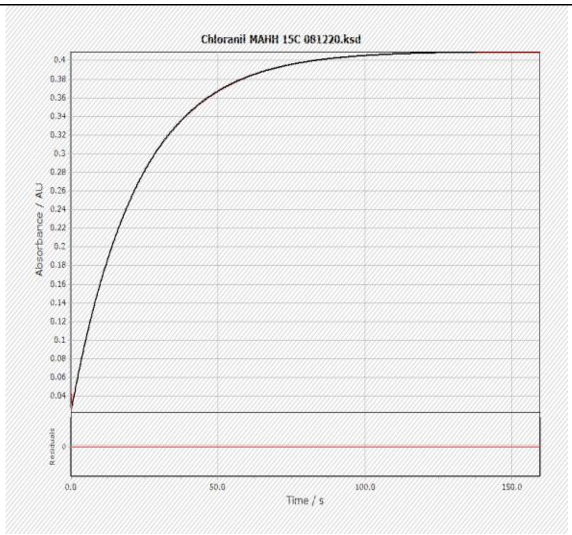
15°C H	

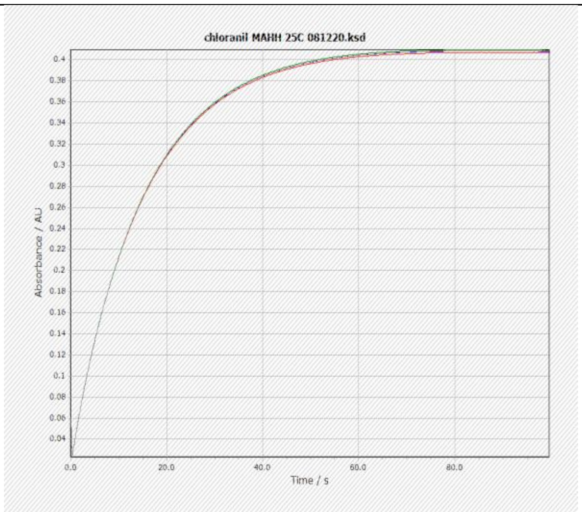

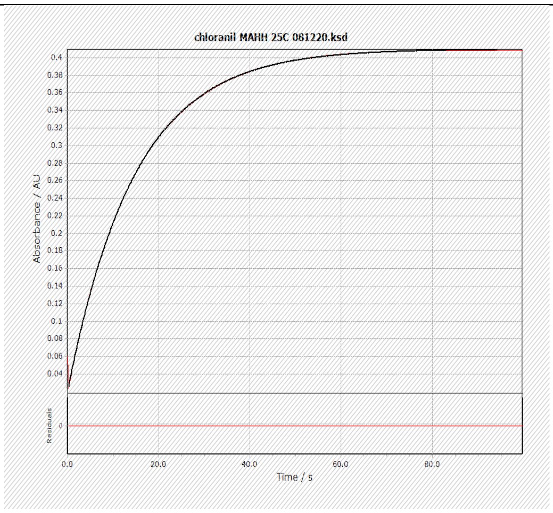

25°C H

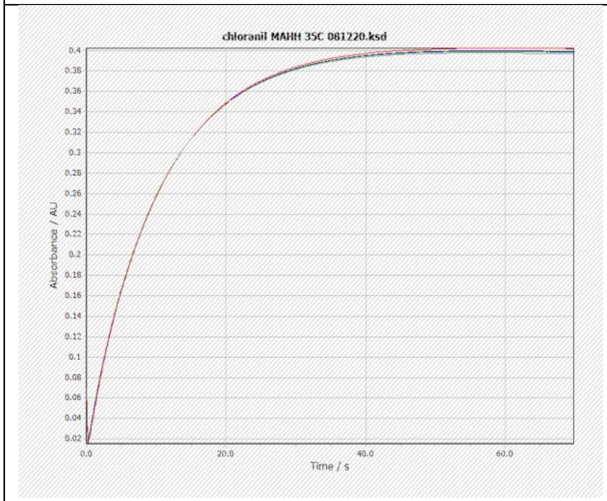

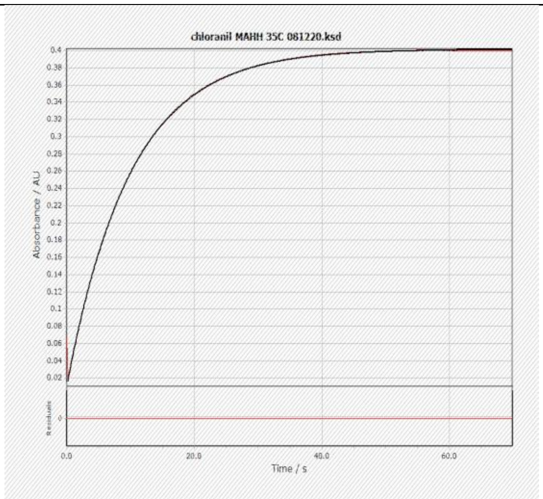

35°C H

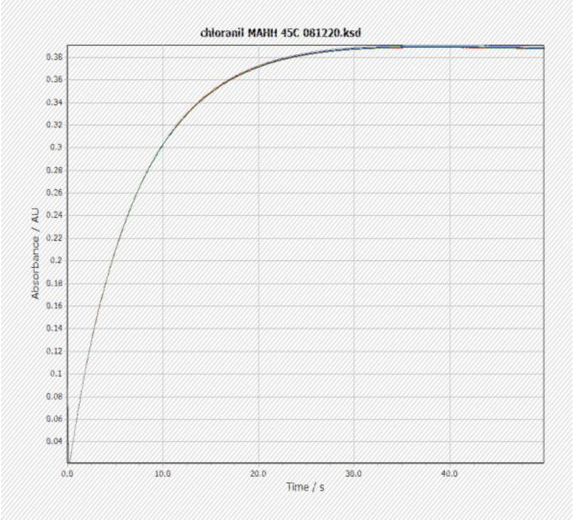

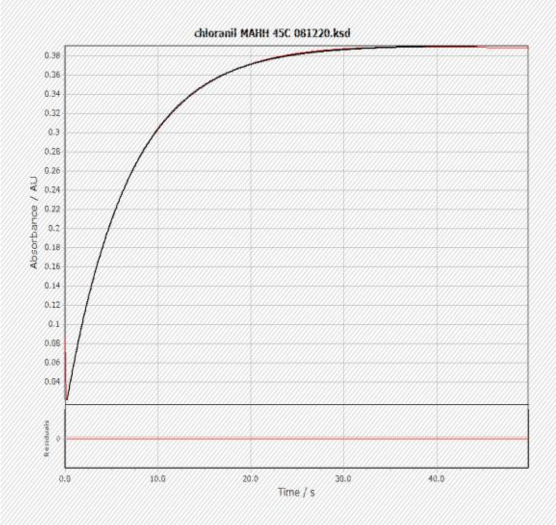

45°C H

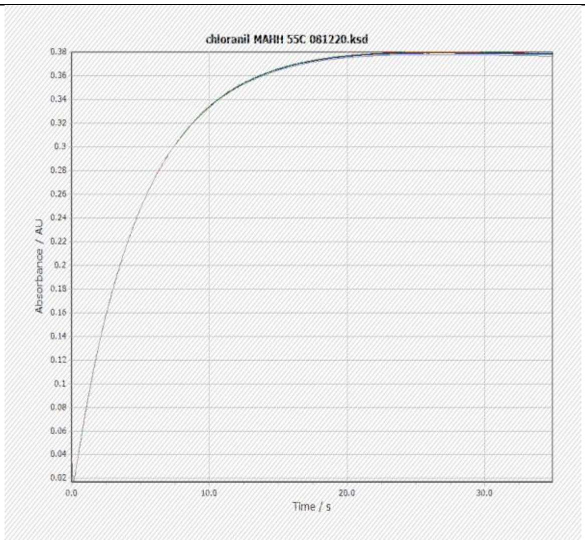

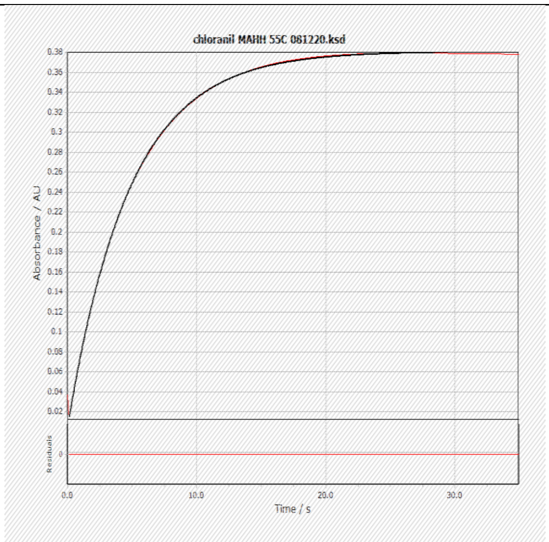

55°C H

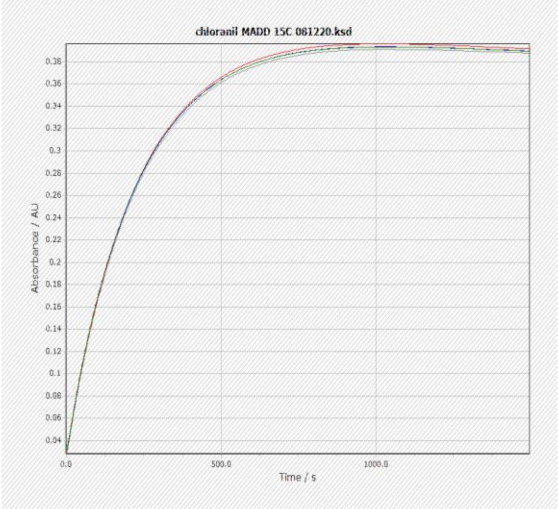

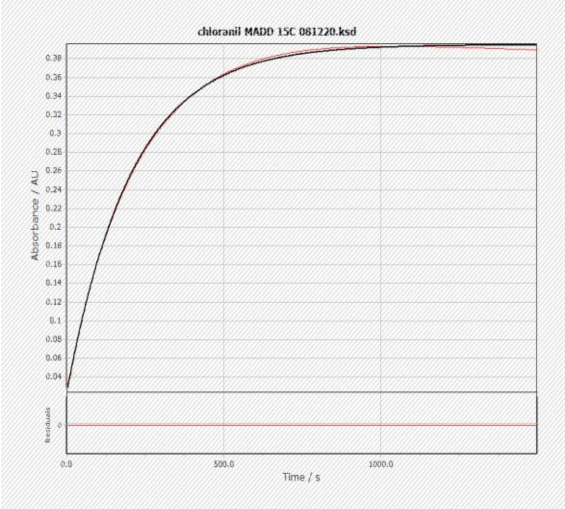

15°C D

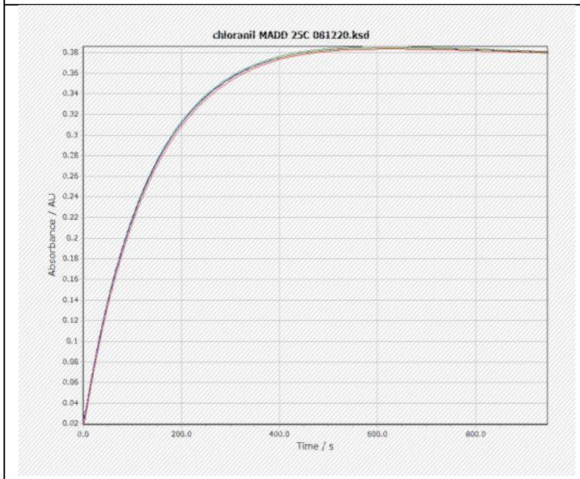

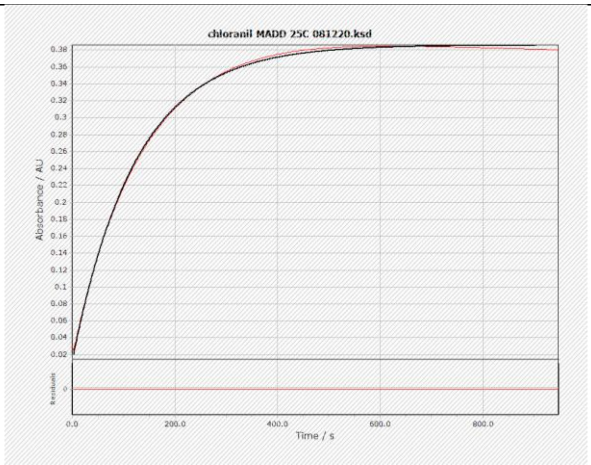

25°C D

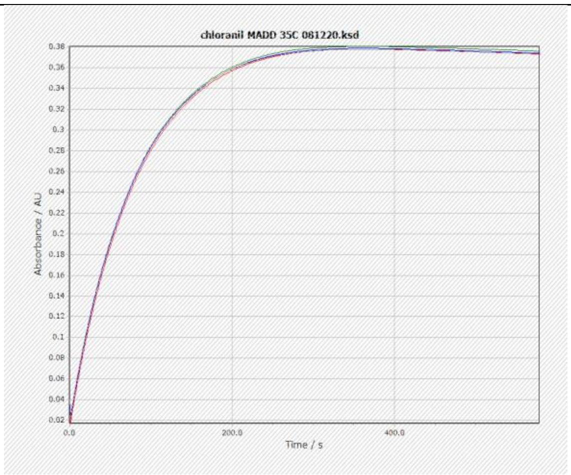

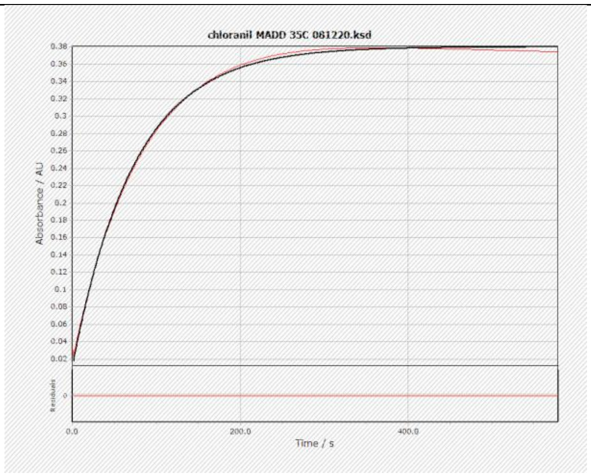

35°C D

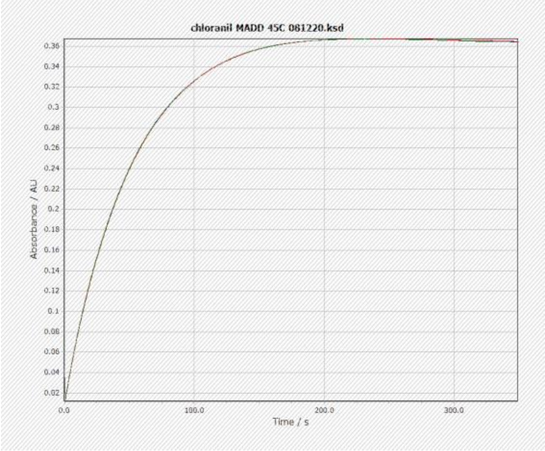

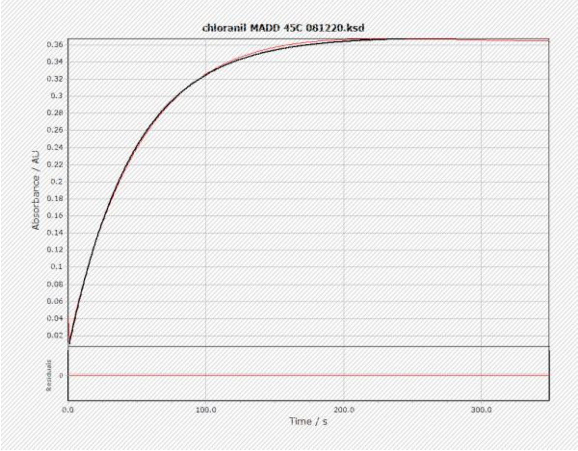

45°C D

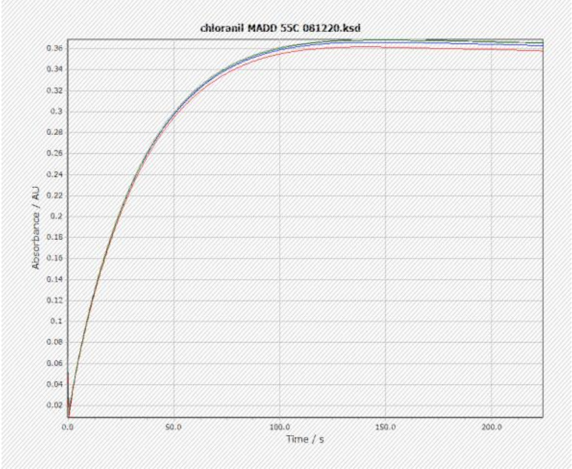

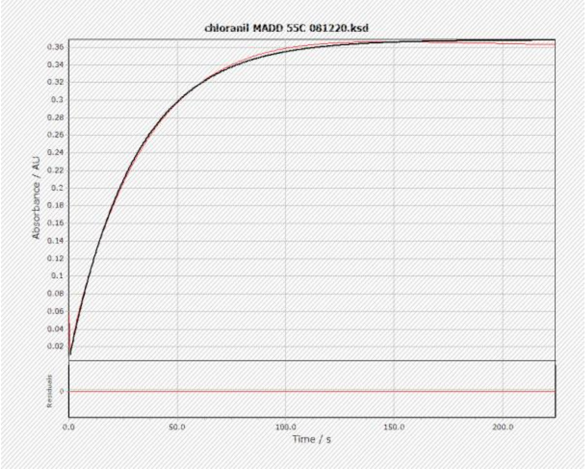

55°C D

Day 2 data (August 14, 2020)

Temp (°C)	Trial H1	Trial H2	Trial H3	Average k_H^{pfo} (s^{-1})	Stdev	k_{2H} [b] ($M^{-1}s^{-1}$)	Stdev [a]
	Pseudo-first-order rate constants k^{pfo} (s^{-1})						
55	0.2104	0.21037	0.21002	0.210263	0.000211266	5.97E+01	0.06002
45	0.14994	0.1485	0.14845	0.148963	0.000846188	4.23E+01	0.24039
35	0.09881	0.09954	0.09978	0.099377	0.000505206	2.82E+01	0.14352
25	0.06796	0.06765	0.06718	0.067597	0.000392726	1.92E+01	0.11157
15	0.044	0.04394	0.044	0.04398	3.4641E-05	1.25E+01	0.00984

Temp (°C)	Trial D1	Trial D2	Trial D3	Average k_D^{pfo} (s^{-1})	Stdev	k_{2D} [b] ($M^{-1}s^{-1}$)	Stdev [a]
55	0.03238	0.03305	0.03231	0.03258	0.000408534	9.26E+00	0.11606
45	0.02021	0.02069	0.02062	0.02051	0.000259294	5.83E+00	0.07366
35	0.01319	0.01327	0.01331	0.01326	6.1101E-05	3.77E+00	0.01736
25	0.00771	0.00798	0.00805	0.00791	0.000179536	2.25E+00	0.05100
15	0.00477	0.0048	0.00481	0.00479	2.08167E-05	1.36E+00	0.00591

[a] = (Stdev(for k^{pfo})/k^{pfo})*k_2; [b] = k^{pfo}/[MAH]

Three kinetic runs together with the averaged data	First-order kinetic fit of the averaged data as an example
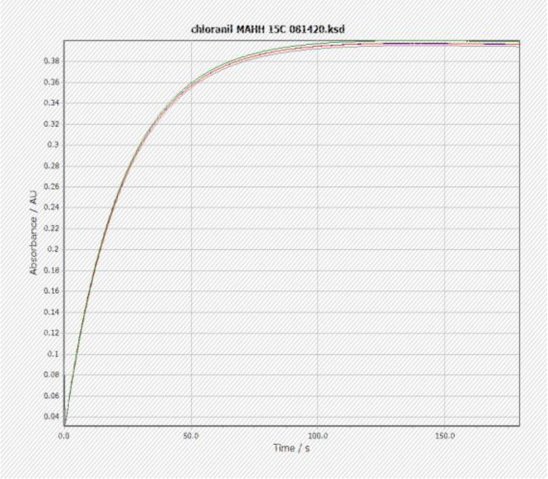	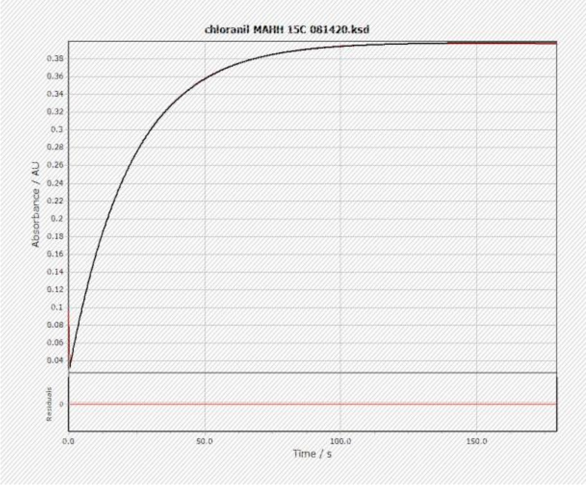
15°C H	

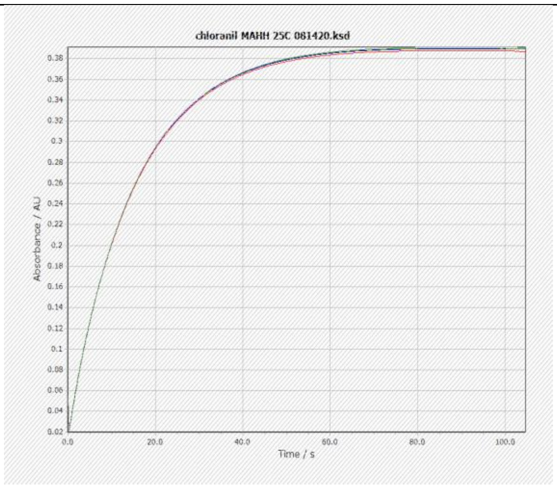

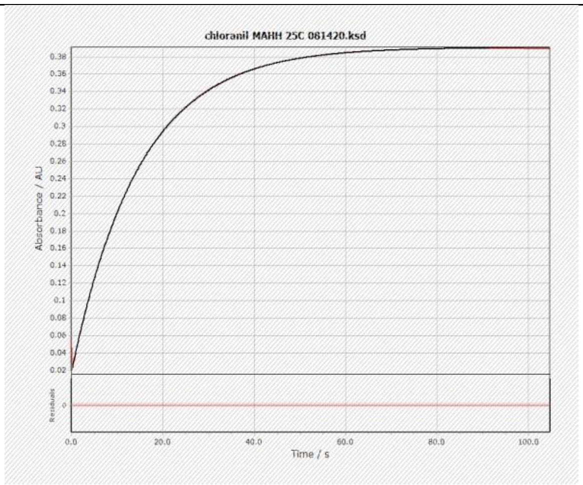

25°C H

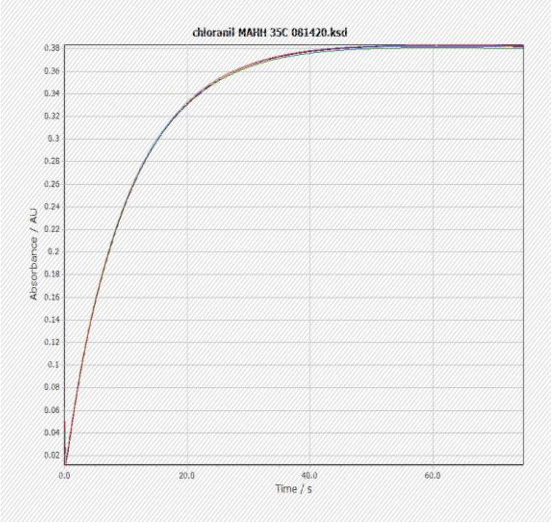

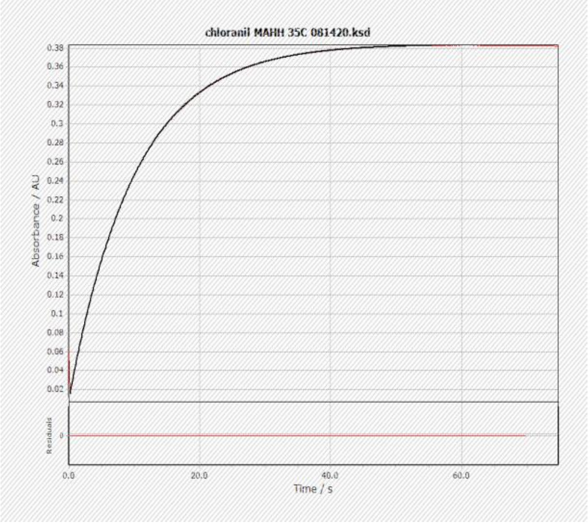

35°C H

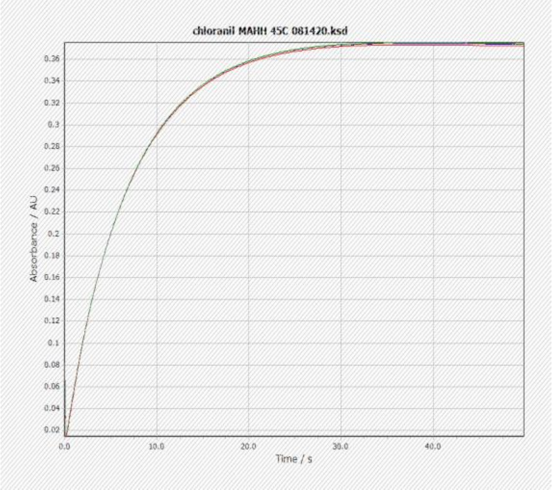

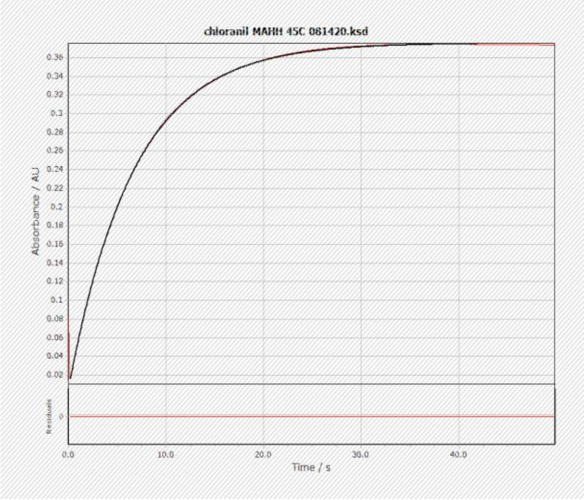

45°C H

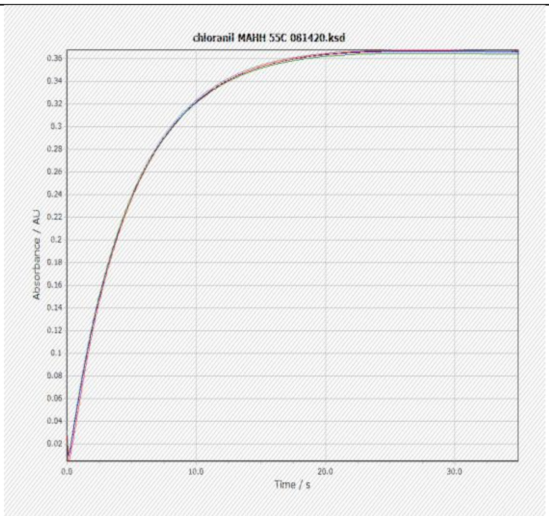

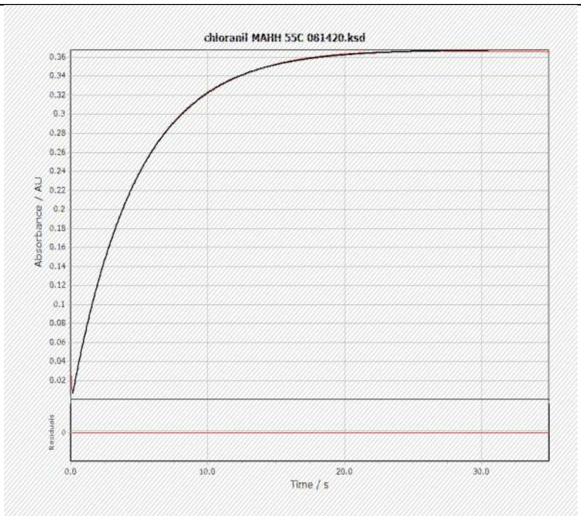

55°C H

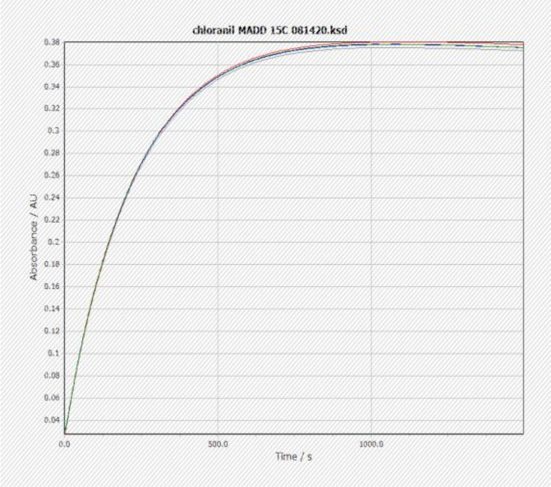

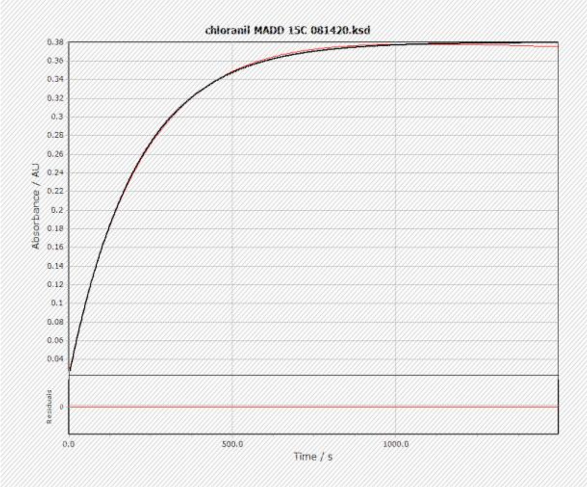

15°C D

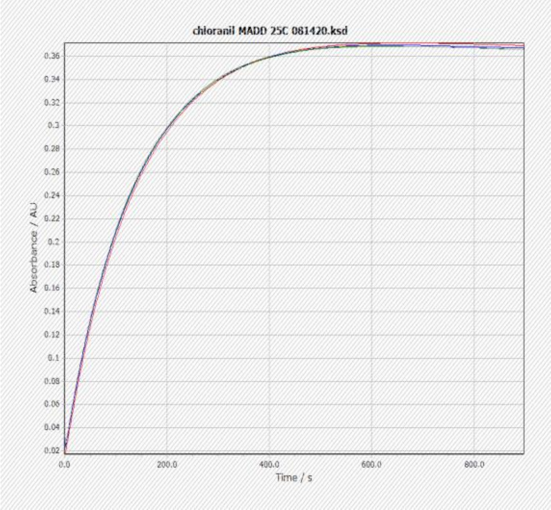

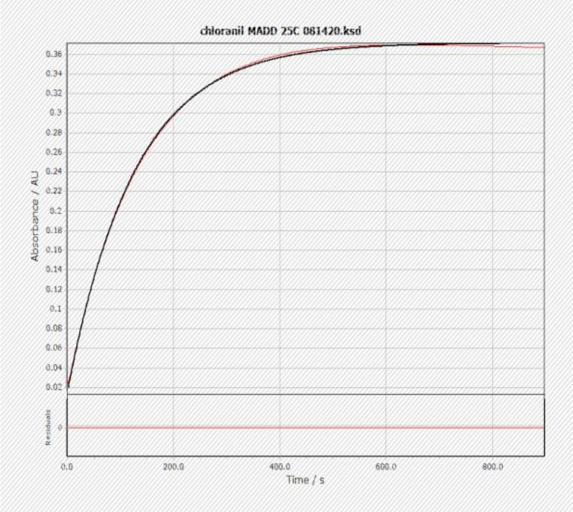

25°C D

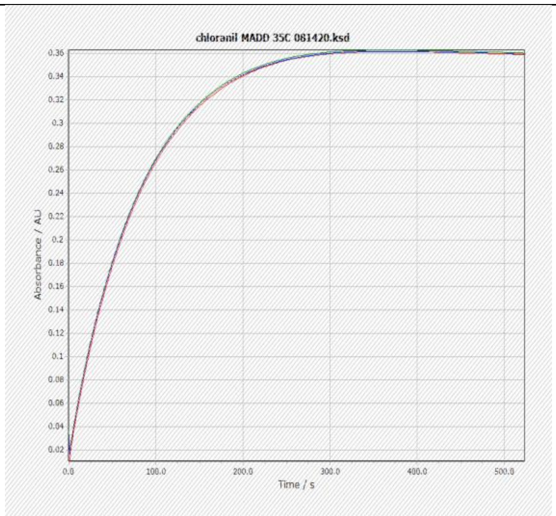

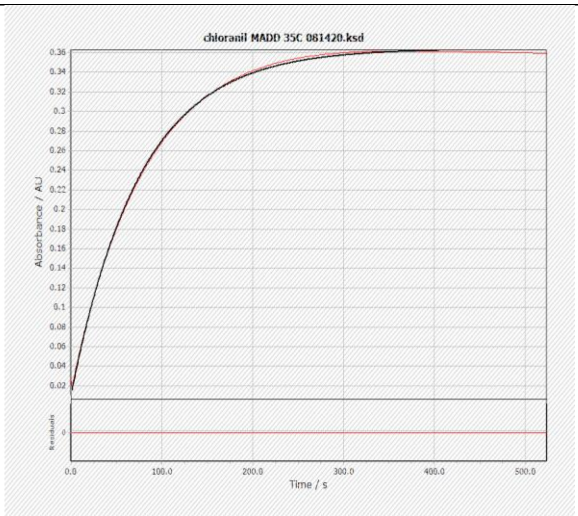

35°C D

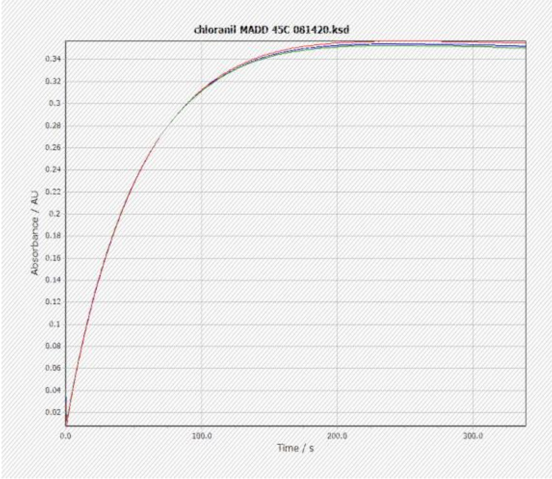

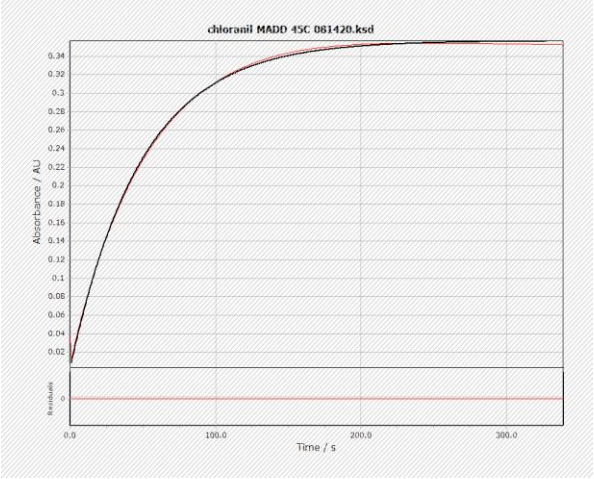

45°C D

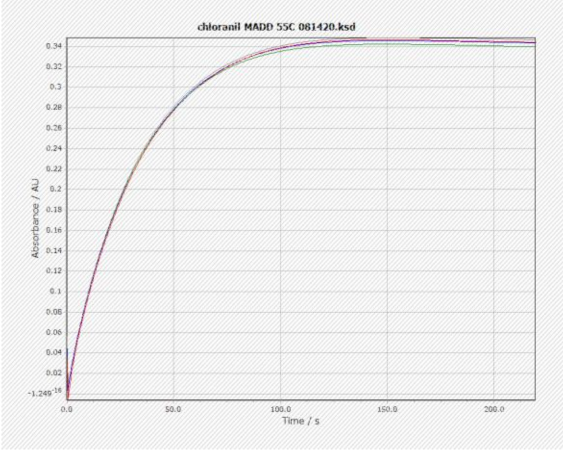

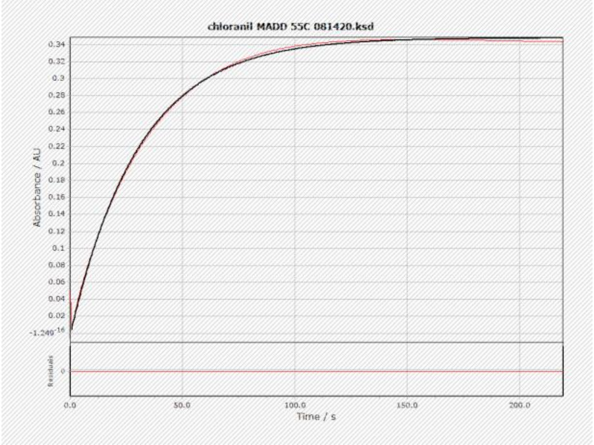

55°C D

Day 3 data (August 15, 2020)

Pseudo-first-order rate constants

k^{pfo} (s^{-1})

**Temp (°C)**	**Trial H1**	**Trial H2**	**Trial H3**	**Average k_H^{pfo} (s^{-1})**	**Stdev**	**k_{2H} [b] ($M^{-1}s^{-1}$)**	**Stdev [a]**
55	0.20503	0.20494	0.20425	0.20474	0.000426732	5.82E+01	0.12123
45	0.14832	0.14796	0.14503	0.147103	0.001804559	4.18E+01	0.51266
35	0.09962	0.09905	0.10052	0.09973	0.000741148	2.83E+01	0.21055
25	0.06765	0.06765	0.06714	0.06748	0.000294449	1.92E+01	0.08365
15	0.04344	0.04367	0.04354	0.04355	0.000115326	1.24E+01	0.03276

**Temp (°C)**	**Trial D1**	**Trial D2**	**Trial D3**	**Average k_D^{pfo} (s^{-1})**	**Stdev**	**k_{2D} [b] ($M^{-1}s^{-1}$)**	**Stdev [a]**
55	0.03381	0.03365	0.0333	0.03359	0.000260832	9.54E+00	0.07410
45	0.02028	0.02064	0.02057	0.02050	0.000190875	5.82E+00	0.05423
35	0.01345	0.01352	0.01323	0.01340	0.000151327	3.81E+00	0.04299
25	0.0077	0.00792	0.00801	0.00788	0.000159478	2.24E+00	0.04531
15	0.00486	0.00482	0.00479	0.00482	3.51188E-05	1.37E+00	0.00998

[a] = (Stdev(for k^{pfo})/k^{pfo})*k_2; [b] = k^{pfo}/[MAH]

Three kinetic runs together with the averaged data	First-order kinetic fit of the averaged data as an example
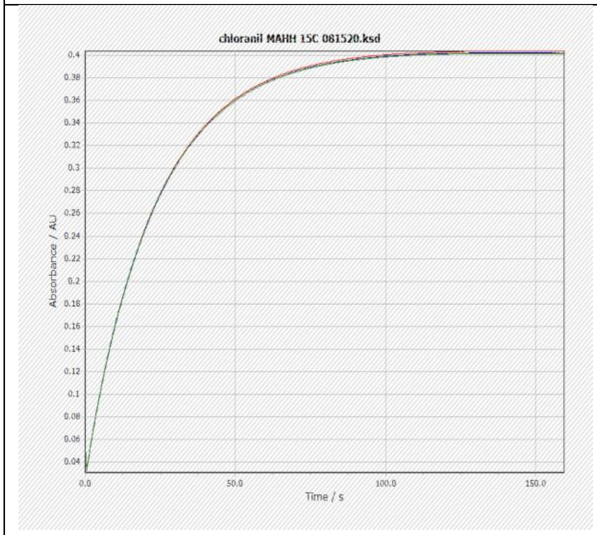	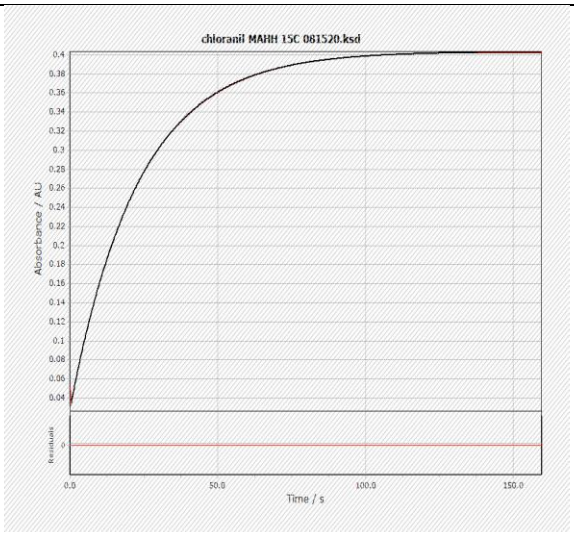
15°C H	

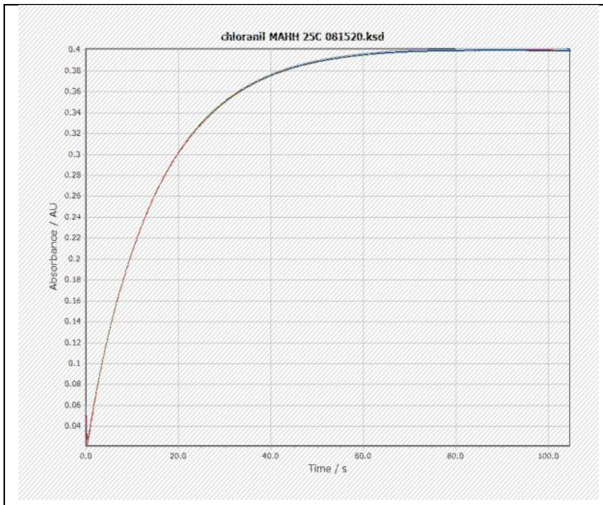
chloranil MAHH 25C 081520.ksd
Absorbance / AU
Time / s

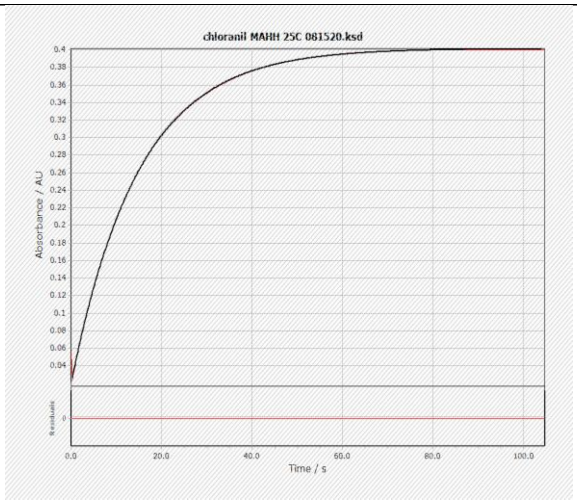
chloranil MAHH 25C 081520.ksd
Absorbance / AU
Residuals
Time / s

25℃ H

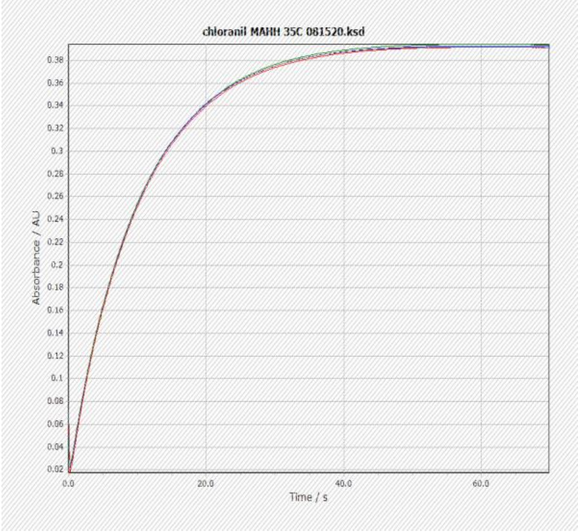
chloranil MAHH 35C 081520.ksd
Absorbance / AU
Time / s

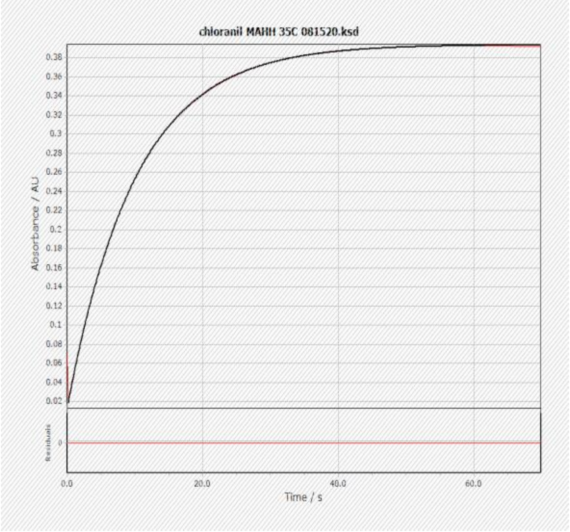
chloranil MAHH 35C 081520.ksd
Absorbance / AU
Residuals
Time / s

35℃ H

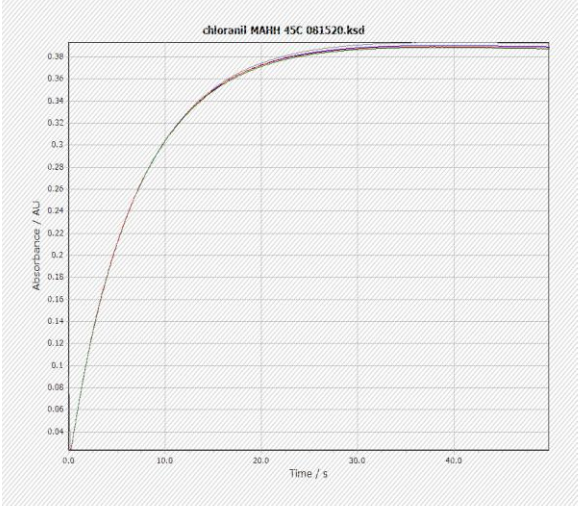
chloranil MAHH 45C 081520.ksd
Absorbance / AU
Time / s

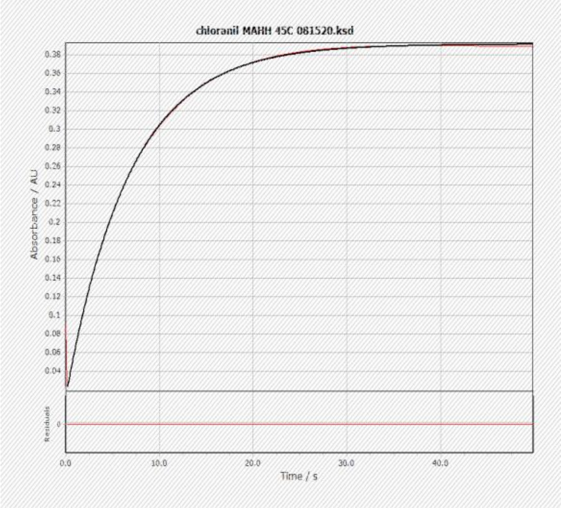
chloranil MAHH 45C 081520.ksd
Absorbance / AU
Residuals
Time / s

45℃ H

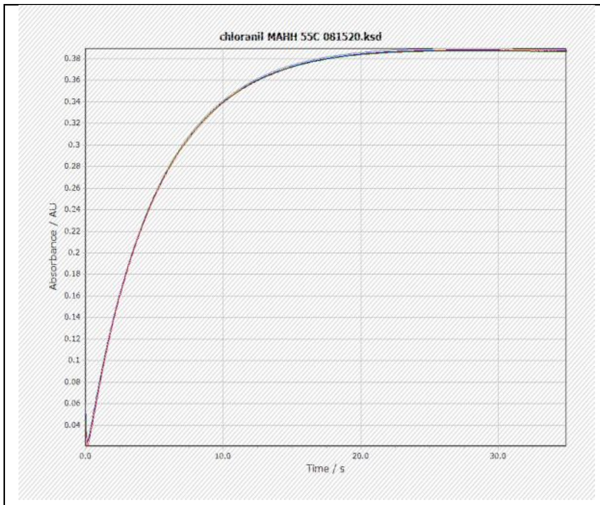
chloranil MAHH 55C 081520.ksd
Absorbance / AU
Time / s

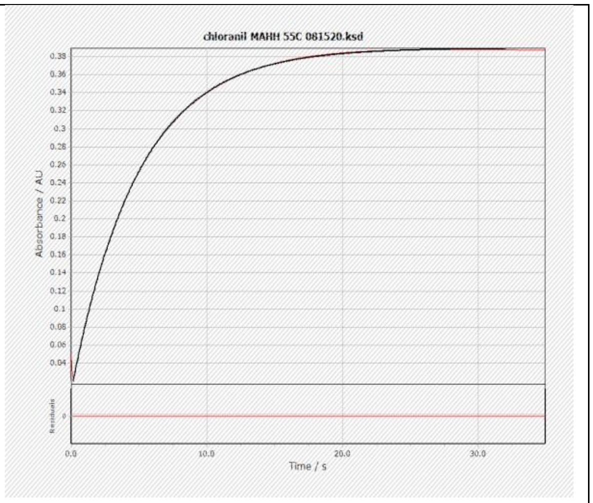
chloranil MAHH 55C 081520.ksd
Absorbance / AU
Residuals
Time / s

55℃ H

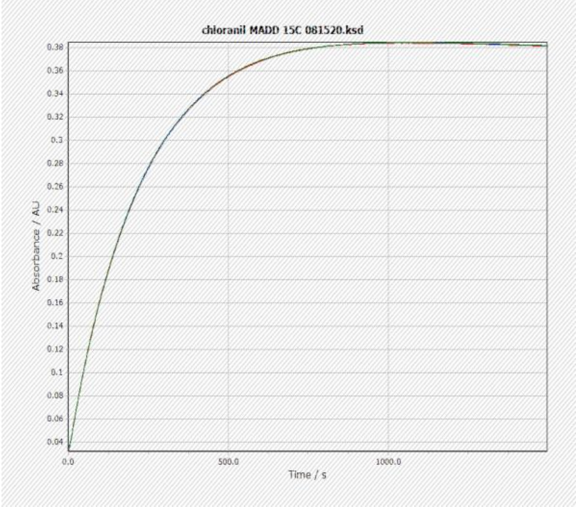
chloranil MADD 15C 081520.ksd
Absorbance / AU
Time / s

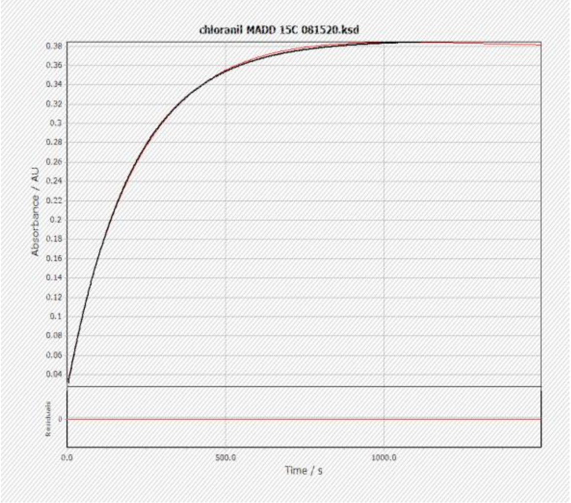
chloranil MADD 15C 081520.ksd
Absorbance / AU
Residuals
Time / s

15℃ D

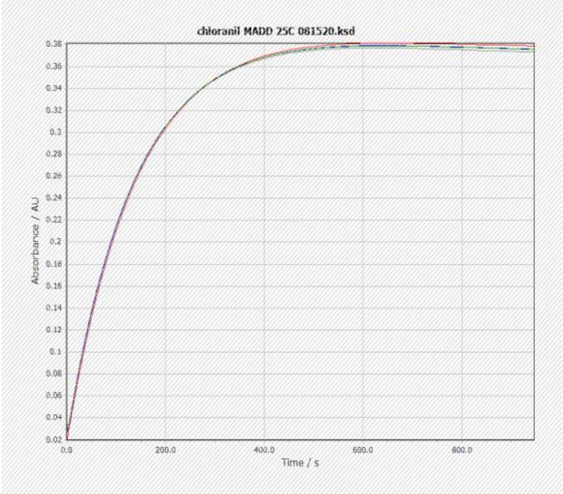
chloranil MADD 25C 081520.ksd
Absorbance / AU
Time / s

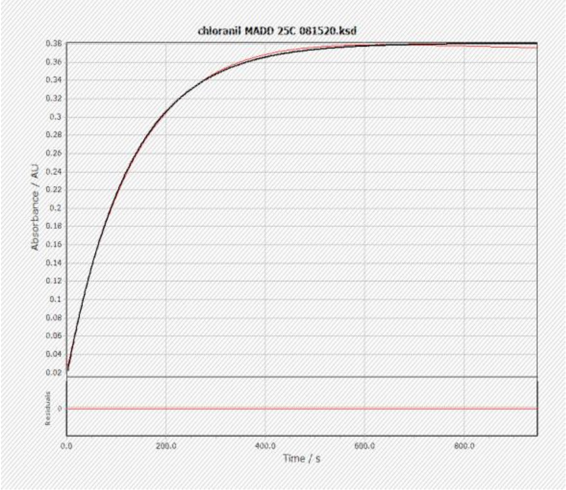
chloranil MADD 25C 081520.ksd
Absorbance / AU
Residuals
Time / s

25℃ D

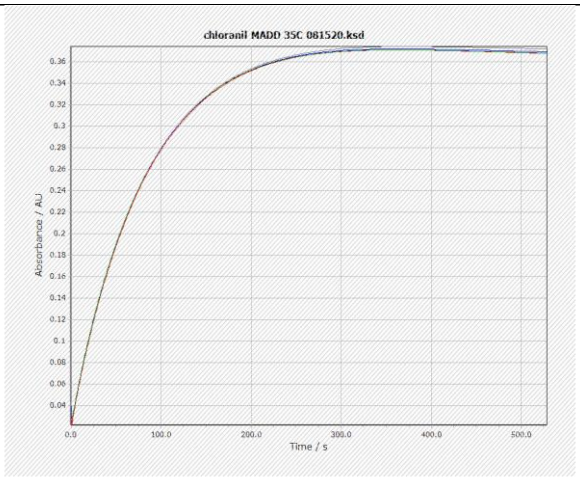

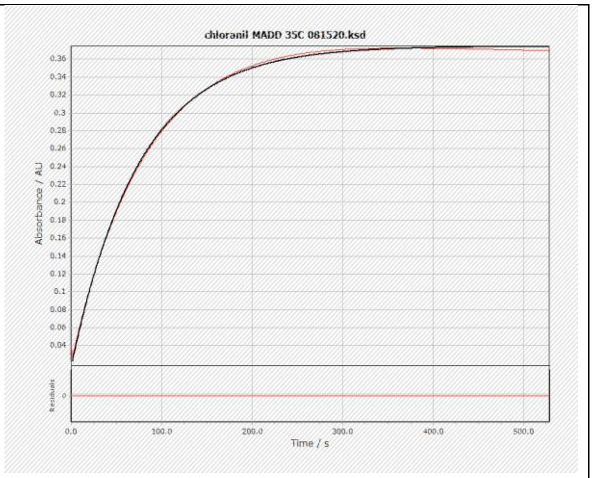

35°C D

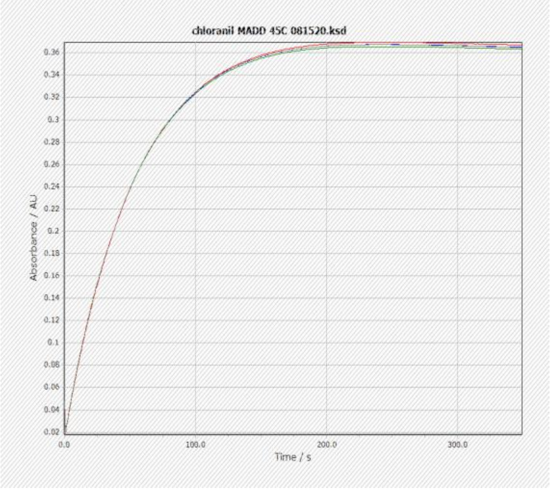

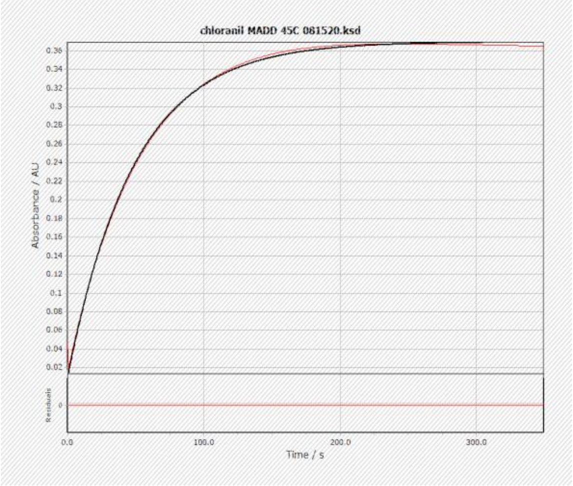

45°C D

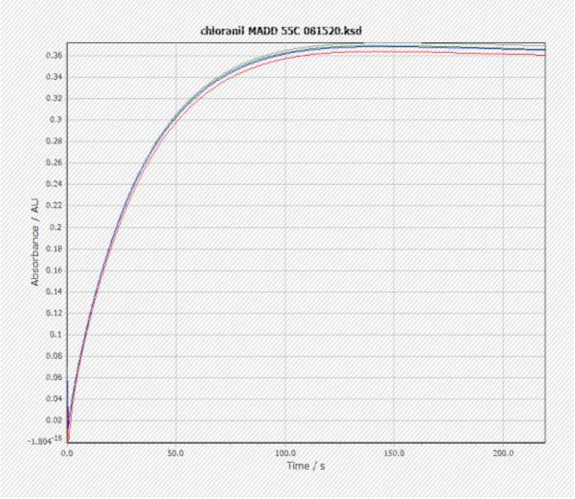

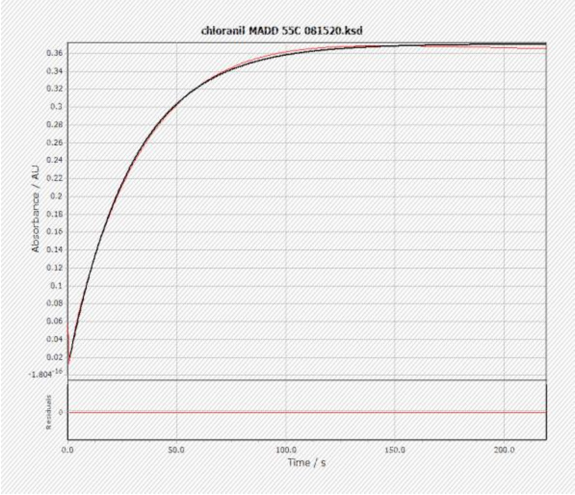

55°C D

Primary kinetic data for the rate constants in Table S4
Day 1 data (April 08, 2024)

Pseudo-first-order rate constants

k^{pfo} (s^{-1})

**Temp (°C)**	**Trial H1**	**Trial H2**	**Trial H3**	**Average k_H^{pfo} (s^{-1})**	**Stdev**	**k_{2H} [b] (M^{-1}s^{-1})**	**Stdev [a]**
55	0.55091	0.5391	0.53809	0.5427	0.00712798	1.54E+02	2.02499
45	0.43034	0.43237	0.43402	0.432243	0.001843267	1.23E+02	0.52366
35	0.32823	0.32456	0.32549	0.326093	0.00190794	9.26E+01	0.54203
25	0.2416	0.24252	0.23869	0.240937	0.001999308	6.84E+01	0.56799
15	0.18931	0.18505	0.18111	0.185157	0.004101041	5.26E+01	1.16507

**Temp (°C)**	**Trial D1**	**Trial D2**	**Trial D3**	**Average k_D^{pfo} (s^{-1})**	**Stdev**	**k_{2D} [b] (M^{-1}s^{-1})**	**Stdev [a]**
55	0.08454	0.08406	0.08254	0.08371	0.001044095	2.38E+01	0.29662
45	0.06154	0.0593000	0.05936	0.06007	0.001276297	1.71E+01	0.36258
35	0.04216	0.042	0.04215	0.04210	8.96289E-05	1.20E+01	0.02546
25	0.02976	0.02897	0.02909	0.02927	0.000425715	8.32E+00	0.12094
15	0.02	0.0203	0.01996	0.02009	0.000185831	5.71E+00	0.05279

[a] = (Stdev(for k^{pfo})/k^{pfo})*k_2; [b] = k^{pfo}/[PAH]

Three kinetic runs together with the averaged data	First-order kinetic fit of the averaged data as an example
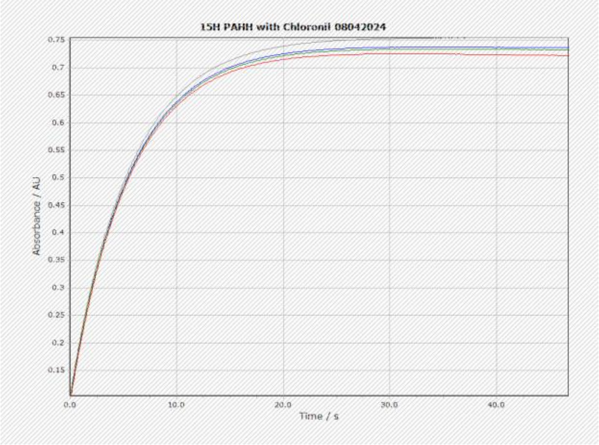	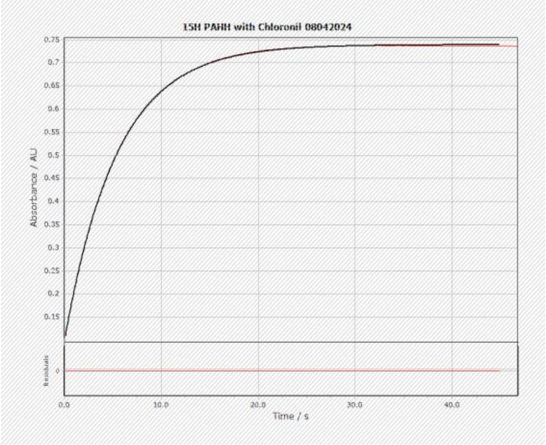
15°C H	

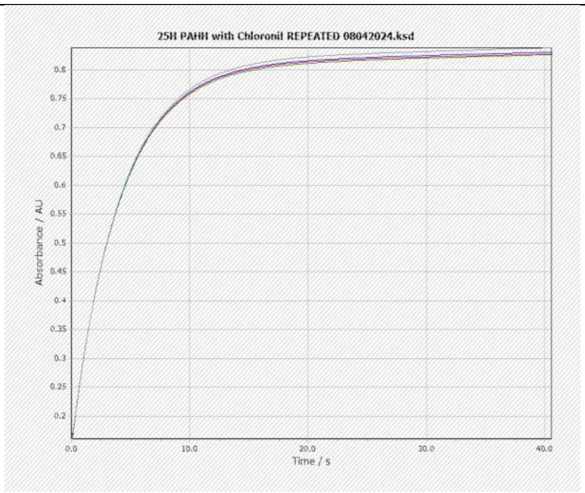

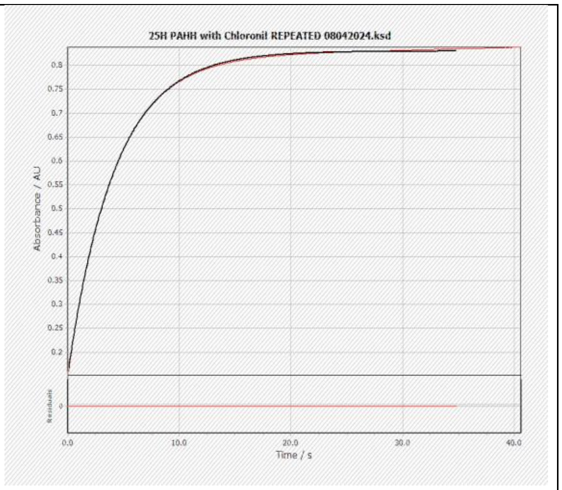

25°C H

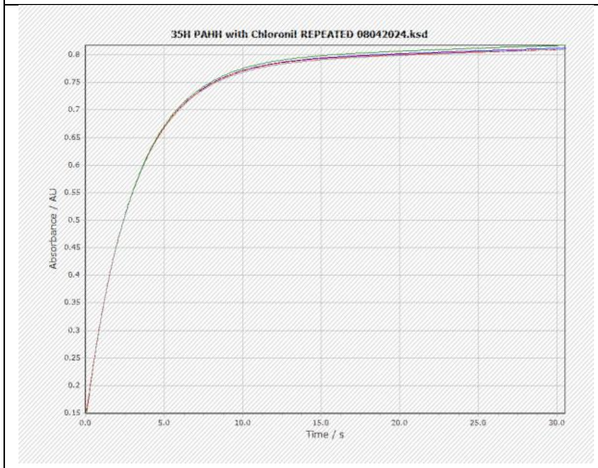

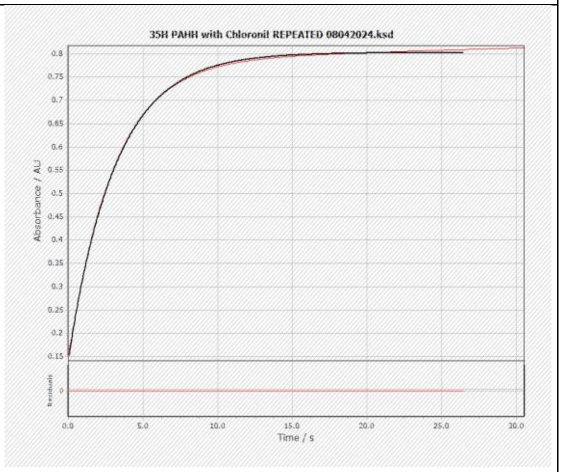

35°C H

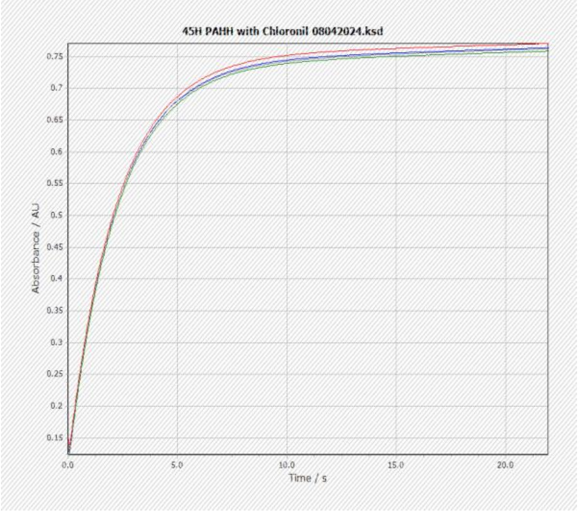

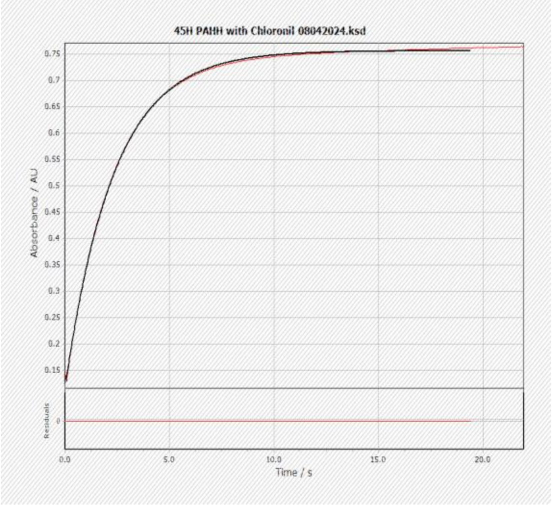

45°C H

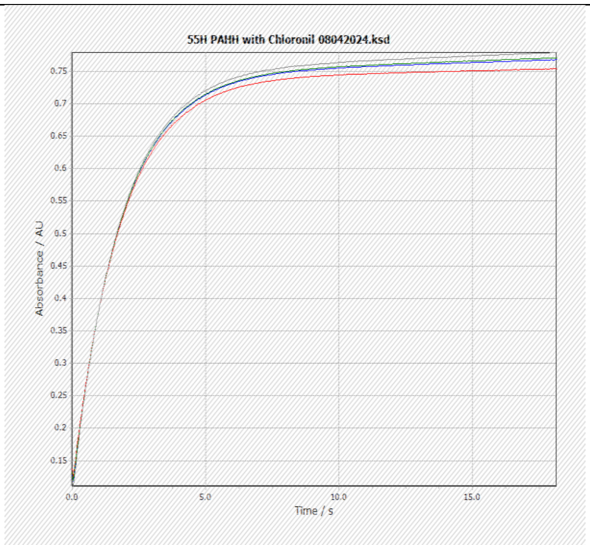

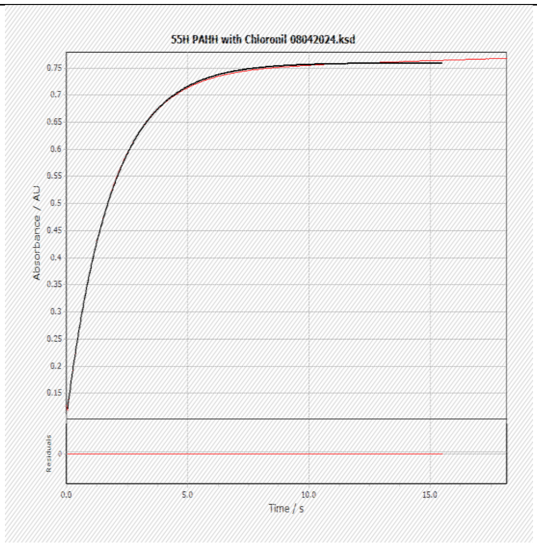

55°C H

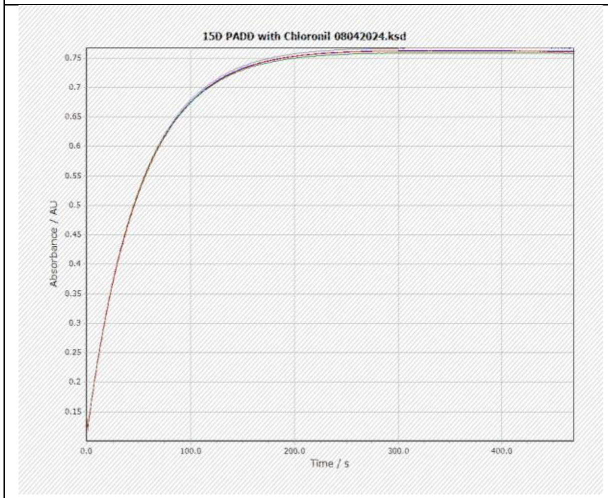

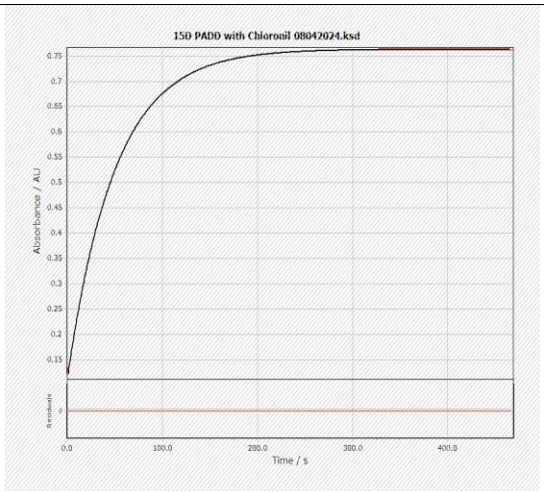

15°C D

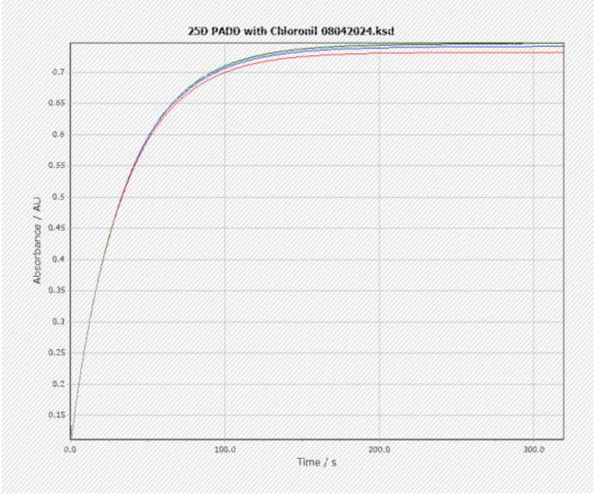

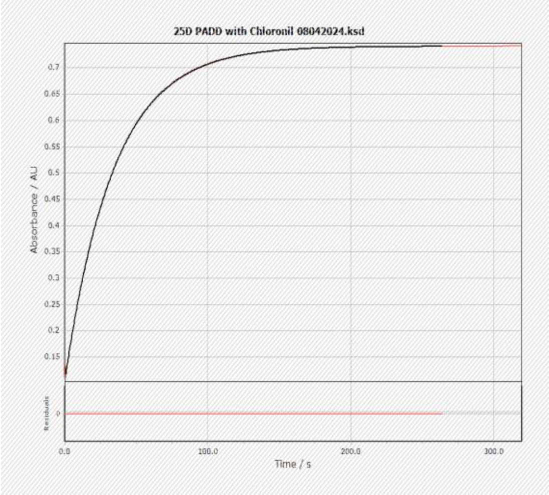

25°C D

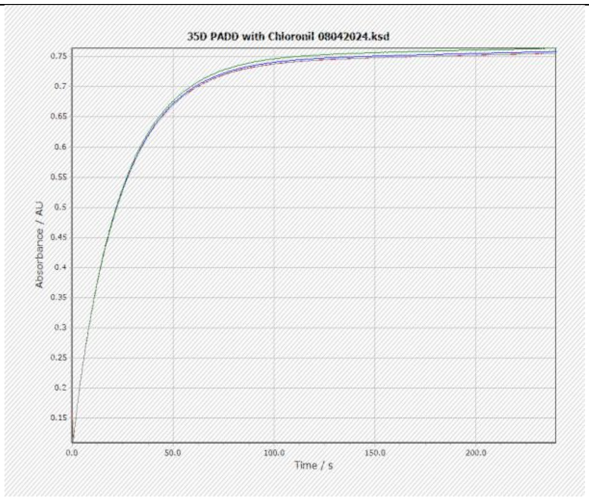
35D PADD with Chloronil 08042024.ksd
Absorbance / AU
Time / s

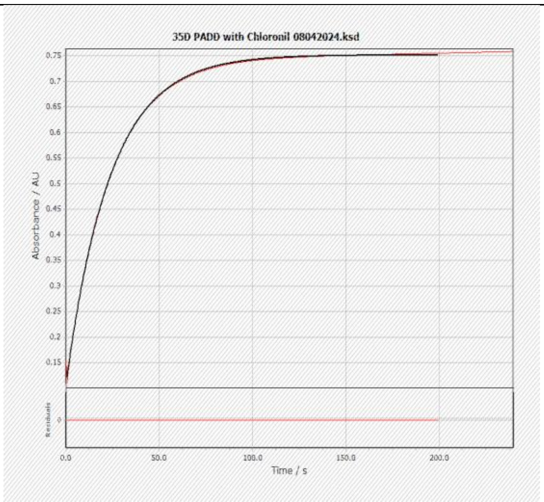
35D PADD with Chloronil 08042024.ksd
Absorbance / AU
Residuals
Time / s

35℃ D

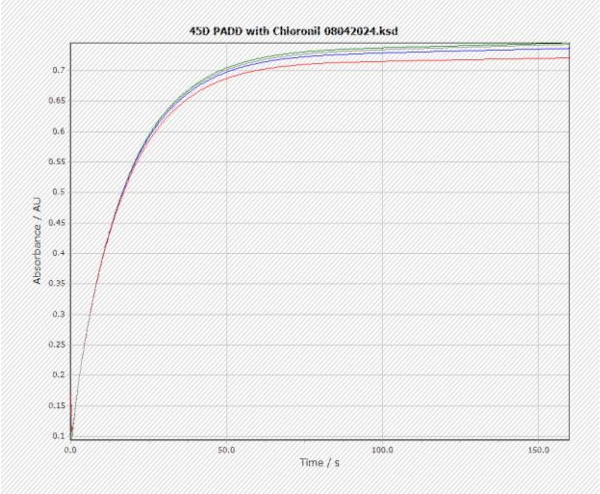
45D PADD with Chloronil 08042024.ksd
Absorbance / AU
Time / s

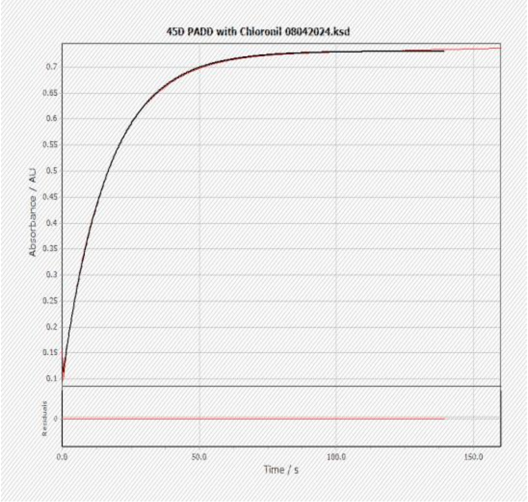
45D PADD with Chloronil 08042024.ksd
Absorbance / AU
Residuals
Time / s

45℃ D

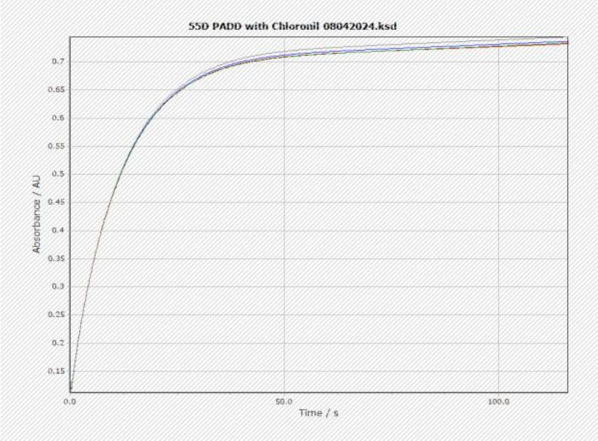
55D PADD with Chloronil 08042024.ksd
Absorbance / AU
Time / s

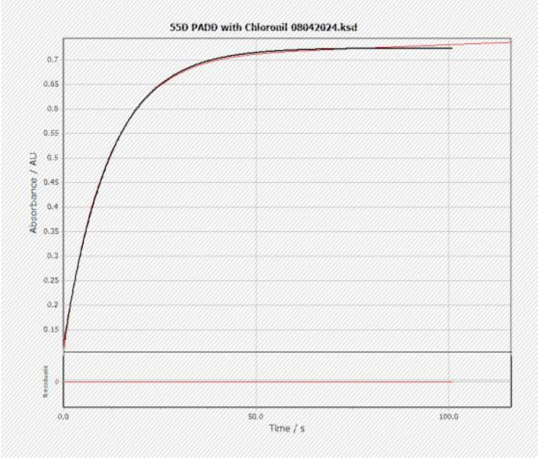
55D PADD with Chloronil 08042024.ksd
Absorbance / AU
Residuals
Time / s

55℃ D

Day 2 data (April 10, 2024)

Pseudo-first-order rate constants

k^{pfo} (s^{-1})

Temp (°C)	Trial H1	Trial H2	Trial H3	Average k_H^{pfo} (s^{-1})	Stdev	k_{2H} [b] (M^{-1}s^{-1})	Stdev [a]
55	0.53289	0.53195	0.54056	0.535133	0.004723075	1.52E+02	1.34178
45	0.42963	0.43256	0.42888	0.430357	0.001944642	1.22E+02	0.55246
35	0.32339	0.31998	0.32582	0.323063	0.002933672	9.18E+01	0.83343
25	0.2419	0.2388	0.23933	0.24001	0.001658101	6.82E+01	0.47105
15	0.17169	0.16977	0.16932	0.17026	0.00125869	4.84E+01	0.35758

Temp (°C)	Trial D1	Trial D2	Trial D3	Average k_D^{pfo} (s^{-1})	Stdev	k_{2D} [b] (M^{-1}s^{-1})	Stdev [a]
55	0.09059	0.08835	0.08591	0.08828	0.002340712	2.51E+01	0.66498
45	0.06189	0.06173	0.06205	0.06189	0.00016	1.76E+01	0.04545
35	0.04393	0.04469	0.04389	0.04417	0.000450777	1.25E+01	0.12806
25	0.02909	0.02886	0.02813	0.02869	0.000501232	8.15E+00	0.14240
15	0.01955	0.01989	0.01969	0.01971	0.00017088	5.60E+00	0.04855

[a] = (Stdev(for k^{pfo})/k^{pfo})*k_2; [b] = k^{pfo}/[PAH]

Three kinetic runs together with the averaged data	First-order kinetic fit of the averaged data as an example
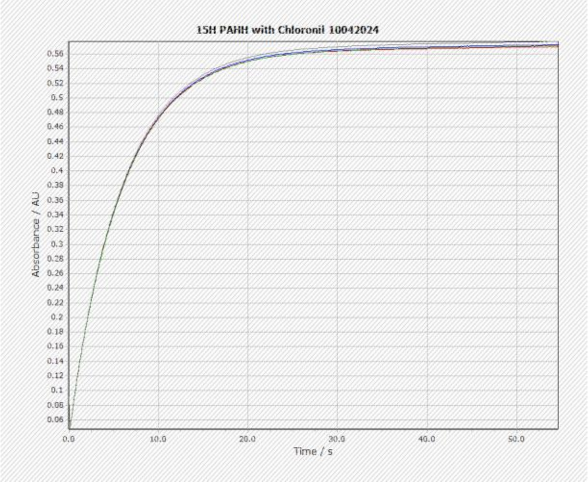	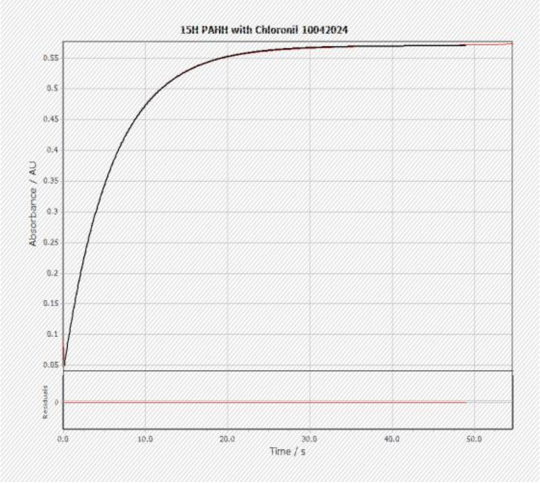
15°C H	

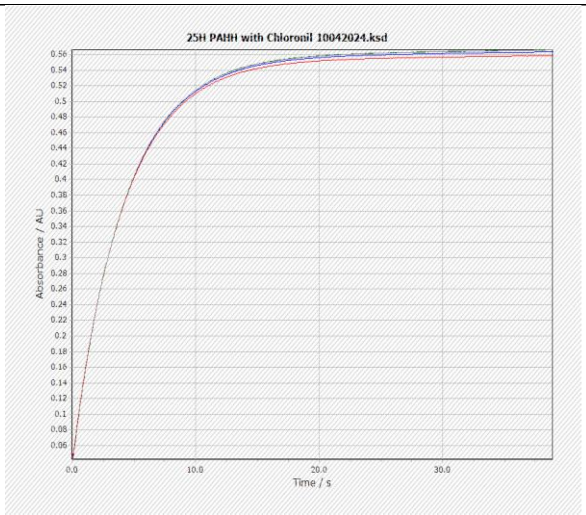

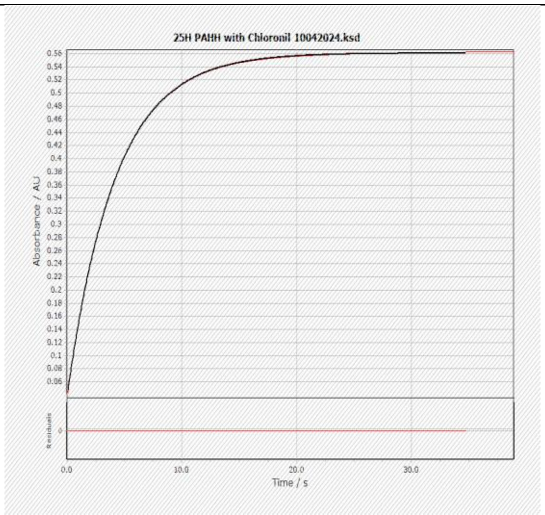

25°C H

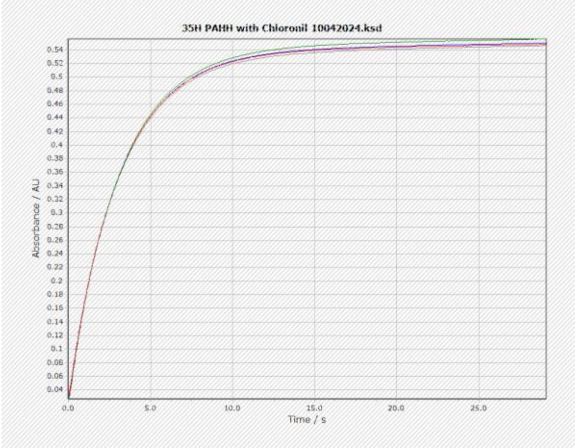

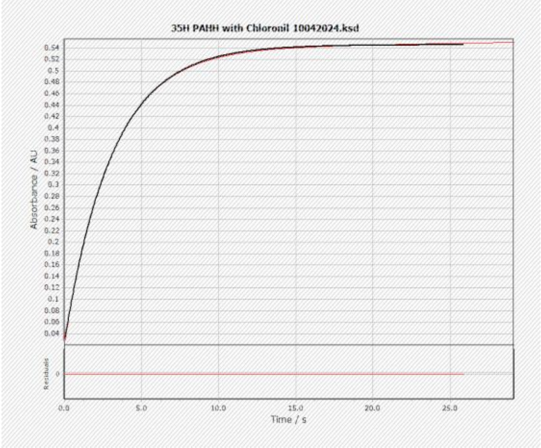

35°C H

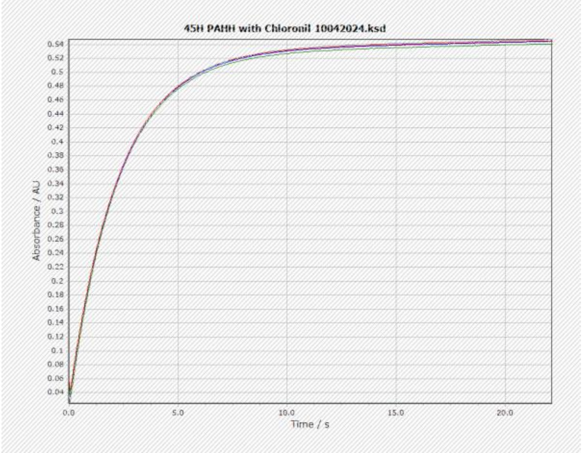

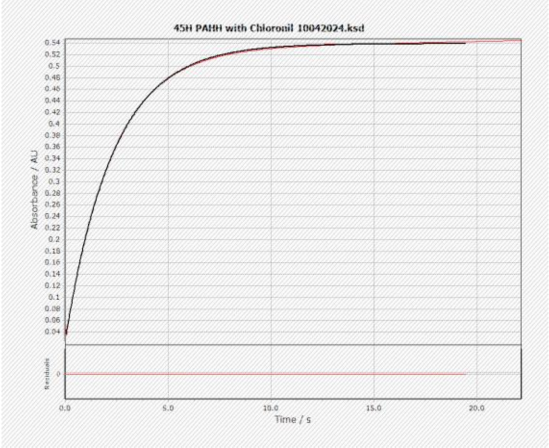

45°C H

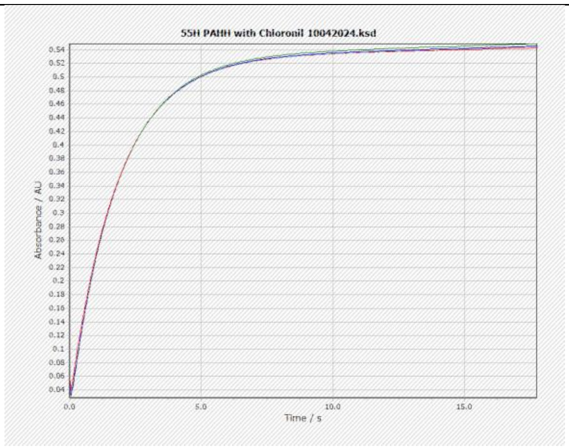

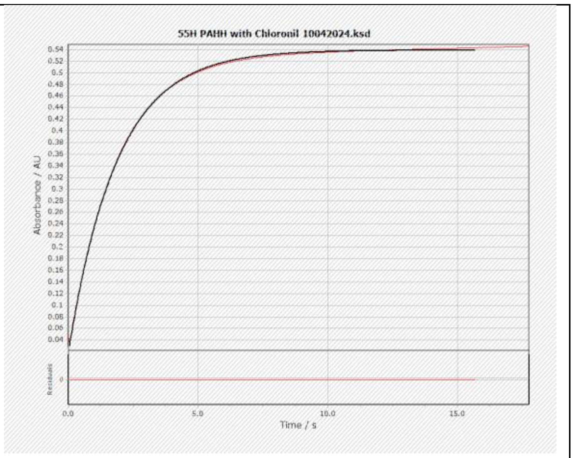

55°C H

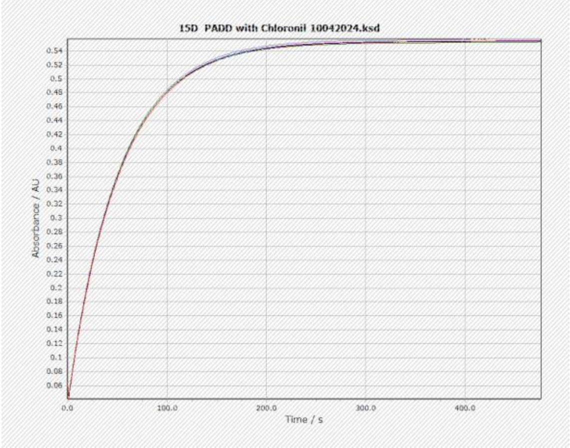

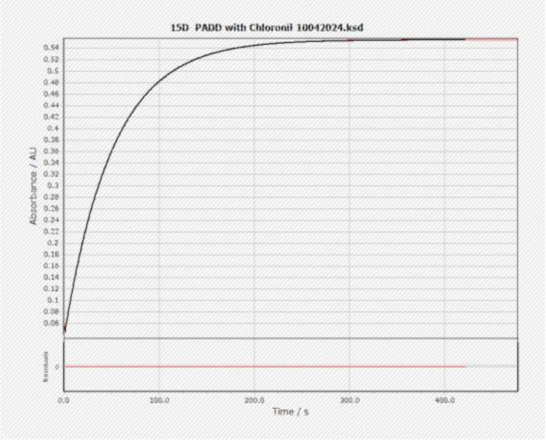

15°C D

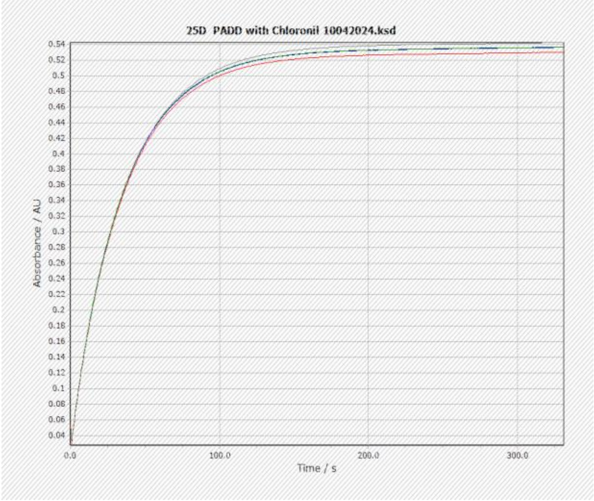

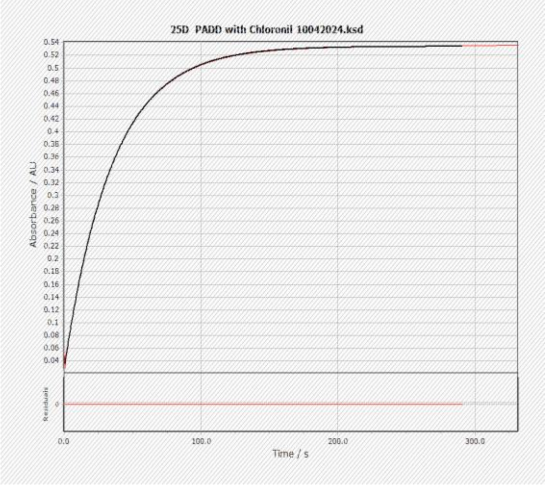

25°C D

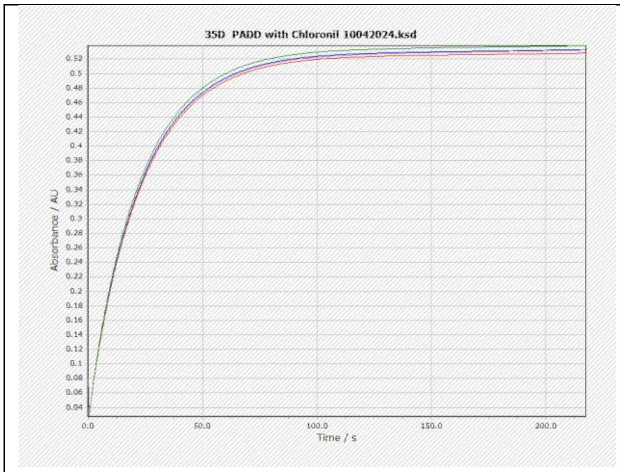

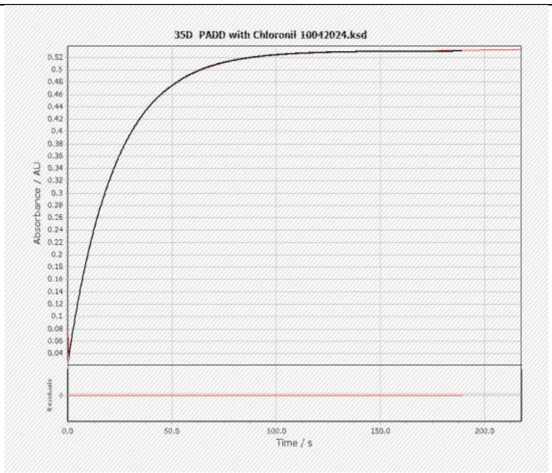

35°C D

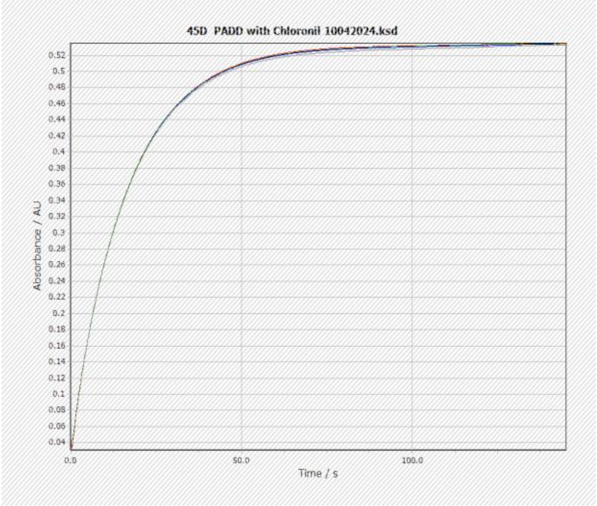

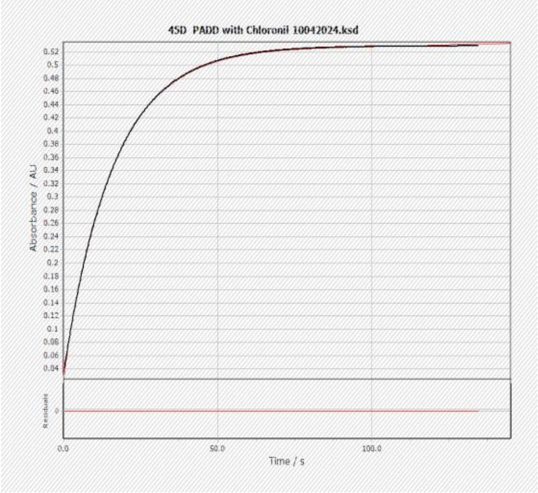

45°C D

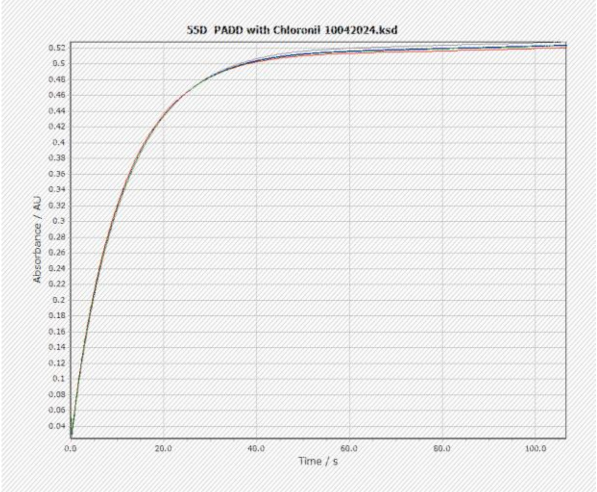

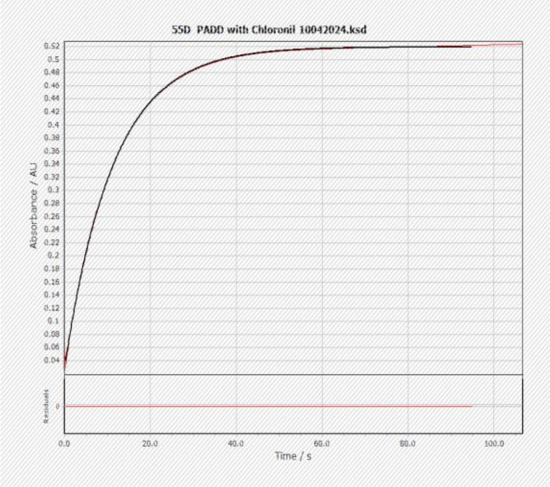

55°C D

Day 3 data (April 19, 2024)

Pseudo-first-order rate constants

k^{pfo} (s^{-1})

Temp (°C)	Trial H1	Trial H2	Trial H3	Average k_H^{pfo} (s^{-1})	Stdev	k_{2H} [b] (M^{-1}s^{-1})	Stdev [a]
55	0.53846	0.53825	0.53063	0.53578	0.004461267	1.52E+02	1.26741
45	0.4223	0.41107	0.42011	0.417827	0.005953019	1.19E+02	1.69120
35	0.32111	0.31803	0.31284	0.317327	0.004179621	9.01E+01	1.18739
25	0.23317	0.23509	0.23514	0.234467	0.001123225	6.66E+01	0.31910
15	0.17548	0.17388	0.17256	0.173973	0.001462236	4.94E+01	0.41541

Temp (°C)	Trial D1	Trial D2	Trial D3	Average k_D^{pfo} (s^{-1})	Stdev	k_{2D} [b] (M^{-1}s^{-1})	Stdev [a]
55	0.0874	0.08643	0.08514	0.08632	0.00113377	2.45E+01	0.32209
45	0.06188	0.06004	0.05856	0.06016	0.00166325	1.71E+01	0.47251
35	0.04298	0.04347	0.043	0.04315	0.000277308	1.23E+01	0.07878
25	0.02827	0.02833	0.02820	0.02827	6.50641E-05	8.03E+00	0.01848
15	0.01938	0.0195	0.01959	0.01949	0.000105357	5.54E+00	0.02993

[a] = (Stdev(for k^{pfo})/k^{pfo})*k_2; [b] = k^{pfo}/[PAH]

Three kinetic runs together with the averaged data

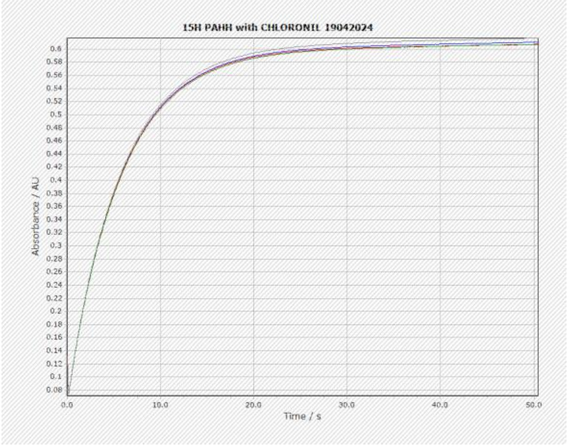

First-order kinetic fit of the averaged data as an example

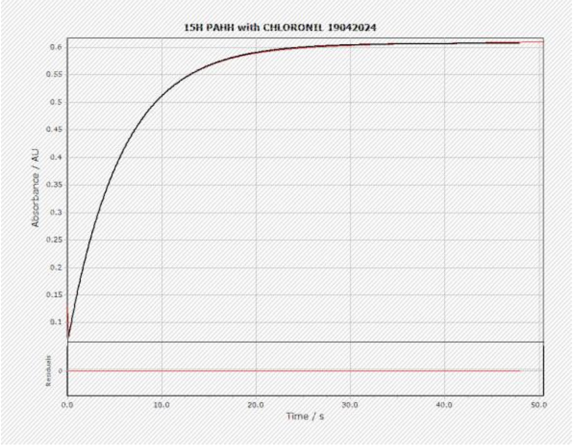

15°C H

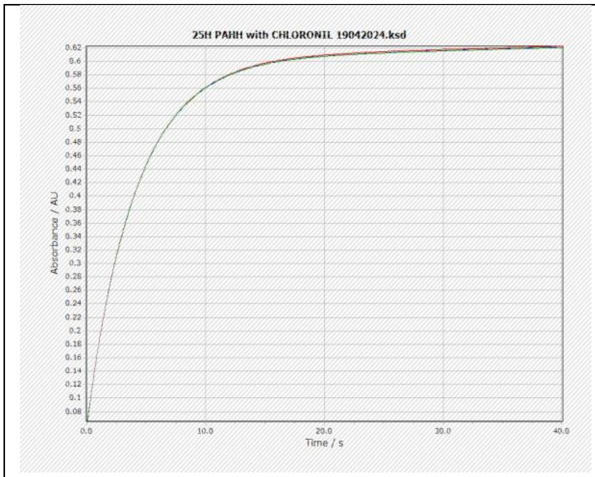

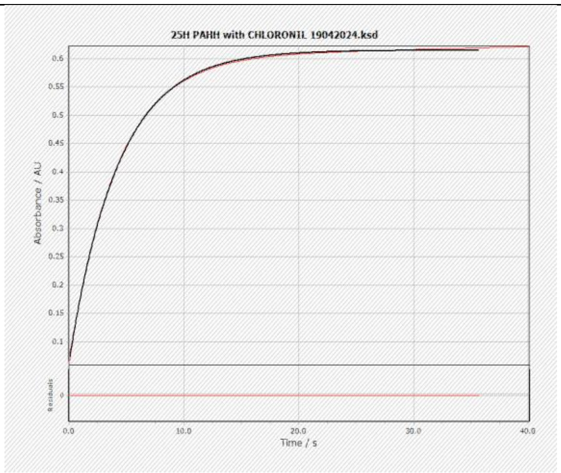

25°C H

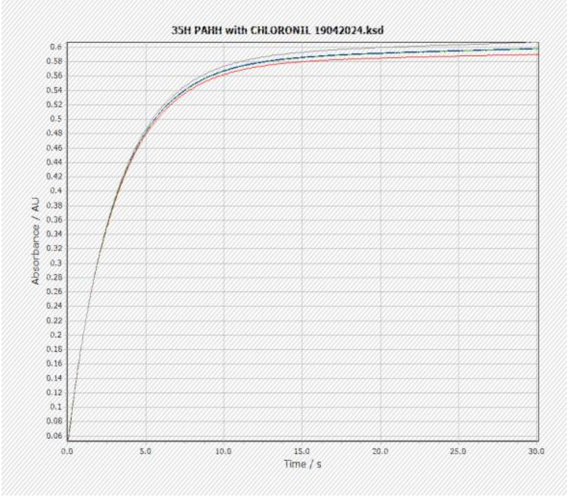

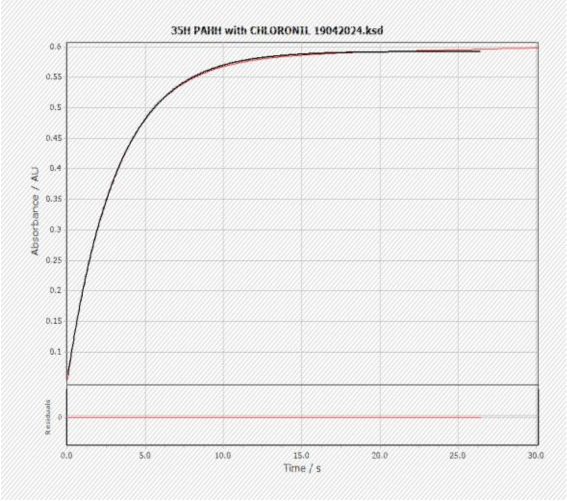

35°C H

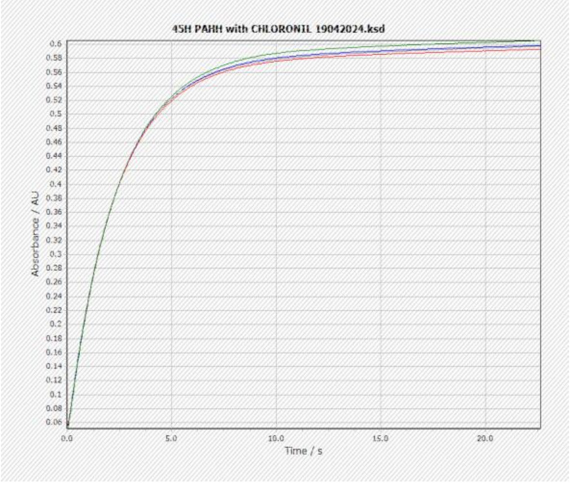

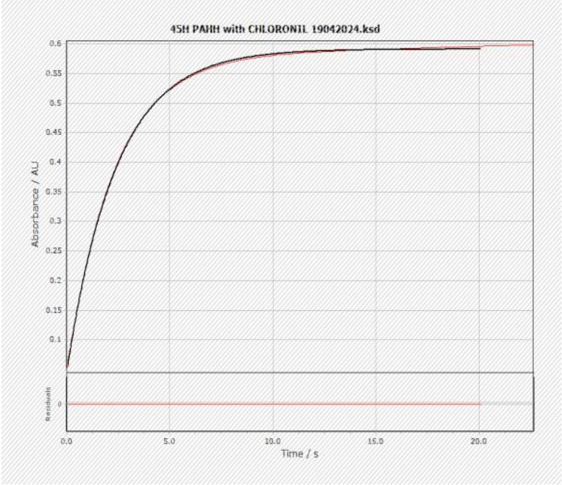

45°C H

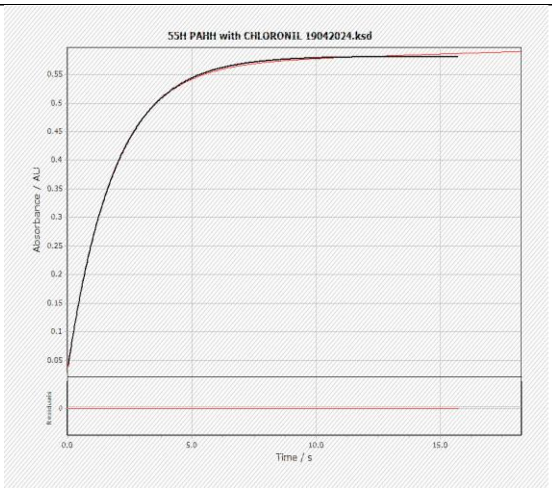

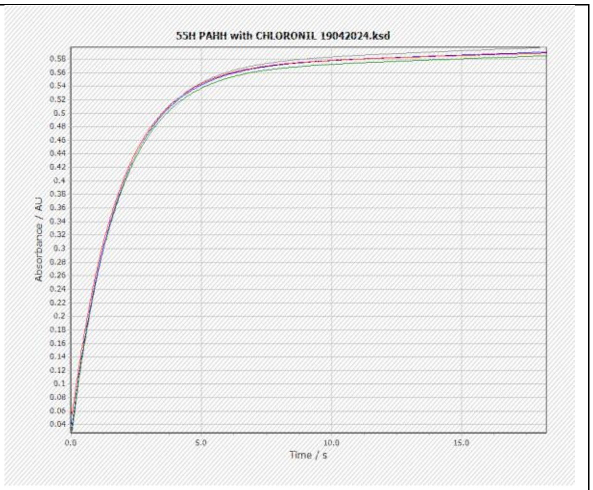

55°C H

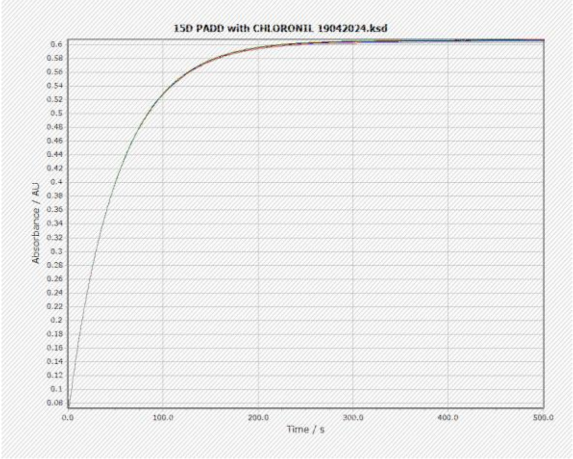

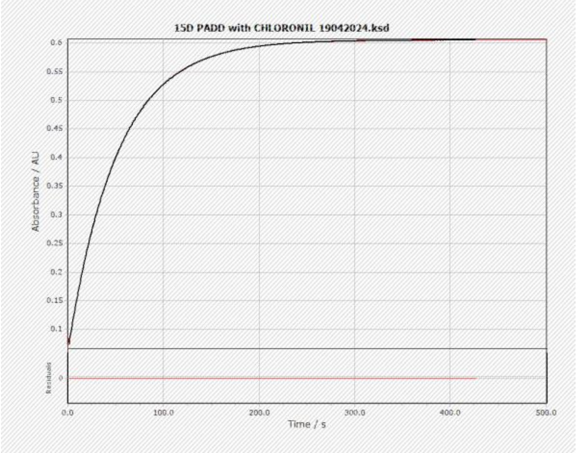

15°C D

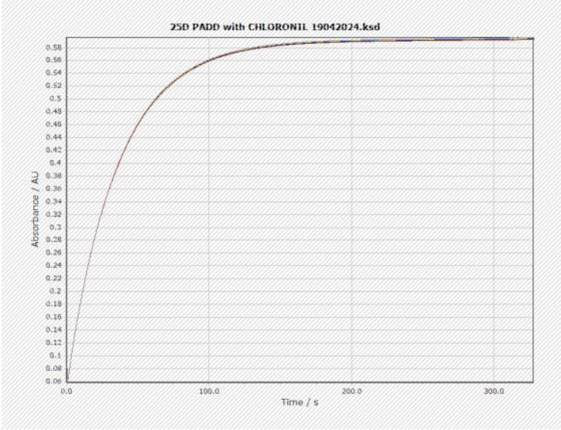

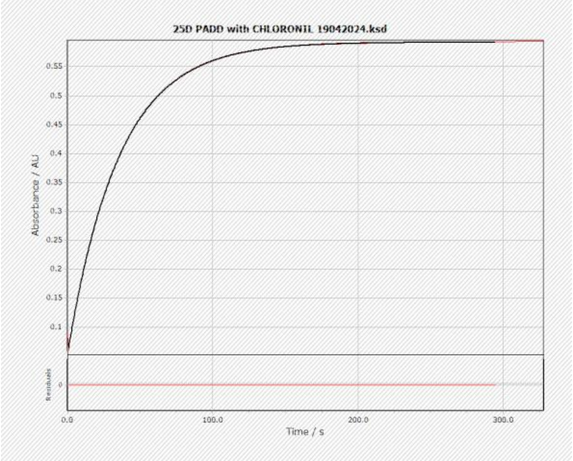

25°C D

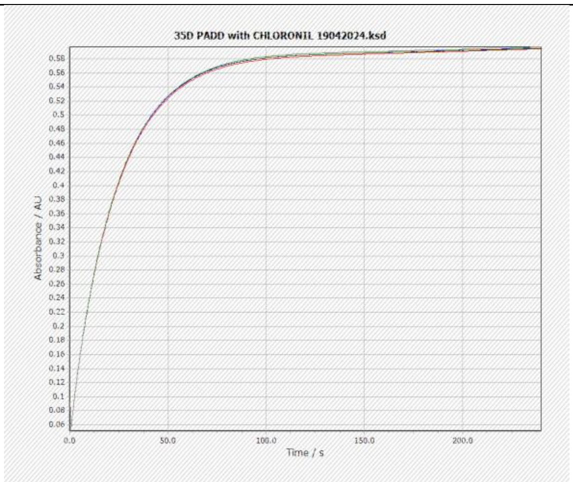

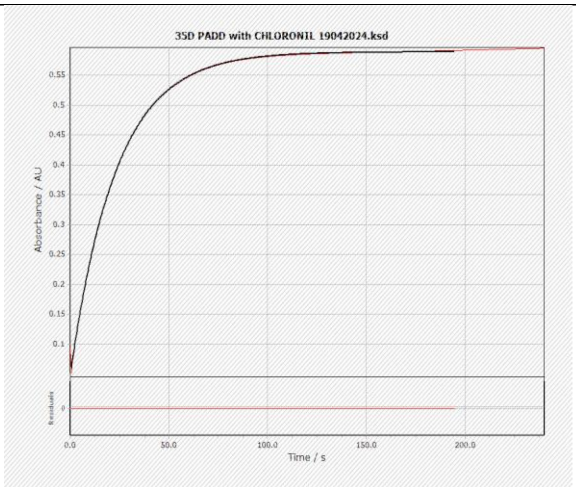

35°C D

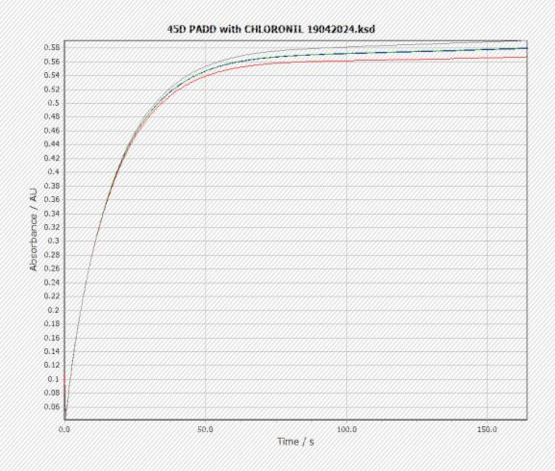

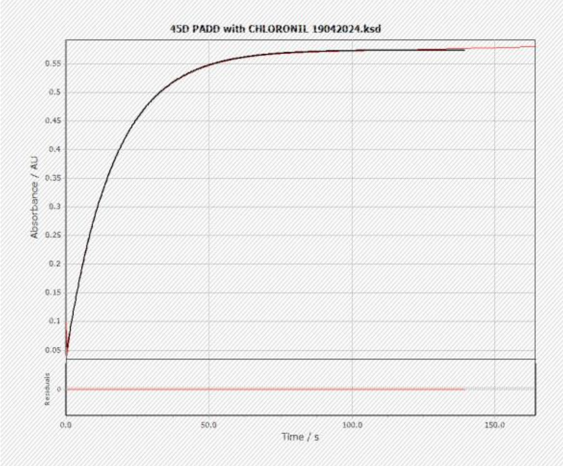

45°C D

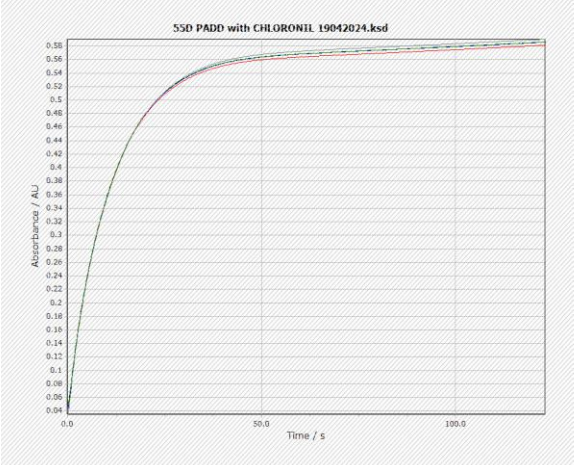

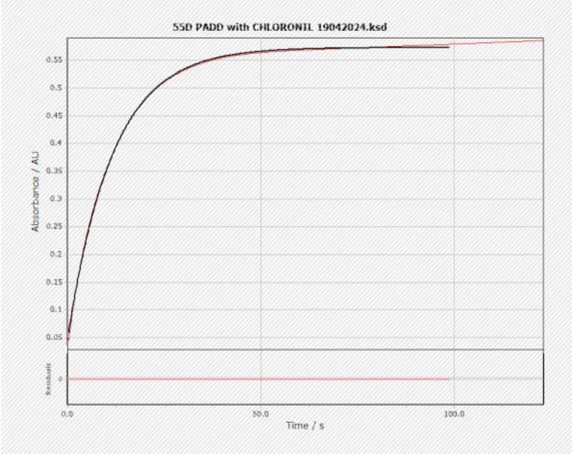

55°C D

Primary kinetic data for the rate constants in Table S5

Day 1 data (June 30, 2025)

Pseudo-first-order rate constants

k^{pfo} (s^{-1})

**Temp (°C)**	**Trial H1**	**Trial H2**	**Trial H3**	**Average k_H^{pfo} (s^{-1})**	**Stdev**	**k_{2H} [b] ($M^{-1}s^{-1}$)**	**Stdev [a]**
55	0.08432	0.08382	0.08535	0.084497	0.00078015	3.60E+01	0.33245
45	0.06006	0.0608	0.06	0.060287	0.000445571	2.57E+01	0.18987
35	0.04183	0.04187	0.04177	0.041823	5.03322E-05	1.78E+01	0.02145
25	0.02871	0.02848	0.0288	0.028663	0.000165025	1.22E+01	0.07032
15	0.01896	0.01891	0.01901	0.01896	5E-05	8.08E+00	0.02131

**Temp (°C)**	**Trial D1**	**Trial D2**	**Trial D3**	**Average k_D^{pfo} (s^{-1})**	**Stdev**	**k_{2D} [b] ($M^{-1}s^{-1}$)**	**Stdev [a]**
55	0.01231	0.01232	0.01215	0.01226	9.53939E-05	5.22E+00	0.04065
45	0.00802	0.0079	0.00784	0.00792	9.16515E-05	3.38E+00	0.03906
35	0.0051	0.00504	0.00512	0.00509	4.16333E-05	2.17E+00	0.01774
25	0.00323	0.00316	0.00315	0.00318	4.3589E-05	1.36E+00	0.01857
15	0.00199	0.00197	0.00197	0.00198	1.1547E-05	8.42E-01	0.00492

[a] = (Stdev(for k^{pfo})/k^{pfo})*k_2; [b] = k^{pfo}/(2[Cl_4Q]/3)

Three kinetic runs together with the averaged data	First-order kinetic fit of the averaged data as an example
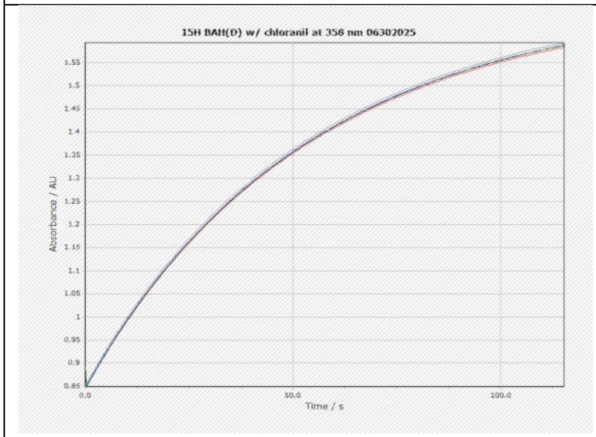	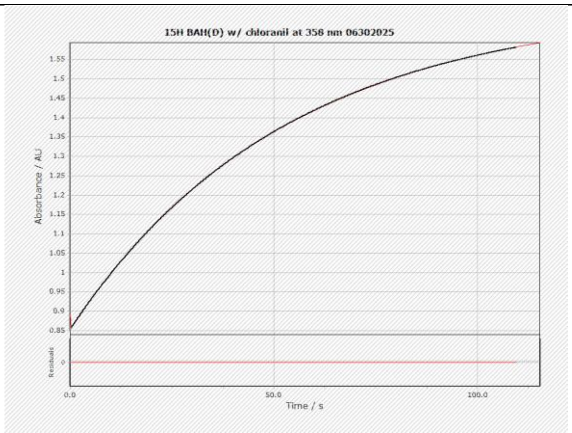
15°C H	

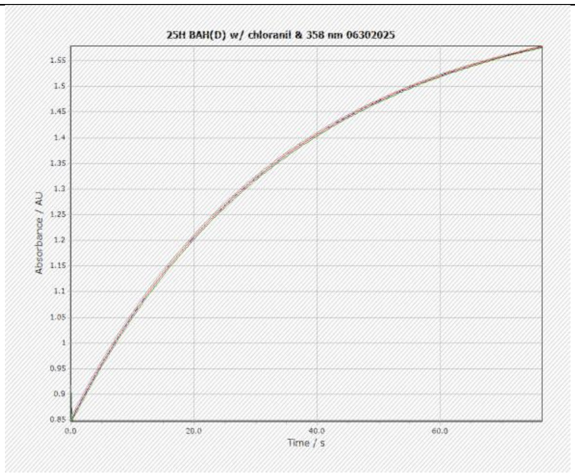

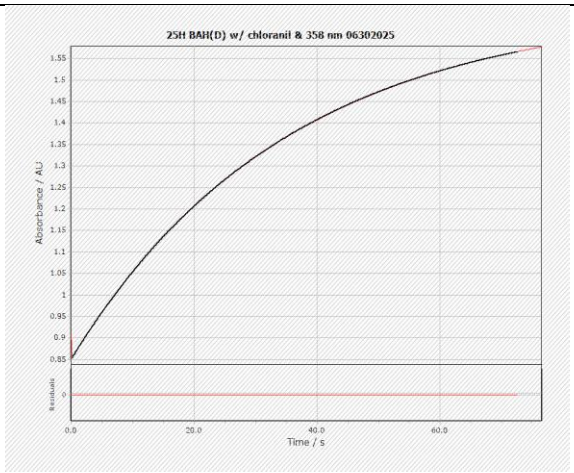

25°C H

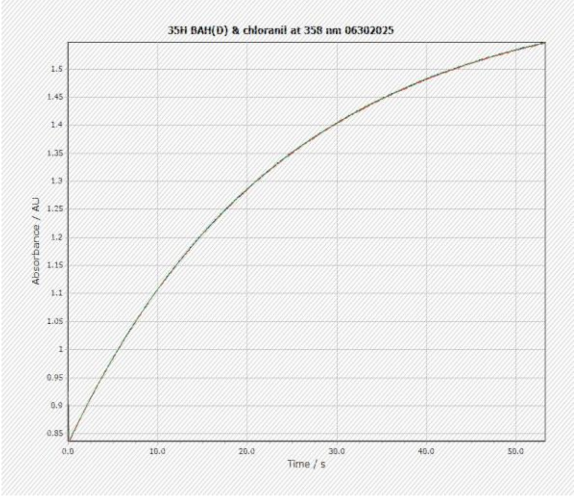

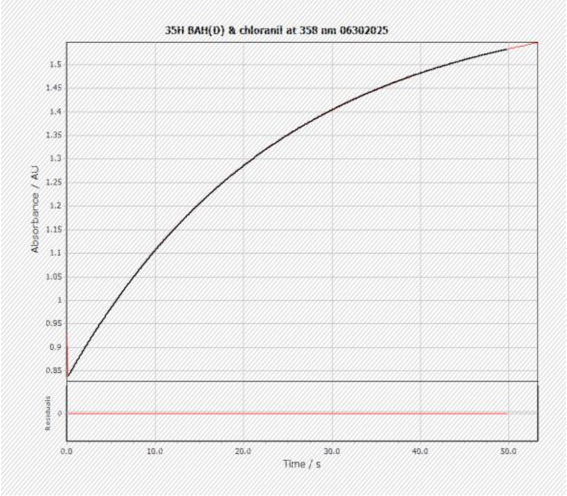

35°C H

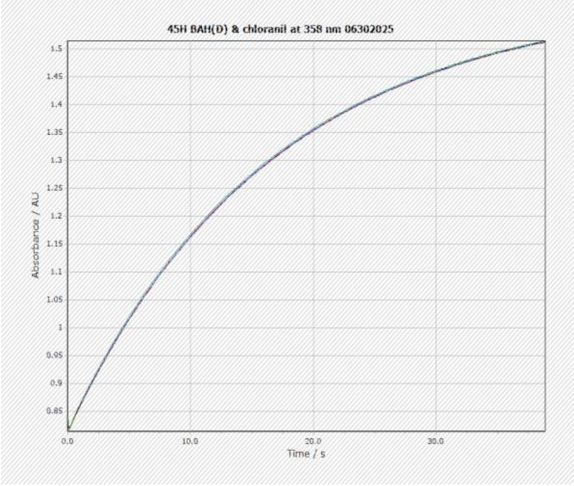

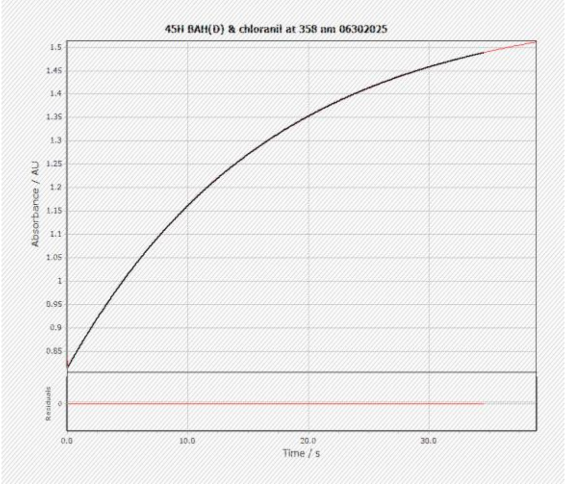

45°C H

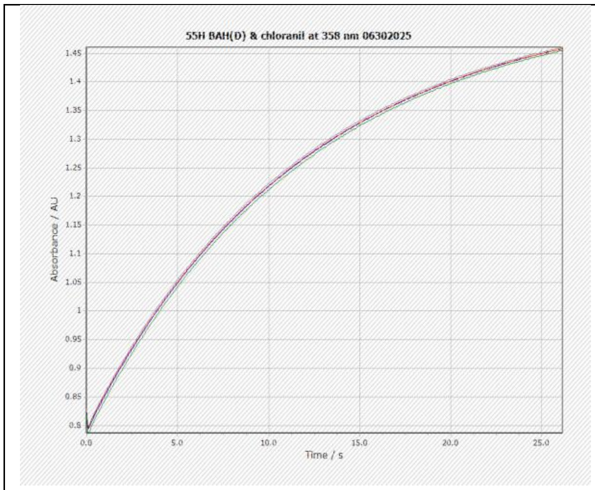
55H BAH(D) & chloranil at 358 nm 06302025
Absorbance / AU
Time / s

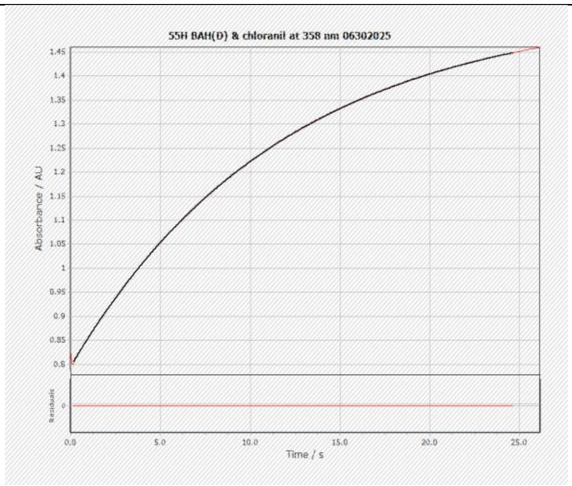
55H BAH(D) & chloranil at 358 nm 06302025
Absorbance / AU
Residuals
Time / s

55℃ H

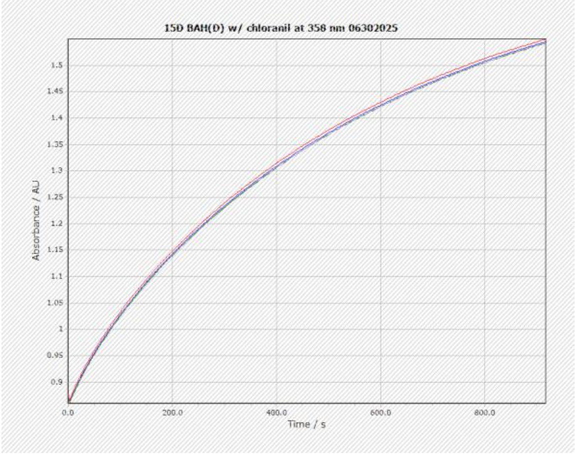
15D BAH(D) w/ chloranil at 358 nm 06302025
Absorbance / AU
Time / s

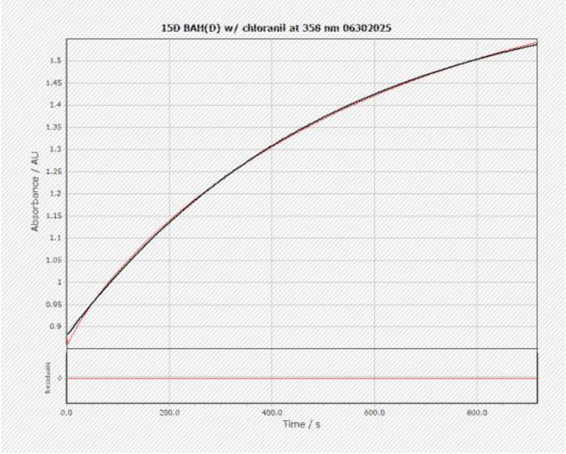
15D BAH(D) w/ chloranil at 358 nm 06302025
Absorbance / AU
Residuals
Time / s

15℃ D

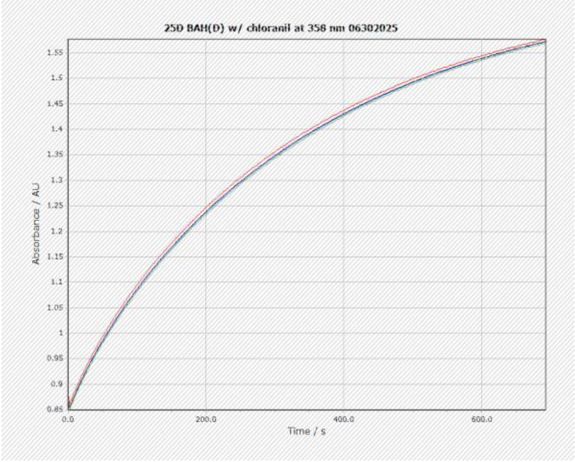
25D BAH(D) w/ chloranil at 358 nm 06302025
Absorbance / AU
Time / s

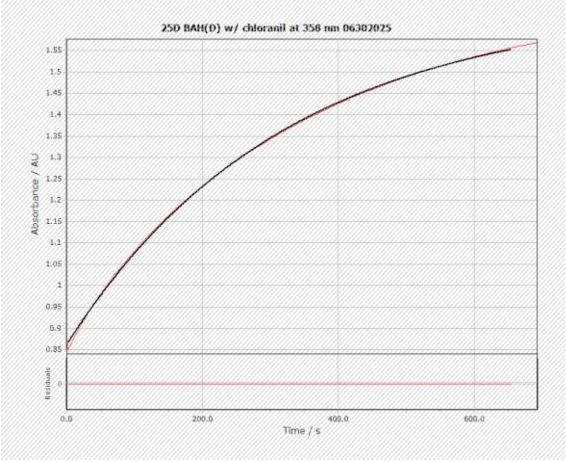
25D BAH(D) w/ chloranil at 358 nm 06302025
Absorbance / AU
Residuals
Time / s

25℃ D

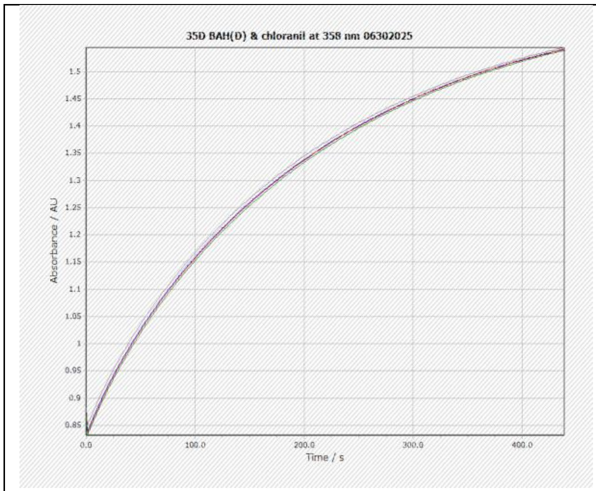

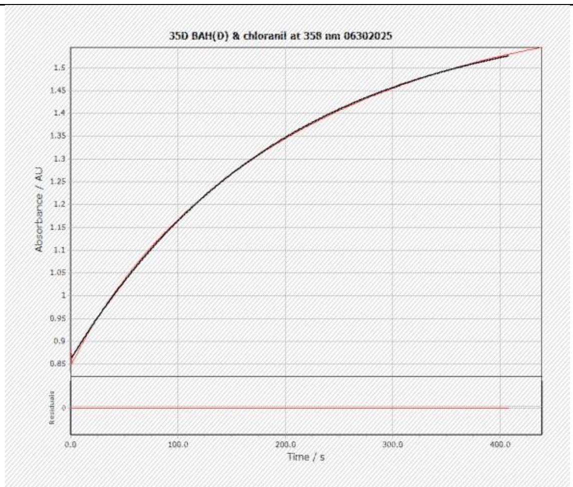

35°C D

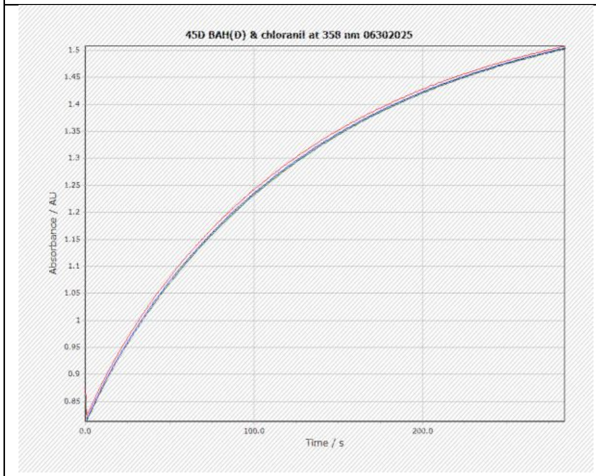

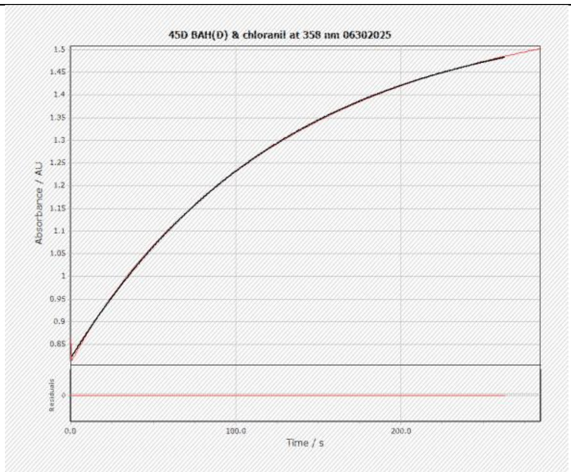

45°C D

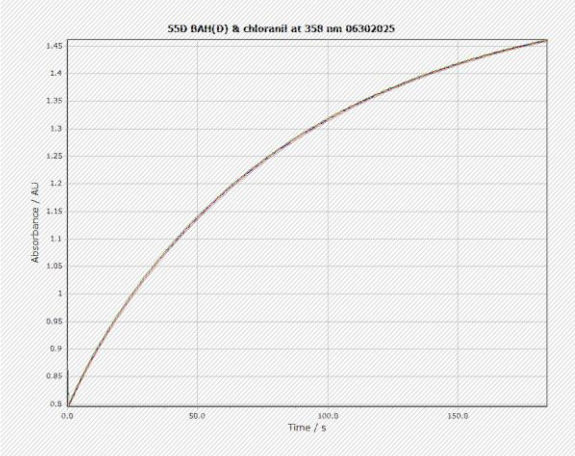

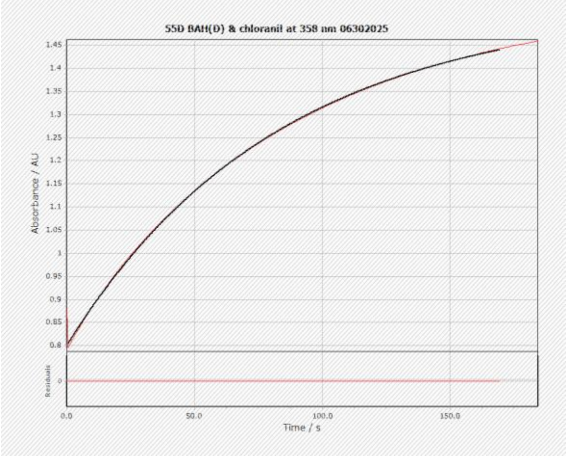

55°C D

Day 2 data (July 08, 2025)

Pseudo-first-order rate constants k^{pfo} (s^{-1})							
**Temp (°C)**	**Trial H1**	**Trial H2**	**Trial H3**	**Average k_H^{pfo} (s^{-1})**	**Stdev**	**k_{2H} [b] ($M^{-1}s^{-1}$)**	**Stdev [a]**
55	0.08729	0.08806	0.08715	0.0875	0.00049	3.73E+01	0.20881
45	0.06198	0.06124	0.06162	0.061613	0.000370045	2.63E+01	0.15769
35	0.0421	0.04197	0.04256	0.04221	0.00031	1.80E+01	0.13210
25	0.02867	0.02862	0.0289	0.02873	0.000149332	1.22E+01	0.06364
15	0.01932	0.01929	0.01907	0.019227	0.000136504	8.19E+00	0.05817
**Temp (°C)**	**Trial D1**	**Trial D2**	**Trial D3**	**Average k_D^{pfo} (s^{-1})**	**Stdev**	**k_{2D} [b] ($M^{-1}s^{-1}$)**	**Stdev [a]**
55	0.01266	0.01266	0.01261	0.01264	2.88675E-05	5.39E+00	0.01230
45	0.00819	0.00814	0.00816	0.00816	2.51661E-05	3.48E+00	0.01072
35	0.00525	0.00523	0.0053	0.00526	3.60555E-05	2.24E+00	0.01536
25	0.00339	0.00339	0.00336	0.00338	1.73205E-05	1.44E+00	0.00738
15	0.00203	0.002	0.00197	0.00200	3E-05	8.52E-01	0.01278

[a] = (Stdev(for k^{pfo})/k^{pfo})*k_2; [b] = k^{pfo}/(2[Cl_4Q]/3)

Three kinetic runs together with the averaged data	First-order kinetic fit of the averaged data as an example

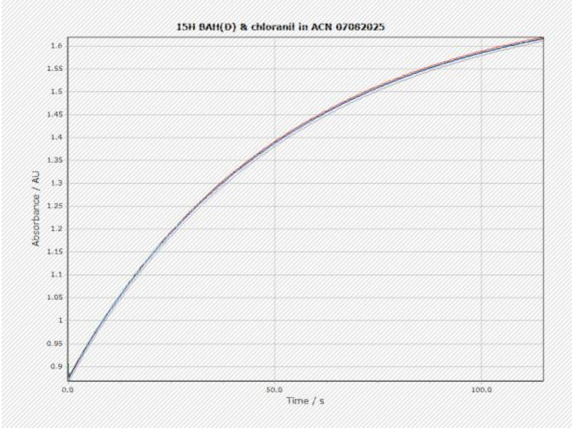

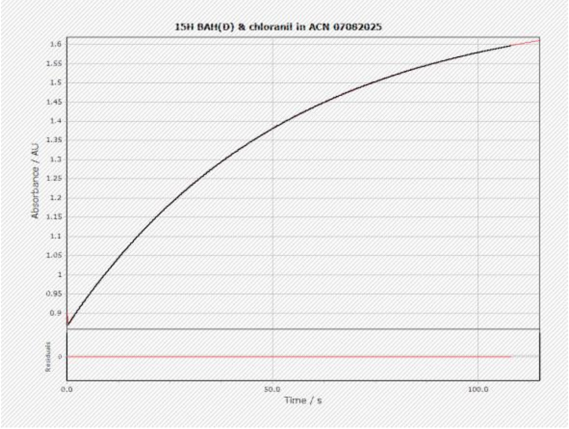

15°C H

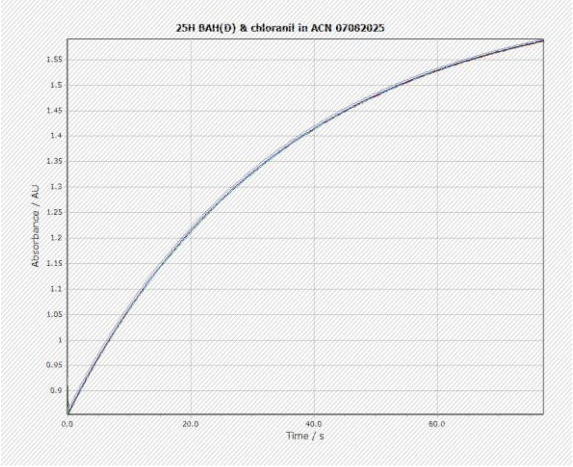

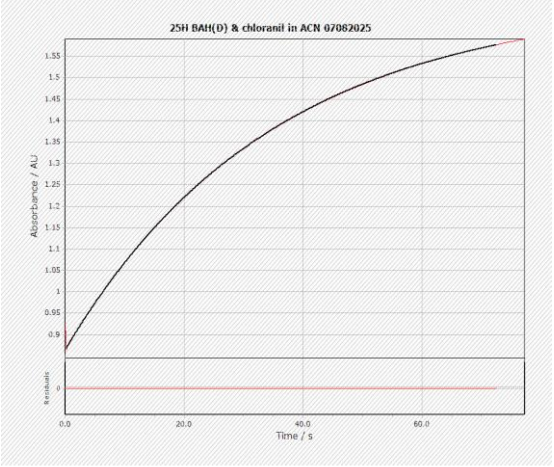

25°C H

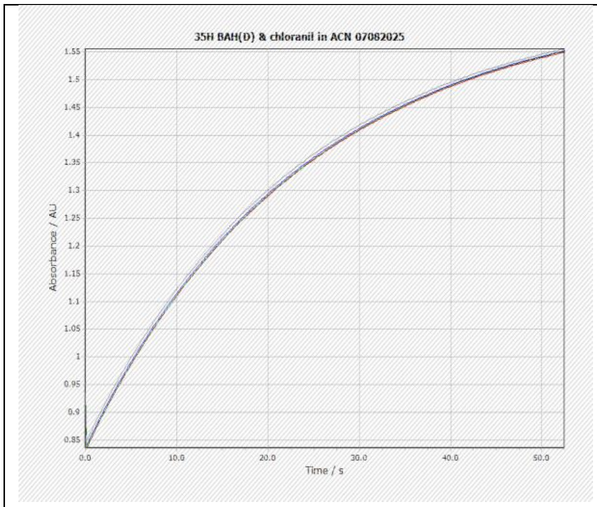
35H BAH(D) & chloranil in ACN 07082025
Absorbance / AU
Time / s

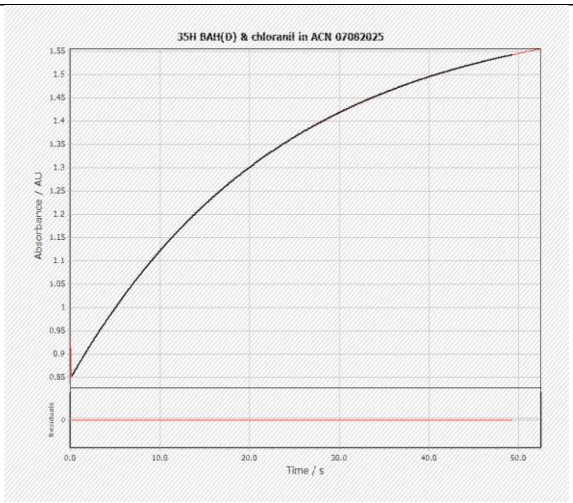
35H BAH(D) & chloranil in ACN 07082025
Absorbance / AU
Residuals
Time / s

35℃ H

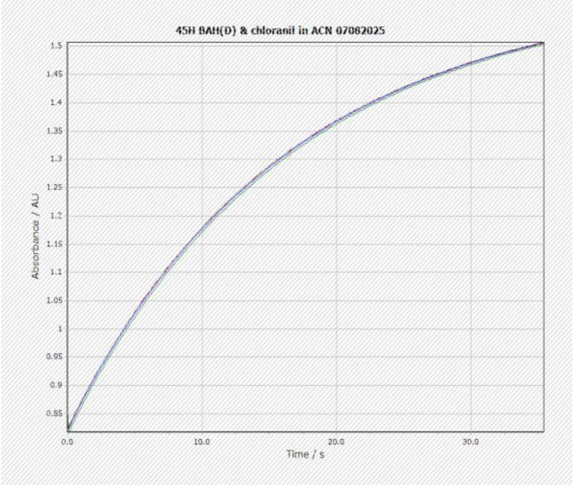
45H BAH(D) & chloranil in ACN 07082025
Absorbance / AU
Time / s

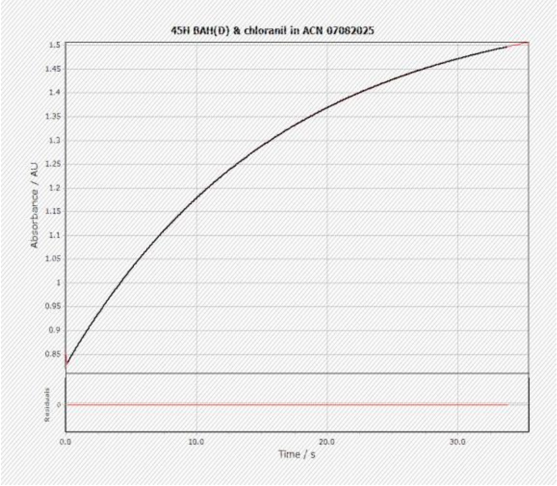
45H BAH(D) & chloranil in ACN 07082025
Absorbance / AU
Residuals
Time / s

45℃ H

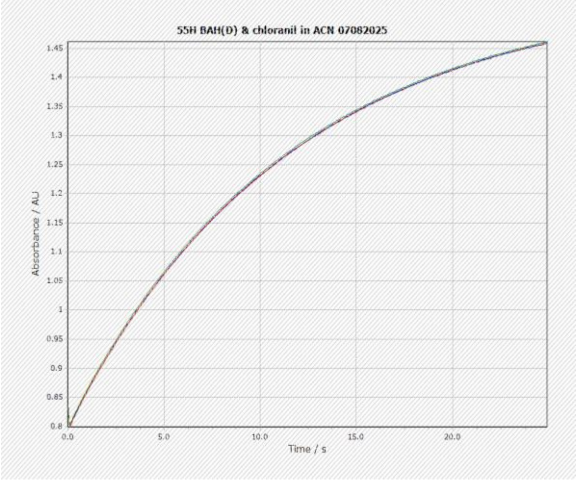
55H BAH(D) & chloranil in ACN 07082025
Absorbance / AU
Time / s

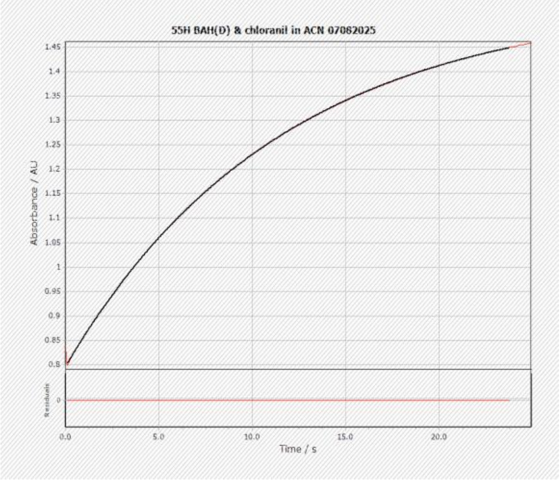
55H BAH(D) & chloranil in ACN 07082025
Absorbance / AU
Residuals
Time / s

55℃ H

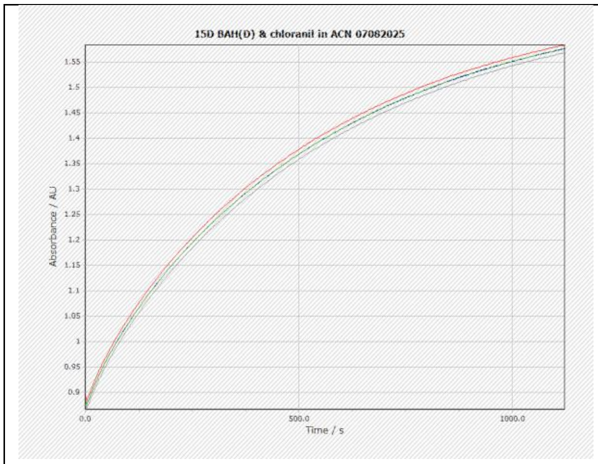
15D BAH(D) & chloranil in ACN 07082025
Absorbance / AU
Time / s

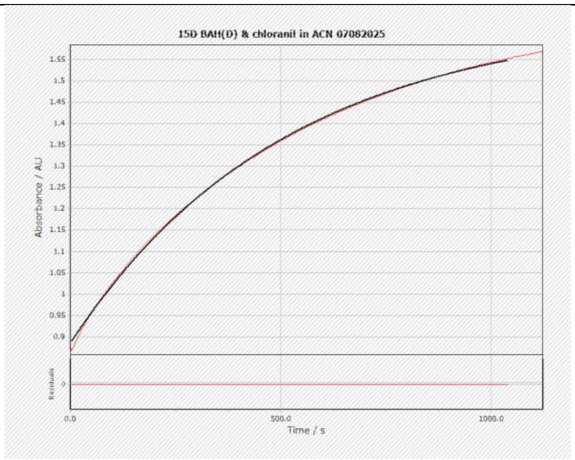
15D BAH(D) & chloranil in ACN 07082025
Absorbance / AU
Residuals
Time / s

15℃ D

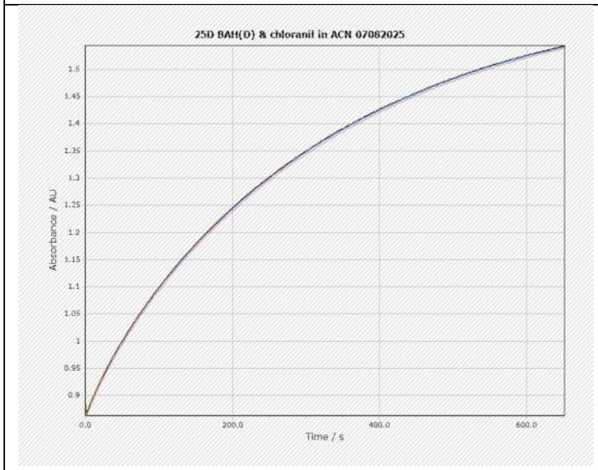
25D BAH(D) & chloranil in ACN 07082025
Absorbance / AU
Time / s

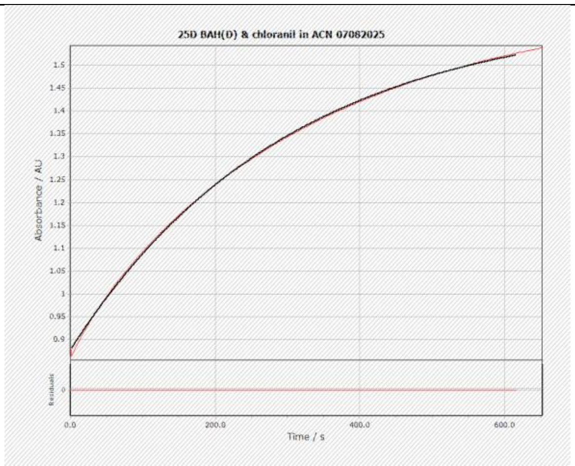
25D BAH(D) & chloranil in ACN 07082025
Absorbance / AU
Residuals
Time / s

25℃ D

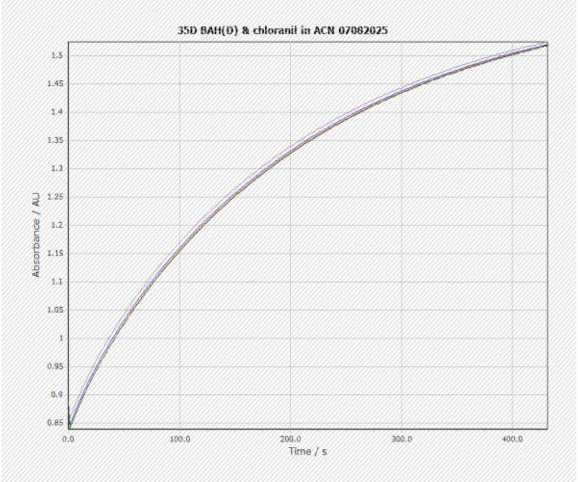
35D BAH(D) & chloranil in ACN 07082025
Absorbance / AU
Time / s

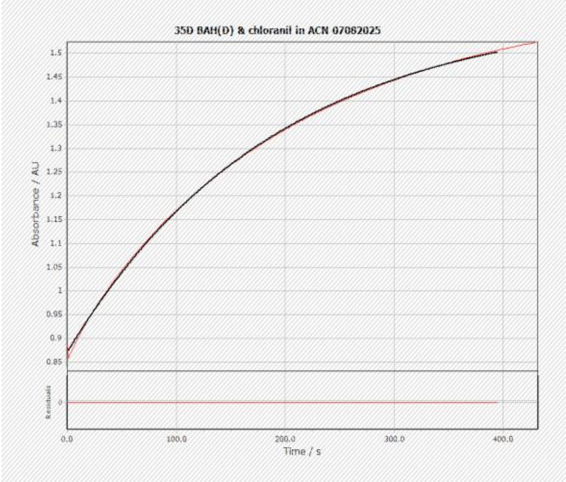
35D BAH(D) & chloranil in ACN 07082025
Absorbance / AU
Residuals
Time / s

35℃ D

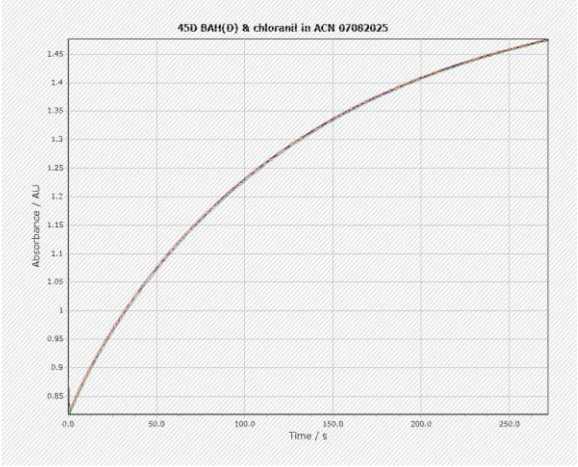	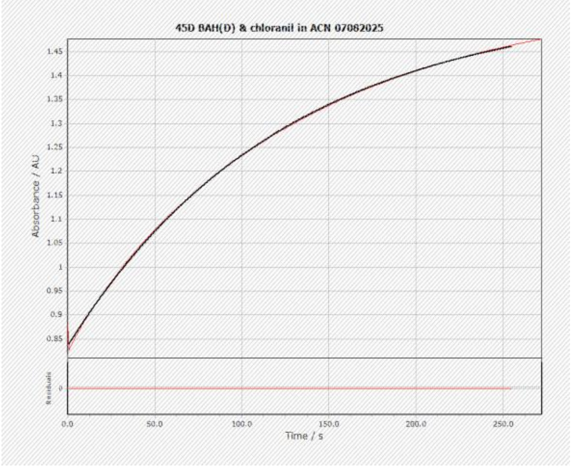
45°C D	
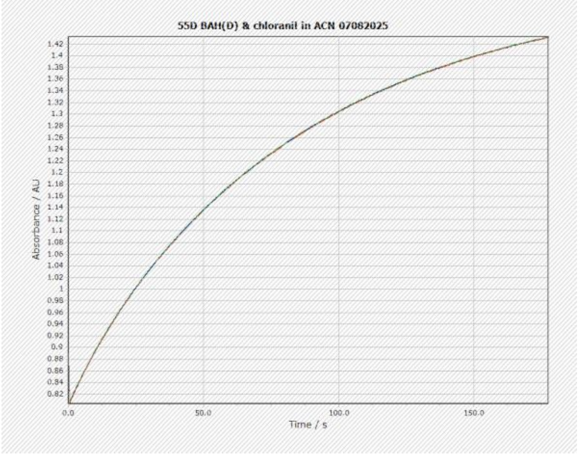	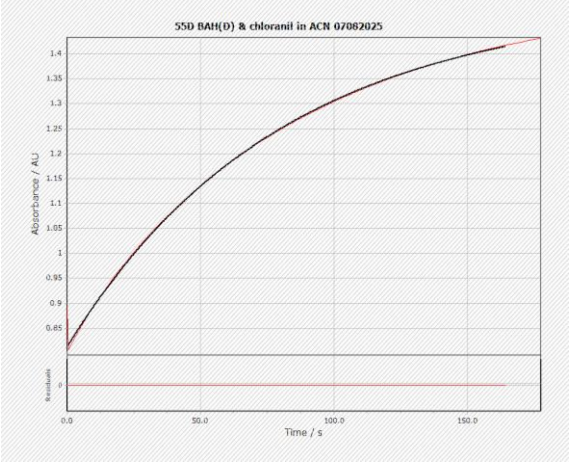
55°C D	

Day 3 data (July 09, 2025)

Pseudo-first-order rate constants

k^{pfo} (s^{-1})

**Temp (°C)**	**Trial H1**	**Trial H2**	**Trial H3**	**Average k_H^{pfo} (s^{-1})**	**Stdev**	**k_{2H} [b] ($M^{-1}s^{-1}$)**	**Stdev [a]**
55	0.08888	0.08822	0.08927	0.08879	0.000530754	3.78E+01	0.22617
45	0.06272	0.06219	0.06297	0.062627	0.000398288	2.67E+01	0.16973
35	0.0425	0.0432	0.04271	0.042803	0.000359212	1.82E+01	0.15307
25	0.02942	0.02957	0.02954	0.02951	7.93725E-05	1.26E+01	0.03382
15	0.01932	0.01954	0.01945	0.019437	0.000110604	8.28E+00	0.04713

**Temp (°C)**	**Trial D1**	**Trial D2**	**Trial D3**	**Average k_D^{pfo} (s^{-1})**	**Stdev**	**k_{2D} [b] ($M^{-1}s^{-1}$)**	**Stdev [a]**
55	0.01272	0.01258	0.01262	0.01264	7.2111E-05	5.39E+00	0.03073
45	0.0081	0.00811	0.00818	0.00813	4.3589E-05	3.46E+00	0.01857
35	0.005	0.00506	0.00506	0.00504	3.4641E-05	2.15E+00	0.01476
25	0.00321	0.00322	0.00323	0.00322	1E-05	1.37E+00	0.00426
15	0.00198	0.00196	0.00196	0.00197	1.1547E-05	8.38E-01	0.00492

[a] = (Stdev(for k^{pfo})/k^{pfo})*k_2; [b] = k^{pfo}/(2[Cl_4Q]/3)

<table>
<tr><td>Three kinetic runs together with the averaged data</td><td>First-order kinetic fit of the averaged data as an example</td></tr>
<tr><td>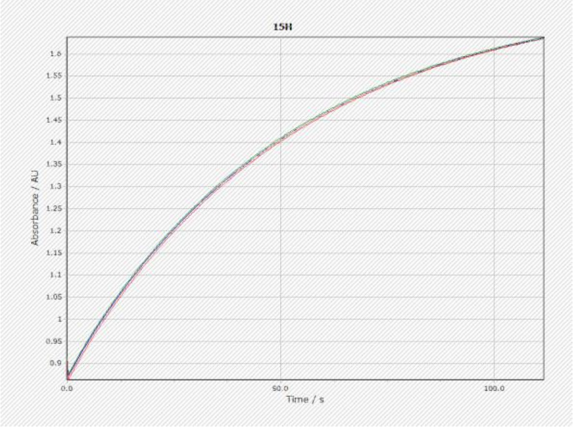
</td><td>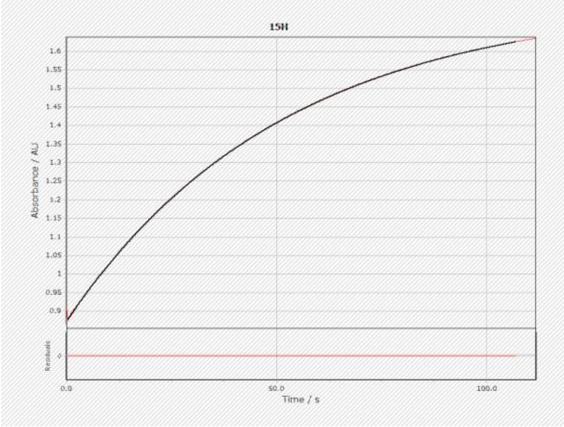
</td></tr>
<tr><td colspan="2">15°C H</td></tr>
<tr><td>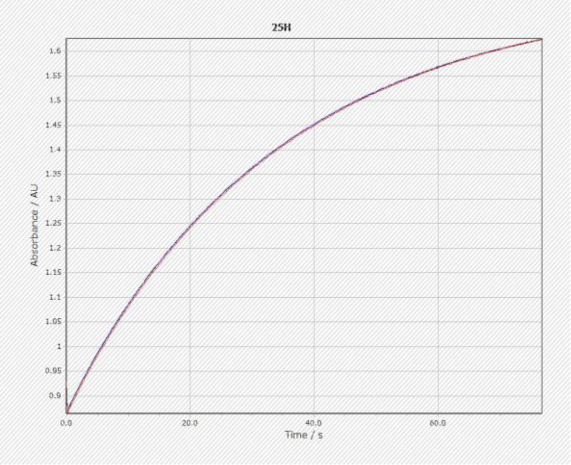
</td><td>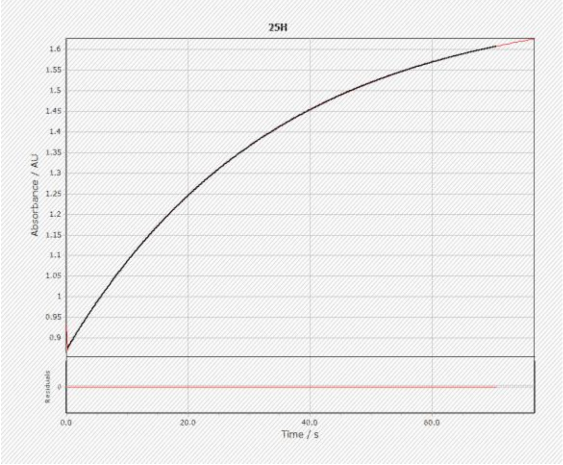
</td></tr>
<tr><td colspan="2">25°C H</td></tr>
<tr><td>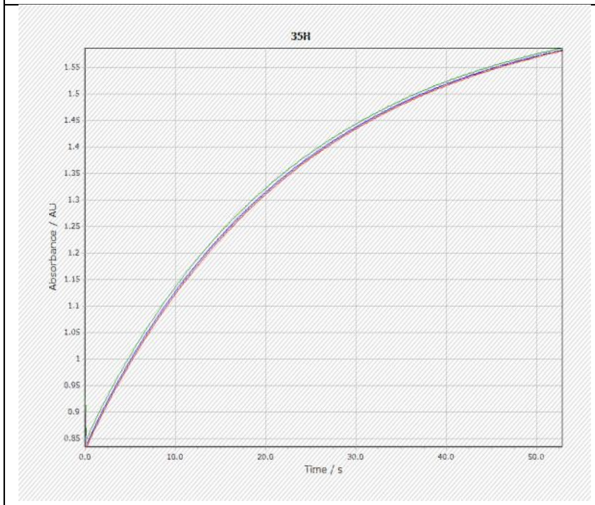
</td><td>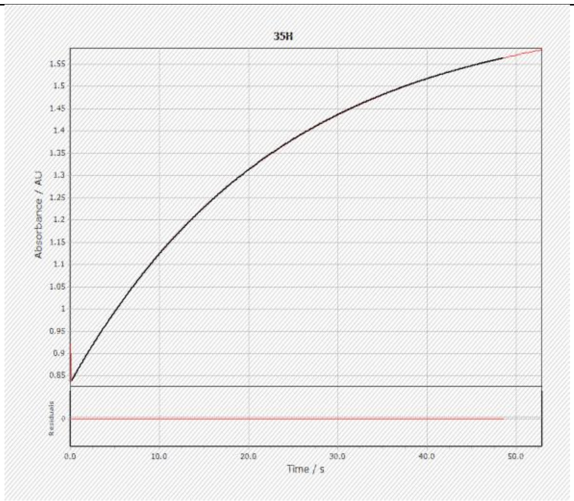
</td></tr>
<tr><td colspan="2">35°C H</td></tr>
</table>

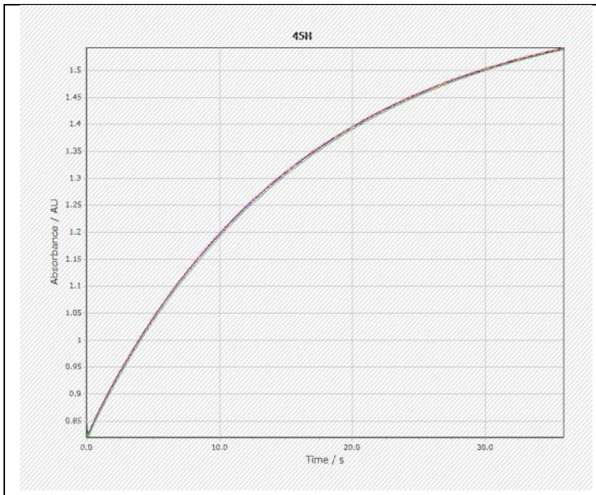

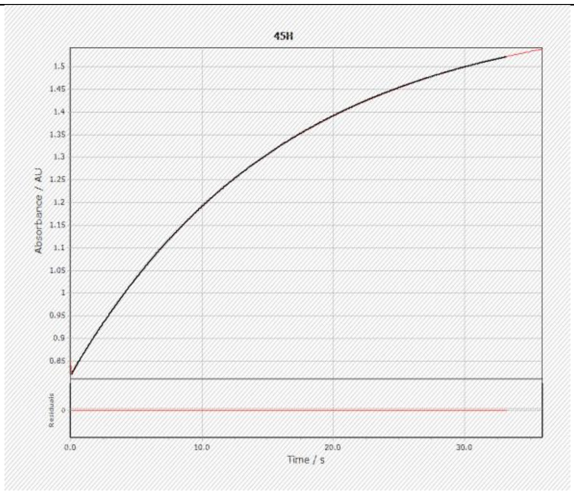

45°C H

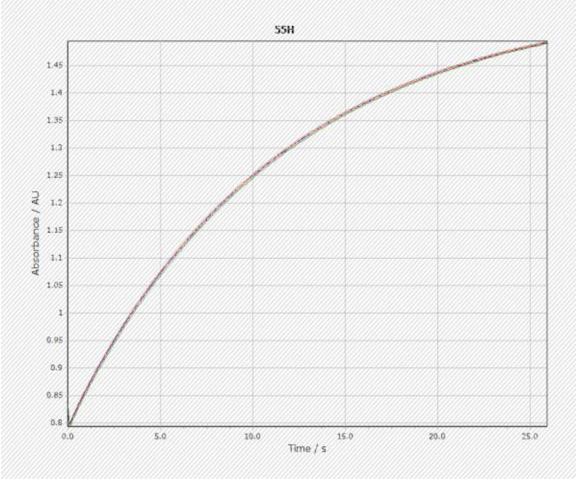

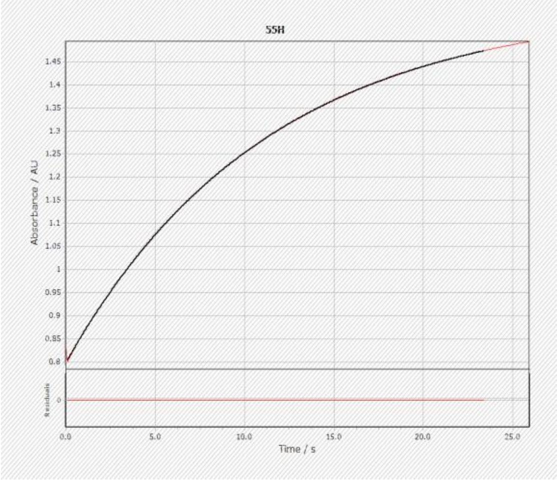

55°C H

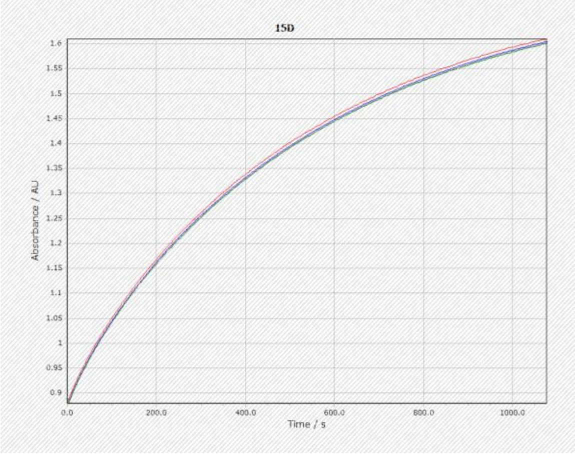

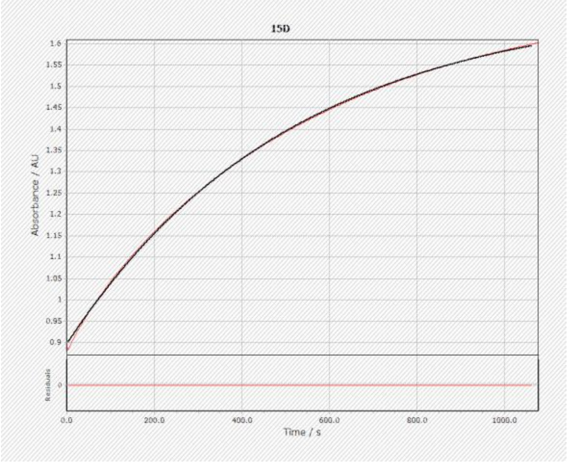

15°C D

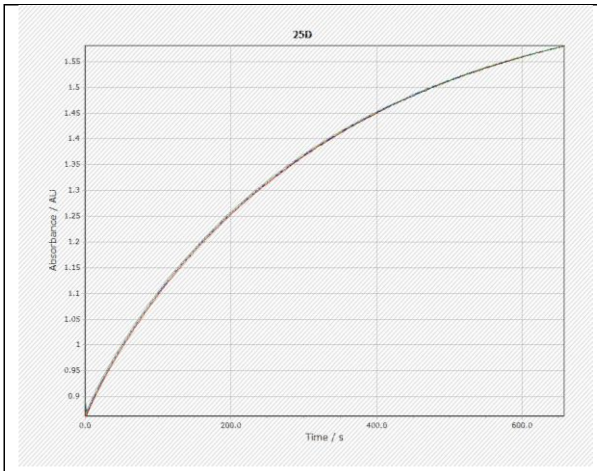

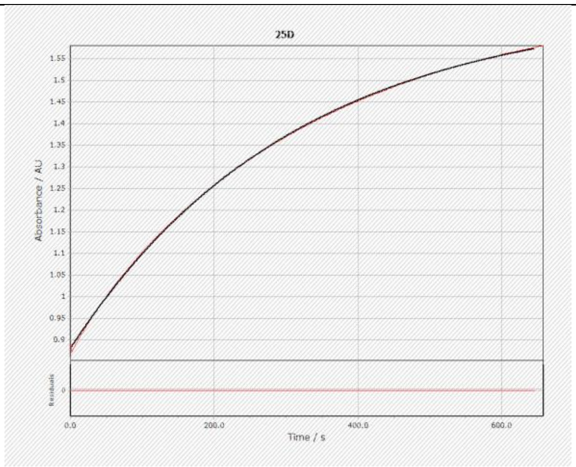

25°C D

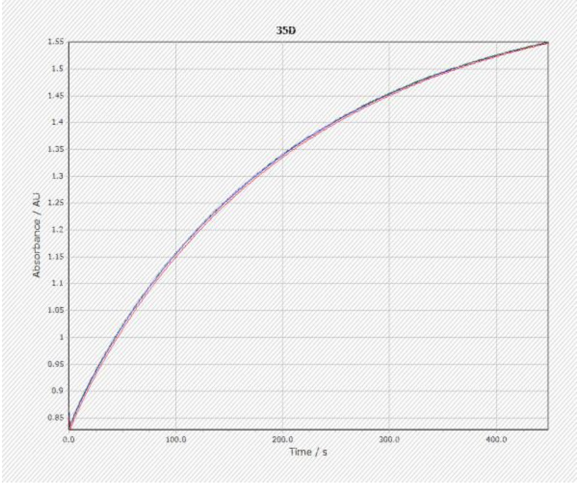

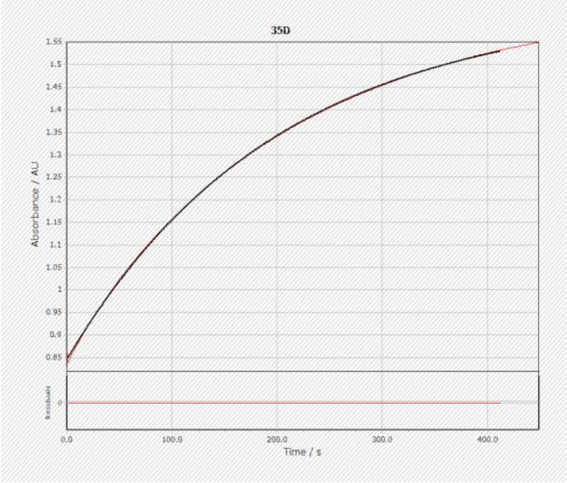

35°C D

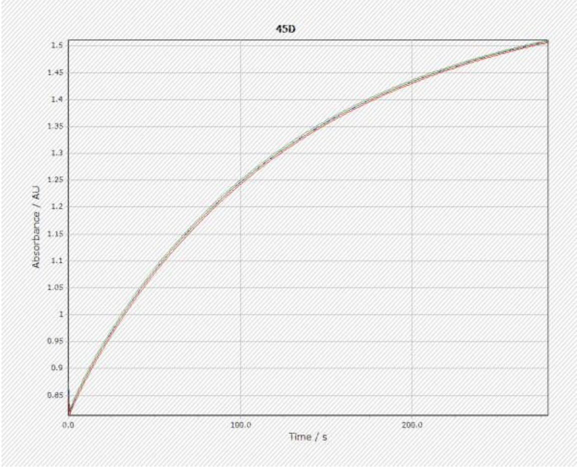

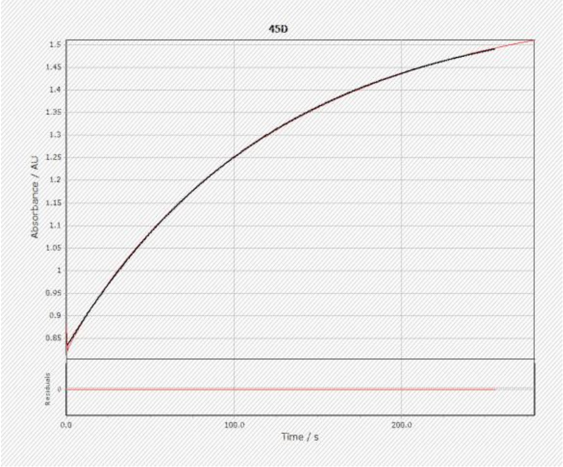

45°C D

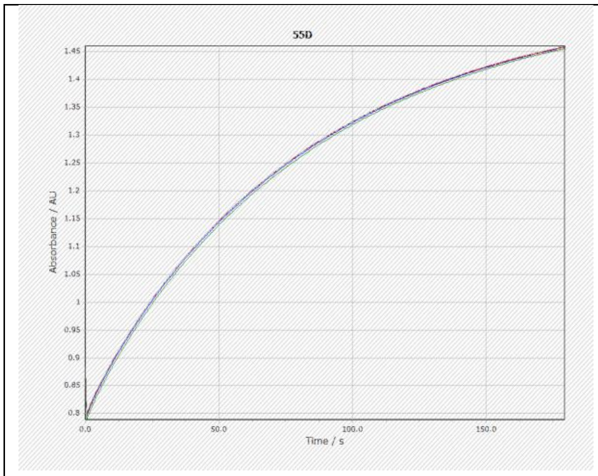

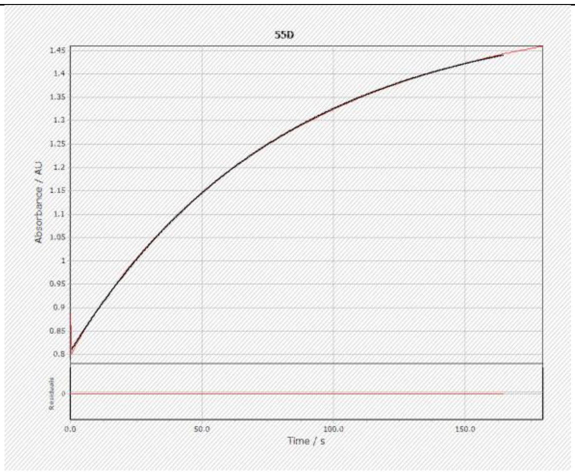

55°C D

Primary kinetic data for the rate constants in Table S6

Day 1 data (October 12, 2024)

Pseudo-first-order rate constants

k^{pfo} (s^{-1})

**Temp (°C)**	**Trial H1**	**Trial H2**	**Trial H3**	**Average k_H^{pfo} (s^{-1})**	**Stdev**	**k_{2H} [b] (M^{-1}s^{-1})**	**Stdev [a]**
55	1.92076	1.96610	1.95538	1.9474	0.0236966	4.43E+02	5.38560
45	1.46644	1.42815	1.46813	1.4542	0.0226104	3.31E+02	5.13873
35	1.06936	1.08779	1.06525	1.0741	0.0120042	2.44E+02	2.72823
25	0.78575	0.79586	0.80297	0.7949	0.0086534	1.81E+02	1.96669
15	0.56638	0.56647	0.57436	0.5691	0.0045815	1.29E+02	1.04125

**Temp (°C)**	**Trial D1**	**Trial D2**	**Trial D3**	**Average k_D^{pfo} (s^{-1})**	**Stdev**	**k_{2D} [b] (M^{-1}s^{-1})**	**Stdev [a]**
55	0.32557	0.32141	0.31942	0.3221	0.003138158	7.32E+01	7.13E-01
45	0.22879	0.22857	0.22821	0.2285	0.000292803	5.19E+01	6.65E-02
35	0.16194	0.16245	0.16395	0.1628	0.001044844	3.70E+01	2.37E-01
25	0.11114	0.11178	0.11186	0.1116	0.000394631	2.54E+01	8.97E-02
15	0.07566	0.07552	0.07511	0.0754	0.000285832	1.71E+01	6.50E-02

[a] = (Stdev(for k^{pfo})/k^{pfo})*k_2; [b] = k^{pfo}/(2[Cl_4Q]/3)

<table>
<tr><td>Three kinetic runs together with the averaged data</td><td>First-order kinetic fit of the averaged data as an example</td></tr>
<tr><td>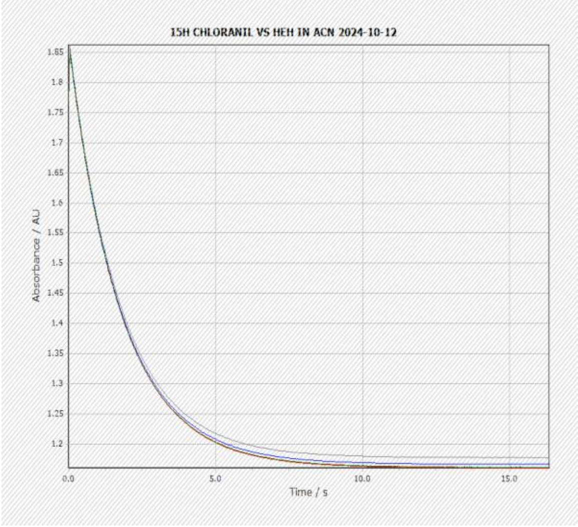
</td><td>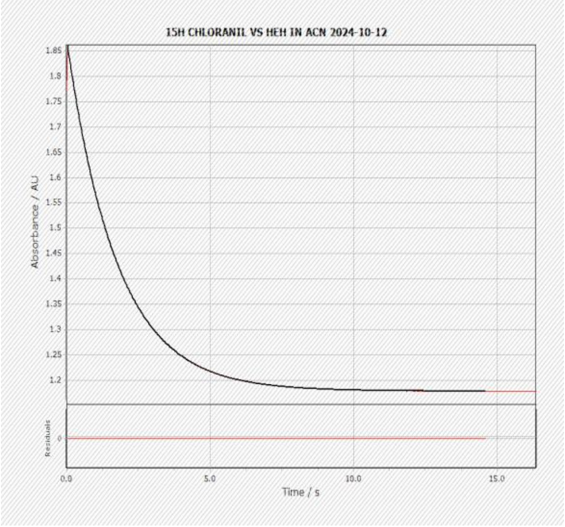
</td></tr>
<tr><td colspan="2">15℃ H</td></tr>
<tr><td>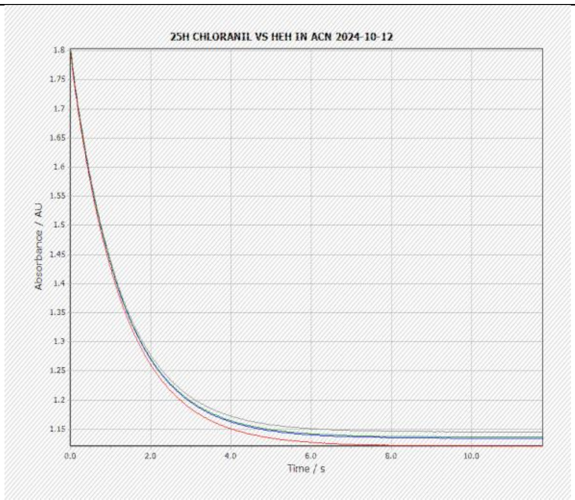
</td><td>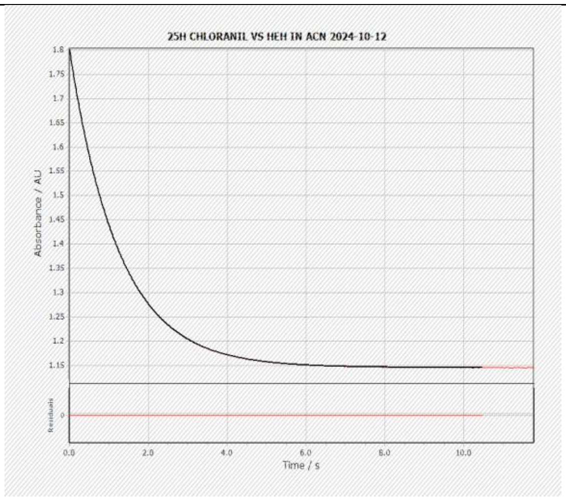
</td></tr>
<tr><td colspan="2">25℃ H</td></tr>
</table>

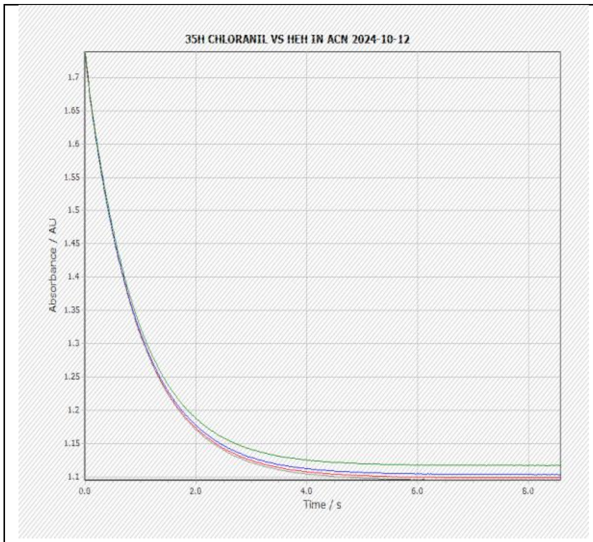
35H CHLORANIL VS HEH IN ACN 2024-10-12
Absorbance / AU
Time / s

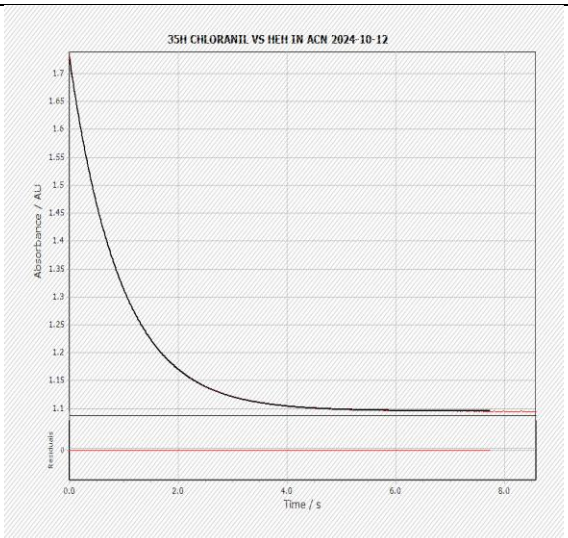
35H CHLORANIL VS HEH IN ACN 2024-10-12
Absorbance / AU
Residuals
Time / s

35°C H

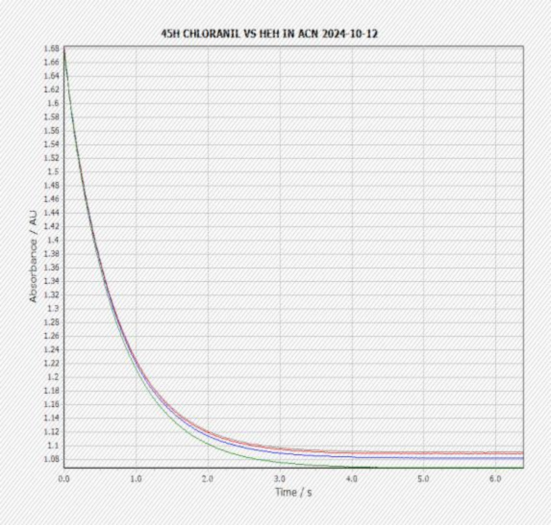
45H CHLORANIL VS HEH IN ACN 2024-10-12
Absorbance / AU
Time / s

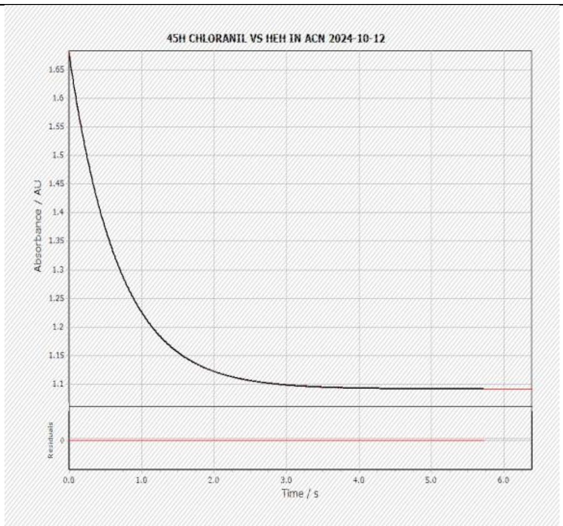
45H CHLORANIL VS HEH IN ACN 2024-10-12
Absorbance / AU
Residuals
Time / s

45°C H

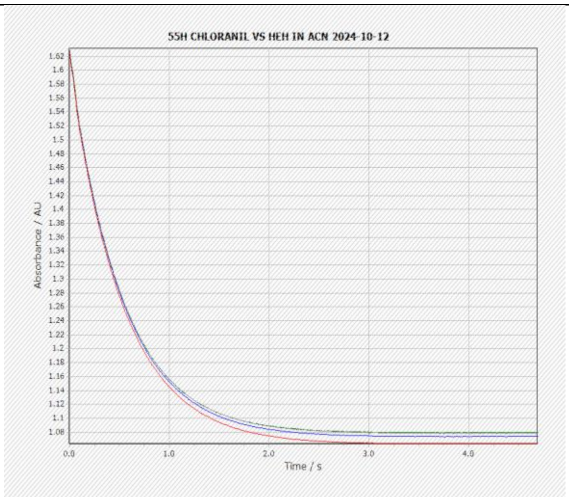
55H CHLORANIL VS HEH IN ACN 2024-10-12
Absorbance / AU
Time / s

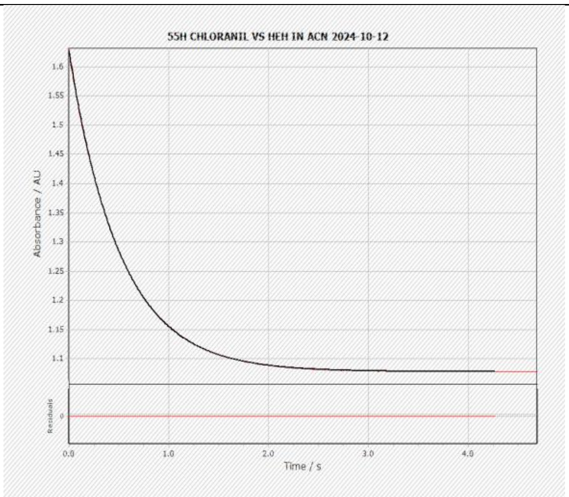
55H CHLORANIL VS HEH IN ACN 2024-10-12
Absorbance / AU
Residuals
Time / s

55℃ H

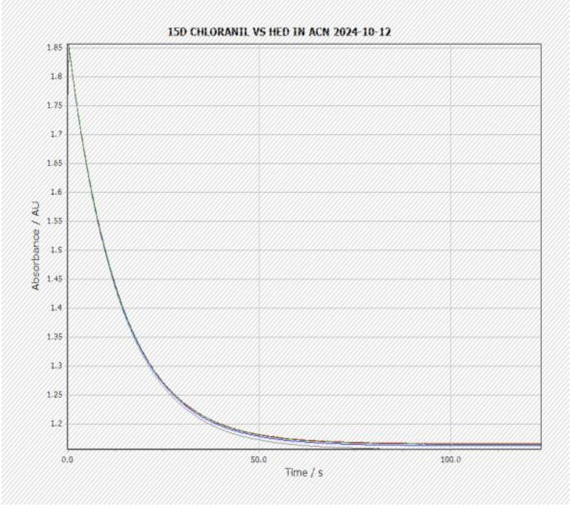

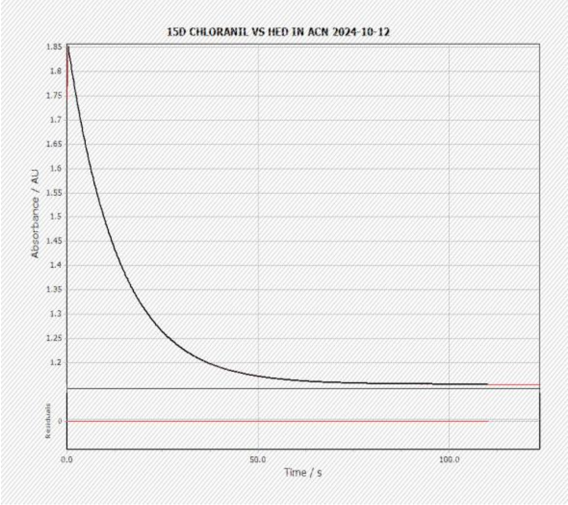

15℃ D

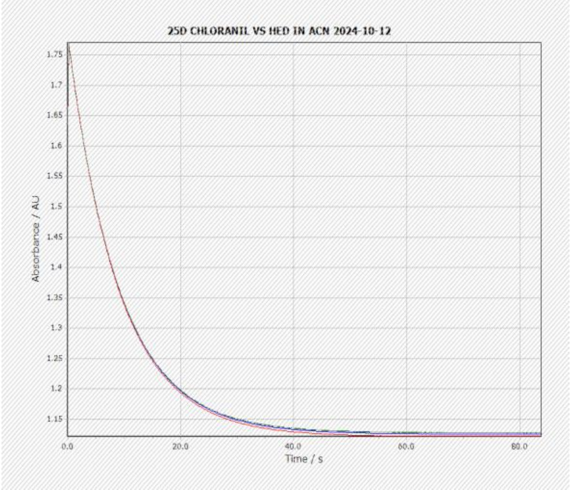

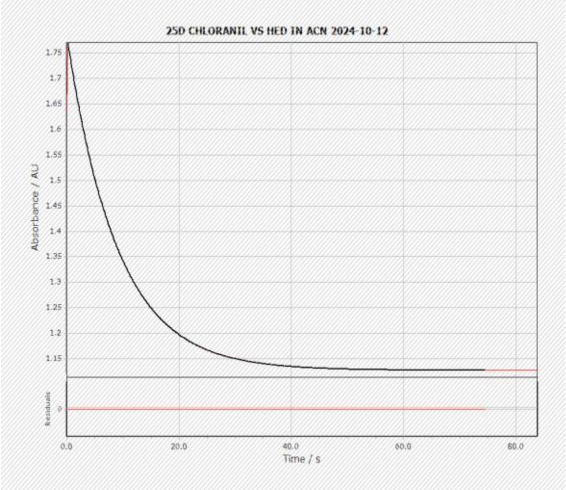

25℃ D

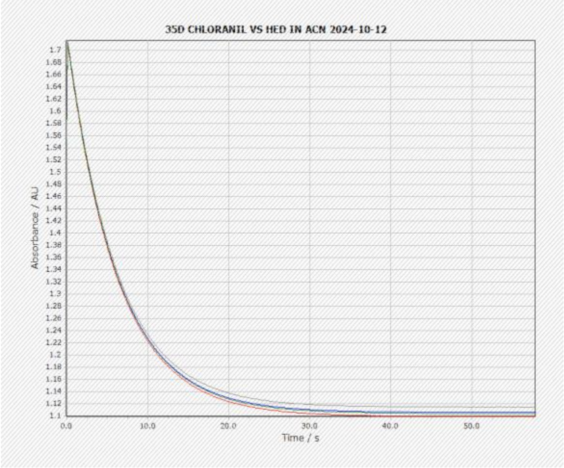

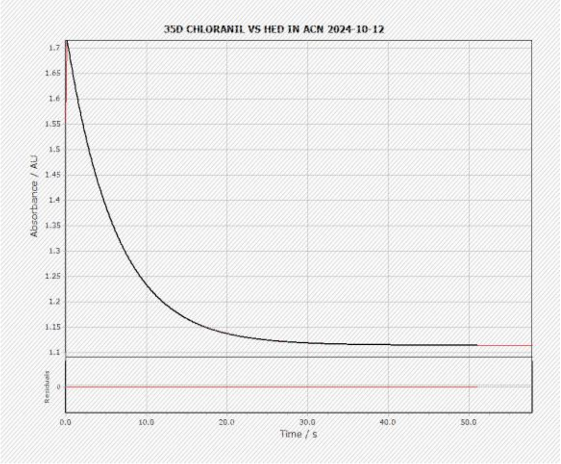

35℃ D

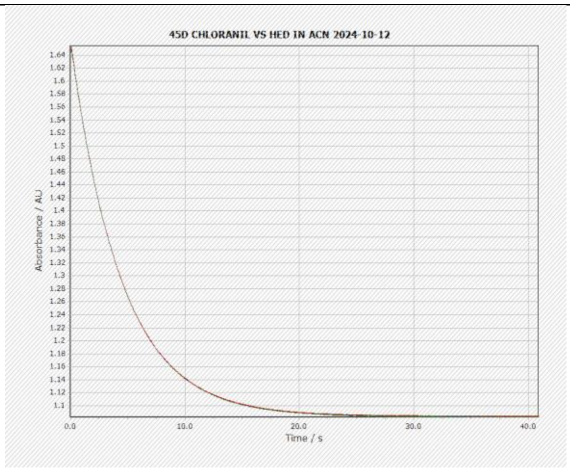

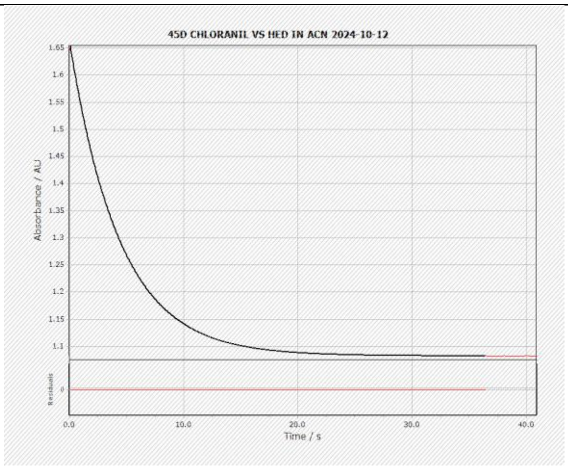

45°C D

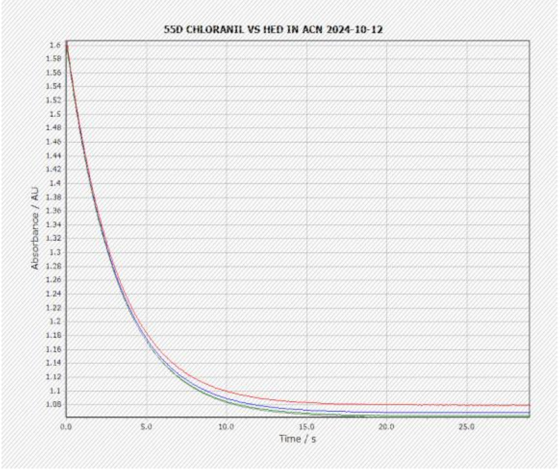

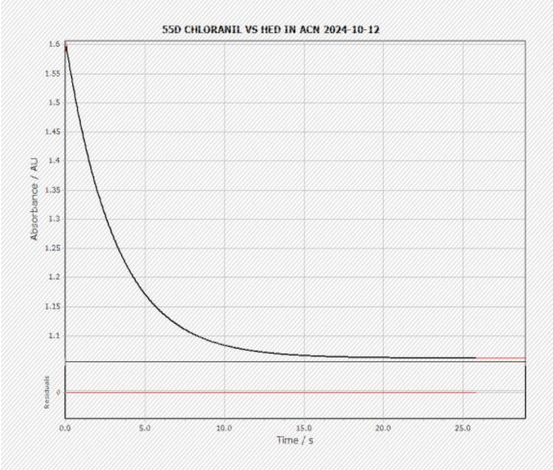

55°C D

Day 2 data (October 14, 2024)

Pseudo-first-order rate constants

Temp (°C)	k^{pfo} (s^{-1}) Trial H1	Trial H2	Trial H3	Trial H4	Trial H5	Trial H6	Average k_H^{pfo} (s^{-1})	Stdev	k_{2H} [b] ($M^{-1}s^{-1}$)	Stdev [a]
55	1.89727	1.89996	1.87107	1.87331	1.89798	1.8811	1.8868	0.0131886	4.29E+02	2.99742
45	1.38098	1.39837	1.41092	1.4137	1.40138	1.41885	1.4040	0.0136453	3.19E+02	3.10121
35	1.05189	1.04852	1.05375	1.03936	1.04191	1.05077	1.0477	0.0057851	2.38E+02	1.31479
25	0.76947	0.76276	0.76644	0.76948	0.77545	0.76671	0.7684	0.0042549	1.75E+02	0.96702
15	0.54133	0.54045	0.54221	0.54533	0.54537	0.54798	0.5438	0.0029026	1.24E+02	0.65969

Temp (°C)	Trial D1	Trial D2	Trial D3	Trial D4	Trial D5	Trial D6	Average k_D^{pfo} (s^{-1})	Stdev	k_{2D} [b] ($M^{-1}s^{-1}$)	Stdev [a]
55	0.31151	0.31116	0.31256	0.31225	0.31263	0.31116	0.3119	0.00068344	7.09E+01	0.15533
45	0.2236	0.22599	0.22662	0.22441	0.22387	0.22295	0.2246	0.00143525	5.10E+01	0.32619
35	0.15766	0.15907	0.15792	0.15784	0.15848	0.15858	0.1583	0.00054016	3.60E+01	0.12276
25	0.10945	0.10958	0.10743	0.10843	0.10812	0.10775	0.1085	0.00088498	2.47E+01	0.20113
15	0.07235	0.07161	0.07221	0.07306	0.07211	0.07207	0.0722	0.00047496	1.64E+01	0.10795

[a] = (Stdev(for k^{pfo})/k^{pfo})*k_2; [b] = k^{pfo}/(2[Cl_4Q]/3)

<table>
<tr><td>Six kinetic runs together with the averaged data</td><td>First-order kinetic fit of the averaged data as an example</td></tr>
<tr><td>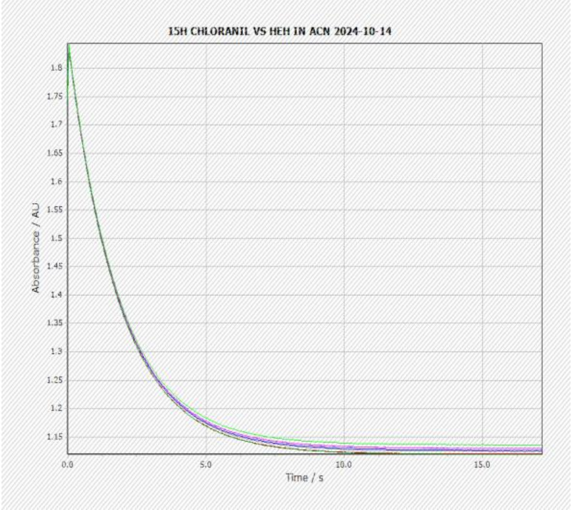
</td><td>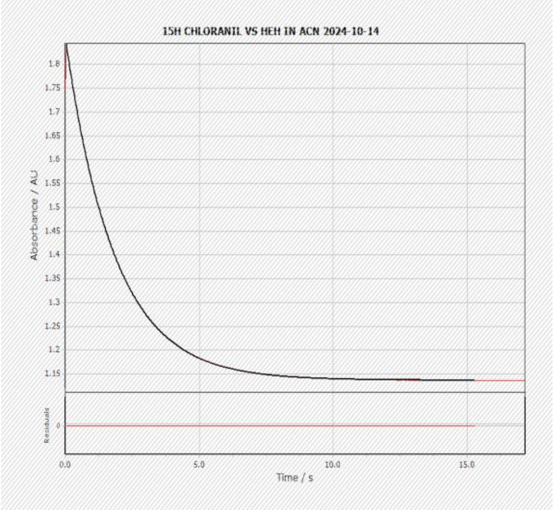
</td></tr>
<tr><td colspan="2">15℃ H</td></tr>
<tr><td>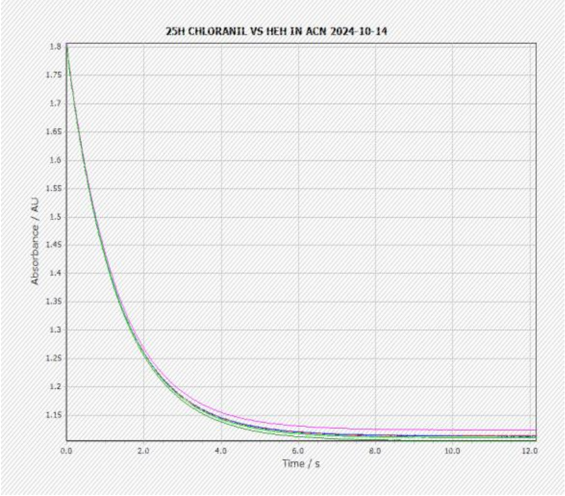
</td><td>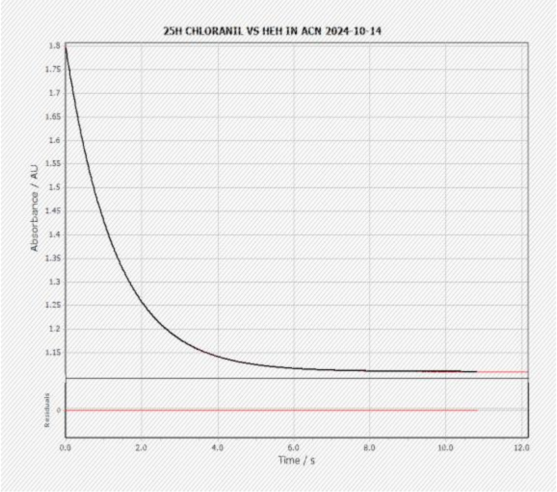
</td></tr>
<tr><td colspan="2">25℃ H</td></tr>
<tr><td>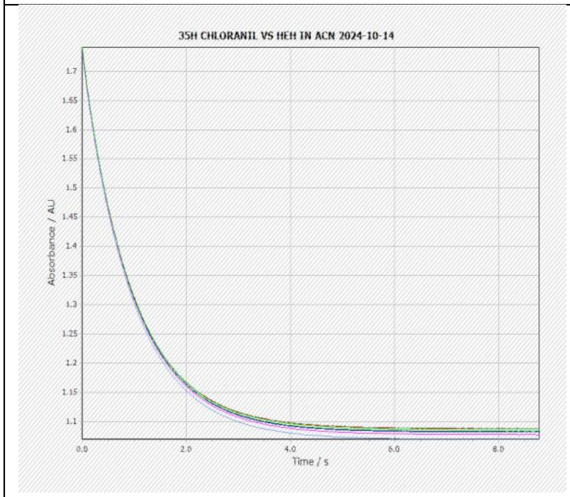
</td><td>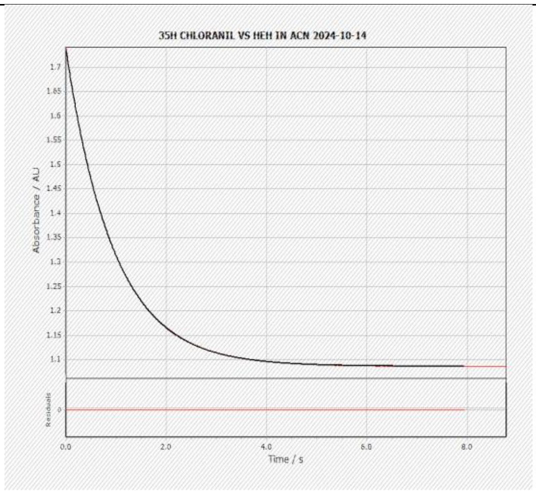
</td></tr>
<tr><td colspan="2">35℃ H</td></tr>
</table>

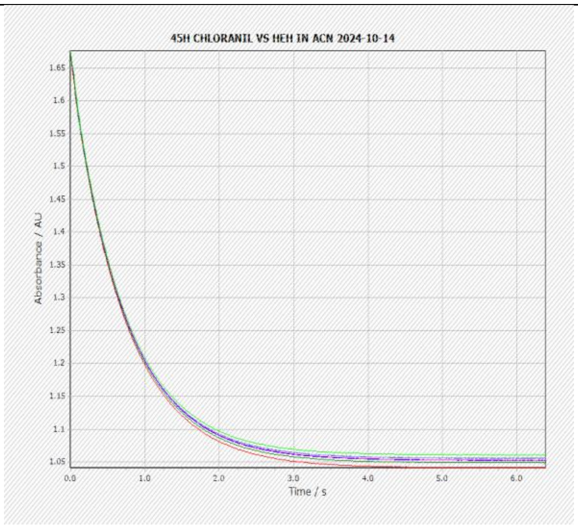

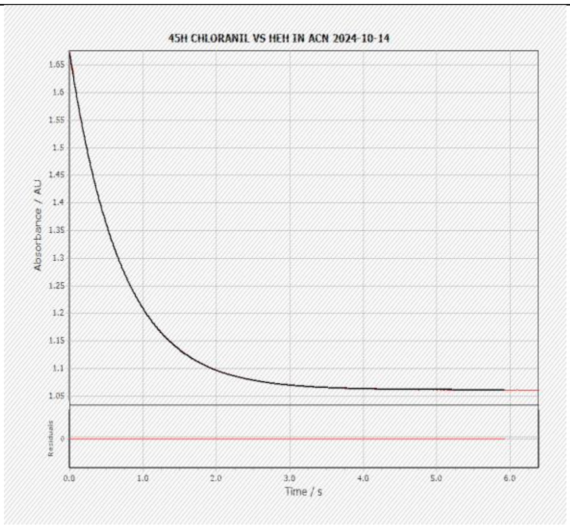

45°C H

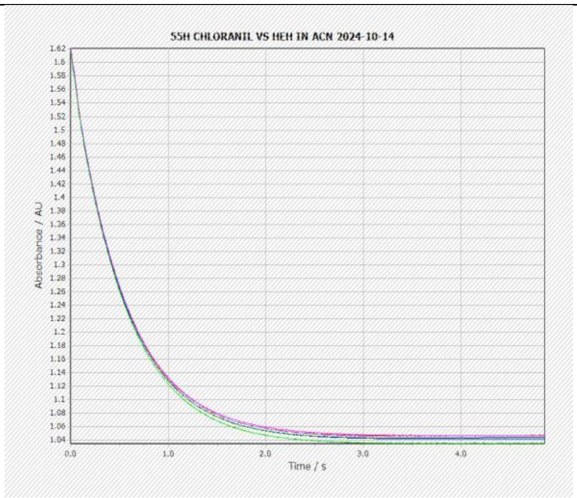

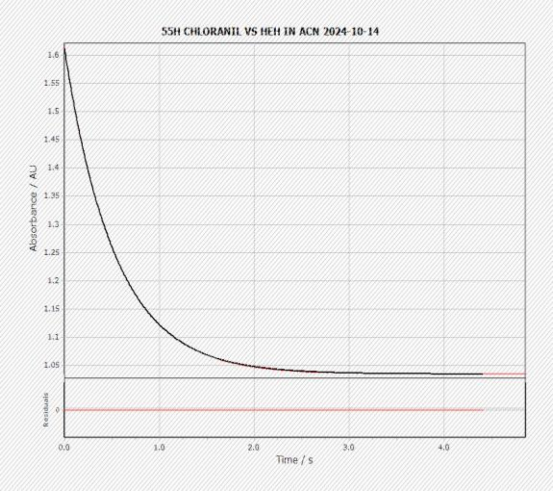

55°C H

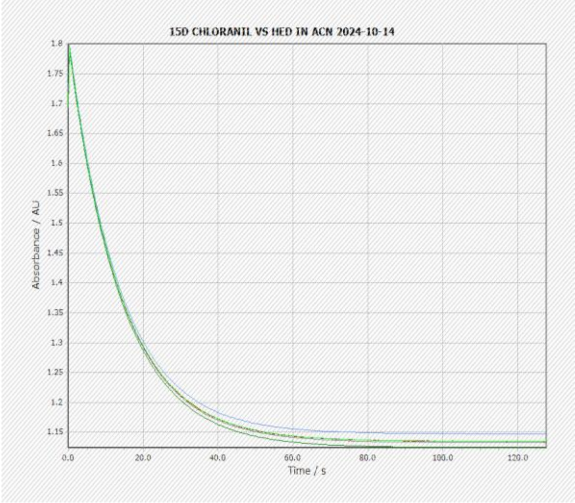

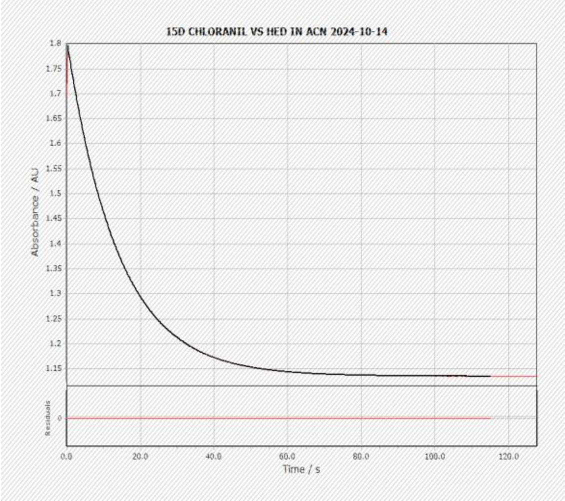

15°C D

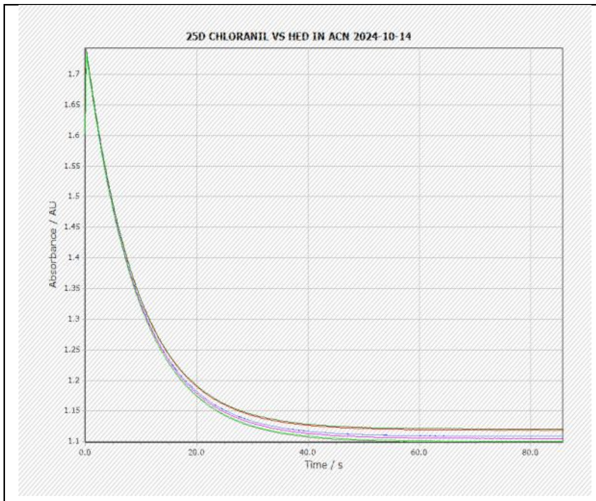

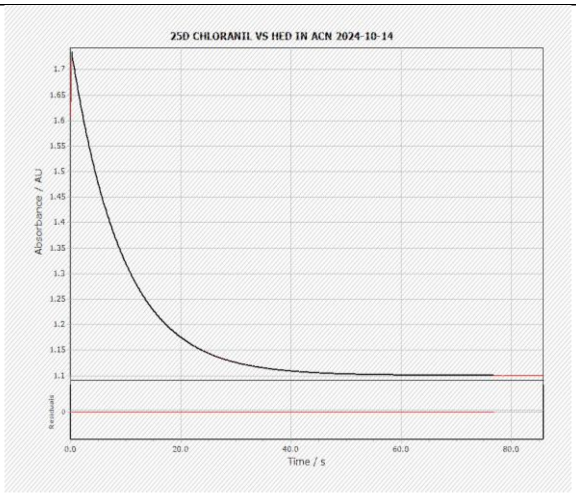

25°C D

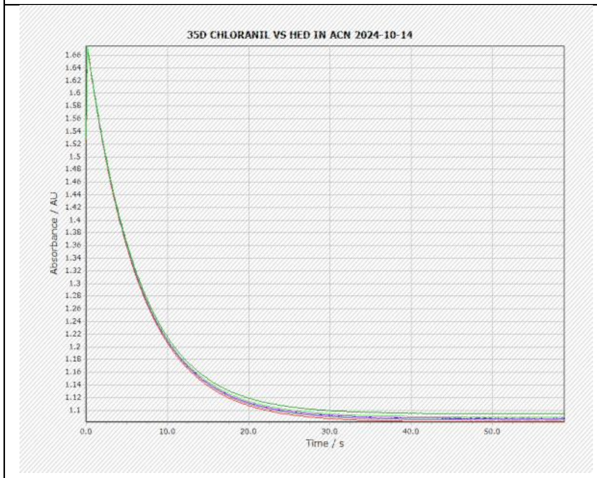

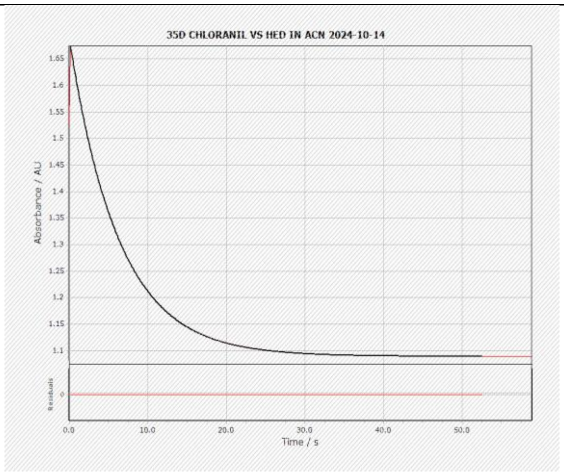

35°C D

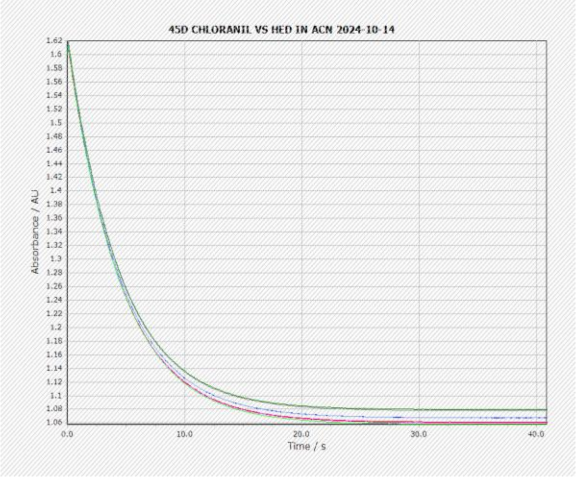

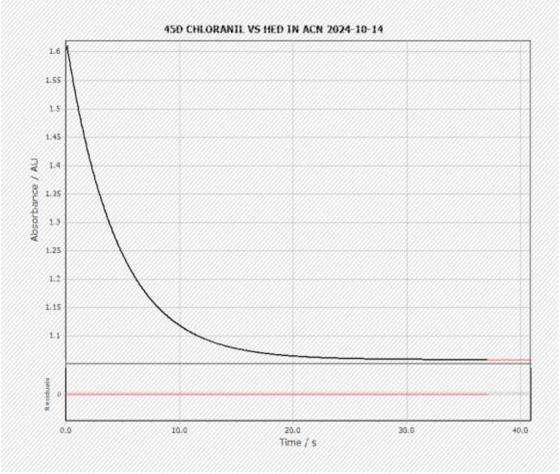

45°C D

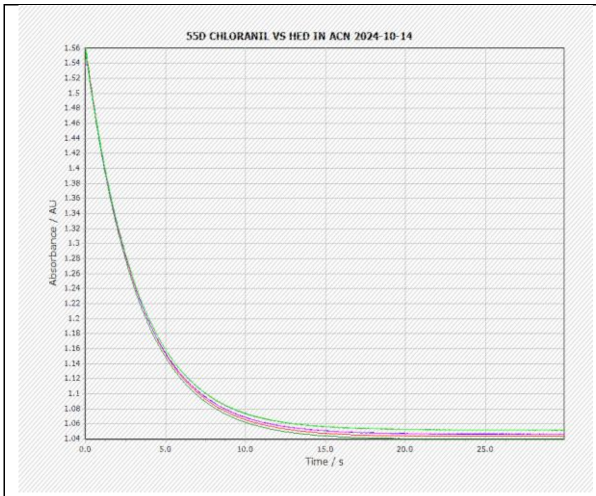	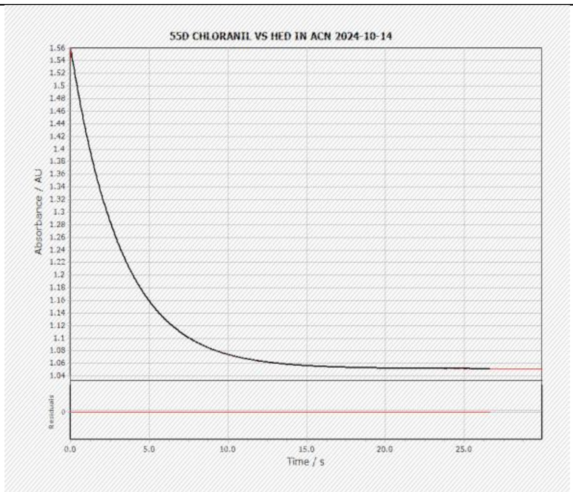
55°C D	

Day 3 data (October 17, 2024)

	Pseudo-first-order rate constants k^{pfo} (s^{-1})									
**Temp (°C)**	**Trial H1**	**Trial H2**	**Trial H3**	**Trial H4**	**Trial H5**	**Trial H6**	**Average k_H^{pfo} (s^{-1})**	**Stdev**	**k_{2H} [b] ($M^{-1}s^{-1}$)**	**Stdev [a]**
55	1.87972	1.88526	1.8813	1.88036	1.86121	1.91443	1.8837	0.0172356	4.28E+02	3.91717
45	1.41286	1.39862	1.4122	1.39909	1.39539	1.40324	1.4036	0.0073807	3.19E+02	1.67744
35	1.04703	1.0538	1.06061	1.05683	1.04991	1.04956	1.0530	0.0051036	2.39E+02	1.15990
25	0.76144	0.75446	0.76854	0.7654	0.76412	0.76657	0.7634	0.004995	1.74E+02	1.13522
15	0.54648	0.53787	0.53730	0.54088	0.54508	0.54023	0.5413	0.0037472	1.23E+02	0.85163
**Temp (°C)**	**Trial D1**	**Trial D2**	**Trial D3**	**Trial D4**	**Trial D5**	**Trial D6**	**Average k_D^{pfo} (s^{-1})**	**Stdev**	**k_{2D} [b] ($M^{-1}s^{-1}$)**	**Stdev [a]**
55	0.31064	0.31060	0.30785	0.31306	0.31009	0.30764	0.3100	0.00201648	7.05E+01	4.58E-01
45	0.2201	0.22183	0.21933	0.22125	0.22042	0.21988	0.2205	0.00092142	5.01E+01	2.09E-01
35	0.1542	0.15675	0.15681	0.15606	0.15505	0.15529	0.1557	0.00103000	3.54E+01	2.34E-01
25	0.10768	0.10833	0.10668	0.10827	0.10841	0.10858	0.1080	0.00071143	2.45E+01	1.62E-01
15	0.07107	0.07179	0.07123	0.07200	0.07177	0.07188	0.0716	0.00037892	1.63E+01	8.61E-02

[a] = (Stdev(for k^{pfo})/k^{pfo})*k_2; [b] = k^{pfo}/(2[Cl_4Q]/3)

<table>
<tr><td>Six kinetic runs together with the averaged data</td><td>First-order kinetic fit of the averaged data as an example</td></tr>
<tr><td>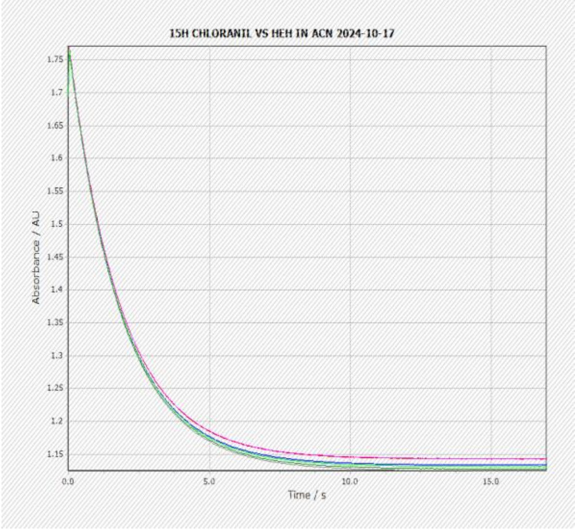
</td><td>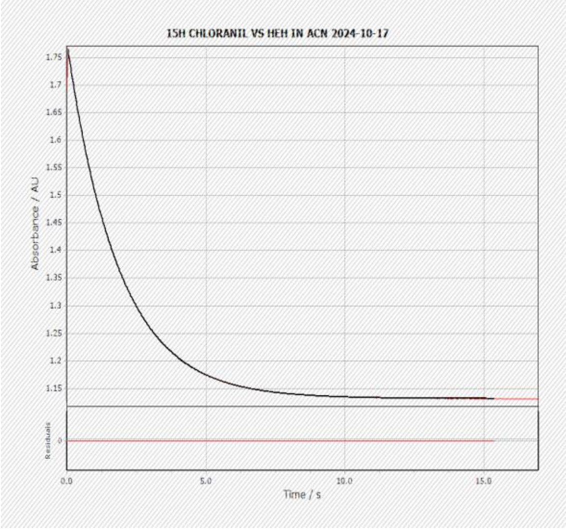
</td></tr>
<tr><td colspan="2">15℃ H</td></tr>
<tr><td>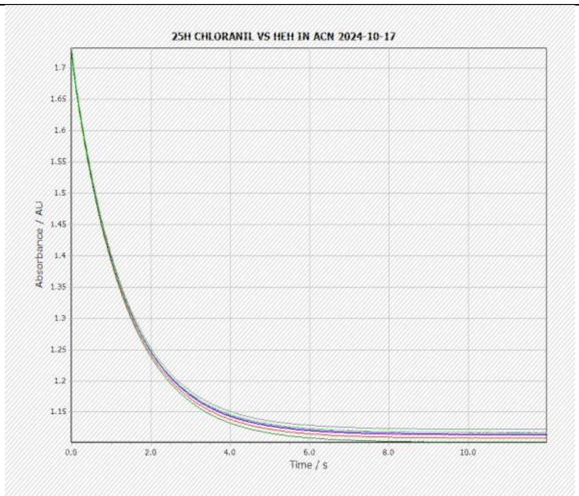
</td><td>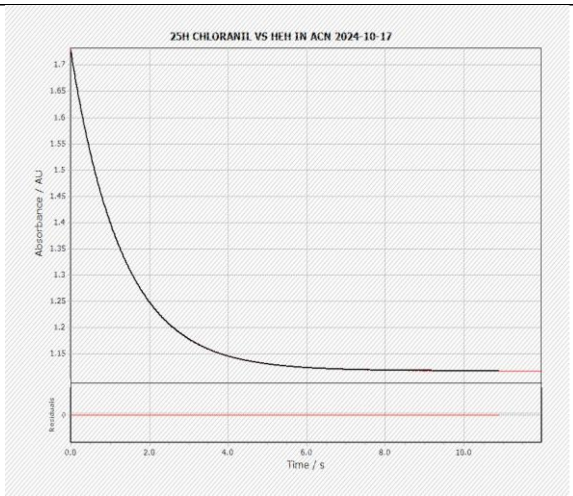
</td></tr>
<tr><td colspan="2">25℃ H</td></tr>
</table>

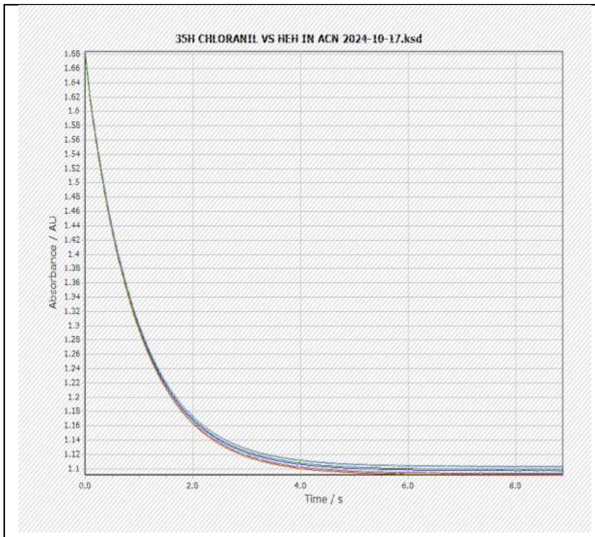
35H CHLORANIL VS HEH IN ACN 2024-10-17.ksd
Absorbance / AU
Time / s

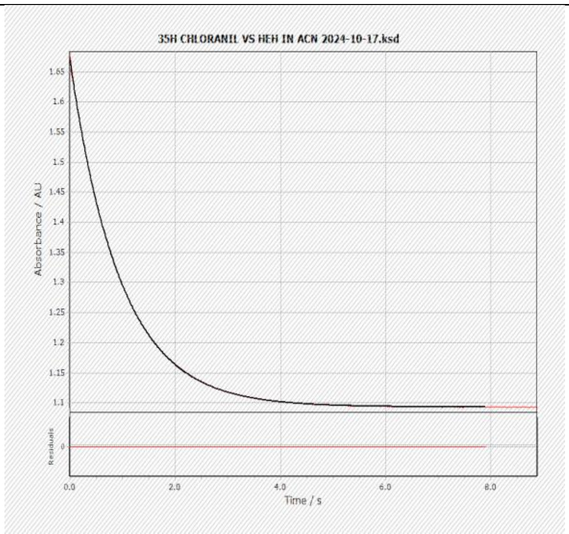
35H CHLORANIL VS HEH IN ACN 2024-10-17.ksd
Absorbance / AU
Residuals
Time / s

35℃ H

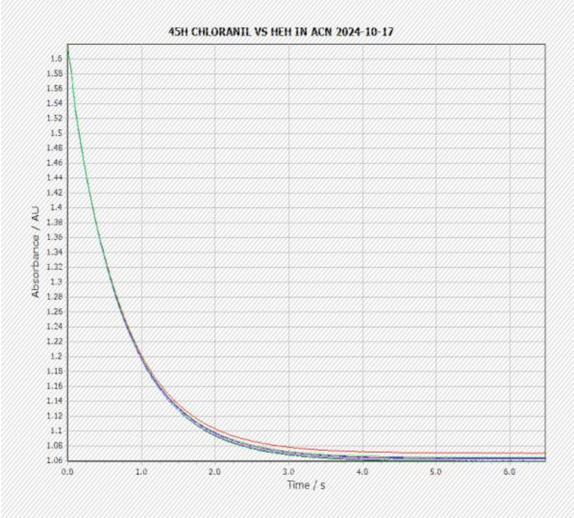
45H CHLORANIL VS HEH IN ACN 2024-10-17
Absorbance / AU
Time / s

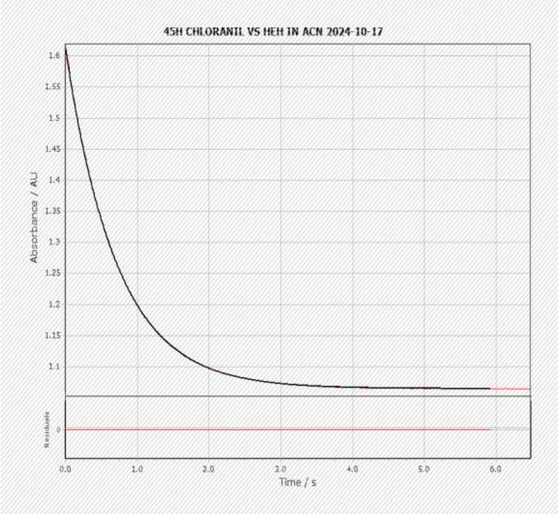
45H CHLORANIL VS HEH IN ACN 2024-10-17
Absorbance / AU
Residuals
Time / s

45℃ H

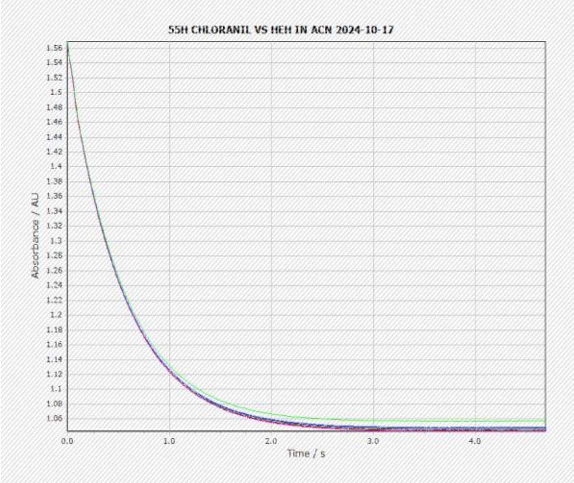
55H CHLORANIL VS HEH IN ACN 2024-10-17
Absorbance / AU
Time / s

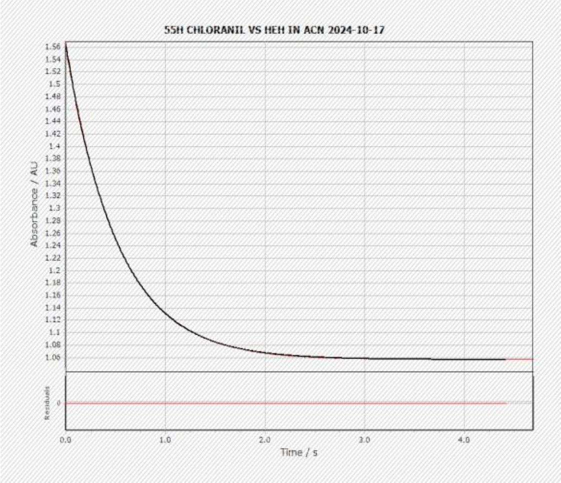
55H CHLORANIL VS HEH IN ACN 2024-10-17
Absorbance / AU
Residuals
Time / s

55℃ H

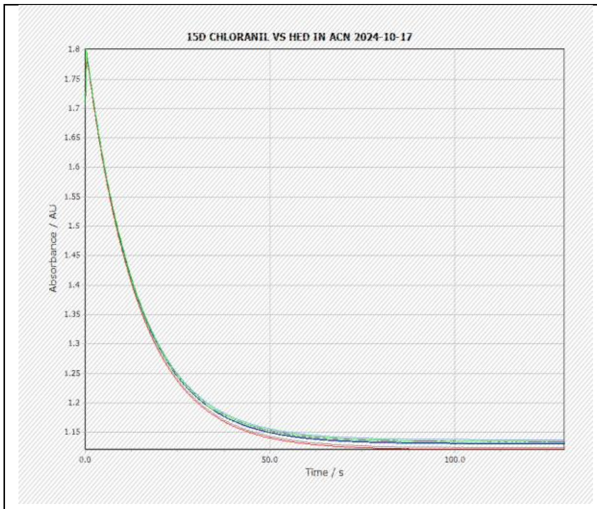

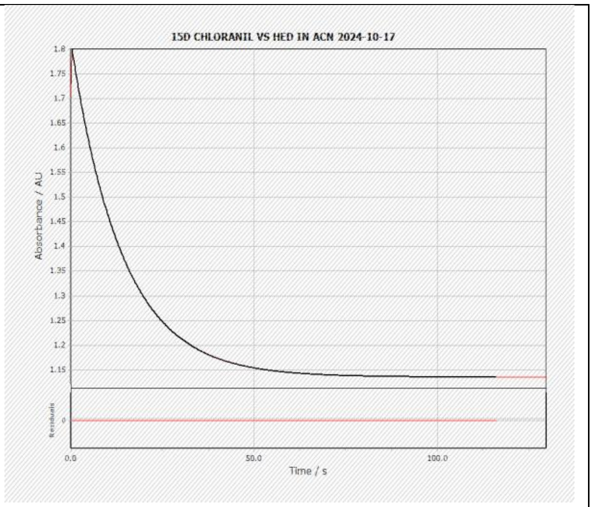

15°C D

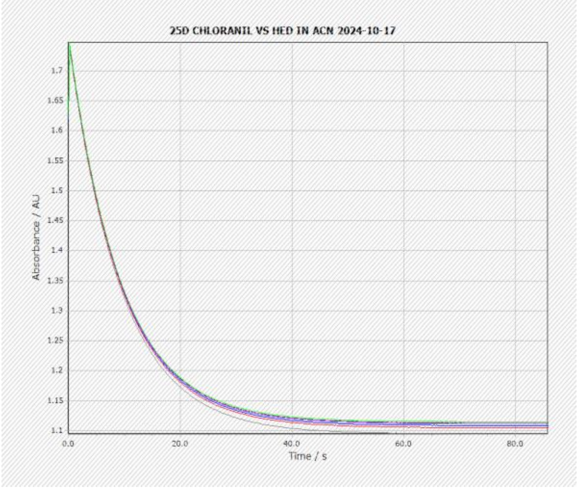

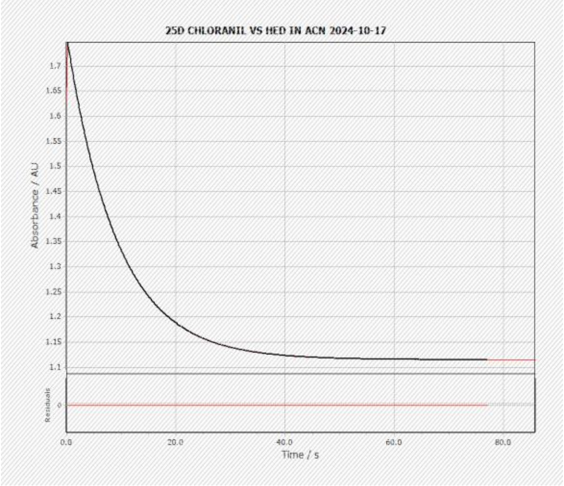

25°C D

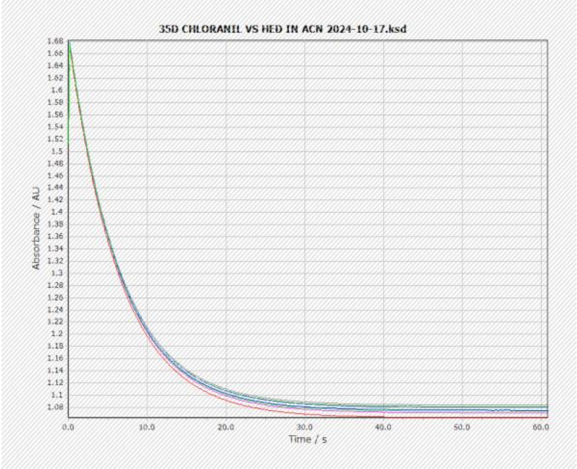

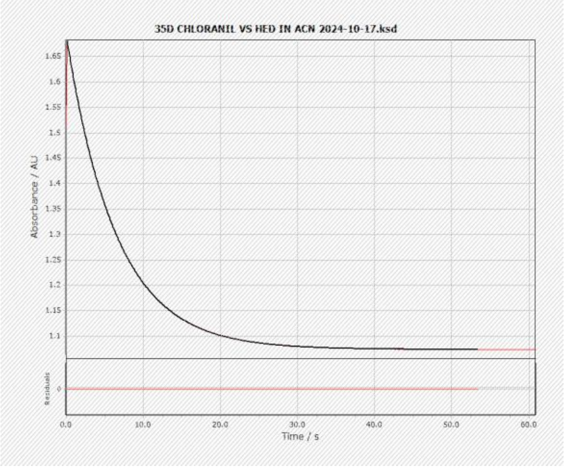

35°C D

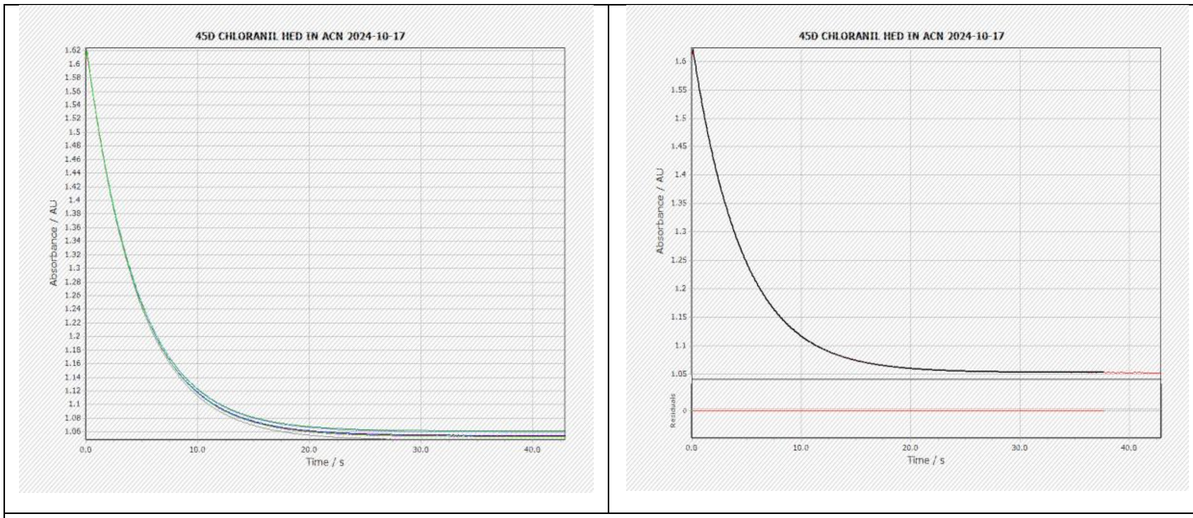

45°C D

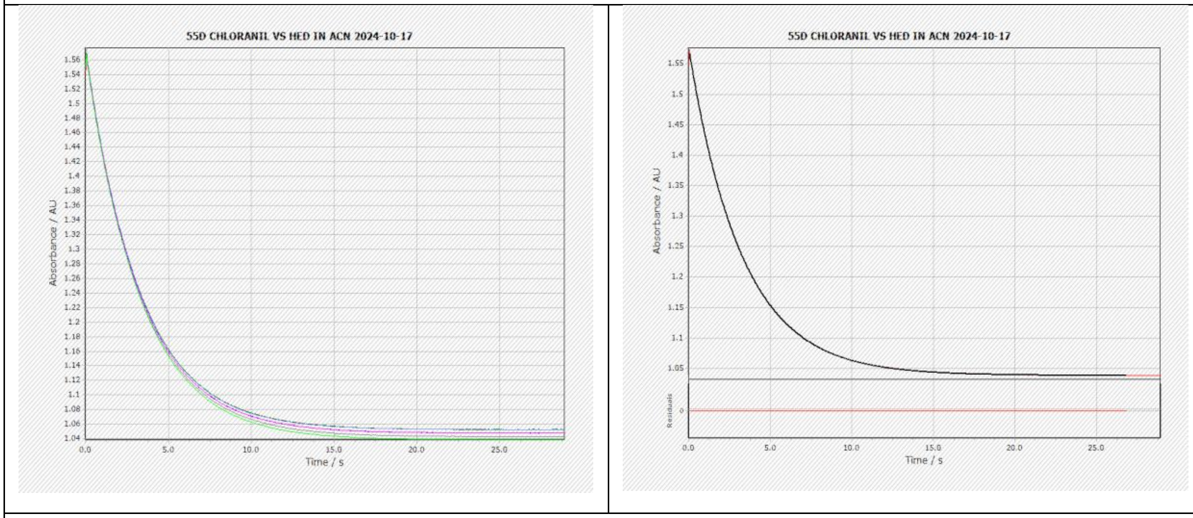

55°C D

Primary kinetic data for the rate constants in Table S7

Set 1 data (November 19, 2024)

	Pseudo-first-order rate constants k^{pfo} (s^{-1})									
**Temp (°C)**	**Trial H1**	**Trial H2**	**Trial H3**	**Trial H4**	**Trial H5**	**Trial H6**	**Average k_H^{pfo} (s^{-1})**	**Stdev**	**k_{2H} [b] (M^{-1}s^{-1})**	**Stdev [a]**
25	0.7594	0.75667	0.75751	0.76504	0.77076	0.75452	0.76065	0.00610444	1.73E+02	1.2665
**Temp (°C)**	**Trial D1**	**Trial D2**	**Trial D3**	**Trial D4**	**Trial D5**	**Trial D6**	**Average k_D^{pfo} (s^{-1})**	**Stdev**	**k_{2D} [b] (M^{-1}s^{-1})**	**Stdev [a]**
25	0.74345	0.74024	0.74689	0.75435	0.751040	0.75659	0.74876	0.00635671	1.70E+02	1.3288

[a] = (Stdev(for k^{pfo})/k^{pfo})*k_2; [b] = k^{pfo}/(2[Cl_4Q]/3)

Six kinetic runs together with the averaged data	First-order kinetic fit of the averaged data as an example
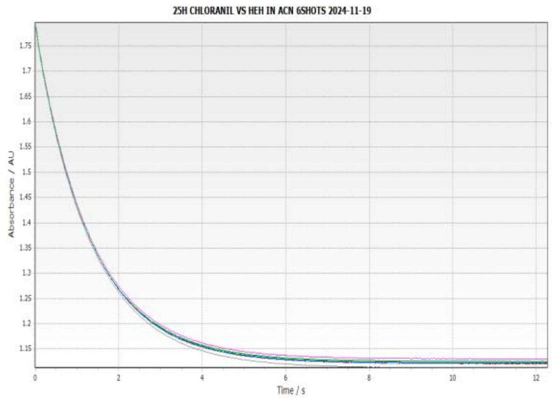	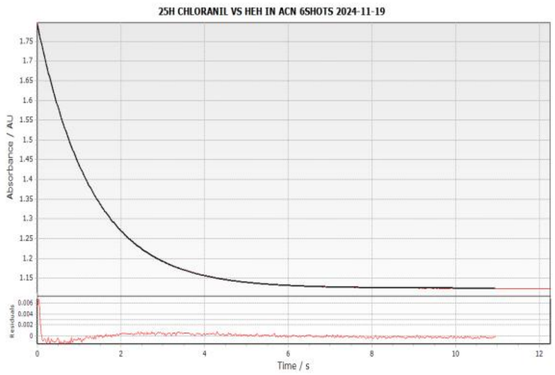
NH	
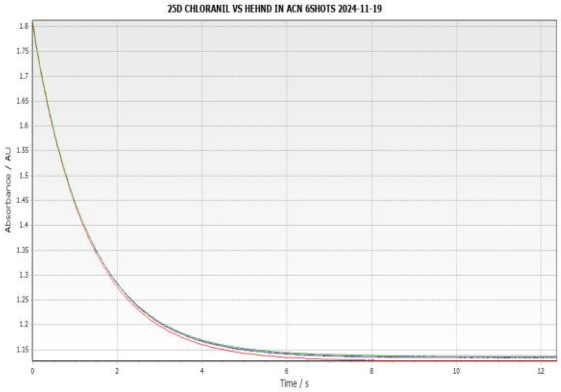	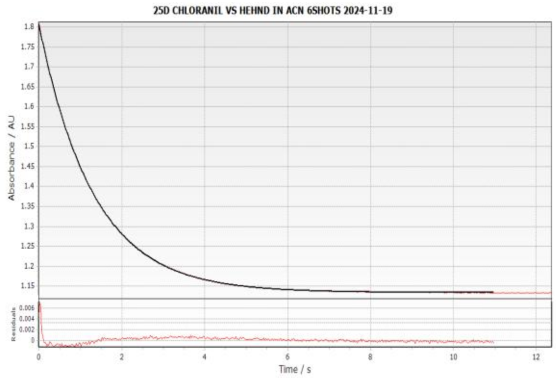
ND	

Set 2 data (November 19, 2024)

	Pseudo-first-order rate constants									
	k^{pfo} (s^{-1})									
**Temp (°C)**	**Trial H1**	**Trial H2**	**Trial H3**	**Trial H4**	**Trial H5**	**Trial H6**	**Average k_H^{pfo} (s^{-1})**	**Stdev**	**k_{2H} [b] (M^{-1}s^{-1})**	**Stdev [a]**
25	0.75812	0.76274	0.75307	0.7585900	0.7637600	0.76036	0.75944	0.00383	172.6	0.7951
**Temp (°C)**	**Trial D1**	**Trial D2**	**Trial D3**	**Trial D4**	**Trial D5**	**Trial D6**	**Average k_D^{pfo} (s^{-1})**	**Stdev**	**k_{2D} [b] (M^{-1}s^{-1})**	**Stdev [a]**
25	0.75516	0.75896	0.76161	0.7594600	0.7588300	0.75978	0.7589	0.00211	172.492	0.4391

[a] = (Stdev(for k^{pfo})/k^{pfo})*k_2; [b] = k^{pfo}/(2[Cl_4Q]/3)

Six kinetic runs together with the averaged data

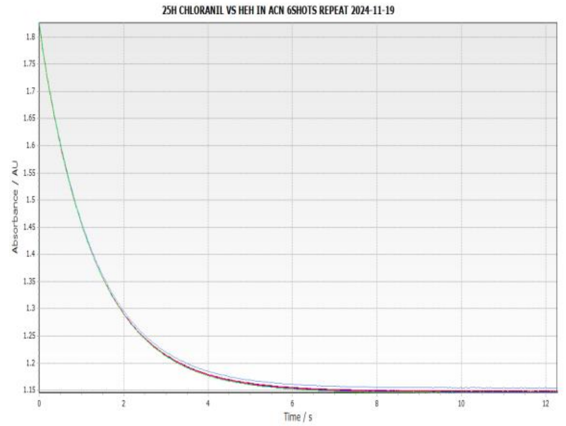

First-order kinetic fit of the averaged data as an example

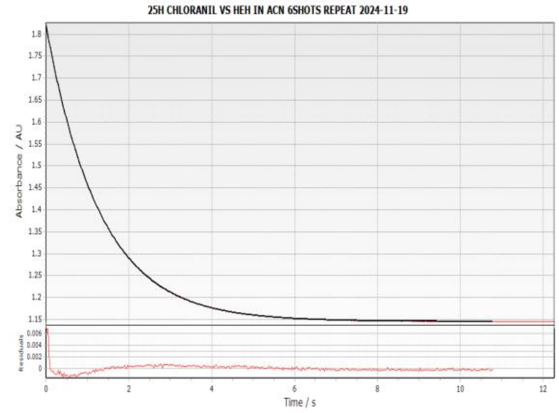

NH

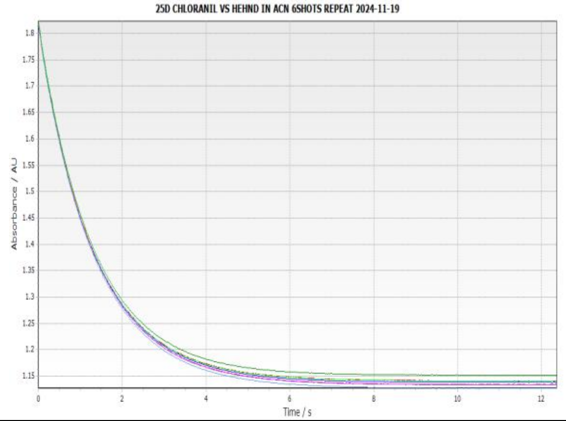

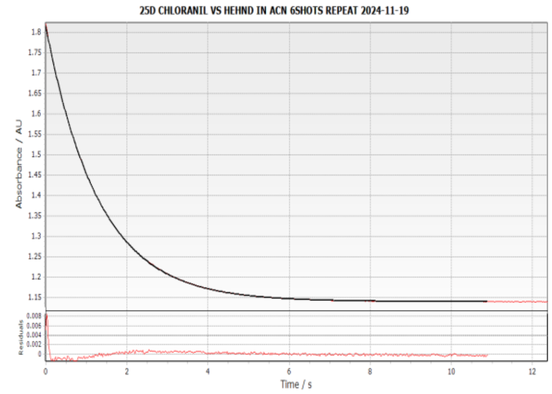

ND

Set 3 data (November 19, 2024)

Pseudo-first-order rate constants

	k^{pfo} (s^{-1})									
**Temp (°C)**	**Trial H1**	**Trial H2**	**Trial H3**	**Trial H4**	**Trial H5**	**Trial H6**	**Average k_H^{pfo} (s^{-1})**	**Stdev**	**k_{2H} [b] ($M^{-1}s^{-1}$)**	**Stdev [a]**
25	0.7709	0.7705	0.7705	0.775290	0.770310	0.7695	0.771203	0.00205145	175.273485	0.42561669
**Temp (°C)**	**Trial D1**	**Trial D2**	**Trial D3**	**Trial D4**	**Trial D5**	**Trial D6**	**Average k_D^{pfo} (s^{-1})**	**Stdev**	**k_{2D} [b] ($M^{-1}s^{-1}$)**	**Stdev [a]**
25	0.7649	0.7736	0.7602	0.756520	0.760720	0.76557	0.763623	0.00594785	173.55075	1.2340062

[a] = (Stdev(for k^{pfo})/k^{pfo})*k_2; [b] = k^{pfo}/(2[Cl_4Q]/3)

<table>
<tr><td>Three kinetic runs together with the averaged data</td><td>First-order kinetic fit of the averaged data as an example</td></tr>
<tr><td>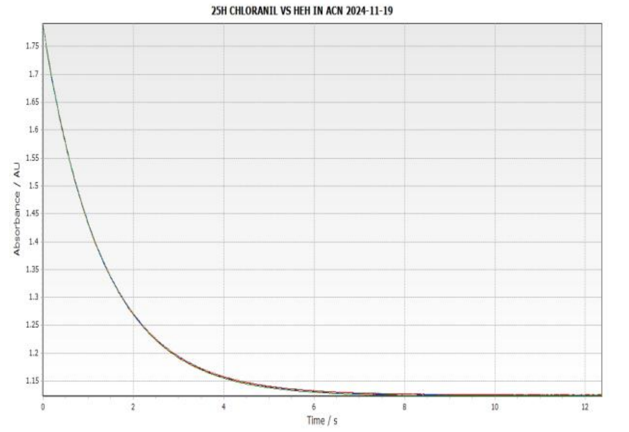
</td><td>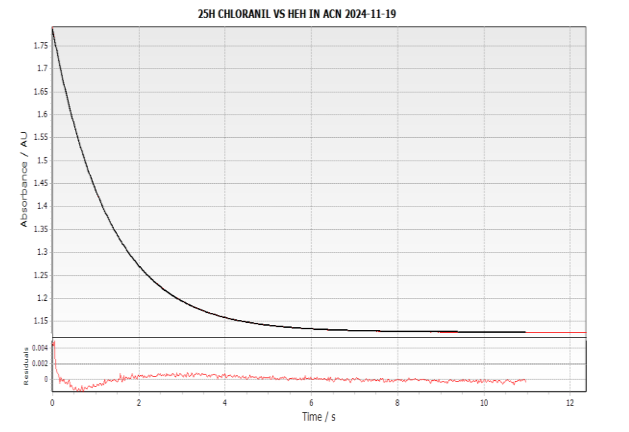
</td></tr>
<tr><td colspan="2">NH(Trails 1-3)</td></tr>
<tr><td>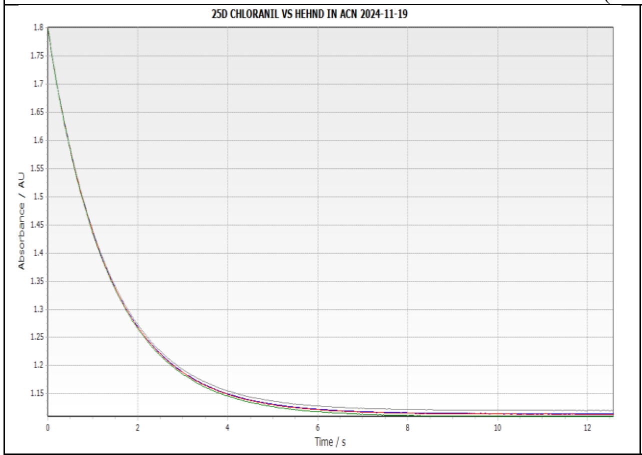
</td><td>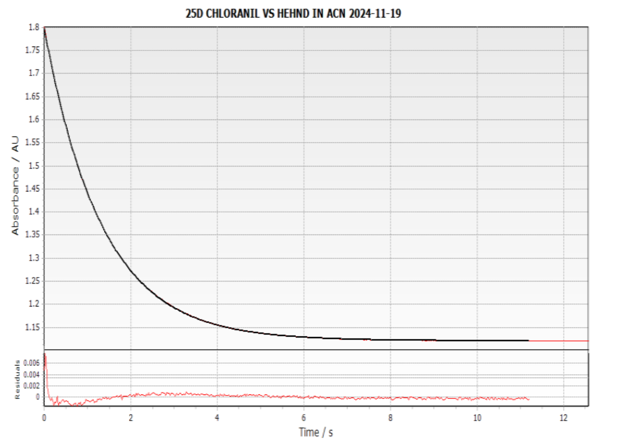
</td></tr>
<tr><td colspan="2">ND(Trails 1-3)</td></tr>
<tr><td>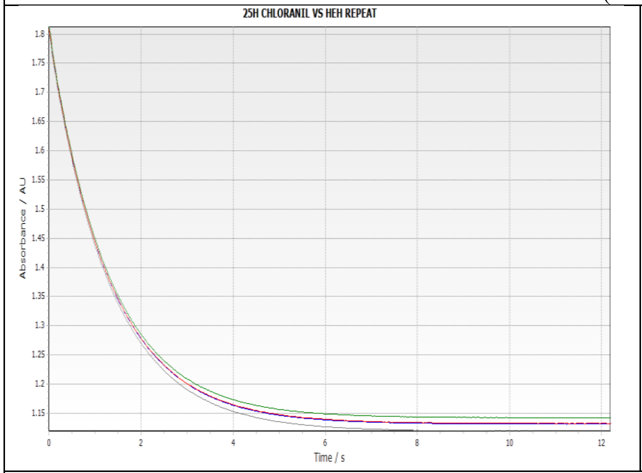
</td><td>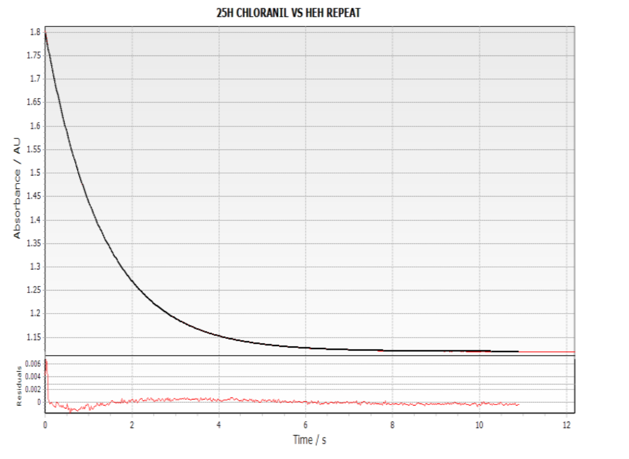
</td></tr>
<tr><td colspan="2">NH(Trails 4-6)</td></tr>
</table>

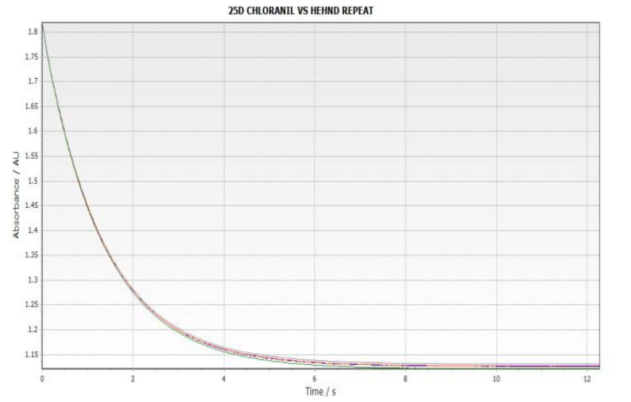
25D CHLORANIL VS HEHND REPEAT
Absorbance / AU
Time / s

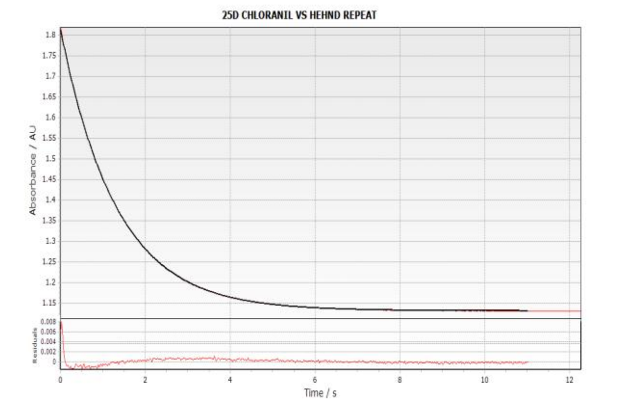
25D CHLORANIL VS HEHND REPEAT
Absorbance / AU
Residuals
Time / s

ND(Trails 4-6)
